# Cyanate Formation via Photolytic Splitting of Dinitrogen

**DOI:** 10.1021/jacsau.1c00117

**Published:** 2021-05-20

**Authors:** Bastian Schluschaß, Jan-Hendrik Borter, Severine Rupp, Serhiy Demeshko, Christian Herwig, Christian Limberg, Nicholas A. Maciulis, Jessica Schneider, Christian Würtele, Vera Krewald, Dirk Schwarzer, Sven Schneider

**Affiliations:** † University of Göttingen, Institute for Inorganic Chemistry, Tammannstraße 4, 37077 Göttingen, Germany; ‡ Department of Dynamics at Surfaces, Max Planck Institute for Biophysical Chemistry, Am Fassberg 11, 37077 Göttingen, Germany; § Theoretische Chemie, Technische Universität Darmstadt, Alarich-Weiss-Str. 4, 64287 Darmstadt, Germany; ∥ Institut für Chemie, Humboldt Universität zu Berlin, Brook-Taylor-Strasse 2, 12489 Berlin, Germany; ⊥ Department of Chemistry, Indiana University, 800 East Kirkwood Avenue, Bloomington, Indiana 47405-7102, United States

**Keywords:** Nitrogen Fixation, Nitride, N_2_ Splitting, Photochemistry, Carbonylation

## Abstract

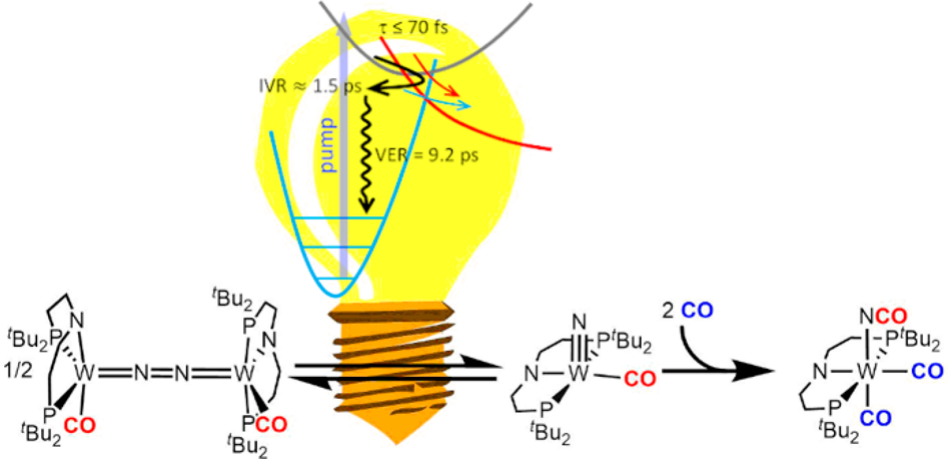

Light-driven N_2_ cleavage into molecular nitrides is an attractive strategy for synthetic nitrogen fixation. However, suitable platforms are rare. Furthermore, the development of catalytic protocols via this elementary step suffers from poor understanding of N–N photosplitting within dinitrogen complexes, as well as of the thermochemical and kinetic framework for coupled follow-up chemistry. We here present a tungsten pincer platform, which undergoes fully reversible, thermal N_2_ splitting and reverse nitride coupling, allowing for experimental derivation of thermodynamic and kinetic parameters of the N–N cleavage step. Selective N–N splitting was also obtained photolytically. DFT computations allocate the productive excitations within the {WNNW} core. Transient absorption spectroscopy shows ultrafast repopulation of the electronic ground state. Comparison with ground-state kinetics and resonance Raman data support a pathway for N–N photosplitting via a nonstatistically vibrationally excited ground state that benefits from vibronically coupled structural distortion of the core. Nitride carbonylation and release are demonstrated within a full synthetic cycle for trimethylsilylcyanate formation directly from N_2_ and CO.

## Introduction

After the seminal report of Laplaza and Cummins in 1995, the splitting of dinitrogen into molecular nitrido complexes has evolved as a synthetic strategy to nitrogen fixation at ambient conditions.^1−4^ Catalytic ammonia formation that commences with full N–N bond rupture, followed by proton-coupled electron transfer (PCET) steps, resembles the mechanism of the heterogeneously catalyzed *Haber–Bosch* process.^5,6^ Such a dissociative mechanism was recently proposed by Nishibayashi and co-workers for the currently most active class of homogeneous catalysts, which are Mo pincer complexes that mediate N_2_ fixation with activities up to TON_max_ = 4350 and TOF_max_ = 117 min^–1^ using SmI_2_/H_2_O as a PCET reductant.^7,8^ Alternatively, nitride formation potentially offers an entry to subsequent C–N bond formation.^3,4,9^ Several groups demonstrated the suitability of dissociative mechanistic scenarios, e.g., to synthesize organic nitriles from N_2_, within stepwise, cyclic reaction schemes (“synthetic cycles”).^10−13^ However, truly catalytic protocols that allow for the direct transformation of N_2_ to organic products remain unknown to date.

**Figure 1:**
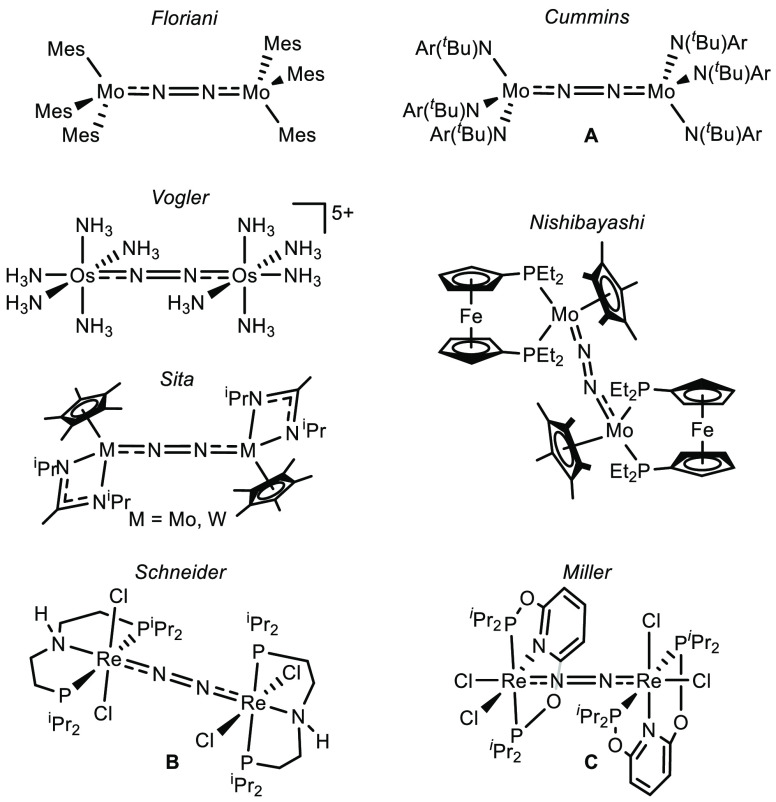
N_2_-bridged complexes that undergo photolytic cleavage into nitrides (Ar = C_6_H_3_-3,5-Me_2_).^15−21^

The thermochemical challenge of the dissociative approach to N_2_ fixation arises from the extraordinarily strong N–N triple bond (BDE = 941 kJ·mol^–1^),^3,4^ which needs to be counterbalanced by the formed M–N bonds. In consequence, C–N bond formation and N-transfer requires quite reactive reagents, such as strong electrophiles that are often incompatible with the reductive conditions of N_2_ activation. Photolytic N_2_ splitting could be an attractive alternative to circumvent the thermochemical constraints and drive endothermic N_2_ cleavage toward more reactive nitrides.^14^ However, platforms that were demonstrated to undergo photodriven N_2_ splitting into molecular nitrides are rare (Figure 1).^15−21^ For almost all of them, the underlying photophysics that enable light-driven N–N cleavage are yet to be systematically examined.^22−27^ Advances for both thermally and light driven N_2_ cleavage still suffer from a relatively poor understanding of structure–reactivity relationships.

**Figure 2:**
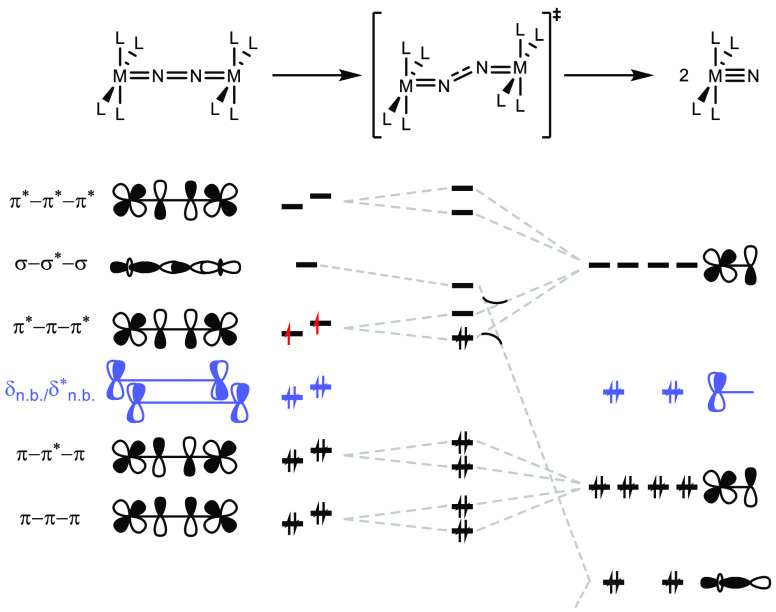
Qualitative molecular orbital correlation diagram for the splitting of μ^2^:η^1^:η^1^-N_2_ bridged complexes with *D*_4*h*_ symmetry into terminal nitrides.

In the past years, we examined thermally, electrochemically, and photochemically driven splitting of dinitrogen into molecular nitrides with group 6 and 7 pincer complexes.^20,28−32^ Like other systems that give terminal nitrides as N_2_ cleavage products,^2,7,33−36^ dinuclear, μ^2^:η^1^:η^1^-N_2_ bridged complexes were identified as key intermediates.^20,29−31^ Based on a covalent bonding model for the {MNNM} core,^37,38^ Cummins and co-workers qualitatively rationalized the N_2_ splitting reactivity of his *pseudo*-tetrahedral platform (Figure 1, **A**) by simple molecular orbital (MO) considerations.^2^ Transfer to idealized *D*_4*h*_ symmetry expands this picture to the emerging class of metal-pincer complexes that mediate N_2_ splitting (Figure 2).^3,4^ As a general feature, the precursors to N_2_ splitting exhibit electronic ground state configurations with 10 electrons in the {MNNM} π-MO manifold, such as [(N_2_){ReCl(PNP)}_2_] (**D**, π_1_^4^π_2_^4^δ_1_^2^δ_2_^2^π_3_^2^) or [(N_2_){MCl(HPNP)}_2_]^2+^ (π_1_^4^π_2_^4^δ_1_^1^δ_2_^1^π_3_^2^), respectively (M = Mo, W; PNP = N(CH_2_CH_2_P^*t*^Bu_2_)_2_).^28−31^ Full N–N cleavage is associated with an electronic rearrangement from the π*−π–π* to the N–N σ* MO via a *zigzag* transition state of the {MNNM} core. So far, this simple picture has proven fully consistent with computational electronic structure treatment of related systems.^7,21,28−32,35,36^ However, several aspects of thermally and photochemically driven N–N cleavage are yet to be addressed:

- Both strongly activated N_2_ complexes and the terminal nitride products can exhibit high degrees of covalent M–N bonding.^39,40^ The actual extent of charge transfer that is associated with cleavage of the N–N bond is surprisingly ill-defined. N_2_ splitting of complexes incorporating the common spectroscopic probe CO,^41^ which is sensitive to electronic changes at the metal center, has not been reported, to date.
- Platforms that undergo N–N splitting generally exhibit strongly σ- and/or π-donating ligands that mix with the frontier orbitals of the {MNNM} core.^29,42^ Auxiliary ligand effects, e.g. from π-acceptors, on the thermodynamics and kinetics of N_2_ cleavage need to be systematically explored.
- Only one photoactive complex (Figure 1, **A**) has been previously examined by transient absorption spectroscopy.^22^ The authors attributed photodriven N–N cleavage to vibrationally hot ground state reactivity. The applicability of these findings to other systems remains unknown.

We here report the synthesis of the CO-ligated complex [(N_2_){W(CO)(PNP)}_2_], which is the first compound that undergoes fully reversible splitting into molecular nitride complexes. The endothermic reaction can alternatively be driven by photolysis in the visible range. The photochemistry was examined by transient spectroscopy and quantum chemical treatment, and the reactivity could be utilized to drive a full synthetic cycle for isocyanate formation from N_2_ and CO.

## Results

### Syntheses and Electronic Structures of N_2_ Bridged Complexes

**Scheme 1:**
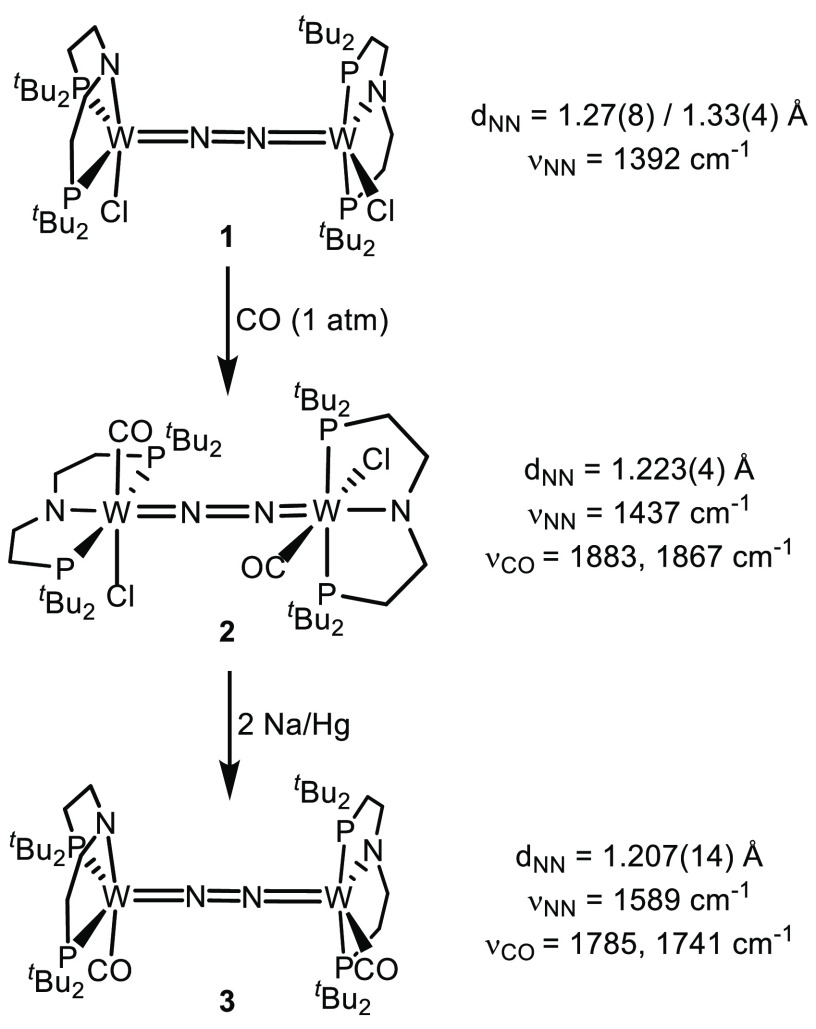
Synthesis and Selected Spectroscopic and Structural Parameters of Complexes **2** and **3**

**Figure 3:**
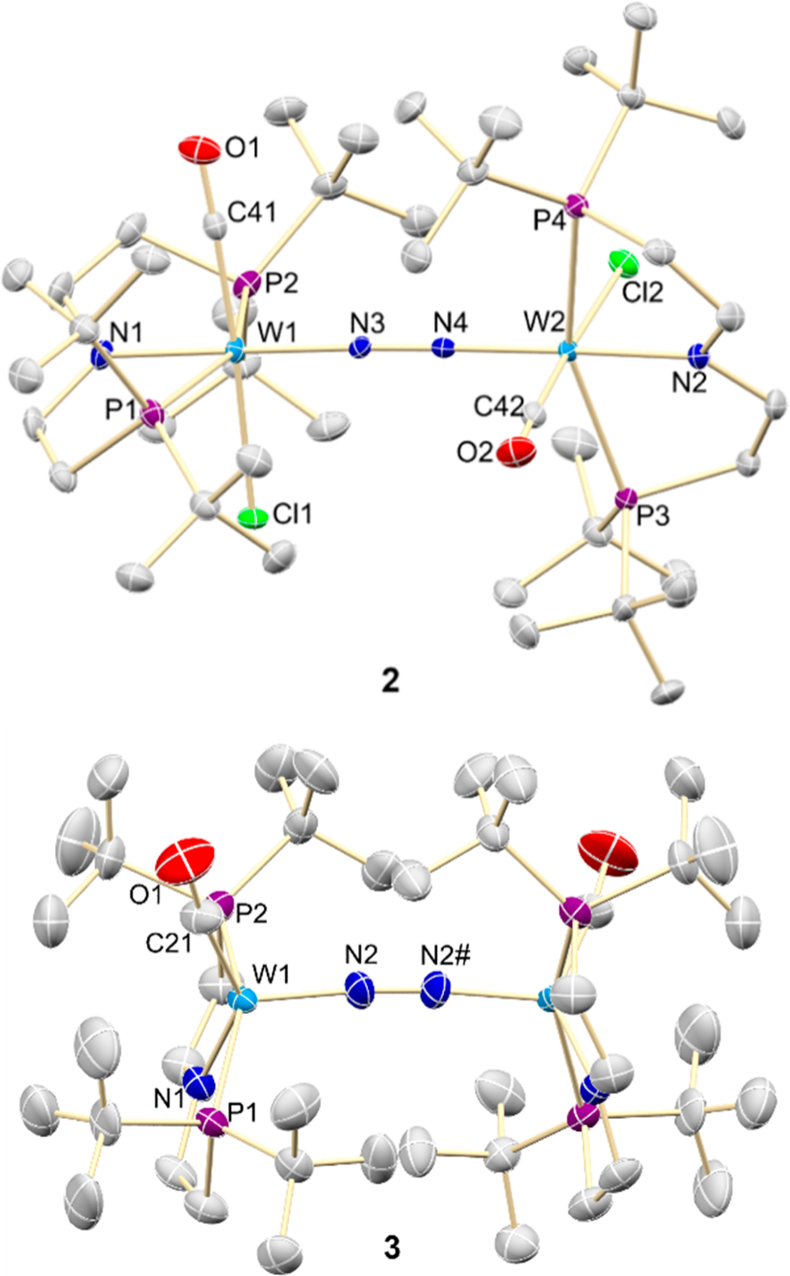
Molecular structures of **2** (*top*) and **3** (*bottom*) in the crystal from X-ray diffraction. Thermal ellipsoids are shown at 50% and 25% probability level, respectively. All hydrogen atoms were omitted for clarity. Selected bond lengths [Å] and angles [°] for **2** [N3–N4 1.223(4), W1–N1 2.032(3), W1–N3 1.870(3), W2–N2 2.023(3), W2–N4 1.872(3); W1–N3–N4 174.2(3), W2–N4–N3 174.2(3), N1–W1–N3 177.38(13), N2–W2–N4 174.40(13), P1–W1–P2 155.22(3), P3–W2–P4 157.19(3), C41–W1–Cl1 176.5(2), C42–W2–Cl2 176.09(15)] and **3** [N2–N2# 1.207(14), W1–C21 1.956(14)/1.97(2), W1–N1 2.043(10)/2.04(2), W1–N2 1.869(7), W1–P1 2.485(3)/2.398(14), W1–P2 2.435(4)/2.517(17); C21–W1–N1 140.9(9)/156(4), P1–W1–P2 154.79(13)/151.7(6), W1–N2–N2# 173.7(7)].

The reaction of [(N_2_){WCl(PNP)}_2_] (**1**)^31^ with CO (1 atm) results in quantitative formation of [(N_2_){WCl(CO)(PNP)}_2_] (**2**) (Scheme 1) within 20 min. Longer reaction times lead to loss of N_2_ and formation of [WCl(CO)_2_(PNP)] (*vide infra*). The ^1^H NMR spectrum of complex **2** features four signals for the ^*t*^Bu-groups as expected for a *C*_2_ symmetric structure, like that of parent **1**. Both sets of phosphorus atoms incidentally coincide as one ^31^P{^1^H} NMR resonance (δ_P_ = 65.9 ppm), which was confirmed by ^31^P HMBC spectroscopy. Conservation of the N_2_-bridge is evidenced by the ^15^N{^1^H} NMR spectrum of a ^15^N_2_ labeled sample (δ_N_ = −0.69 ppm; ^1^*J*_NW_ = 70 Hz). The idealized *C*_2_ symmetric molecular structure was also found in the solid state (Figure 3). In comparison with **1**, CO coordination results in slight contraction of the N–N bond (Δ*d*_NN_ = −0.08 Å)^43^ and a bathochromic shift of the N–N stretching frequency (Δν = +45 cm^–1^), which is attributed to competing backbonding to the CO and N_2_ ligands, respectively. All attempts to obtain analogous isonitrile complexes resulted in W–N_2_ dissociation.

Reduction of **2** with Na/Hg or CoCp*_2_ (2 equiv), respectively, gives deep red [(N_2_){W(CO)(PNP)}_2_] (**3**) in isolated yields up to 60% (Scheme 1). In the solid state, the structure of complex **3** resembles that of **1**, where Cl is replaced by CO (Figure 3). The pyramidally coordinated tungsten ions (τ_5_ = 0.23)^44^ are linearly bridged by the N_2_ ligand in the apical sites. As was found for the chloro analogues [(N_2_){MCl(PNP)}_2_] (M = W (**1**), Mo, Re),^29−31^ the two {W(CO)(PNP)} fragments are twisted with respect to each other by about 87° presumably due to the steric bulk of the ^*t*^Bu-substituents.

**Scheme 2:**
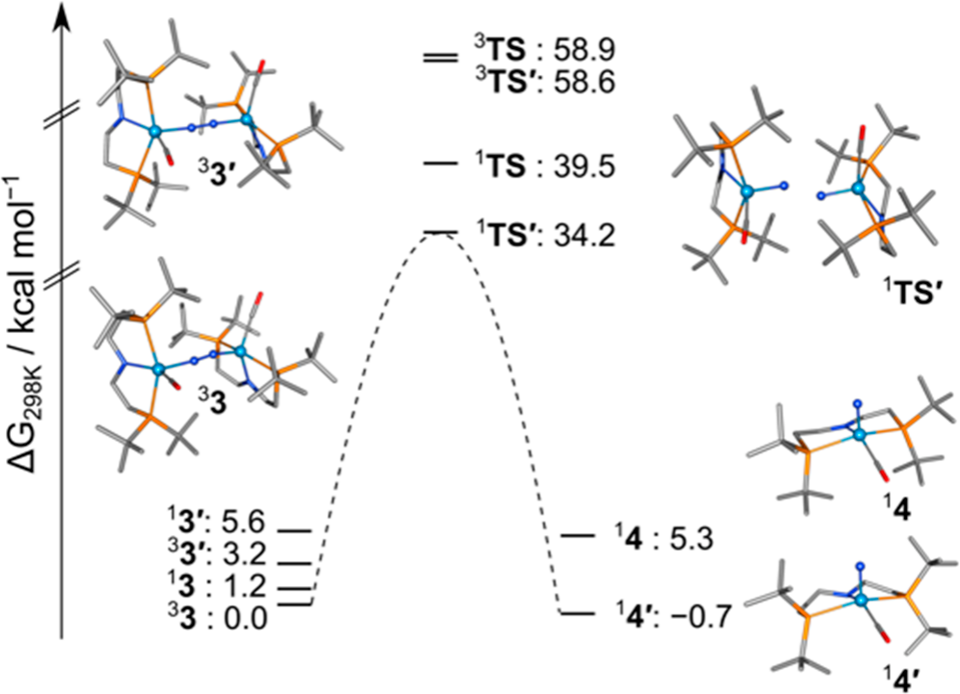
Computed Energy Profile for the Thermal Splitting of **3** into **4** at Room Temperatures All values are given in kcal·mol^–1^ referenced to the triplet ground-state and are not drawn to scale.

The approximate *C*_2_ symmetry of **3** in the solid state is in line with the number of ^1^H NMR resonances in solution. All signals are sharp, but paramagnetically shifted over a wide range (Δδ = 31 ppm). The absence of a ^31^P{^1^H} NMR signal further indicates an open-shell ground state. This interpretation was confirmed by SQUID magnetometry. The magnetic moment at room temperature (μ_eff_ = 2.3 ± 0.1 μ_B_) supports two unpaired electrons with considerable orbital contributions. Below 150 K, the χ_M_*T* vs *T* curve features temperature-independent susceptibility. The magnetic data could be fitted to a zero-field splitting (ZFS) spin-Hamiltonian (*S* = 1, *g*_av_ = 1.74) with large axial ZFS (*D* = 407 cm^–1^), which is in line with a triplet ground state that is energetically well separated due to large spin–orbit coupling (SOC).^17,45−48^ DFT computations with the PBE functional confirmed the triplet ground state of complex **3**. However, the corresponding closed-shell solution was found only 1.1 kcal·mol^–1^ higher in energy suggesting multireference character of the ground-state wave function, which is supported by the magnetic properties yet not sufficiently expressed by DFT computations. Note that a similar spin state splitting was found with hybrid functionals, like PBE0 (2.0 kcal·mol^–1^), suggesting that the spin state energetics are not very sensitive to the extent of exact exchange admixture, as was previously found by Harvey and Poli for tungsten complexes.^49,50^ For both spin states, additional conformers of the pincer ligand were found close in energy (see Scheme 2 and the SI), as an expression of the high flexibility of the saturated aliphatic backbone. The computed lowest conformer of **3** closely resembles the experimental structure in the crystal, while a different conformer (**3′**) was found 1.1 and 5.3 kcal·mol^–1^ higher in free energy in the triplet and singlet states, respectively.

The reduced complex **3** exhibits a lower degree of N_2_ activation than both parent complexes **1** and **2** (Scheme 1) as judged by the shorter N–N bond (**3***d*_NN_ = 1.207(14) Å) and higher energy of the N–N stretching vibration (**3** ν_NN_ = 1589 cm^–1^). On first sight, this might seem counterintuitive when comparing the bathochromically shifted CO stretching vibrations of **3** (ν_CO_ = 1785, 1741 cm^–1^) vs **2** (ν_CO_ = 1883, 1867 cm^–1^).^51^ However, according to the qualitative electronic structure considerations (Figure 2), complexes **1** and **2** both exhibit {π^8^δ^4^} closed-shell configurations of the {WNNW} core.^3,4^ The *S* = 1 ground state of **3** is in line with the population of two orthogonal, nearly degenerate {π*−π–π*}-MOs upon reduction. Their N–N bonding character reduces the degree of N_2_ activation, which comes closer to, e.g., the {π^10^δ^4^} complex **D** (*d*_NN_ = 1.202(10) Å) or complex **A** (*d*_NN_ = 1.212(2)/1.217(2) Å, ν_NN_ = 1630 cm^–1^).^2,16,30^ Note that the different symmetry of **A** (*S*_6_) leads to a {π^10^} configuration with closely related N_2_ bonding that results from overall 10 electrons in the π-MO manifold and high-lying, vacant d-orbitals of δ symmetry.^4^ These qualitative electronic structure considerations are fully corroborated for **3** by the DFT computations. Importantly, the DFT results show significant backdonation from the δ orbitals to CO and, in addition, admixture of CO character in the π-manifold of the {WNNW} unit (see the SI). Based on this picture, the significant reduction of the degree of N_2_ activation is rationalized as an expression of a high degree of covalency in W–N bonding.

### Thermally Driven Splitting of N_2_

**Figure 4:**
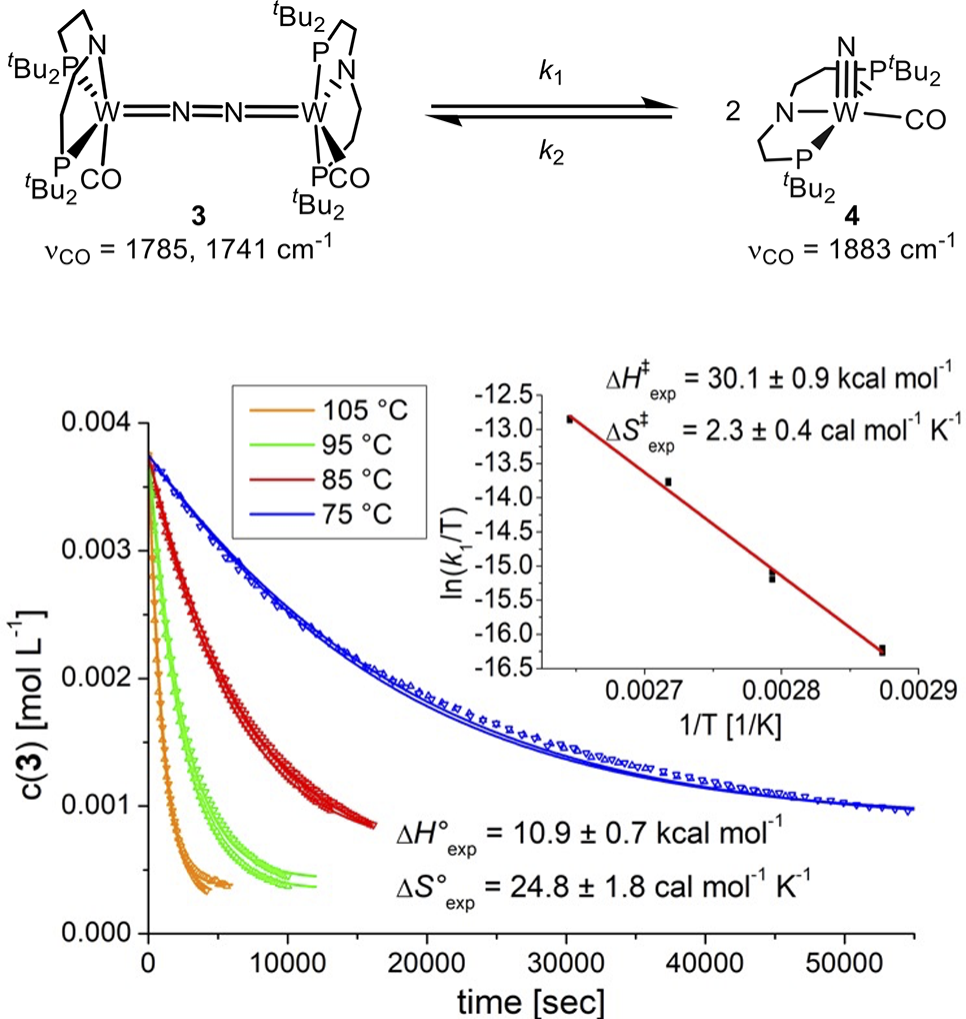
Concentration vs time plot for the thermal dissociation of **3** at different temperatures. The solid lines represent the results from fitting to the kinetic model. (*inset*) Eyring plot for the conversion of **3** into **4** (*R*^2^ = 0.995).

While **3** is stable at room temperature in solution for several days, heating (*T* = 80 °C) over several hours affords the pale blue nitrido complex [W(N)(CO)(PNP)] (**4**, Figure 4). NMR spectra of the diamagnetic N_2_ cleavage product **4** are in agreement with a square-pyramidal, *C*_s_ symmetric structure in solution. N_2_ splitting was confirmed by thermolysis of a ^15^N_2_ labeled sample (δ_N_ = 447 ppm). The W≡N stretching vibration (ν_WN_) was found at 998 cm^–1^ (ν_WN_(^15^N-**4**) = 973 cm^–1^), which is close to values found for related tungsten nitrido complexes.^31,52^

N_2_ splitting is associated with a distinct hypsochromic shift of the CO stretching frequency (ν_CO_ = 1883 cm^–1^) with respect to parent **3** (ν_CO_ = 1785, 1741 cm^–1^). The spectroscopic probe therefore supports a significant degree of metal to nitrogen charge transfer reflecting a reductive nature of N–N bond cleavage that leads from the N_2_ bridged {π^10^δ^4^} triplet species to the closed-shell terminal nitrides.

The N_2_ splitting reaction was monitored by ^1^H NMR spectroscopy at four different temperatures between 75 and 105 °C (Figure 4). Interestingly, reaction progress terminates prior to full conversion, suggesting slow equilibration of forward N_2_ splitting and reverse nitride coupling.^53−60^ This interpretation is supported by a control experiment which proved the formation of N_2_ bridged complex **3** by ^1^H NMR spectroscopy upon prolonged heating of independently prepared **4** under the exclusion of light. The kinetic data for the splitting of **3** could be fitted to an equilibrium model affording both thermodynamic and kinetic parameters by van’t Hoff and Eyring analyses, respectively. The equilibrium data (Δ*H*°_exp_ = 10.9 ± 0.7 kcal·mol^–1^, Δ*S*°_exp_ = 24.8 ± 1.8 cal·mol^–1^·K^–1^; Δ*G*°_exp_ = +3.6 kcal·mol^–1^) show that endothermic N_2_ splitting is entropically driven at higher temperatures. Furthermore, the forward activation parameters (Δ*H*^‡^_exp_ = 30.1 ± 0.9 kcal·mol^–1^; Δ*S*^‡^_exp_ = +2.3 ± 0.4 cal·mol^–1^·K^–1^; Δ*G*^‡^_298_ = 29.4 kcal·mol^–1^) confirm a prohibitively high kinetic barrier for either direction at room temperature. An almost identical entropy of activation was reported for the cleavage of complex **A** (Δ*H*^‡^_exp_ = 23.3 ± 0.3 kcal·mol^–1^; Δ*S*^‡^_exp_ = +2.3 ± 1.1 cal·mol^–1^K^–1^), which proceeds via the *zigzag* transition state described above. Nishibayashi and co-workers previously reported the photolytic splitting of an N_2_ bridged complex and reverse N–N coupling upon oxidation of the resulting molecular nitride.^18^ However, the thermal interconversion of **3** and **4** represents the first example of fully reversible N_2_ splitting and nitride coupling, without the addition of external redox reagents.

Thermal N_2_ splitting was examined computationally by DFT (Scheme 2), corroborating the equilibrium found for the N–N splitting reaction (Δ*G*°_DFT_ = −0.7 kcal·mol^–1^). Notably, the computed minimum structure of the nitride product resembles the pincer conformation of dimer **3′** with increased pyramidalization of the PNP nitrogen atom in comparison to **3**. The higher stability of this conformation in **4** is attributed to competing π-donation of the amide and nitride ligands. The transition state (**TS**) for splitting of complex **3** is found at considerably lower energy on the singlet than on the triplet surface (^1^**TS** Δ*H*^‡^_calc,S_ = 37.6 kcal·mol^–1^, ν_img_ = −357 cm^–1^; ^3^**TS** Δ*H*^‡^_calc,T_ = 59.1 kcal·mol^–1^, ν_img_ = −161 cm^–1^). Closer agreement with experiment is obtained for the activation barrier of the singlet conformer **3′** (**1TS′** Δ*H*^‡^_calc,S_ = 33.6 kcal·mol^–1^, ν_img_ = −368 cm^–1^), suggesting facile conformational rearrangement of the pincer backbone with negligible kinetic impact on route to the singlet transition state. All TS structures exhibit *zigzag* distorted {WNNW} cores with evolving W–N multiple bond character as indicated by bond shortening (^3^**3**_DFT_ 1.885 Å; ^1^**TS** 1.753 Å; ^3^**TS** 1.752 Å; ^3^**3′**_DFT_ 1.931 Å; ^1^**TS′** 1.755 Å). A considerably smaller degree of distortion from the ground state geometry is required on the singlet surface, as expressed by the shorter N–N distance (^1^**TS** 1.809 Å, ^1^**TS′** 1.789 Å vs ^3^**TS** 1.981 Å) and smaller W–N–N angle (^1^**TS** 151°, ^1^**TS′** 152° vs ^3^**TS** 160°), which is in line with the lower kinetic barrier. The preference for the characteristic in plane *zigzag*^1^**TS** reflects previous computational studies for systems that undergo N–N cleavage or reverse nitride coupling, such as {π^10^} complex **A** (^1^**TS***d*_MoN_ = 1.760 Å, *d*_NN_ = 1.623 Å, θ_MoNN_ = 148°), the computational model complex [N_2_{W(NH_2_)_3_}_2_] (^1^**TS***d*_WN_ = 1.781 Å, *d*_NN_ = 1.458 Å, θ_WNN_ = 145°), or [(N_2_){WCl(HPNP)}_2_]^2+^ (**^1^TS***d*_WN_ = 1.764/1.740 Å, *d*_NN_ = 1.598 Å, θ_WNN_ = 140.67°/153.54°), respectively.^31,57,61,62^

**Figure 5:**
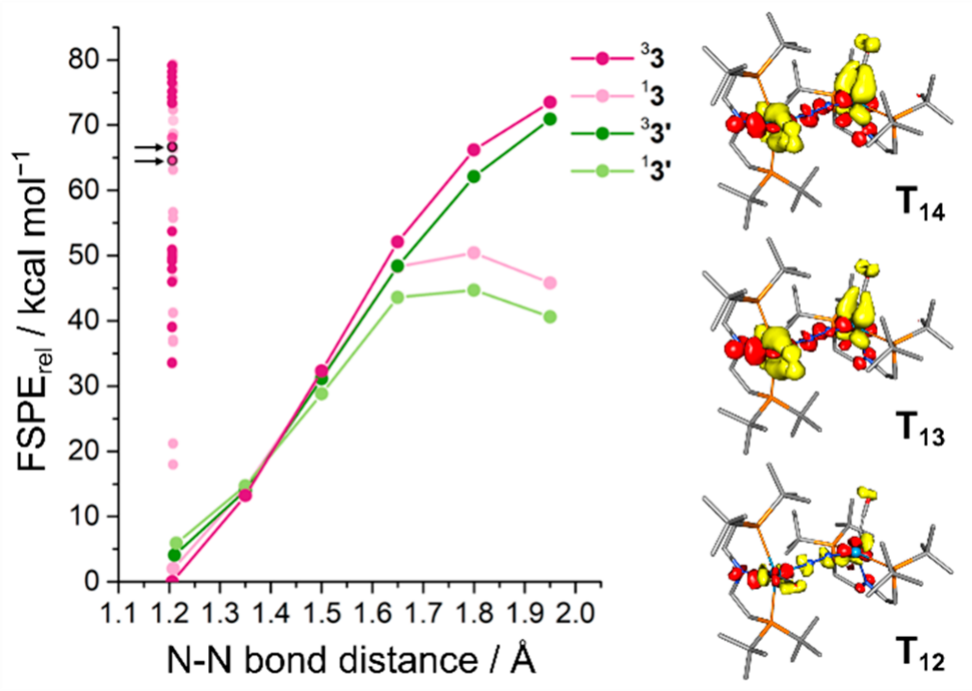
(*left*) Relaxed surface scan for **3** and **3**’ in their singlet and triplet states, respectively. Excited states in the ground state geometry of **3** as predicted with TD-DFT are shown as smaller circles and the states T_12_ and T_13_/T_14_ in the Franck–Condon region are marked with black arrows. (*right*) Difference densities (yellow density loss, red density gain, contour value 0.003) of the excited states T_12_, T_13_, and T_14_.

More details with respect to the relevant spin-state energetics were obtained from a relaxed surface scan along the N–N bond, considering the two pincer conformations that start from **3** and **3′** in their singlet and triplet electronic configurations (Figure 5). At no point along the scan, a clear open-shell singlet (OSS) state could be identified; the Mulliken spin populations on the tungsten ions remain below ±0.25 for a putative OSS in any of the available geometries. At N–N distances between 1.35–1.50 Å, the singlet and triplet states are essentially degenerate, while above 1.65 Å the singlet states of each conformer are energetically favored with ^1^**3′** forming the lowest-lying surface. In comparison, for N_2_ bridged Mo triamide platforms, the singlet and triplet state surfaces were computed to cross at larger separations (ca. 1.5–1.6 Å).^67,63^

In comparison with our structurally and electronically related {π^10^δ^4^} complex [(N_2_){ReCl(PNP)}_2_] (**D**; Δ*H*°_DFT_ = 26.0 kcal·mol^–1^; Δ*H*^‡^_exp_ = +24 ± 1 kcal·mol^–1^), thermal cleavage of **3** exhibits less favorable thermochemistry and kinetics.^30^ The simplified electronic structure considerations for N–N splitting discussed above (Figure 2) imply a reorganization of the ^3^{WNNW} core that leads to transfer of two electrons from the ground-state π*−π–π* MO to the σ–σ*−σ originating MO and crossing onto the dissociative ^1^{W≡N + N≡W} surface. The thermochemistry and kinetics should therefore correlate with the relative energies of these MOs along the reaction coordinate. From this picture, some qualitative predictions can be derived upon replacing a weak π-donor ligand (**D**) for the strong π-acceptor CO, which mixes with the π-MO manifold. Depletion of electron density from the metal by backdonation to CO should thermodynamically disfavor N–N splitting, which is reductive in nature, as evidenced by the CO stretching vibrations of **3** and **4** (see above). Furthermore, stabilization of both π*−π–π* MOs in the *C*_2_ symmetric dicarbonyl dimer is expected to raise the overall barrier for N–N scission. We therefore attribute the less favorable thermochemistry and higher kinetic barriers for N–N cleavage of **3** vs **D** at least in part to the presence of the CO ligands.

### Photodissociation of N_2_

**Figure 6:**
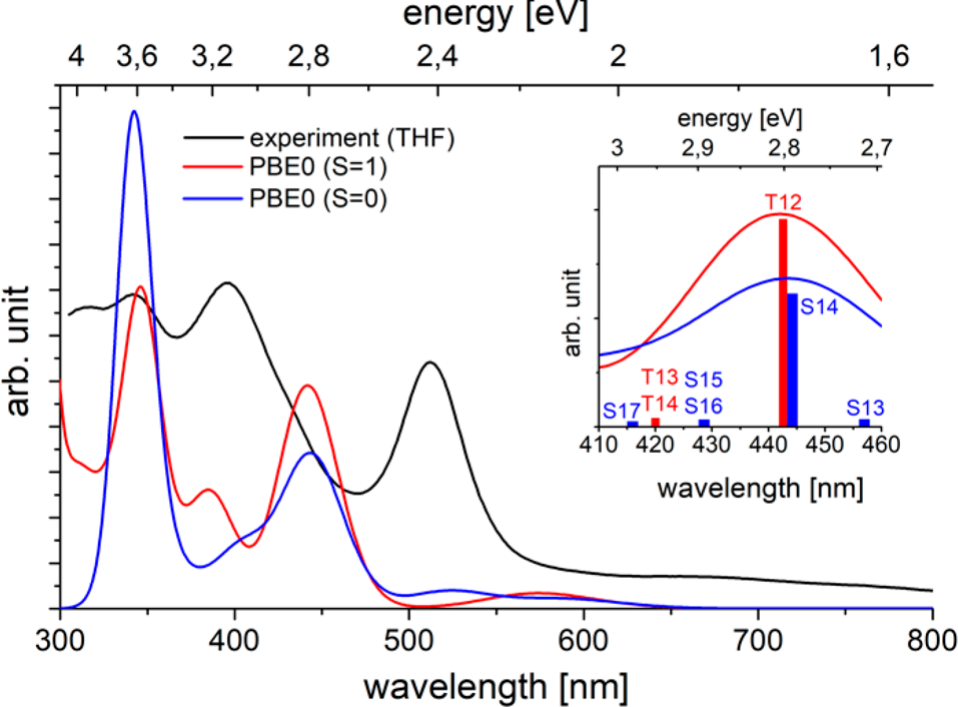
Experimental (black) and TD-DFT-computed (blue *S* = 0; red *S* = 1; see the SI for details) electronic absorption spectra. (*inset*) Computed productive region for N_2_ cleavage.

The photodriven splitting of related Re pincer platforms was recently reported by the groups of Schneider (**B**, Figure 1) and Miller (**C**) and was therefore also examined for **3**.^20,22^ While thermal dissociation at room temperature is both thermochemically and kinetically unfavorable, quantitative N–N splitting is obtained within 8 h upon photolysis in benzene at λ = 427 nm (LED, Δλ = 10 nm). As for complex **B**, a low quantum yield below 1% (Φ_427 nm_ = 0.37 ± 0.03%) was obtained. The quantum yield shows no significant temperature dependence over a wide range (−80 to 25 °C), suggesting that conformational equilibria of the ground state have no effect on the photochemical process. Broadband irradiation with a Xe-arc lamp (λ = 395–590 nm) around the strong absorption band at 511 nm (Figure 6) gave similar results. Photolysis with wavelengths >540 nm resulted in significantly reduced photocleavage rates and no conversion was obtained above λ > 590 nm. On the other hand, shorter wavelengths (λ < 395 nm) gave substantial amounts of undefined side products. Competing N–N vs M–N_2_ photodissociation was observed for some other N_2_ bridged group six complexes.^15,16,22^ However, photolysis of **3** at λ > 305 nm under ^15^N_2_ does not lead to ^15^N incorporation into the nitride photoproduct, suggesting that the W–N_2_ bond is photostable under these conditions. Photolysis of **4** at λ > 305 nm also showed decomposition of the nitride into undefined products. The photodegradation at low wavelengths might be attributed to CO dissociation from **3** and/or **4**.

The density and nature of accessible electronic states upon photoexcitation were examined computationally with TD-DFT. The computed electronic absorption spectra for ^3^**3** and ^1^**3** are blue-shifted by ca. 0.38 eV with respect to the experimental spectrum (Figure 6). ^3^**3** shows best agreement with the experimental intensity distribution and relative energies (see the SI). However, ^1^**3** exhibits excitations of almost identical character in the spectral region that is relevant for the photoreactivity (see Figure 6 and the SI). The intense band in the visible range (*E*_exp_ = 2.4 eV (511 nm)) overlaps with the low energy edge of the photochemically productive region (∼550 nm). It is assigned to transition T_12_ (^3^**3***E*_calc_ = 2.8 eV (443 nm)) as an excitation within the {WNNW} π manifold that shifts electron density from the N_2_-bridge to the metal ions (Figure 5). At slightly higher energy, two transitions of low intensity (T_13_, T_14_; *E*_calc_ = 2.89 eV) involve excitations from the δ-type orbitals with significant CO contributions into π*−π*−π* MOs that are delocalized over the {WNNW} core. Both types of states therefore mainly exhibit charge transfer character within the core, either predominantly N_2_-to-W (T_12_) or W-to-N_2_ (T_13_/T_14_), respectively. Additional CT character to the pincer nitrogen atom is more pronounced in T_13_/T_14_ than in T_12_.

A high density of states around and below the photochemically relevant excited states T_12_ and T_13_/T_14_ was found. In these states, orbitals of predominant π*−π–π* and π*−π*−π* character are partially occupied (see the SI). The system may therefore evolve in the FC region to SOC-coupled singlet and triplet states with excitation character of initially δ to π*−π*−π* or δ to π*−π–π* type. However, derivation of energy gradients by TD-DFT excited-state relaxation was not successful. Furthermore, TD-DFT cannot describe homolytic bond cleavage at large displacement from the equilibrium geometry beyond the Coulson–Fischer point^64−69^ and does not capture double excitations, which are expected to become increasingly relevant closer to the dissociation limit. Note that within the simple MO considerations (Figure 2), the ^1^**4** product surface can be considered a doubly excited state of ^1^**3**. Theoretical description of the excited state dynamics therefore requires more refined treatment, which is impeded by the currently available computational methodologies and resources for a complex as large as **3**.

### Spectroscopic Examination of N_2_ Photodissociation

**Figure 7:**
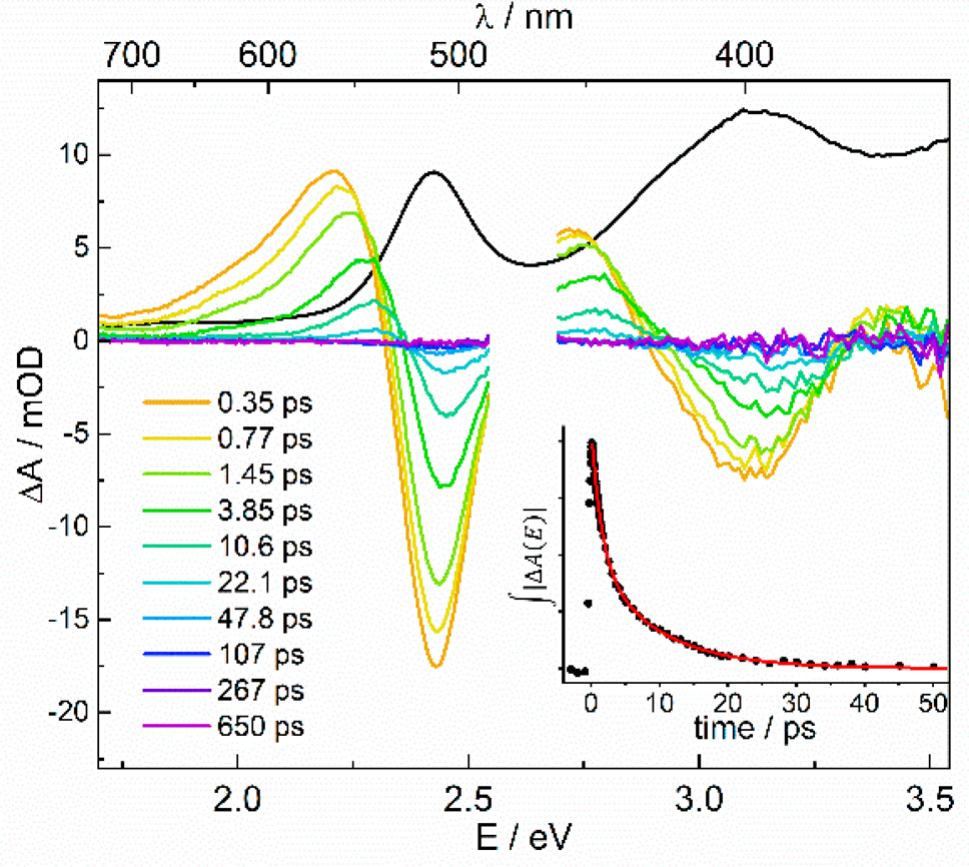
Transient UV/vis difference spectra of **3** in THF at selected pump–probe delays (pump wavelength 475 nm). The black line shows the scaled linear absorption spectrum. (*inset*) Time-dependence of the integrated absolute absorption changes (the red line is a biexponential fit).

The photochemistry of **3** was examined by ultrafast UVvis/UVvis and UVvis/IR transient absorption spectroscopies in THF. Different pump wavelengths in the productive range (400, 440, 475, 511, 530 nm) were applied, all giving similar observations (Figure 7 and the SI). Directly after excitation, the transient difference spectra show bleaching in the centers and enhanced absorption at the low energy sides of the ground state absorption spectrum. This is a clear signature of a vibrationally hot electronic ground state molecule being formed within the temporal resolution of the experiment (τ_exc_ ≈ 70 ± 20 fs), as no features of an electronically excited state were found. Experiments using 330 and 380 nm pump wavelengths confirmed slow decomposition into undefined products, corroborating the results from steady state photolysis.

Thermal cooling results in almost full relaxation at times >60 ps, which is consistent with the low quantum yield for N–N bond cleavage. The relaxation dynamics of the ground state were quantified by analyzing the integral over the absolute value of the UVvis/UVvis difference spectra |Δ*A*(*E*)| over the whole measured spectral range (inset in Figure 7). Its time dependence was fitted by a biexponential decay giving time constants (relative amplitudes) of τ_1_ = 1.5 ± 0.2 ps (54%) and τ_2_ = 9.2 ± 0.5 ps (46%), respectively. The 9.2 ps component is a typical value for the vibrational energy transfer time of a highly excited molecule in a solvent. The fast component hints at a nonstatistical energy distribution created by preferential population of those vibrational modes, which couple to the electronic transition. The time scale of τ_1_ = 1.5 ps is consistent with intramolecular vibrational redistribution (IVR) to establish a quasi-equilibrium of the internal energy.^70−75^ This assignment is supported by the observation that the amplitudes of the hot bands depend on the pump wavelength (see the SI). UV-pump (400 nm) mid-IR-probe spectroscopy using the strong CO stretching modes as spectroscopic probes also indicate fast internal conversion (IC) followed by cooling dynamics in the ground state (see the SI). Here, recovery of the ground state bleach of the CO absorption band occurs with a characteristic time of 16 ± 3 ps.

The transient spectroscopy data is in agreement with two conceivable pathways for the photoreactivity of **3**, i.e., (a) nonradiative electronic electron/hole recombination within temporal resolution followed by N–N dissociation of the vibrationally hot ground state or (b) ultrafast internal conversion from the Franck–Condon (FC) region onto the dissociative singlet surface. The experimental derivation of the ground-state kinetic barrier (Δ*H*^‡^_exp_ = 30.1 ± 0.9 kcal·mol^–1^; Δ*S*^‡^_exp_ = +2.3 ± 0.4 cal·mol^–1^·K^–1^) allowed for estimating whether the photon energy is sufficient for a vibrationally hot and internally equilibrated ground state to dissociate within the time scale of thermal cooling in the solvent bath. Using the ground-state frequency computations obtained from DFT, an upper limit for the internal temperature was estimated (*T*_exc_ ≈ 500 K) that arises from excitation with a 400 nm photon, followed by ultrafast internal conversion (IC) to the ground-state and IVR mediated vibrational equilibration (see the SI). Importantly, at that temperature the unimolecular rate for N–N dissociation (*k*_500 K_ = 2.3 s^–1^) cannot compete with the rapid cooling rate (τ_2_ ≈ 9.2 ps).

**Figure 8:**
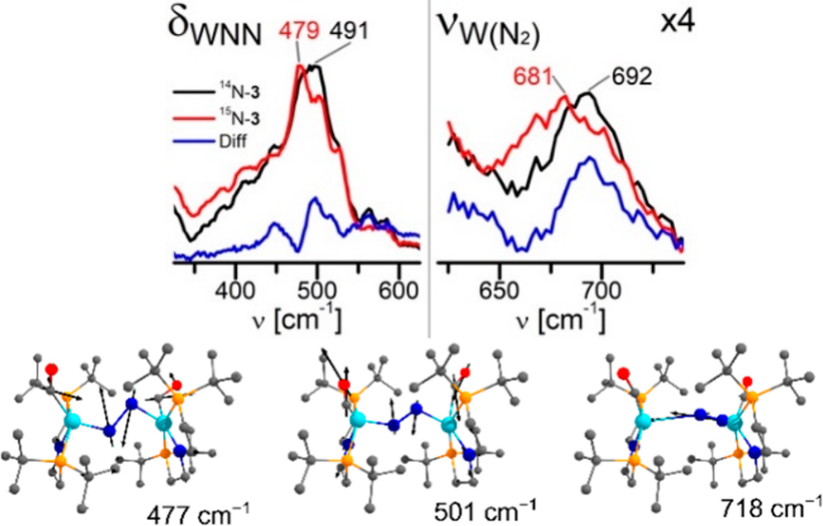
(*top*) Expansions of the rR spectra (λ_exc_ = 514.5 nm; −50 °C) of **3** (black) and ^15^N_2_-**3** (red) and difference spectrum for the two isotopologues (blue) with band assignments (the right spectrum is scaled by a factor of 4). (*bottom*) Computed bending modes of the {WNNW} core of **3**.

In consequence, photoreactivity from a hot ground state requires nonstatistical vibrational energy distribution, which rapidly decays with the time scale of IVR (τ_1_ = 1.5 ps). Thus, productive vibrational modes might be activated directly upon excitation. We therefore turned to resonance Raman (rR) spectroscopy, which exhibits a signal enhancement, if a dipole allowed electronic transition is coupled to a vibrational mode that is totally symmetric for the ground and excited state geometries and aligns with the displacement of the potential energy surface upon excitation.^76^ The low symmetry of **3** in the ground state (*C*_2_) should be beneficial to allocate the fundamental modes of the {WNNW} core that are coupled to the strong absorption band at 511 nm, which marks the low energy edge of the photochemically productive spectral window. rR spectra (λ_exc_ = 514.5 nm) of **3** and the isotopologue ^15^N_2_-**3** showed distinct differences for three bands: Besides the N–N stretching mode (ν_NN_), the maximum of a broad feature at 491 cm^–1^ is red-shifted by around −12 cm^–1^ for ^15^N_2_-**3** (Figure 8). Furthermore, the weak band at 692 cm^–1^ exhibits an isotope shift of −11 cm^–1^. These features lie within the range for deformation (δ_MNN_) and stretching (ν_MN_) modes of terminal and linearly bridged N_2_ complexes.^77−81^ The assignment of the isotope sensitive rR bands are supported by DFT computations. Below 500 cm^–1^, two modes that represent a *zigzag*-type distortion of the {WNNW} core were found (δ_WNN_^DFT^ = 475 cm^–1^, 477 cm^–1^; Figure 8) with isotopic shifts of Δδ_WNN_^DFT^ = −8 and −6 cm^–1^, respectively. The weaker band is assigned to a W–N_2_ stretching mode (ν_MN_^DFT^ = 718 cm^–1^; Δν_MN_^DFT^ = −19 cm^–1^). The rR data therefore support the coupling of bending modes of the {WNNW} core, which reflect the ground-state reaction coordinate, with excitation(s) in the photochemically productive region.

### Nitride Carbonylation and Transfer

**Figure 9:**
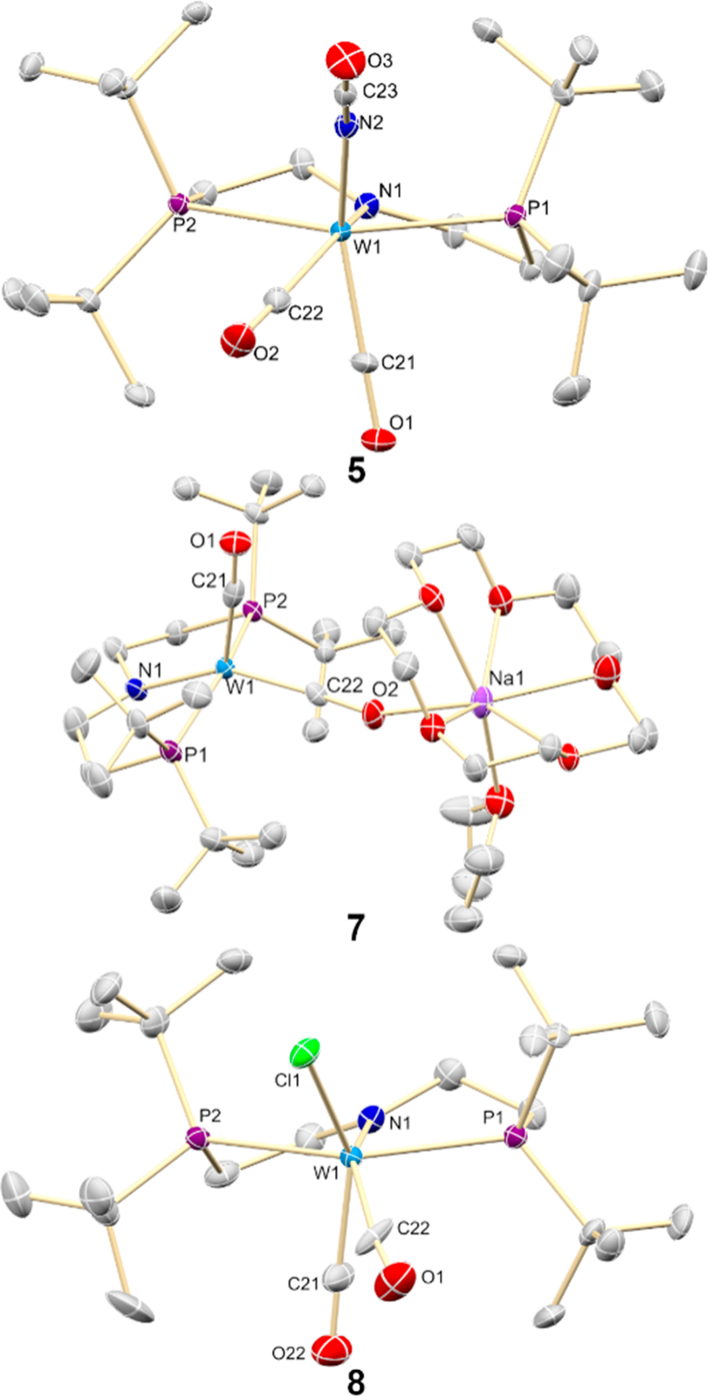
Molecular structures of **5**, **7**, and **8** in the crystal from X-ray diffraction. Hydrogen atoms were omitted for clarity. Selected bond lengths [Å] and angles [°] for **5** [W1–N1 2.011(3), W1–C21 1.964(4), W1–C22 2.028(4), W1–P1 2.5077(10), W1–P2 2.5030(10), W–N2 2.116(3); C21–W1–N1 153.31(14), C22–W1–N1 85.54(15), C21–W1–C22 77.03(16), P1–W1–P2 155.09(3), C21–W1–N2 146.74(15)], **7** [W1–N1 2.088(6), W1–C21 1.902(9), W1–C22 1.911(8), W1–P1 2.426(2), W1–P2 2.4484(19); C21–W1–N1 148.0(3), C22–W1–N1 124.3(3), C21–W1–C22 87.7(3), P1–W1–P2 156.74(6)], and **8** [W1–N1 2.013(6), W1–C21 1.939(8), W1–C22 2.056(8), W1–P1 2.5175(19), W1–P2 2.516(2), W1–Cl1 2.4682(19); C21–W1–N1 152.9(3), C22–W1–N1 89.2(3), C21–W1–C22 73.0(3), P1–W1–P2 155.24(6), C21–W1–Cl1 143.0(3)].

**Scheme 3:**
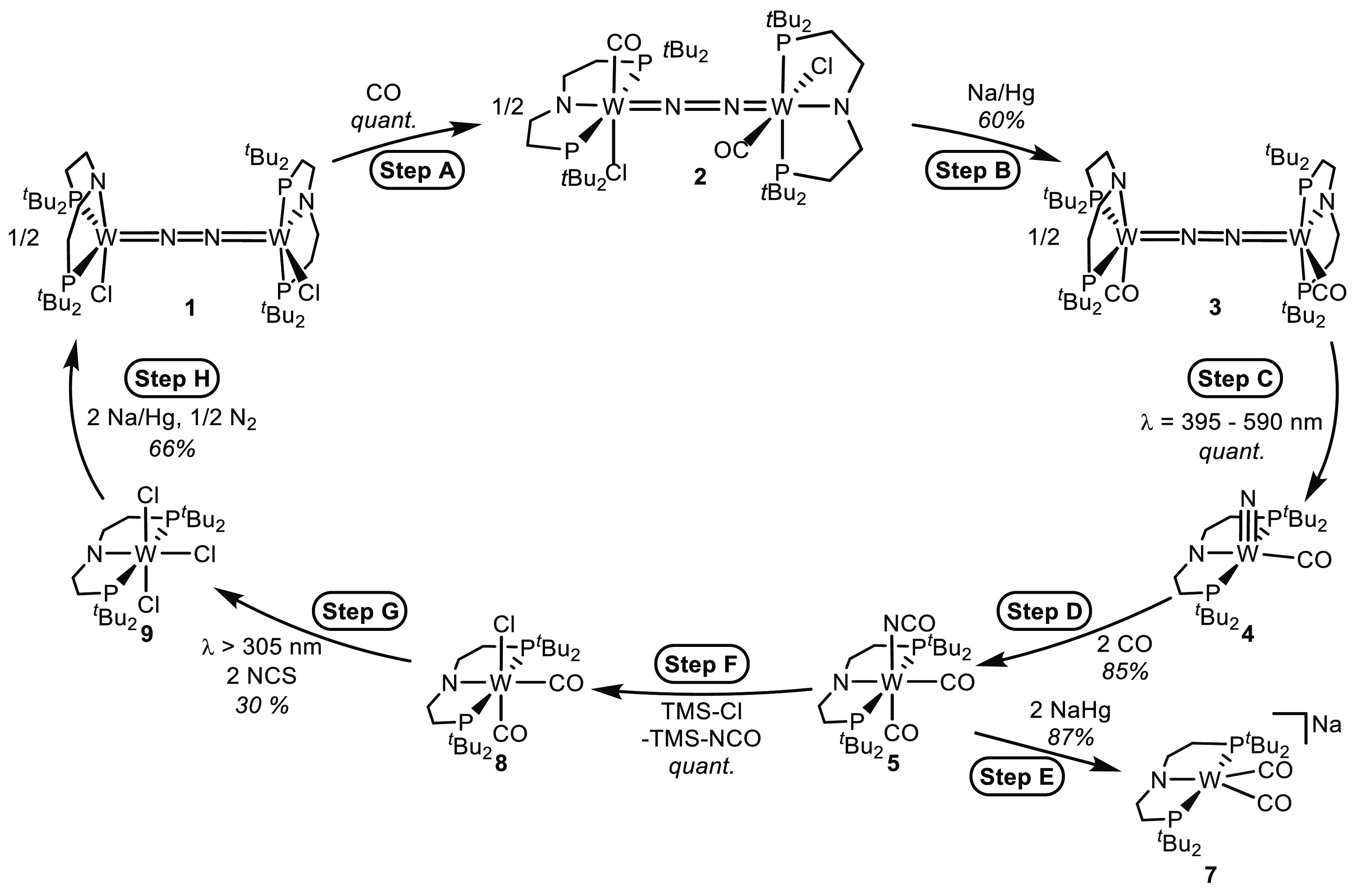
Synthetic Cycle for Photodriven Formation of Me_3_SiNCO from N_2_ and CO NCS = *N*-chlorosuccinimide.

Terminal nitride complexes that were reported from thermal N_2_ splitting are generally weak nucleophiles often requiring strong electrophiles for functionalization. The endothermic nature of the N_2_ splitting reaction might lead to more activated nitrides and facilitate nitrogen transfer reactivity. This was evaluated by isocyanate formation. Besides C–N coupling of N_2_ complexes with CO,^9^ only one example for initial N_2_ splitting and subsequent nitride carbonylation is currently known.^82^ Reaction of **4** with CO (1 atm) gives deep purple [W(NCO)(CO)_2_(PNP)] (**5**) in yields up to 85% (Scheme 3, Step D). In the IR spectrum of **5**, the intense band at ν_NCO_ = 2203 cm^–1^ (Δν_15N_ = 6 cm^–1^) and two CO stretching modes (ν_CO_ = 1909, 1832 cm^–1^) evidence the formation of the dicarbonyl isocyanate complex. The ^15^NCO isotopologue was obtained from isotopically labeled ^15^N-**4**, confirming N_2_ as nitrogen source. The ^15^N NMR signal of ^15^NCO-**5** (δ_N_ = −347 ppm; ^2^*J*_NP_ = 2.6 Hz) is flanked by tungsten satellites, corroborating *N*-coordination of the cyanate-ligand. The *cis*-dicarbonyl configuration of **5** was further confirmed by X-ray crystallography (Figure 9, *top*).

**Figure 10:**
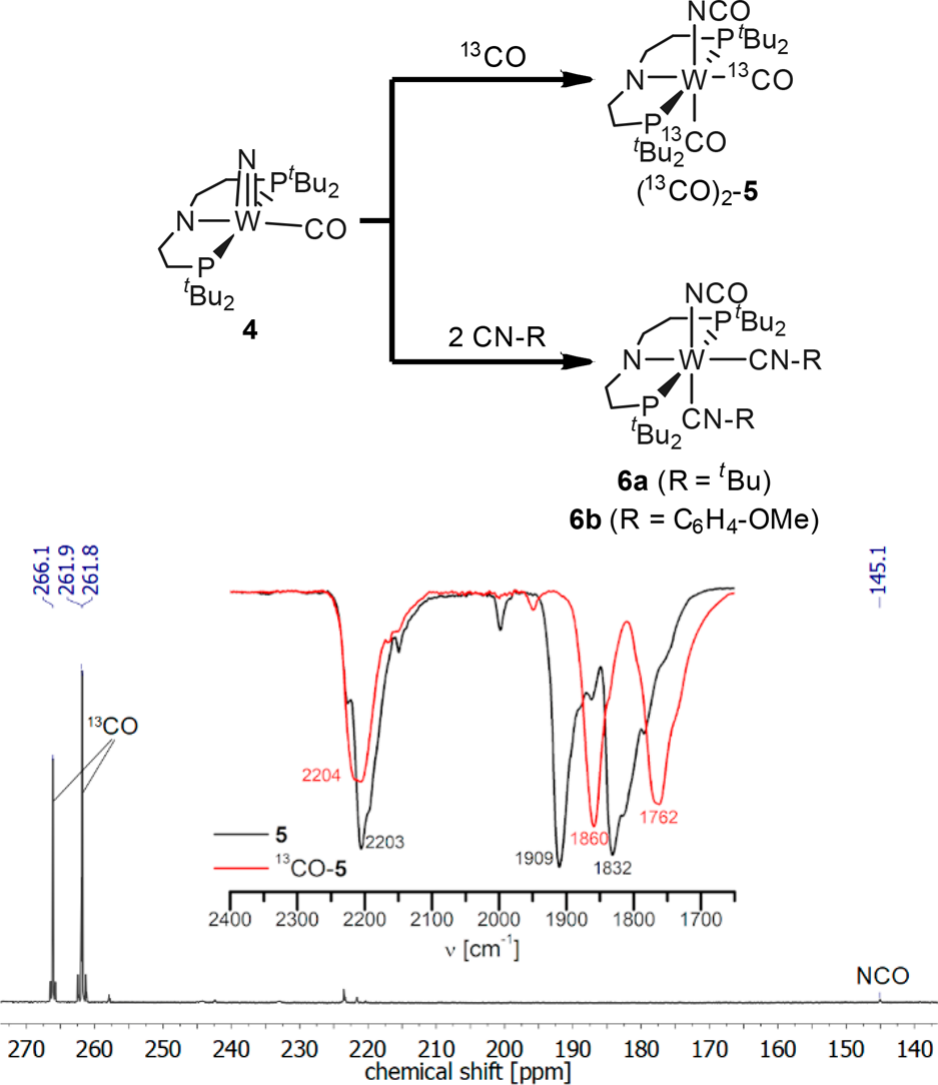
(*top*) Reactions of nitride complex **4** with ^13^CO and isocyanides. (*bottom*) ^13^C{^1^H} NMR spectrum of (^13^CO)_2_-**5** and IR spectra of **5** (black) and (^13^CO)_2_-**5** (red).

Two pathways for isocyanate formation are conceivable, i.e., (a) direct, outer-sphere attack of CO at the nitrogen atom, in reversion of the related N_2_ elimination reaction from coordinated azide or (b) a stepwise mechanism with initial coordination of CO to the metal and subsequent transfer to the nitride ligand. Inter- vs intramolecular C–N bond formation was distinguished by a ^13^CO labeling experiment. Reaction of **4** with ^13^CO selectively yields [W(NCO)(^13^CO)_2_(PNP)] ((^13^CO)_2_-**5**), as evidenced by IR and ^13^C NMR spectroscopy (Figure 10). Analogous results were obtained upon reaction of **4** with isocyanides (CNR, R = ^*t*^Bu, C_6_H_4_–OMe; Figure 10) with no indication for carbodiimide isomers. Both the labeling experiment and the reaction with isocyanide therefore confirm intramolecular attack at the nitride ligand as the favored pathway for heterocumulene formation.

Cyanate release was examined on two different routes. Reduction of **5** with Na/Hg (2 equiv) yields bright orange Na[W(CO)_2_(PNP)] (**7**; Scheme 3, Step E) in isolated yields up to 87%. The tungstate(0) product **7** exhibits square pyramidal (τ_5_ = 0.15)^44^ coordination in the solid state with an apical CO ligand (Figure 9, *middle*). A single ^13^C NMR CO signal (δ_C_ = 240 ppm) and one ^*t*^Bu ^1^H NMR resonance (δ_H_ = 1.32 ppm) indicate averaged *C*_2*v*_ symmetry on the NMR time scale. Strong backbonding is evidenced by low CO stretching frequencies (ν_CO_ = 1677, 1604 cm^–1^). Alternatively, cyanate release is enabled by salt-metathesis with Me_3_SiCl. [WCl(CO)_2_(PNP)] (**8**) and Me_3_SiNCO are obtained in almost quantitative spectroscopic yields, respectively (Scheme 3, Step F). The chloro complex **8** features similar spectroscopic and structural properties as parent **5** (Figure 9, *bottom*). Me_3_SiNCO can be easily separated from the reaction mixture by *trap-to-trap* transfer of the solvent and was identified spectroscopically by comparison with an authentic sample. Silylisocyanate generation from N_2_ was finally confirmed by ^15^N labeling. The full synthetic cycle for the conversion of N_2_ into trimethylsilylisocyanate could finally be closed by oxidation of **8** with *N*-chlorosuccinimide (NCS, 2 equiv.) under photolytic conditions (λ > 305 nm). The tungsten(IV) trichloride [WCl_3_(PNP)] (**9**) was obtained in yields up to 30 % (Scheme 3, Step G). Irradiation is required to obtain complete decarbonylation. Complex **9** is the direct precursor to the N_2_ complex **1** (Scheme 3, Step H).^31^

## Discussion

**Figure 11:**
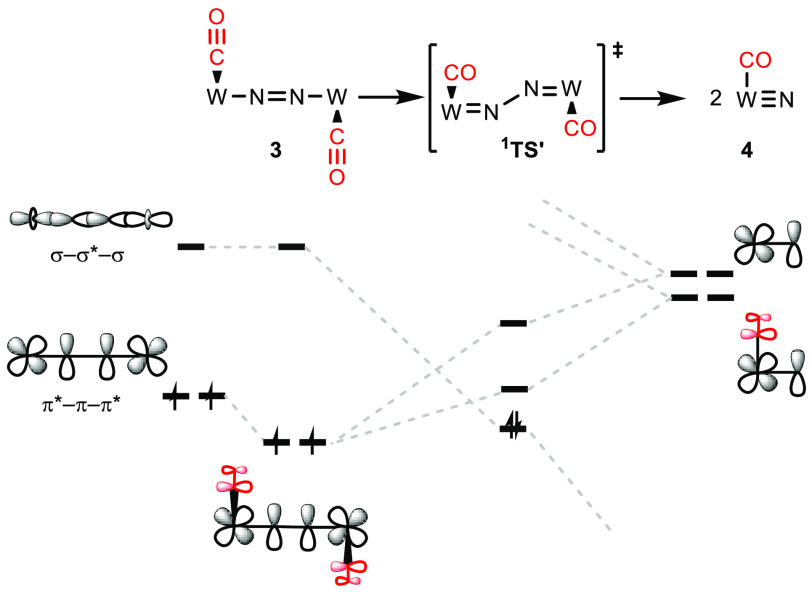
Qualitative MO correlation diagram with relevant interactions for the splitting of **3** into **4**.

The thermal dissociation of linearly N_2_ bridged ditungsten complex **3** into terminal nitride complex **4** is a unique example of fully reversible N_2_ cleavage. The reaction is endothermic and entropically driven at elevated temperatures. As for Cummins’ complex **A** (Figure 1), a similarly small entropy of activation was found. Computational analysis confirmed an analogous *zigzag* distortion of the {π^10^δ^4^} ^3^{WNNW} core when approaching the transition state, which is located on the singlet surface. This displacement lifts the quasi-degenerate MOs of the {WNNW} π-manifold and stabilizes the vacant σ–σ*−σ MO. Reduction of the symmetry by bending leads to mixing of σ/π MOs, which lowers the energy for intersystem crossing of the ^3^A starting and ^1^A product states and ultimately the kinetic barrier for N_2_ dissociation. Our DFT results reflect the analysis for oxygen atom transfer from R_3_P=O to M(OSiR)_3_ (M = V, Nb, Ta) by Wolczanski and Cundari as well as Cummins’ qualitative bonding model for N_2_ splitting (Figure 2).^2,83^ Along these lines, we associate the endothermic nature of N–N scission with the presence of the strongly π-accepting carbonyl ligands, which compete with the N_2_ bridge for back-bonding from the metal ions. These considerations similarly apply to the kinetic barrier, which should be increased by π-accepting ligands that stabilize the π*−π–π* donor level of the ground state (Figure 11). In fact, **3** is the first carbonyl dinitrogen complex that was reported to undergo N_2_ splitting.

The presence of the CO ligands allows for estimating the degree of the net tungsten to nitrogen electron transfer that is associated with N–N cleavage. This is by no means obvious. Significant covalent contributions to metal bonding with the nitride ligand are expected,^84−86^ as was shown for various terminal nitride complexes, e.g. by electronic and EPR spectroscopy and computational bond analysis.^28,55,87,88^ Bendix et al. therefore proposed the use of the Enemark–Feltham notation for nitride complexes to avoid ambiguities from formal oxidation states which lose their physical meaning with increasing covalency.^39,40^ Similarly, Holland pointed out for N_2_ complexes that the broad range of N–N stretching vibrations exhibits a decent agreement with Badger’s rule, indicating a continuum of electron transfer that arises from covalent contributions to M–N_2_ bonding and backbonding.^3,4,89^ In the current case, N–N cleavage results in a distinct blue-shift of the CO stretching vibration by more than 100 cm^–1^. For comparison, the 1-electron oxidation of monocarbonyl complex *trans*-[ReCl(CO)(Ph_2_PCH_2_CH_2_PPh_2_)_2_] is associated with a smaller blueshift of 74 cm^–1^,^90^ suggesting that the electronic and structural reorganization associated with N–N cleavage is accompanied by considerable net M-to-N electron transfer.

**Figure 12:**
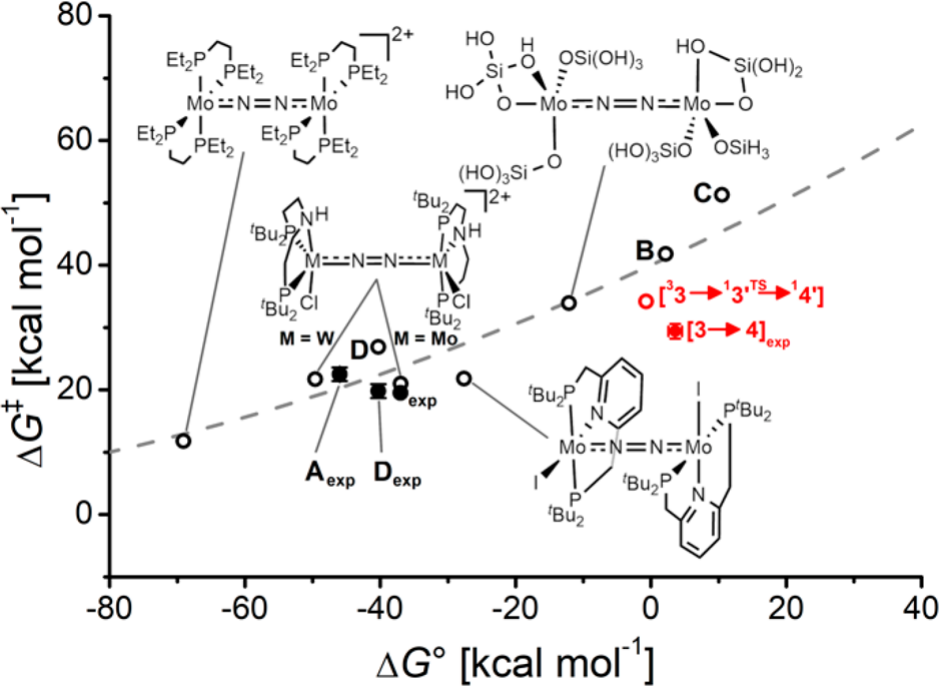
Correlation of reaction free energies and free energies of activation for the splitting of μ^2^-η^1^:η^1^-N_2_ bridged complexes into terminal nitrides (open circles Δ*G*^‡^DFT(Δ*G*°_DFT_); closed circles Δ*G*^‡^exp(Δ*G*°_DFT_) and Δ*G*^‡^_exp_(Δ*G*°_exp_) for **3**). The dashed line denotes a Marcus model (Δ*G*^‡^ = (λ + Δ*G*°)2/4λ) for λ = 160 kcal·mol^–1^ [Adapted with permission from ref (4). Copyright 2021 American Chemical Society].

A limited number of mechanistic studies reported computed thermochemical and kinetic parameters for the splitting of μ^2^-η^1^:η^1^-N_2_ bridged complexes into terminal nitrides.^2,7,20,21,29−31,35,36,91^ In some cases, experimental kinetic data was obtained and generally showed good agreement of the kinetic barrier (Δ*G*^‡^) within about 5 kcal·mol^–1^. As all of these systems were computed to proceed through the distinct *zigzag* transition state, a scaling relationship for the reaction free energies and free energies of activation should arise, if the electronic rearrangement within the {MNNM} core determines the thermochemistry and kinetics of N–N splitting. In fact, the computational data for the reported 4*d*/5*d* platforms that cover a variety of metals, ligands, redox and spin states, and coordination geometries exhibit a surprisingly good agreement with a simple Marcus-type quadratic free energy relationship (Δ*G*^‡^ = (λ + Δ*G*°)^2^/4λ) using the reorganization energy λ as a single parameter (Figure 12).^4,92^ The correlation supports that the reaction energetics are dominated by the electronic reorganization through the common, *zigzag* transition state (Figure 2), while other factors like sterics are less relevant. The current study allows for the first time experimental benchmarking of both kinetic and thermochemical computational parameters with satisfying results. The computed value fits well with the previous data for λ = 160 kcal·mol^–1^ (Figure 12). This high reorganization energy suggests that accessible kinetic barriers require driving forces around or below Δ*G*° = −20 kcal·mol^–1^ for thermal dissociation of μ^2^-η^1^:η^1^-N_2_ bridged complexes. In consequence, the resulting terminal nitride complexes are easily overstabilized hampering subsequent functionalization or even catalytic turnover. Photochemically driven N–N scission is therefore an interesting strategy to break this unfavorable scaling relation and even benefit from kinetically inaccessible barriers for the reverse process, i.e. bimolecular nitride coupling.

Photolysis of **3** in the range λ = 395–590 nm quantitatively produces nitride **4** at room temperature. UVvis/UVvis and UVvis/IR pump–probe spectroscopy revealed ultrafast excited state decay within the temporal resolution of the optical probe experiment (τ_exc_ ≈ 70 ± 20 fs). Rapid nonradiative excited state decay is in line with the high computed density of states in the FC region and below according to the “energy gap law”.^93,94^ In comparison, the photostable complex [(μ^2^-η^1^:η^1^-N_2_){Mo(PPh_2_Me)_2_(^Ph^Tpy)}_2_]^2+^ (^Ph^Tpy = 4′-Ph-2,2′,6′,2″-terpyridine) exhibits much slower recovery of the electronic ground state within 15–25 ps.^23^ This π^10^-complex displays a similar degree of N_2_ activation (ν_NN_ = 1563 cm^–1^) as **3** (ν_NN_ = 1598 cm^–1^), yet with a ^1^A_g_ ground state that originates from splitting of the π*−π–π* MOs in *D*_2*h*_ symmetry.^95^ In contrast to **3**, excitations in the vis/NIR region were assigned to ^1^MLCT transitions to the Tpy auxiliary ligands. Relaxation kinetics were attributed to rapid intersystem crossing (ISC) and decay via a cascade of ^3^MLCT states as main channel, which shifts electron density back to the {MoNNMo} core. Notably, TD-DFT modeling of the ^1^MLCT excited state indicate preserved linearity of the core. In consequence, the ν_NN_ stretching mode, which exhibits a Fermi resonance with the Tpy chromophore, is the only rR active mode that is ^14/15^N_2_ sensitive.^24^ It is tempting to associate the lack of N–N photodissociation of this complex with a lack of bending modes that are activated by electronic excitation and prepare a vibrationally excited core that passes through the *zigzag* TS in the electronic ground state.

This notion is supported by results from Blank and Cummins for the photochemically active complex **A** (Figure 1).^22^ Dissociation proceeds upon excitation (540 nm) to a triplet state with (π–π*−π)^3^(π*−π–π*)^3^ character, which reflects the nature of the intense transition T_12_ of **3** (Figure 6). Based on simple orbital considerations, this is remarkable, as this transition should weaken M–N and strengthen N–N bonding relative to the triplet (π–π*−π)^4^(π*−π–π*)^2^ ground state. However, as in the case of **3**, subps electron–hole recombination was observed and N–N dissociation was attributed to vibrationally excited ground-state reactivity. An oscillation in the pump–probe decay (70 cm^–1^) was associated with an activated low energy {MoNNMo} bending mode. Further support for nonstatistical vibrational energy distribution came from different N–N over Mo–N dissociation yields for the photochemical and thermal routes, respectively.

**Figure 13:**
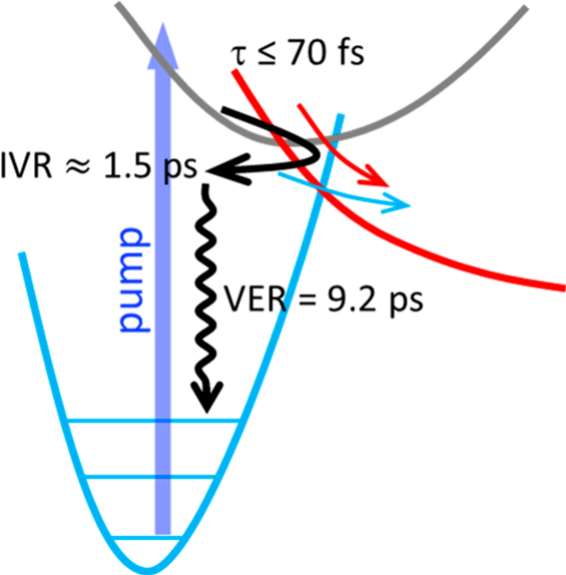
Schematic sketch of the conceivable paths for photodissociation of **3** via a vibrationally excited ground state (black/blue path) or excited state crossing onto the dissociative product surface (red path).

Our comparison of the cooling kinetics of **3** with thermal dissociation rates emphasizes that a vibrationally equilibrated ground state cannot dissociate within the time scale of cooling in the solvent bath. Nonstatistical vibrational energy distribution would therefore be a prerequisite for hot ground state reactivity, reflecting Blank’s and Cummins’ results. It is plausible that the accessible CT states within the core, such as T_12_, experience some degree of distortion with respect to the near linear ground-state geometry. McNaughton et al. presented a detailed analysis of vibronic coupling in [Mo^III^(N_2_)(N_3_N)].^96^ Its ^2^E ground state configuration (i.e., (π–π*)^3^ applying the notation used in Figure 2 to this mononuclear complex with an end-on N_2_ ligand) is pseudo-Jahn–Teller coupled to bending modes that are perpendicular to the Mo–N–N axis. The rR data of complex **3** indicate that stretching and bending modes of the {WNNW} core are activated upon excitation in the productive optical region. To this end, our results are in line with a scenario, in which vibronically coupled modes that align with the *zigzag* reaction coordinate facilitate quasi-thermal N–N photodissociation on the ground state surface (Figure 13, blue path). Besides **A**, such a path was also observed for N_2_ photoelimination from a ferric azide complex.^97^ Computational examinations of thermal N_2_ elimination from azide complexes feature related [M=N···N=N] *zigzag* transition states.^55,98^ Notably, dissociative chemisorption of N_2_ on heterogeneous ruthenium catalysts was recently shown to be facilitated by plasma-induced vibrational excitation of N_2_.^99,100^

We need to emphasize that the ultrafast electronic relaxation of **3** and low quantum yield did not allow for excluding a path that leads from the FC region to the photoproduct without repopulation of the ^3^A ground state (Figure 13, red path). We note in passing that the excited states T_13_/T_14_ resemble our assignments for the productive excitations of photoactive complex **B** (Figure 1), while the analogous transition of T_12_ was in that case outside the suitable energy window.^20^ However, our simple Marcus analysis (Figure 12) suggests that in the ground state geometry of **3** the dissociative ^1^A potential energy surface should be at high energy (λ = 160 kcal·mol^–1^), implying large structural reorganization to enable surface crossing. Detailed theoretical analysis of the excited state dynamics with consideration of vibronic interactions and competing SOC requires the use of multideterminantal methods, which is currently impeded by the large size of the system.

The enhanced reactivity of the photolytically produced nitride was demonstrated by near quantitative nitride carbonylation at ambient conditions. Isocyanate formation from nitride complexes and CO was previously reported in several instances,^101−109^ yet only for one example coupled to N_2_ splitting.^88^ In that case, selective carbonylation of the N_2_ splitting product required oxidation from V^IV^/V^IV^ to V^V^/V^V^. In a complementary approach, Sita and co-workers reported the coupling of an N_2_ derived terminal imido ligand with CO, giving free Me_3_ENCO (E = C, Si, Ge).^19^ Given this precedence, nitride carbonylation is mechanistically surprisingly ill-defined regarding direct CO attack at the nitride vs intramolecular coupling of coordinated CO. A computational study by Liddle and co-workers supported CO coordination to azide-derived U^V^ and U^VI^ nitrides, prior to C–N bond formation.^113^ Such an inner-sphere pathway was also proposed for CO oxygenation by terminal oxo complexes and the microscopic reverse oxidative addition of CO_2_.^110−112^^13^CO labeling of nitride **4** experimentally confirmed intramolecular CO attack at the nitride ligand, which is in agreement with Liddle’s findings. It is reasonable to assume that C–N coupling is triggered by initial coordination of CO at the five-coordinate nitride complex. The strong π-acceptor ligand in *trans*-position should enhance the electrophilicity of the nitride ligand and facilitate reaction with ambiphilic CO.

The synthetic cycle for isocyanate formation from N_2_ and CO (Scheme 3) also demonstrates the limitations of this system. The dicarbonyl complex **8** that is obtained after isocyanate release does not directly undergo reductive N_2_ activation but requires full oxidative decarbonylation under photolytic conditions, which comes with moderate yields. Future work will therefore target heterocumulene formation without deactivation by additional CO.

## Experimental Section

### Materials and Synthetic Methods

All experiments were carried out under inert conditions using standard Schlenk and glovebox techniques (argon atmosphere). All solvents were purchased in HPLC quality (Sigma-Aldrich) and dried using an MBRAUN Solvent Purification System. THF, HMDSO, and toluene were additionally dried over an Na/K-alloy. Deuterated solvents were obtained from Eurisotop GmbH and dried over an Na/K-alloy (C_6_D_6_, THF-d_8_, Tol-d_8_), distilled by trap-to-trap transfer *in vacuo* and degassed by three *freeze–pump–thaw* cycles, respectively. Silica gel 60 silanized was purchased from Merck KGaA and heated at 120 °C *in vacuo* for 5 days prior to use. Purification of CO gas (Air Liquide) was obtained by passing the gas through a steel coil cooled to −78 °C. ^13^CO (Eurisotop GmbH, 99.30%) and ^15^N_2_ (Sigma-Aldrich, 98% ^15^N) were used without further purification. *N*-Chlorosuccinimide (NCS) was sublimed prior to use. Me_3_SiNCO and Me_3_SiCl were distilled and degassed. *t*-Butylisocyanide (Sigma-Aldrich) and 4-methoxyphenylisocyanide (Sigma-Aldrich) were used as purchased without further purification, whereas [(N_2_){WCl(PNP)}_2_] (**1**) was synthesized according to published procedures.^31^

### Analytical Methods

NMR spectra were recorded on Bruker Avance III 300 or Avance III 400 spectrometers or an Avance 500 spectrometer with a Prodigy broadband cryoprobe, respectively, and calibrated to the residual solvent signals (C_6_D_6_ δ_H_ = 7.16 ppm, δ_C_ = 128.4 ppm; THF-d_8_ δ_H_ = 3.58 ppm, δ_C_ = 67.6 ppm, Tol-d_8_ δ_H_ = 2.09 ppm, δ_C_ = 20.4 ppm). ^31^P and ^15^N NMR chemical shifts are reported relative to external phosphoric acid and nitromethane (δ = 0.0 ppm), respectively. Signal multiplicities are abbreviated as s (singlet), d (doublet), m (multiplet), br (broad).

Elemental analyses were obtained from the Analytisches Labor, Georg-August-Universität (Göttingen, Germany) using an Elementar Vario EL 3 analyzer. LIFDI-MS (linden cms) spectra were measured by the Zentrale Massenabteilung, Fakultät für Chemie, Georg-August-Universität Göttingen. Resonance Raman spectra for **2** and **3** were recorded using a HORIBA Scientific LabRAM HR 800 spectrometer with open-electrode CCD detector in combination with a free space optical microscope and a He:Ne-laser (632.8 nm). Additionally, Raman spectra for **3** were recorded using a Triple Raman Spectrometer TR 557 from S&I (Spectroscopy & Imaging GmbH). IR spectra were recorded using a Bruker ALPHA FT-IR spectrometer with Platinum ATR module.

Magnetic moments in solution were determined by Evans’ method as modified by Sur and corrected for diamagnetic contribution.^113,114^ Magnetic susceptibility measurements in the solid state were carried out with a Quantum Design MPMS-XL-5 SQUID magnetometer in the temperature range from 295 to 2 K at 0.5 T applied field. The powdered sample was contained in a Teflon bucket and fixed in a nonmagnetic sample holder. Each raw data point for the measured magnetic moment of the sample was corrected for the diamagnetic contribution by subtraction of the experimentally determined magnetic measurement of the Teflon bucket. The molar susceptibility data were corrected for the diamagnetic contribution using the Pascal constants and the increment method according to Haberditzel.^115,116^ Experimental data were modeled with the julX program.^117^ UVvis spectra were recorded on an Agilent Cary 60 equipped with an Unisoku Cryostat (CoolSpek) and magnetic stirrer using quartz cuvettes with an attached tube and a J-Young-cap. All UVvis samples were prepared in a glovebox and transferred out of the glovebox prior to the measurement.

### Synthesis

#### [(N_2_){WCl(CO)(PNP)}_2_] (**2**)

[(N_2_){WCl(PNP)}_2_] (**1**) (100 mg, 84 μmol) is dissolved in benzene (10 mL), degassed via two freeze–pump–thaw cycles and stirred under CO (1 atm) for 20 min. After removal of volatiles *in vacuo*, **2** is obtained as a black-yellow solid in quantitative yield. Longer reaction times lead to loss of N_2_ and formation of [WCl(CO)_2_(PNP)] (**8**). Crystals suitable for X-ray diffraction were obtained by cooling a saturated Et_2_O solution to −40 °C. The synthesis of ^15^N-**2** was carried out starting from [(^15^N_2_){WCl(PNP)}_2_].

^**1**^**H{**^**31**^**P} NMR** (C_6_D_6_, 500 MHz, [ppm]): δ = 3.55 (m, 4 H, NC*H*H), 3.26 (m, 4 H, NCH*H*), 2.42 (m, 4 H, PC*H*H), 1.89 (m, 4 H, PCH*H*), 1.60 (s, 18 H, C*Me*_*3*_), 1.53 (s, 18 H, C*Me*_*3*_), 1.43 (s, 18 H, C*Me*_*3*_), 1.31 (s, 18 H, C*Me*_*3*_). ^**13**^**C{**^**1**^**H} NMR** (C_6_D_6_, 126 MHz, [ppm]): δ = 25.4 (AXY, *N* = |^1^*J*_AX_ + ^3^*J*_AY_| = 16.9 Hz, 2x P*C*H_2_), 25.7 (AXY, *N* = |^1^*J*_AX_ + ^3^*J*_AY_| = 17.1 Hz, 2x P*C*H_2_), 31.0 (m, 2x C*Me*_*3*_), 31.1 (m, 2x C*Me*_*3*_), 31.3 (m, 2x C*Me*_*3*_), 31.4 (m, 2x C*Me*_*3*_), 37.2 (AXY, *N* = |^1^*J*_AX_ + ^3^*J*_AY_| = 16.0 Hz, 2x P*C*Me_3_), 37.8 (AXY, *N* = |^1^*J*_AX_ + ^3^*J*_AY_| = 16.7 Hz, 2x P*C*Me_3_), 38.4 (AXY, *N* = |^1^*J*_AX_ + ^3^*J*_AY_| = 10.5 Hz, 2x P*C*Me_3_), 38.8 (AXY, *N* = |^1^*J*_AX_ + ^3^*J*_AY_| = 11.5 Hz, 2x P*C*Me_3_), 59.2 (AXY, *N* = |^2^*J*_AX_ + ^4^*J*_AY_| = 9.9 Hz, 2x N*C*H_2_), 59.4 (AXY, *N* = |^2^*J*_AX_ + ^4^*J*_AY_| = 9.6 Hz, 2x N*C*H_2_), 263 (m, 2x CO). ^**15**^**N{**^**1**^**H} NMR** (THF-d_8_, 50.7 MHz, [ppm]): δ = −0.69 (s). ^**31**^**P{**^**1**^**H] NMR** (THF-d_8_, 162 MHz,[ppm]): δ = 65.9 (s). **Elem. Anal.** found (calc) for C_42_H_88_Cl_2_N_4_O_2_P_4_W_2_: C 40.63 (40.56); H6.69 (7.13); N4.52 (4.51). **IR** (ATR-IR, cm^–1^): 1883 (ν_CO_); 1867 (ν_CO_). **rRaman** (λ_ex_ = 457 nm, frozen THF-d_8_, [cm^–1^]): ^14^N-**2**1437 (ν_NN_); ^15^N-**2** 1394 (ν_NN_).

#### [(N_2_){W(CO)(PNP)}_2_] (**3**)

##### 2

Complex (80 mg, 67 μmol, 1.0 equiv) and Na/Hg (2.2 g, 162 μmol, 2.4 equiv) are stirred for 12 h in benzene (20 mL) under the exclusion of light. After removal of the solvent *in vacuo*, the residue is extracted over *celite* with pentane to give **3** as a red-brown solid (45 mg, 57%). Crystals suitable for X-ray diffraction were obtained by layering a saturated THF solution with HMDSO. ^15^N-**3** was synthesized starting from ^15^N-**2**.

^**1**^**H{**^**31**^**P} NMR** (C_6_D_6_, 300 MHz, [ppm]): δ = 14.6 (s, CH*H*), 13.6 (s, CH*H*), 12.9 (s, CH*H*), 7.79 (s, CH*H*), 7.25 (s, ^*t*^Bu), 6.45 (s, CH*H*), 6.38 (s, ^*t*^Bu), 4.54 (s, ^*t*^Bu), 3.53 (s, ^*t*^Bu), −2.58 (s, CH*H*), −14.4 (s, CH*H*), −16.0 (s, CH*H*). **Elem. Anal.** found (calc) for C_42_H_88_N_4_O_2_P_4_W_2_: C 43.17 (43.01), H 7.23 (7.56), N 3.64 (4.78). (The lower N content found is attributed to partial N_2_ loss during combustion analysis.) **IR** (ATR-IR, cm^–1^): 1785 (ν_CO_); 1741 (ν_CO_). **μ**_**eff**_ = 2.4 ± 0.1 μ_B_. **rRaman** (λ_ex_ = 633 nm, frozen THF-d_8_, [cm^–1^]): ^14^N-**3** 1589 (ν_NN_); ^15^N-**3** 1540 (ν_NN_). **rRaman** (λ_ex_ = 514.5 nm, THF-d_8_, −50 °C [cm^–1^]): ^14^N-**3** 1571 (ν_NN_) 692 (ν_WN_) 491 (δ_WNN_); ^15^N-**3** 1522 (ν_NN_), 681 (ν_WN_) 479 (δ_WNN_).

#### [W(N)(CO)(PNP)] (**4**)

##### (a) Photolytic N_2_ Splitting

Complex **3** (10 mg, 8.53 μmol) is dissolved in C_6_D_6_ and photolyzed (λ = 427 nm, LED, Δλ = 10 nm) for 8 h in a water bath. The color changes from deep red to pale blue. After evaporation of the solvent, **4** is obtained in quantitative yield. The synthesis of ^15^N-**4** was carried out with ^15^N-**3**.

##### (b) Thermal N_2_ Splitting

Complex **3** (10 mg, 8.53 μmol) is dissolved in C_6_D_6_ and heated to 80 °C for 16 h with concomitant color change from deep red to pale blue.

^**1**^**H{**^**31**^**P} NMR** (C_6_D_6_, 500 MHz, [ppm]): δ = 3.90 (m, 2 H, NC*H*H), 3.76 (m, 2 H, NC*H*H), 1.79 (m, 2 H, PC*H*H), 1.55 (m, 2 H, PC*H*H), 1.49 (s, 18 H, 2x C(C*H*_3_)_3_), 0.89 (s, 18 H, 2x C(C*H*_3_)_3_). ^**13**^**C{**^**1**^**H} NMR** (C_6_D_6_, 126 MHz, [ppm]): δ = 24.5 (AXY, *N* = |^1^*J*_AX_ + ^3^*J*_AY_| = 18.5 Hz, 2x P*C*H_2_), 29.1 (AXY, *N* = |^2^*J*_AX_ + ^4^*J*_AY_| = 5.4 Hz, 2x C(*C*H_3_)_3_), 29.3 (AXY, *N* = |^2^*J*_AX_ + ^4^*J*_AY_| = 5.5 Hz, 2x C(*C*H_3_)_3_), 35.0 (AXY, *N* = |^1^*J*_AX_ + ^3^*J*_AY_| = 15.5 Hz, 2x *C*(CH_3_)_3_), 35.1 (AXY, *N* = |^1^*J*_AX_ + ^3^*J*_AY_| = 20.5 Hz, 2x *C*(CH_3_)_3_), 66.2 (AXY, *N* = |^2^*J*_AX_ + ^4^*J*_AY_| = 14.8 Hz, 2x N*C*H_2_), 283.4 (*t*, ^2^*J*_CP_ = 4.40 Hz, *C*O). ^**15**^**N{**^**1**^**H} NMR** (C_6_D_6_, 50.7 MHz, [ppm]): δ = 447.0 (s). ^**31**^**P{**^**1**^**H} NMR** (C_6_D_6_, 203 MHz, [ppm]): δ = 104.4 (s). **Anal. found (calc)** for C_21_H_44_N_2_OP_2_W: C 43.03 (43.01), H 7.53 (7.56), N 4.93 (4.78). **IR (ATR-IR, cm**^**–1**^**)**: 1883 (ν_CO_), 998 (ν_W≡N_).

#### Coupling of Complex **4**

Isolated **4** (5.2 mg, 8.87 μmol) was dissolved in toluene-d_8_, heated to 95 °C over 24 h under the exclusion of light and cooled to room temperature to freeze the equilibrium. ^1^H NMR spectroscopy confirmed the selective conversion of about 10% of **4** to dinuclear **3**.

#### [W(NCO)(CO)_2_(PNP)] (**5**)

Complex **4** (20 mg, 34.1 μmol) is dissolved in benzene. After degassing the solution by two freeze–pump–thaw cycles, the flask is backfilled with CO (1 atm) and solution stirred at room temperature. After 14 h, the solvent is removed *in vacuo* and the residue extracted through a plug of silanized silica 60. Evaporation of the solvent gives **5** as a deep purple solid (18.5 mg, 85%). The synthesis of ^15^N-**5** was carried out with ^15^N-**4**. Crystals suitable for X-ray diffraction were obtained by slow evaporation of a saturated Et_2_O solution at −40 °C.

^**1**^**H{**^**31**^**P} NMR** (C_6_D_6_, 500 MHz, [ppm]): δ = 1.02 (s, 18 H, 2x ^*t*^Bu), 1.21 (s, 18 H, 2x ^*t*^Bu), 1.76–1.88 (m, 4 H, 2x PC*H*H), 2.50–2.56 (m, 2 H, 2x PC*H*H), 2.95–3.01 (m, 2 H, 2x NC*H*H). ^**13**^**C{**^**1**^**H} NMR** (C_6_D_6_, 126 MHz, [ppm]): δ = 26.9 (AXY, *N* = |^1^*J*_AX_ + ^3^*J*_AY_| = 17.1 Hz, 2x P*C*H_2_), 29.8 (AXY, *N* = |^2^*J*_AX_ + ^4^*J*_AY_| = 4.5 Hz, 2x PC(*C*H_3_)_3_), 30.4 (AXY, *N* = |^2^*J*_AX_ + ^4^*J*_AY_| = 3.9 Hz, 2x PC(*C*H_3_)_3_), 37.3 (AXY, *N* = |^1^*J*_AX_ + ^3^*J*_AY_| = 14.3 Hz, 2x P*C*(CH_3_)_3_), 37.4 (AXY, *N* = |^1^*J*_AX_ + ^3^*J*_AY_| = 14.3 Hz, 2x P*C*(CH_3_)_3_), 68.2 (AXY, *N* = |^2^*J*_AX_ + ^4^*J*_AY_| = 10.8 Hz, 2x N*C*H_2_), 145 (s_br_, NCO), 261.8 (t, ^2^*J*_CP_ = 8.3 Hz, CO), 266.1 (t, ^2^*J*_CP_ = 4.40 Hz, CO). ^**15**^**N{**^**1**^**H} NMR** (C_6_D_6_, 50.7 MHz, [ppm]): δ = −347 (t, ^2^*J*_NP_ = 2.6 Hz). ^**31**^**P{**^**1**^**H} NMR** (C_6_D_6_, 162 MHz, [ppm]): δ = 76.6 (s). **Anal. found (calc)** for C_23_H_44_N_2_O_3_P_2_W: 42.97 (43.00), H 6.82 (6.90), N 4.37 (4.36). **IR (ATR-IR, cm**^**–1**^**):** 2205 (ν_NCO_), 1910 (ν_CO_), 1831 (ν_CO_). **LIFDI-MS** (*m*/*z*) found (calc) for [C_23_H_44_N_2_O_3_P_2_W]: 642.2 (642.2), 644.2 (644.2).

#### [W(NCO)(^13^CO)_2_(PNP)] (^13^CO-**5**)

Complex **4** (10.0 mg, 17.1 μmol) is dissolved in C_6_D_6_. After degassing the solution by two freeze–pump–thaw cycles, the flask is backfilled with ^13^CO (1 atm) and solution stirred at room temperature for 14 h. After removal of the solvent *in vacuo* the purple residue is extracted with Et_2_O over a plug of silanized silica 60. Evaporation of the solvent gives ^13^CO-**5** as a purple solid.

^**13**^**C{**^**1**^**H} NMR** (C_6_D_6_, 126 MHz, [ppm]): δ = 145 (s_br_, NCO), 261.8 (dt, ^2^*J*_CP_ = 8.5 Hz, ^2^*J*_CC_ = 8.5 Hz, CO), 266.1 (dt, ^2^*J*_CP_ = 4.40 Hz, ^2^*J*_CC_ = 9.0 Hz, CO). ^**31**^**P{**^**1**^**H} NMR** (C_6_D_6_, 162 MHz, [ppm]): δ = 76.6 (dd, ^2^*J*_CP_ = 8.4 Hz, ^2^*J*_CP_ = 4.3 Hz). **IR (ATR-IR, cm**^**–1**^**)**: 2205 (ν_NCO_), 1860 (ν_13CO_), 1762 (ν_13CO_). **LIFDI-MS** (*m*/*z*) found (calc) for [C_21_^13^C_2_H_44_N_2_O_3_P_2_W]: 644.2 (644.2), 646.2 (646.2).

#### [W(NCO)(CN^t^Bu)_2_(PNP)] (**6a**)

CN^*t*^Bu (7.8 μL, 5.7 mg, 69 μmol, 1.9 equiv) is added to a solution of **4** (21.3 mg, 36.3 μmol, 1.0 equiv) in benzene (20 mL). The mixture is heated to 85 °C for 3 h. After removal of the solvent *in vacuo*, the residue is extracted with benzene over silanized silica 60. After evaporation of the solvent, **6a** is obtained as a green solid (15.3 mg, 56%). Crystals suitable for X-ray diffraction were obtained by slow evaporation of a saturated Et_2_O solution at −40 °C.

^**1**^**H{**^**31**^**P} NMR** (C_6_D_6_, 300 MHz, [ppm]): δ = 3.10 (m, 2 H, NC*H*H), 2.76 (m, 2 H, NC*H*H), 1.99 (m, 2 H, PC*H*H), 1.90 (m, 2 H, PC*H*H), 1.46 (s, 9 H, CN-C*Me*_*3*_), 1.39 (s, 18 H, 2x C*Me*_*3*_), 1.27 (s, 18 H, 2x C*Me*_*3*_), 1.11 (s, 9 H, CN-C*Me*_*3*_). ^**13**^**C{**^**1**^**H} NMR** (C_6_D_6_, 126 MHz, [ppm]): δ = 27.4 (AXY, *N* = |^1^*J*_AX_ + ^3^*J*_AY_| = 14.0 Hz, 2x P*C*H_2_), 30.7 (s, 2x P(C*Me*_*3*_)_2_), 31.0 (s, 2x P(C*Me*_*3*_)_2_), 32.2 (s, CN-C*Me*_*3*_), 32.3 (s, CN-C*Me*_*3*_), 37.7 (AXY, *N* = |^1^*J*_AX_ + ^3^*J*_AY_| = 11.8 Hz, 2x P(*C*Me_3_)_2_), 39.0 (AXY, *N* = |^1^*J*_AX_ + ^3^*J*_AY_| = 12.6 Hz, 2x P(*C*Me_3_)_2_), 58.4 (s, CN-*C*Me_3_), 63.6 (s, CN-*C*Me_3_), 69.6 (AXY, *N* = |^2^*J*_AX_ + ^4^*J*_AY_| = 12.1 Hz, 2x N*C*H_2_), 143 (s_br_, N*C*O), 213 (s, *C*N-^*t*^Bu), 246 (s, CN-^*t*^Bu). ^**31**^**P{**^**1**^**H} NMR** (C_6_D_6_, 121 MHz, [ppm]): δ = 76.6 (s). **Elem. Anal.** found (calc) for C_37_H_58_N_4_O_3_P_2_W: C 49.65 (49.47), H 7.78 (8.30), N 7.00 (7.44). **IR (ATR-IR, cm**^**–1**^**):** ν = 2203 (ν_NCO_), 1994 (ν_C≡N_), 1832 (ν_C≡N_).

#### [W(NCO)(CNC_6_H_4_OMe)_2_(PNP)] (**6b**)

CNC_6_H_4_OMe (4.5 mg, 34.1 μmol, 2.0 equiv) is added to a solution of **4** (10.0 mg, 17.1 μmol, 1.0 equiv) in benzene (5 mL). The mixture is heated to 85 °C for 3 h. After removal of the solvent *in vacuo*, the residue is extracted with benzene through silanized silica 60. After evaporation of the solvent, **6b** is obtained as a yellow-brownish solid (8.4 mg, 58%). Crystals suitable for X-ray diffraction were obtained by slow evaporation of a saturated Et_2_O solution at −40 °C.

^**1**^**H{**^**31**^**P} NMR** (C_6_D_6_, 300 MHz, [ppm]): δ = 7.33 (d, ^3^*J*_HH_ = 8.99 Hz, 2 H, Ar-*H*), 6.78 (d, ^3^*J*_HH_ = 8.94 Hz, 2 H, Ar-*H*), 6.75 (d, ^3^*J*_HH_ = 8.91 Hz, 2 H, Ar-*H*), 6.67 (d, ^3^*J*_HH_ = 8.93 Hz, 2 H, Ar-*H*), 3.31 (m, 2 H, NC*H*H), 3.23 (s, 3 H, O*Me*), 3.21 (s, 3 H, O*Me*), 2.84 (m, 2 H, NC*H*H), 2.01 (m, 4 H, PC*H*H), 1.37 (s, 18 H, 2x P(C*Me*_3_)_2_), 1.18 (s, 18 H, 2x P(C*Me*_*3*_)_2_). ^**13**^**C{**^**1**^**H} NMR** (C_6_D_6_, 126 MHz, [ppm]): δ = 27.7 (AXY, *N* = |^1^*J*_AX_ + ^3^*J*_AY_| = 15.6 Hz, 2x P*C*H_2_), 30.5 (AXY, *N* = |^2^*J*_AX_ + ^4^*J*_AY_| = 5.21 Hz, 2x P(C*Me*_*3*_)_2_), 30.8 (AXY, *N* = |^2^*J*_AX_ + ^4^*J*_AY_| = 4.20 Hz, 2x P(C*Me*_*3*_)_2_), 36.0 (AXY, *N* = |^1^*J*_AX_ + ^3^*J*_AY_| = 13.8 Hz, 2x P(*C*Me_3_)_2_), 37.8 (AXY, *N* = |^1^*J*_AX_ + ^3^*J*_AY_| = 13.2 Hz, 2x P(*C*Me_3_)_2_), 55.0 (s, O-*Me*), 69.6 (AXY, *N* = |^2^*J*_AX_ + ^4^*J*_AY_| = 11.4 Hz, 2x N*C*H_2_), 115 (s, 2x ^Ar^C), 114 (s, 2x ^Ar^C), 122 (s, 2x ^Ar^C), 124 (s, 2x ^Ar^C), 135 (s, ^Ar^C_q_), 136 (t,^4^J_CP_ = 2.47 Hz, 2x ^Ar^C_q_), 143 (s_br_, N*C*O), 157 (s, ^Ar^C_q_), 158 (s, ^Ar^C_q_), 246 (s, *C*N-R), 257 (*C*N-R). ^**31**^**P{**^**1**^**H} NMR** (C_6_D_6_, 121 MHz, [ppm]): δ = 78.7 (s). **Elem. Anal.** found (calc) for C_31_H_62_N_4_OP_2_W: C 52.37 (52.12), H 6.30 (6.86), N 6.20 (6.57). **IR (ATR-IR, cm**^**–1**^**)**: ν = 2205 (ν_NCO_), 1911 (ν_C≡N_), 1757 (ν_C≡N_).

#### Na[W(CO)_2_(PNP)] (**7**)

Complex **5** (17.5 mg, 27.5 μmol, 1.0 equiv) and Na/Hg (823 mg, 60.5 μmol, 2.2 equiv) are stirred in THF for 4 h. The color changes from purple to bright orange. After filtration and evaporation of the solvent *in vacuo*, **5** is obtained as an orange solid (15 mg, 87%). After addition of 15-cr-5 (1.0 equiv), crystals suitable for X-ray diffraction were grown by diffusion of pentane into a saturated THF solution at −40 °C.

^**1**^**H{**^**31**^**P} NMR** (THF-d_8_, 500 MHz, [ppm]): δ = 3.22 (t, ^2^*J*_HH_ = 6.42 Hz, 4 H, NC*H*_2_), 1.94 (t, ^2^*J*_HH_ = 6.39 Hz, 4 H, PC*H*_2_), 1.32 (s, 36 H, 4x ^*t*^Bu). ^**13**^**C{**^**1**^**H} NMR** (THF-d_8_, 126 MHz, [ppm]): δ = 27.4 (AXY, *N* = |^1^*J*_AX_ + ^3^*J*_AY_| = 10.8 Hz, 2x P*C*H_2_), 30.9 (AXY, *N* = |^2^*J*_AX_ + ^4^*J*_AY_| = 6.3 Hz, 4x P(C*Me*_3_)_2_), 38.5 (AXY, *N* = |^1^*J*_AX_ + ^3^*J*_AY_| = 11.5 Hz, 4x P(*C*Me_3_)_2_), 66.4 (AXY, *N* = |^2^*J*_AX_ + ^4^*J*_AY_| = 19.7 Hz, 2x N*C*H_2_), 240 (s, 2x CO). ^**31**^**P{**^**1**^**H} NMR** (THF-d_8_, 121 MHz, [ppm]): δ = 105.4 (s). **Elem. Anal.** found (calc) for C_22_H_44_NNaO_2_P_2_W: C 42.35 (42.39), H 6.97 (7.11), N 2.21 (2.25). **IR (ATR-IR, cm**^**–1**^**)**: ν = 1677 (ν_CO_), 1604 (ν_CO_).

#### [WCl(CO)_2_(PNP)] (**8**)

Me_3_SiCl (1.0 μL, 0.9 mg, 7.8 μmol, 1.0 equiv) is added to a solution of **5** (5.0 mg, 7.8 μmol, 1.0 equiv) in THF-d_8_ (0.5 mL). The solution is stirred overnight. **8** and Me_3_SiNCO are obtained as products in quantitative spectroscopic yield after separation by vacuum trap-to-trap transfer. Crystals suitable for X-ray diffraction were obtained by slow evaporation of a saturated Et_2_O solution at −40 °C.

^**1**^**H{**^**31**^**P} NMR** (C_6_D_6_, 300 MHz, [ppm]): 3.13–2.99 (m, 2 H, NC*H*H), 2.70–2.58 (m, 2 H, NC*H*H), 2.05–1.84 (m, 4 H, PC*H*_*2*_), 1.34 (s, 18 H, CMe_3_), 1.10 (s, 18 H, CMe_3_). ^**13**^**C{**^**1**^**H} NMR** (C_6_D_6_, 126 MHz, [ppm]): δ = 26.9 (AXY, *N* = |^1^*J*_AX_ + ^3^*J*_AY_| = 17.1 Hz, 2x P*C*H_2_), 30.1 (AXY, *N* = |^2^*J*_AX_ + ^4^*J*_AY_| = 4.6 Hz, 2x PC(*C*H_3_)_3_), 31.0 (AXY, *N* = |^2^*J*_AX_ + ^4^*J*_AY_| = 4.0 Hz, 2x PC(*C*H_3_)_3_), 37.8 (AXY, *N* = |^1^*J*_AX_ + ^3^*J*_AY_| = 13.6 Hz, 2x P*C*(CH_3_)_3_), 38.5 (AXY, *N* = |^1^*J*_AX_ + ^3^*J*_AY_| = 14.6 Hz, 2x P*C*(CH_3_)_3_), 67.9 (AXY, *N* = |^2^*J*_AX_ + ^4^*J*_AkY_| = 11.0 Hz, 2x N*C*H_2_), 259 (t, ^2^*J*_CP_ = 8.7 Hz, CO), 264 (t, ^2^*J*_CP_ = 4.8 Hz, CO). ^**31**^**P{**^**1**^**H} NMR** (C_6_D_6_, 162 MHz, [ppm]): δ = 73.9 (s). **Anal. found (calc)** C_22_H_44_ClNO_2_P_2_W: C 41.35 (41.56); H 7.00 (6.98); N 2.19 (2.20). **IR (ATR-IR, cm**^**–1**^**):** 1914 (ν_CO_), 1815 (ν_CO_). *(a) Characterization of TMS-NCO*. ^**1**^**H NMR** (THF-d_8_, 300 MHz, [ppm]): δ = 0.25 (s, 9 H, Si(C*H*_3_)_3_). ^**13**^**C{**^**1**^**H} NMR** (THF-d_8_, 126 MHz, [ppm]): δ = 0.79 (s, 3 C, Si(*C*H_3_)_3_). ^**29**^**Si{**^**1**^**H} NMR** (THF-d_8_, 90.4 MHz, [ppm]): δ = 4.5 (s). *(b) Characterization of TMS-*^*15*^*NCO*. ^**1**^**H NMR** (THF-d_8_, 500 MHz, [ppm]): δ = 0.25 (d, ^3^*J*_HN_ = 1.4 HZ, 9 H, Si(C*H*_3_)_3_). ^**13**^**C{**^**1**^**H} NMR** (THF-d_8_, 126 MHz, [ppm]): δ = 0.79 (d, ^2^*J*_CN_ = 2.8 Hz, 3 C, Si(*C*H_3_)_3_). ^**15**^**N{**^**1**^**H} NMR** (THF-d_8_, 50.7 MHz, [ppm]): δ = −346 (s). ^**29**^**Si{**^**1**^**H} NMR** (THF-d_8_, 90.4 MHz, [ppm]): δ = 4.5 (d, ^1^*J*_SiN_ = 14.2 Hz).

#### Regeneration of [(WCl_3_(PNP)}] (**9**) from **8**

Complex **8** (6.4 mg, 10.1 μmol, 1.0 equiv) and *N*-chlorosuccinimide (3.0 mg, 22.1 μmol, 2.2 equiv) are dissolved in C_6_D_6_ (0.5 mL) and photolyzed (λ > 305 nm) for 3 h. The color changes from deep purple to dark yellow and a dark precipitate forms. After removal of all volatiles *in vacuo* the residue is dissolved in a solution of C_6_D_6_ (0.5 mL) and 1,3,5-trimethoxybenzene as internal standard. **9** is obtained in 30% spectroscopic yield.

## Supporting Information Available

Spectroscopic, kinetic, magnetic, crystallographic, and computational data (PDF, CIFs)

## Supplementary Material

au1c00117_si_001.pdf

au1c00117_si_002.cif

au1c00117_si_003.cif

au1c00117_si_004.cif

au1c00117_si_005.cif

au1c00117_si_006.cif

au1c00117_si_007.cif

au1c00117_si_008.cif
