# Supplementary material for: Cyanate Formation via Photolytic Splitting of Dinitrogen
Source: JACS Au. 2021 May 20;1(6):879–94. doi: 10.1021/jacsau.1c00117 (PMC8243327; doi:10.1021/jacsau.1c00117)
Supplement: Supplementary file 1 — au1c00117_si_001.pdf [file au1c00117_si_001.pdf]

Supporting Information

Cyanate Formation via Photolytic Splitting of Dinitrogen

Bastian Schluschaß,[a] Jan-Hendrik Borter,#,[b] Severine Rupp,#,[c] Serhiy Demeshko,[a] Christian Herwig,[d] Christian Limberg,[d] Nicholas A. Maciulis,[e] Jessica Schneider,[a] Christian Würtele,[a] Vera Krewald,*[c] Dirk Schwarzer*[b] and Sven Schneider*,[a]

[a] University of Göttingen, Institute for Inorganic Chemistry
Tammannstraße 4, 37077 Göttingen, Germany

[b] Department of Dynamics at Surfaces, Max Planck Institute for Biophysical Chemistry,
Am Fassberg 11, 37077 Göttingen, Germany

[c] Theoretische Chemie, Technische Universität Darmstadt
Alarich-Weiss-Str. 4, 64287 Darmstadt, Germany

[d] Institut für Chemie, Humboldt Universität zu Berlin
Brook-Taylor-Strasse 2, 12489 Berlin, Germany

[e] Department of Chemistry, Indiana University
800 East Kirkwood Avenue, Indiana 47405-7102, USA

* krewald@chemie.tu-darmstadt.de; dschwar@gwdg.de; sven.schneider@chemie.uni-goettingen.de.

Table of Contents

1. Experimental ... 4

1.1 Kinetic Analysis ... 4

1.1.1 Reaction Monitoring ... 4

1.1.2 Kinetic modelling ... 4

1.1.3 Eyring Analysis ... 5

1.1.4 Van't Hoff Analysis ... 6

2. Spectroscopic Results ... 7

2.1 Characterization ... 7

2.1.1 [(N_2){WCl(CO)(PNP)}$_2$] (**2**) ... 7

2.1.2 [(N_2){W(CO)(PNP)}$_2$] (**3**) ... 10

2.1.3 [W(N)(CO)(PNP)] (**4**) ... 13

2.1.4 Coupling of [W(N)(CO)(PNP)] (**4**) ... 14

2.1.5 [W(NCO)(CO)$_2$(PNP)] (**5**) ... 15

2.1.6 [W(NCO)(^{13}CO)$_2$(PNP)] (^{13}CO-**5**) ... 17

2.1.7 [W(NCO)(CN-^{t}Bu)$_2$(PNP)] (**6a**) ... 18

2.1.8 [W(NCO)(CNC$_6$H$_4$OMe)$_2$(PNP)] (**6b**) ... 19

2.1.9 Na[W(CO)$_2$(PNP)] (**7**) ... 21

2.1.10 [WCl(CO)$_2$(PNP)] (**8**) ... 23

2.2. Quantum Yield Determination ... 25

2.2.1 Quantum Yield Determination at r.t. ... 25

2.2.1 Quantum Yield Determination at variable temperatures ... 26

2.3 Transient Spectroscopy ... 27

2.3.1 Transient UV/Vis spectra ... 27

2.3.2 Transient IR spectra ... 30

3. Crystallographic Details ... 31

3.1 Crystal Structure of **2** ... 31

3.2 Crystal Structure of **3** ... 38

3.3 Crystal Structure of **5** ... 44

3.4 Crystal Structure of **6a** ... 48

3.5 Crystal Structure of **6b** ... 53

3.6 Crystal Structure of **7** ... 59

3.7 Crystal Structure of **8** ... 65

4. Computational Details ... 69

4.1 Computational details ... 69

4.2 Geometries and Mayer bond orders ... 69

4.3 Computed UV-vis spectra 71
4.4 Thermochemistry data 85
4.4.1 Thermodynamics and kinetics for thermal dissociation of tungsten dimer for various functionals 85
4.4.2 Thermodynamics and kinetics for thermal dissociation of tungsten dimer for selected functionals with extended basis sets 88
4.5 Cartesian coordinates of ground state structures (Å) 91
4.6 Cartesian coordinates of the transition state structures (Å) 103
References 116

1. Experimental

1.1 Kinetic Analysis

1.1.1 Reaction Monitoring

0.5 mL of a stock solution of **3** (3.75 mM in toluene-d_8) in a J-Young NMR tube was inserted into the preheated NMR spectrometer. The progress of the reaction was monitored *via* ^{1}H NMR at different temperatures (75, 85, 95, 105 °C). Every experiment was repeated at least twice.

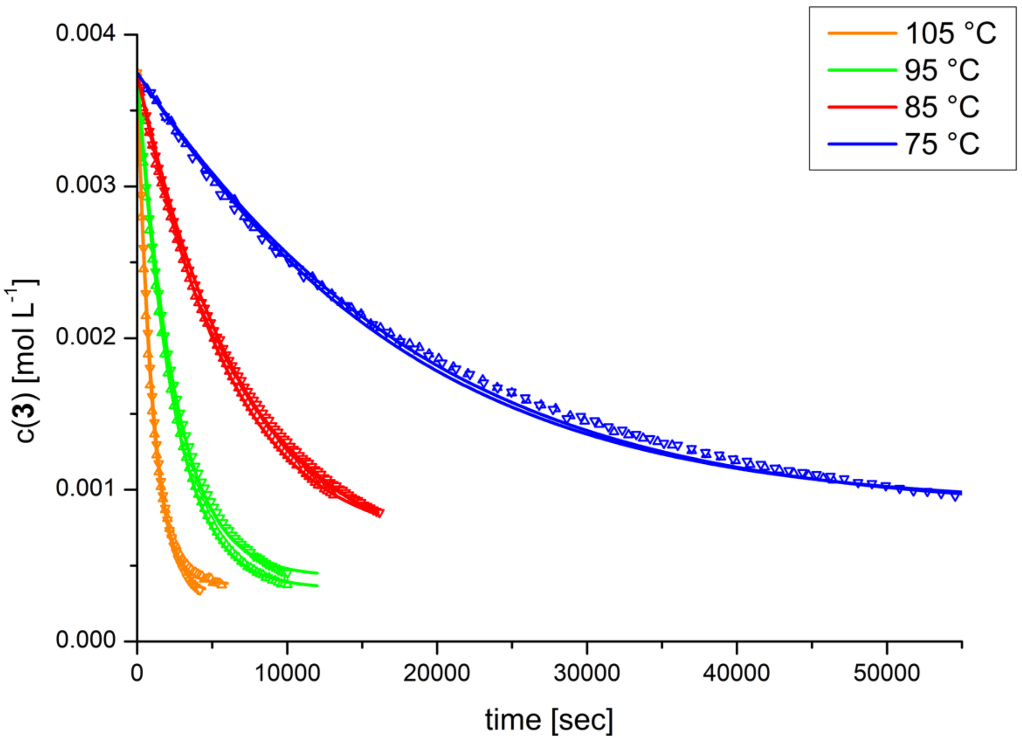

Figure S1 Concentration *vs.* time plot for the decay of **3** upon heating to 75, 85, 95 or 105 °C. Each triangle represents one measurement point, while the solid lines represent the fitting curves.

1.1.2 Kinetic modelling

The kinetic profiles were fitted using the COPASI 4.27 (Build 213) software[1] according to the model shown in **Scheme S1**. The fitted rate constants k_1 and k_2 and the equilibrium constant $K = k_1/k_2$ at different temperatures are given in **Table S1**.

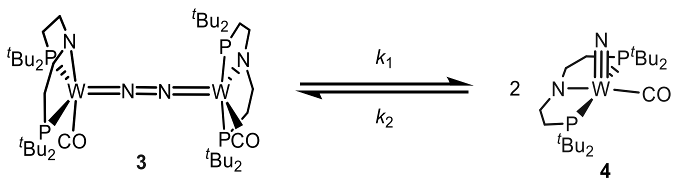

$$\frac{d[\mathbf{3}]}{dt} = -k_1[\mathbf{3}] + k_2[\mathbf{4}]^2$$

Scheme S1. The model used for fitting the kinetic data.

Table S1 Rate constants k_1 and k_2 and equilibrium constant K for the dissociation of **3**.

T [K]	k_1 [s^{-1}]	k_2 [$L\ mol^{-1}\ s^{-1}$]	K [$mol\ L^{-1}$]
348	$3.0 \cdot 10^{-5}$	$7.7 \cdot 10^{-4}$	0.039
348	$3.2 \cdot 10^{-5}$	$8.63 \cdot 10^{-4}$	0.037
358	$1.0 \cdot 10^{-4}$	$16.4 \cdot 10^{-4}$	0.061
358	$0.9 \cdot 10^{-4}$	$14.5 \cdot 10^{-4}$	0.062
368	$3.9 \cdot 10^{-4}$	$35.5 \cdot 10^{-4}$	0.110
368	$3.8 \cdot 10^{-4}$	$36.9 \cdot 10^{-4}$	0.103
378	$9.8 \cdot 10^{-4}$	$82 \cdot 10^{-4}$	0.120
378	$10 \cdot 10^{-4}$	$76 \cdot 10^{-4}$	0.132

1.1.3 Eyring Analysis

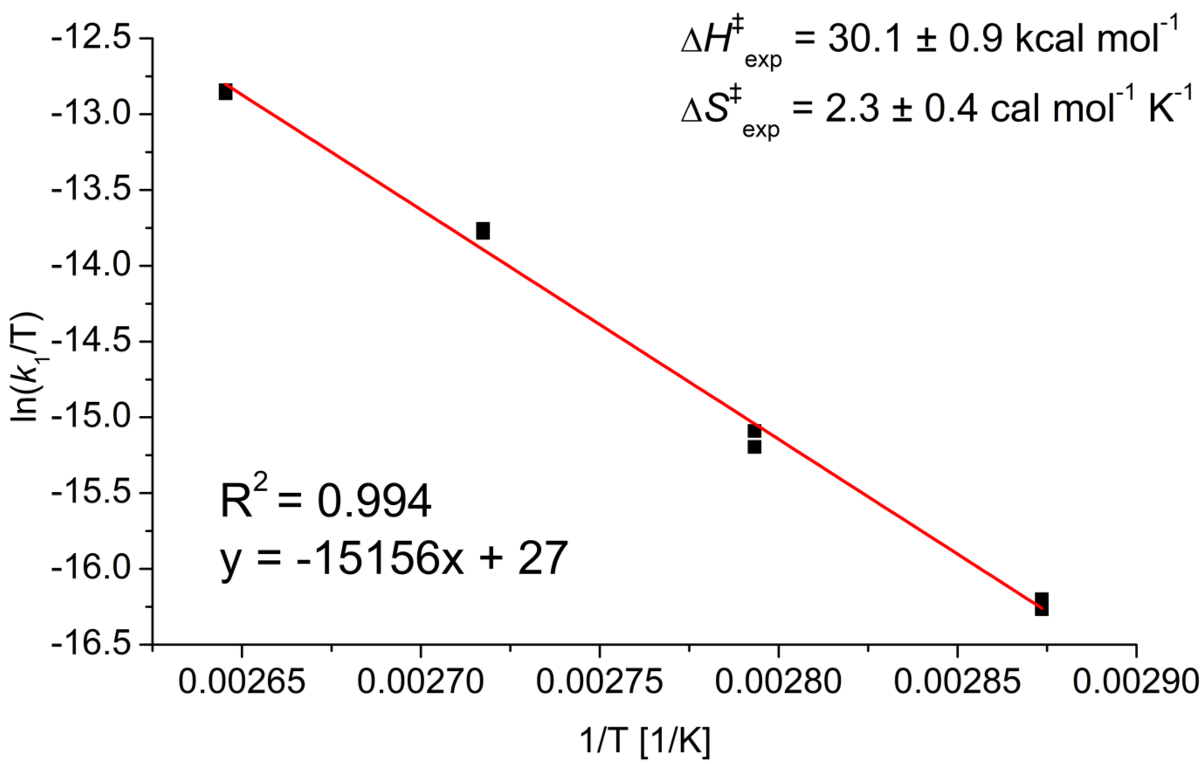

Figure S2. Eyring plot for the dissociation of **3** to **4**.

1.1.4 Van't Hoff Analysis

For Van't Hoff analysis, the equilibrium constants *K*(T) were obtained from the fitted parameters k_1 and k_2 (**Table S1**) and directly from the experimental equilibrium concentrations of **3** at different temperatures (75, 85, 95 °C).

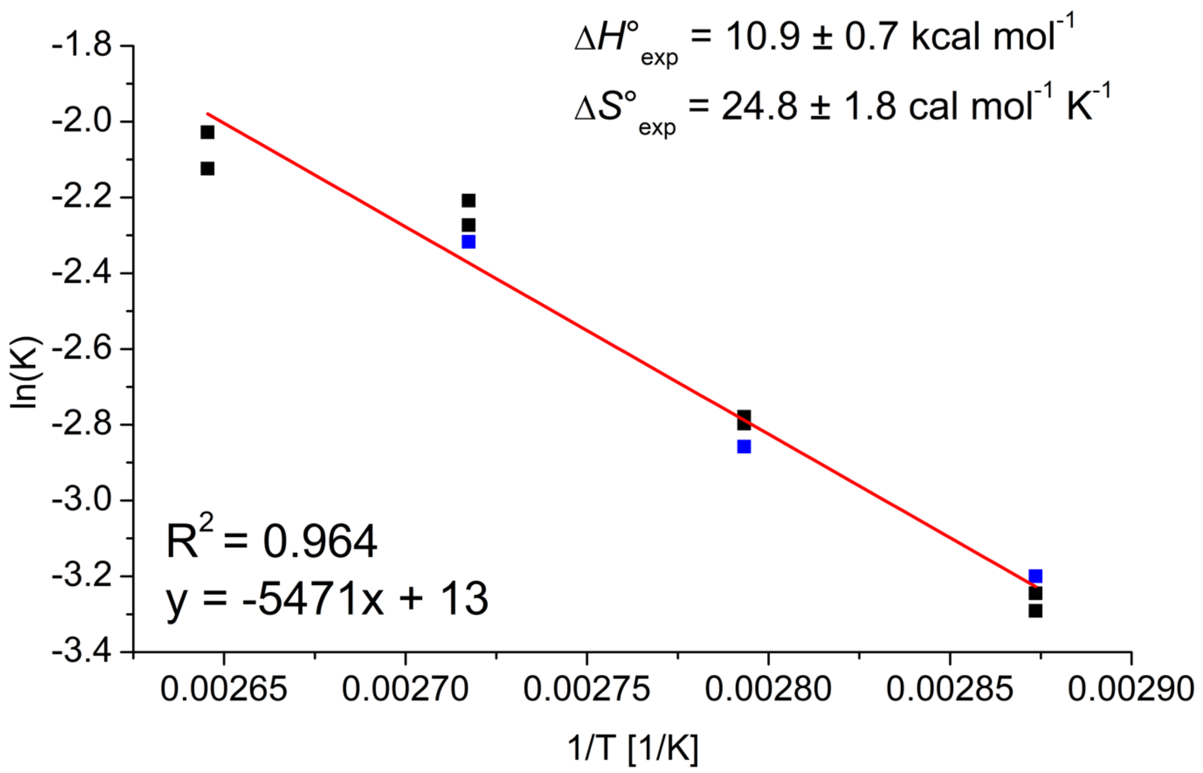

Figure S3. *Van't Hoff* plot for the splitting reaction of **3** to **4**. The black data points were obtained from the kinetic model ($K = k_1/k_2$) and the blue data points from the concentrations after equilibration at the respective temperature.

2. Spectroscopic Results

2.1 Characterization

2.1.1 [(N_2){WCl(CO)(PNP)}$_2$] (**2**)

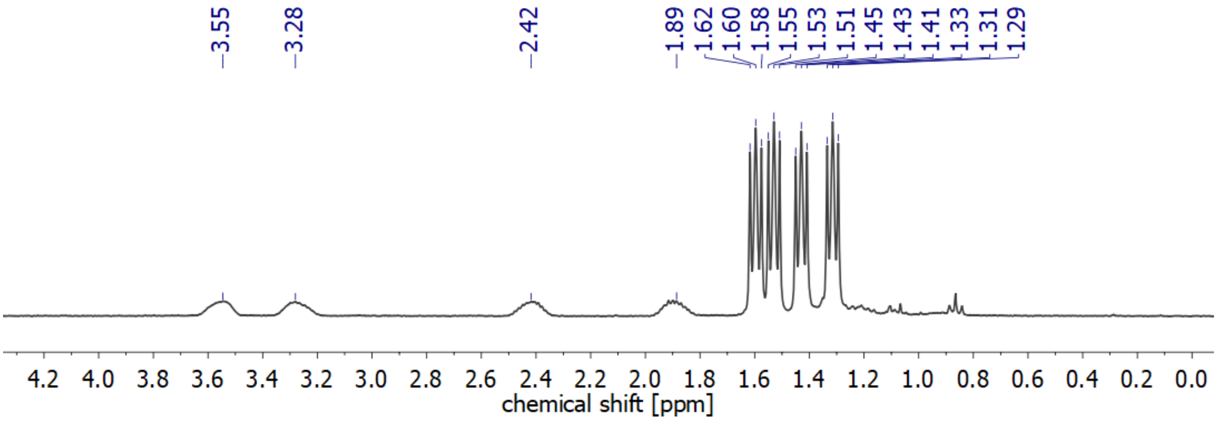

Figure S4. ^{1}H NMR spectrum of **2** in C_6D_6 at room temperature.

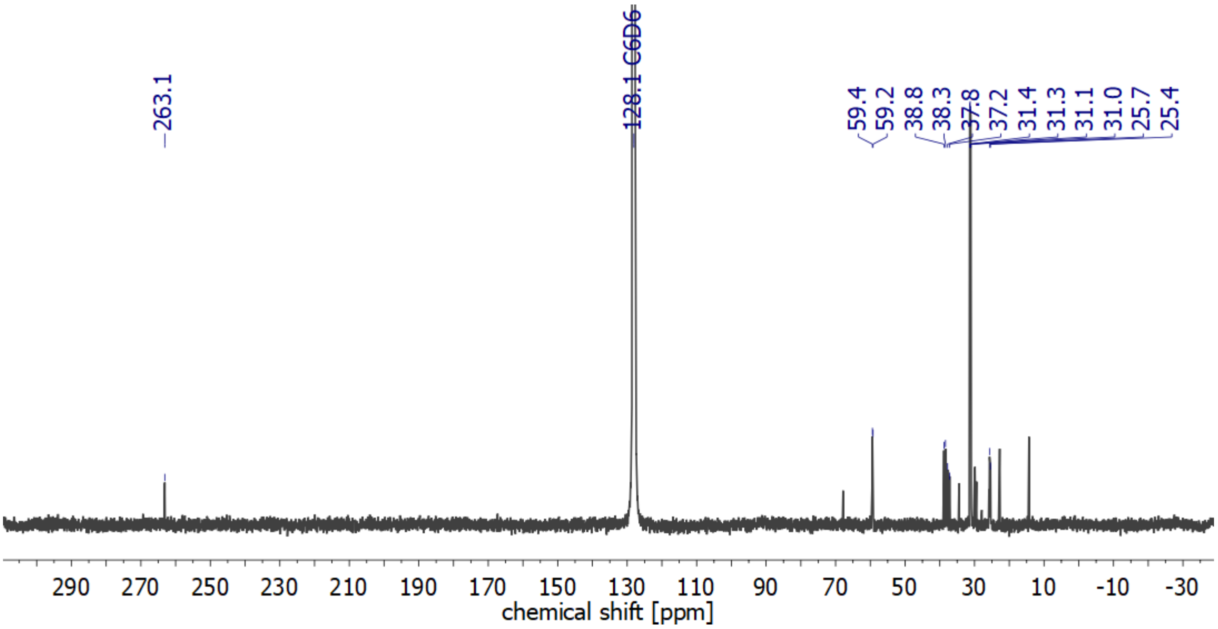

Figure S5. ^{13}C{^{1}H} NMR spectrum of **2** in C_6D_6 at room temperature.

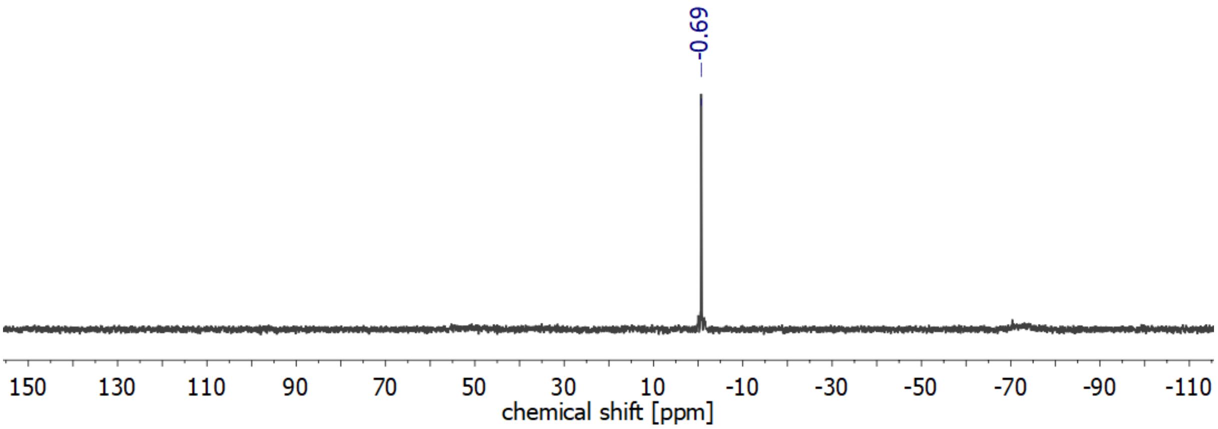

Figure S6. ^{15}N{^{1}H} NMR spectrum of ^{15}N-**2** in C_6D_6 at room temperature.

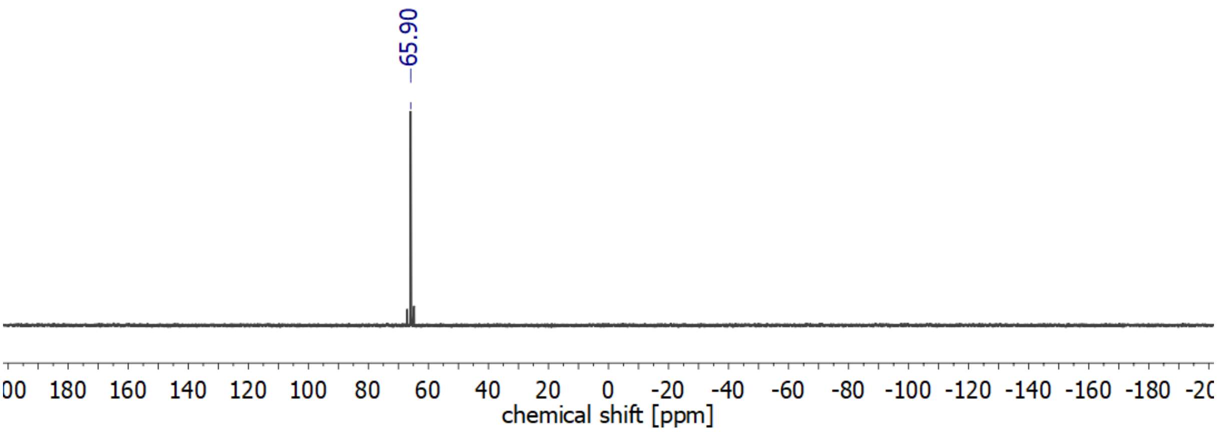

Figure S7. $^{31}P\{^{1}H\}$ NMR spectrum of **2** in C_6D_6 at room temperature.

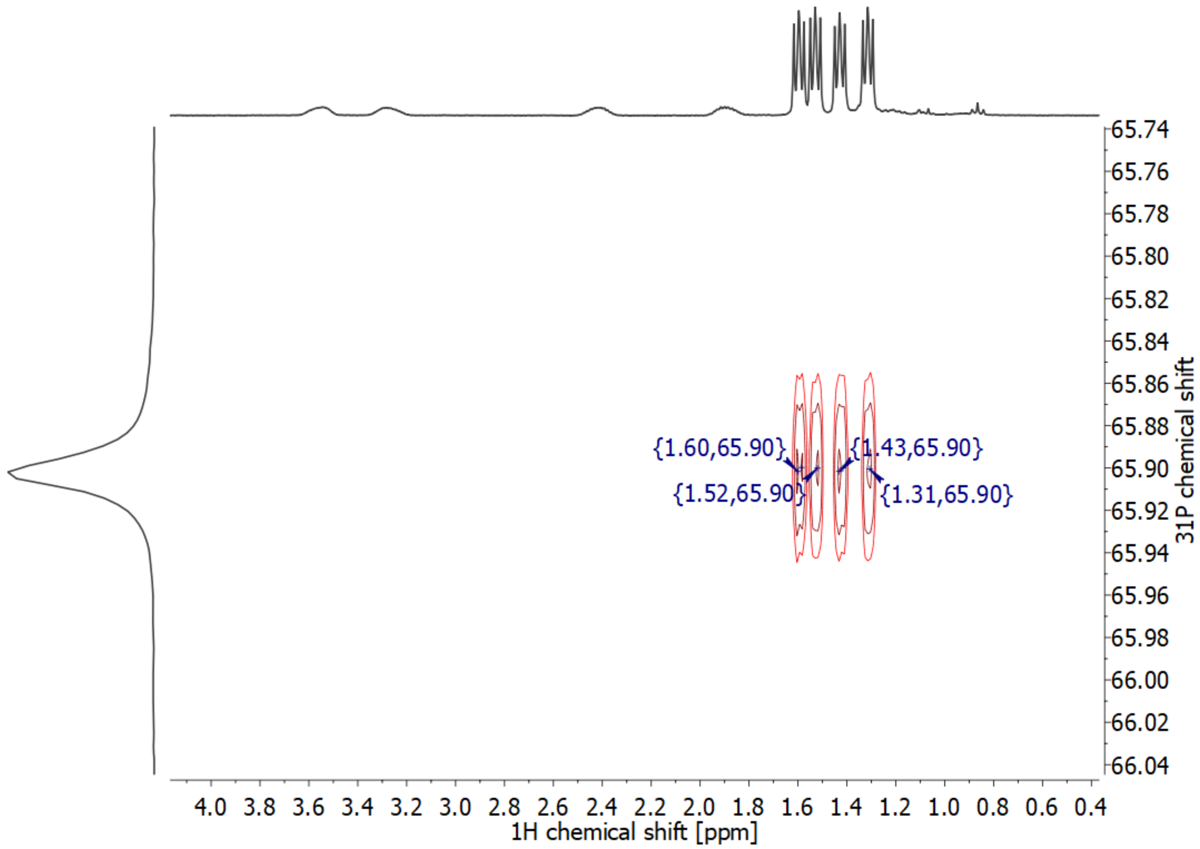

Figure S8. ^{1}H-^{31}P HMBC NMR spectrum of **2** in C_6D_6 at room temperature.

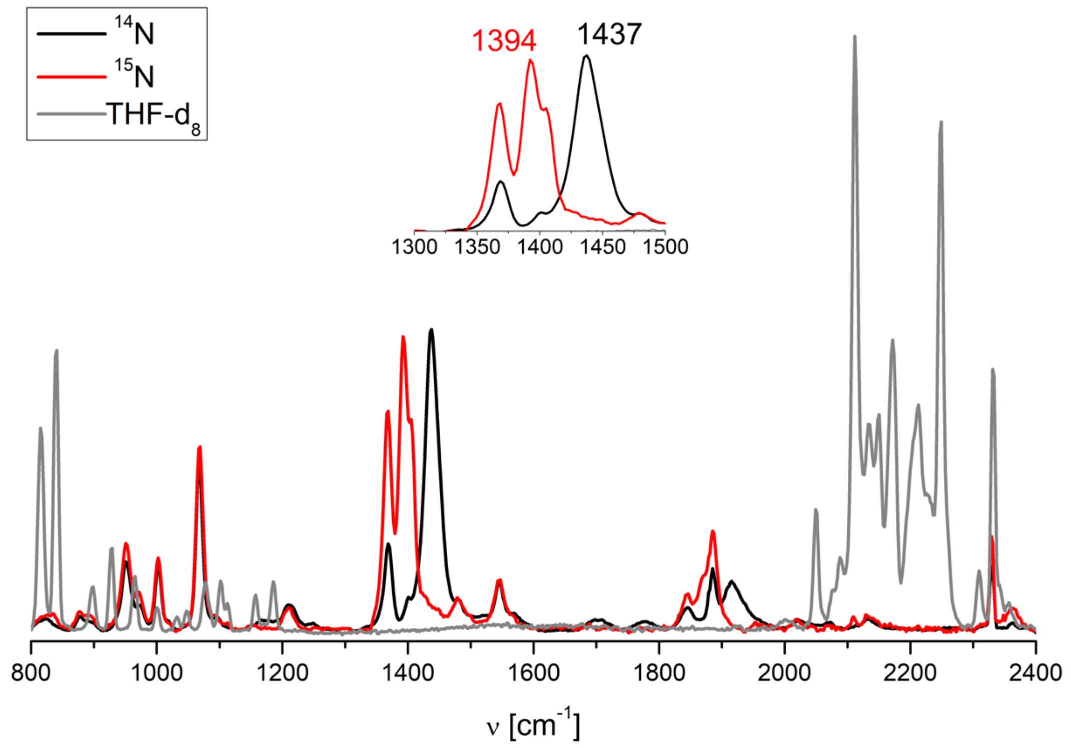

Figure S9. rRaman (λ = 457 nm) spectrum of **2** and ^{15}N-**2** in frozen THF-d8.

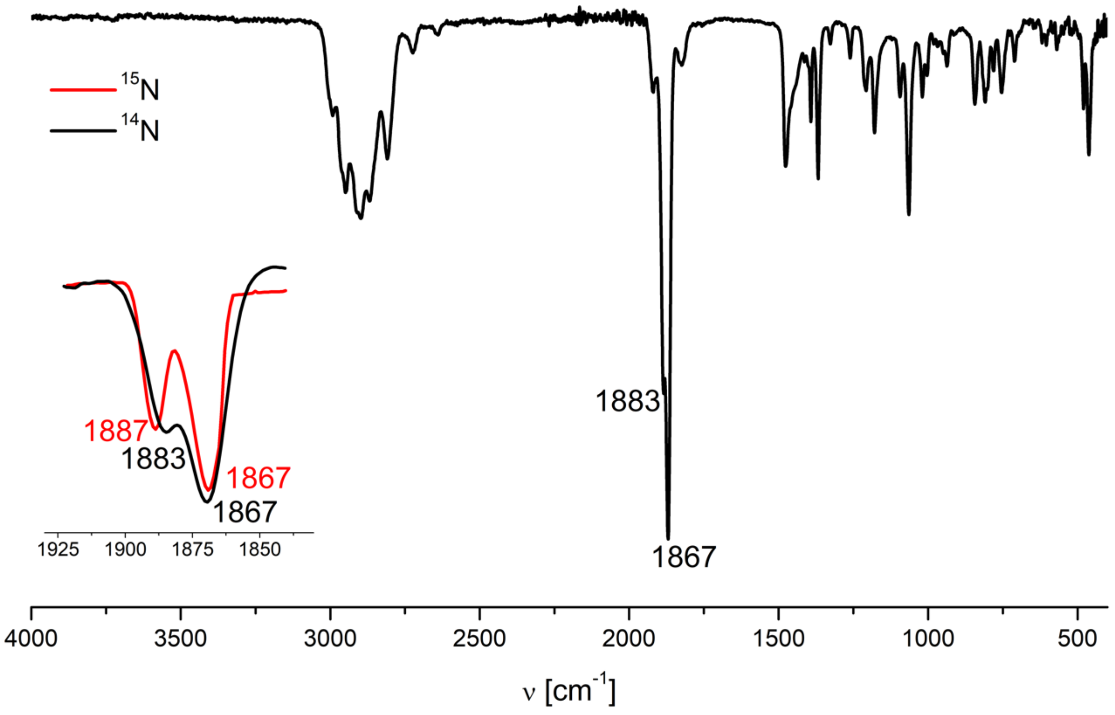

Figure S10. ATR-IR spectrum of solid **2** at room temperature.

2.1.2 [$(N_2)\{W(CO)(PNP)\}_2$] (**3**)

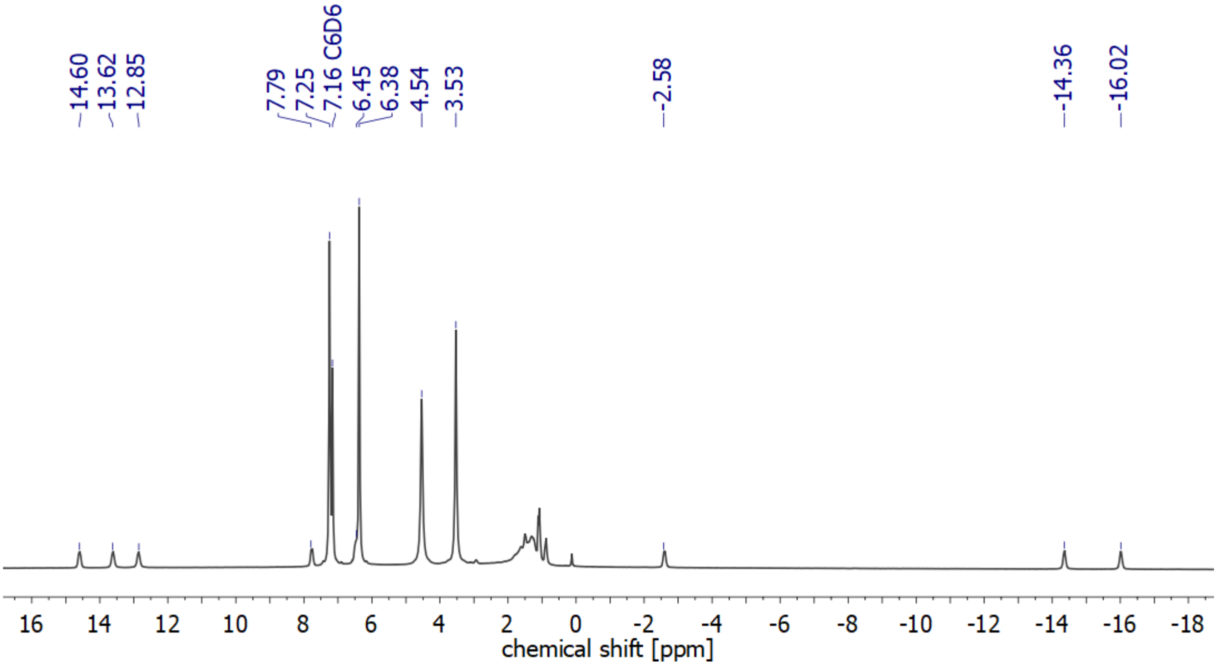

Figure S11. ^1H NMR spectrum of **3** in C_6D_6 at room temperature.

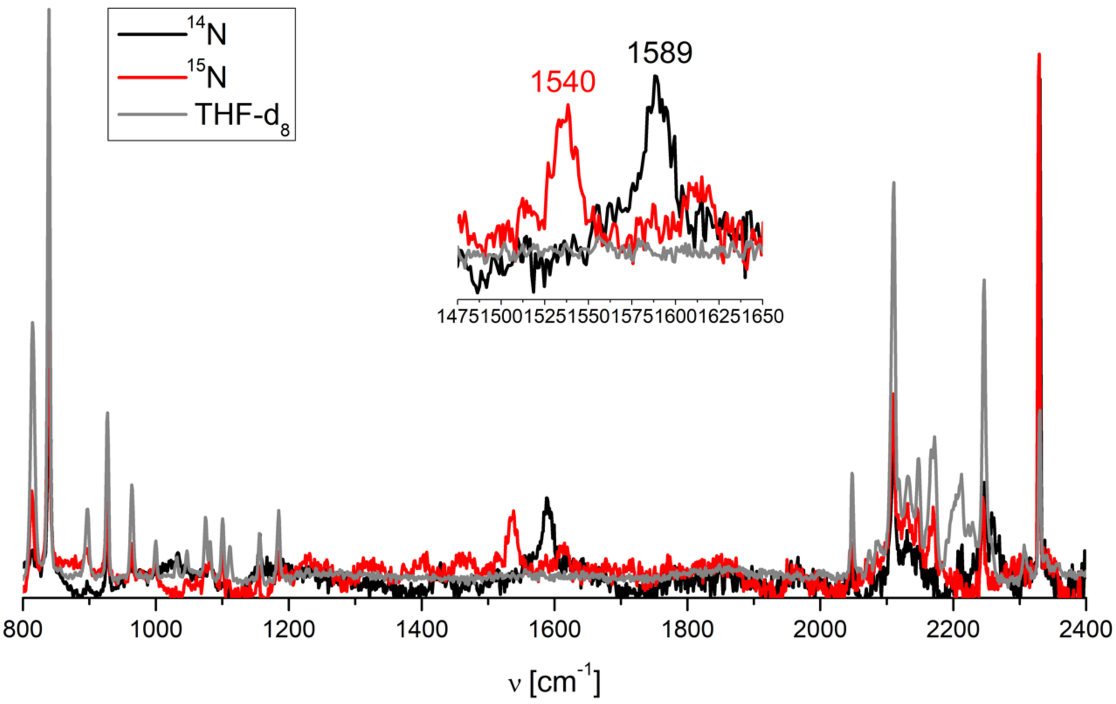

Figure S12. rRaman (λ = 633 nm) spectrum of **3** and ^{15}N-**3** in frozen THF-d_8.

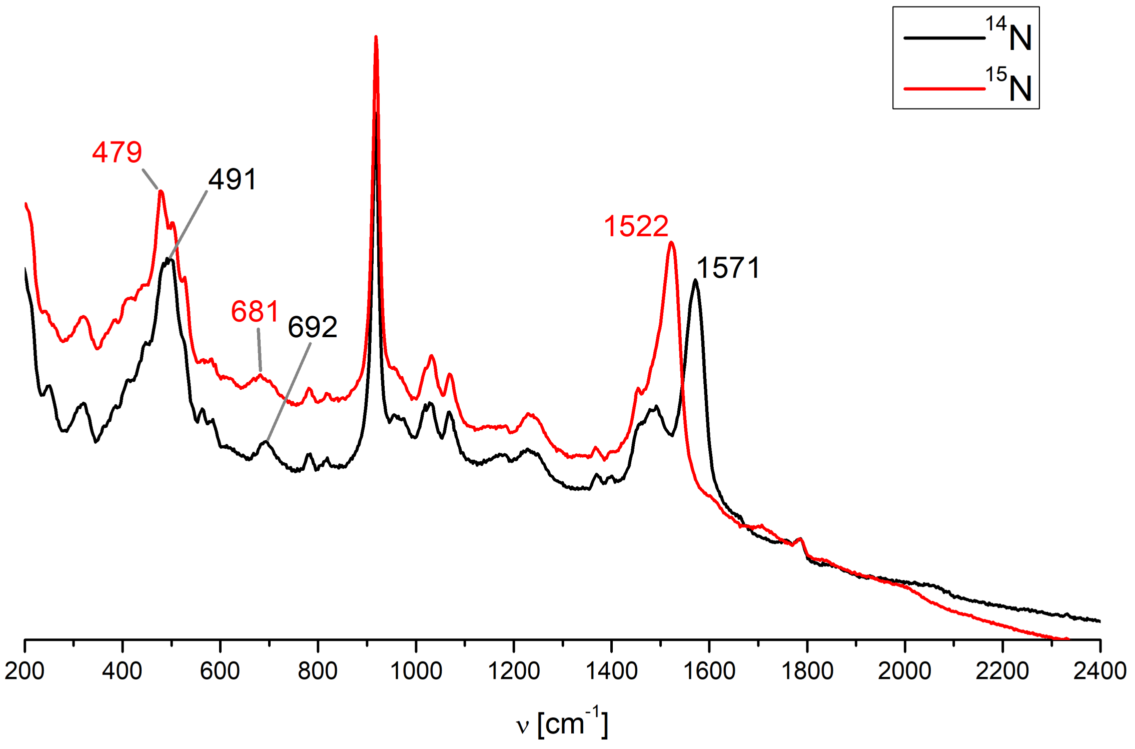

Figure S13. rRaman (λ = 514.5 nm) spectrum of **3** and ^{15}N-**3** in THF at -50 °C.

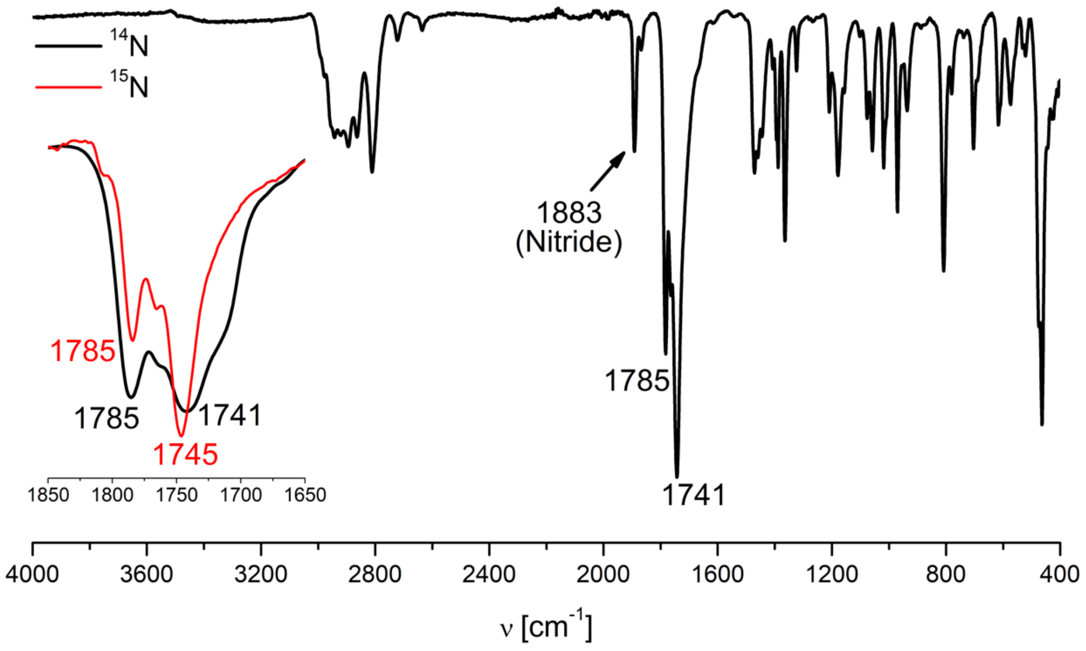

Figure S14. ATR-IR spectrum of solid **3** at room temperature.

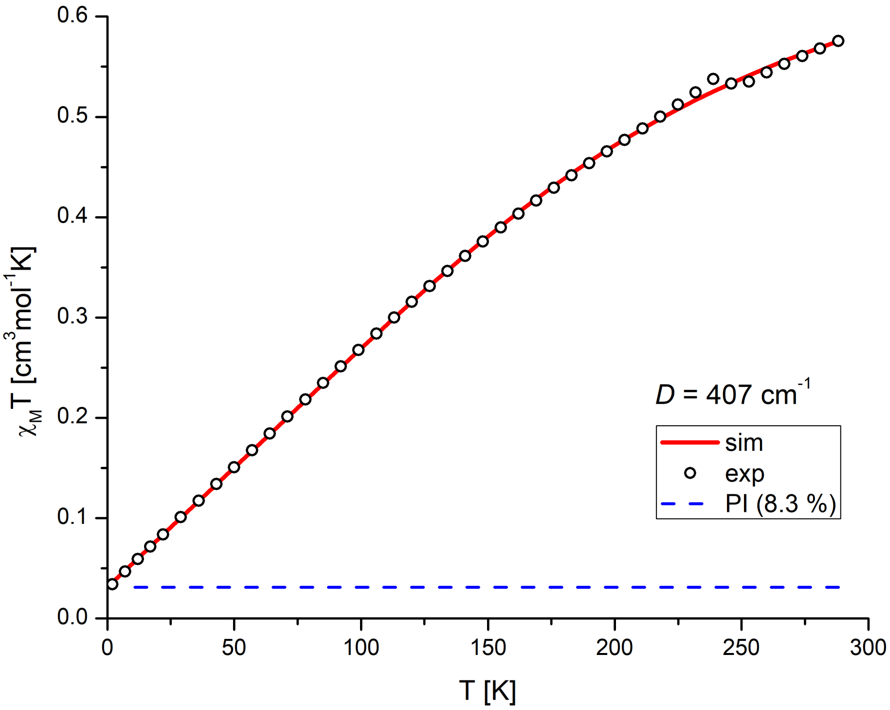

Figure S15. $\chi_M T$ vs. T plot for **3**. Open circles represent experimental data and the red solid line corresponds to the best fit using a Hamiltonian that includes Zeemann splitting and an axial Zero-Field-Splitting term (eqn. 2.1) with fit parameters g_{av} = 1.74, D = 407 cm^{-1} and 8.3 % of a paramagnetic impurity with S = 1/2 (PI, blue dashed line).

$$\hat{H} = g\mu_B \vec{B}\vec{S} + D\left[\hat{S}_z^2 - \frac{1}{3}S(S+1)\right] \qquad 2.1$$

2.1.3 [W(N)(CO)(PNP)] (**4**)

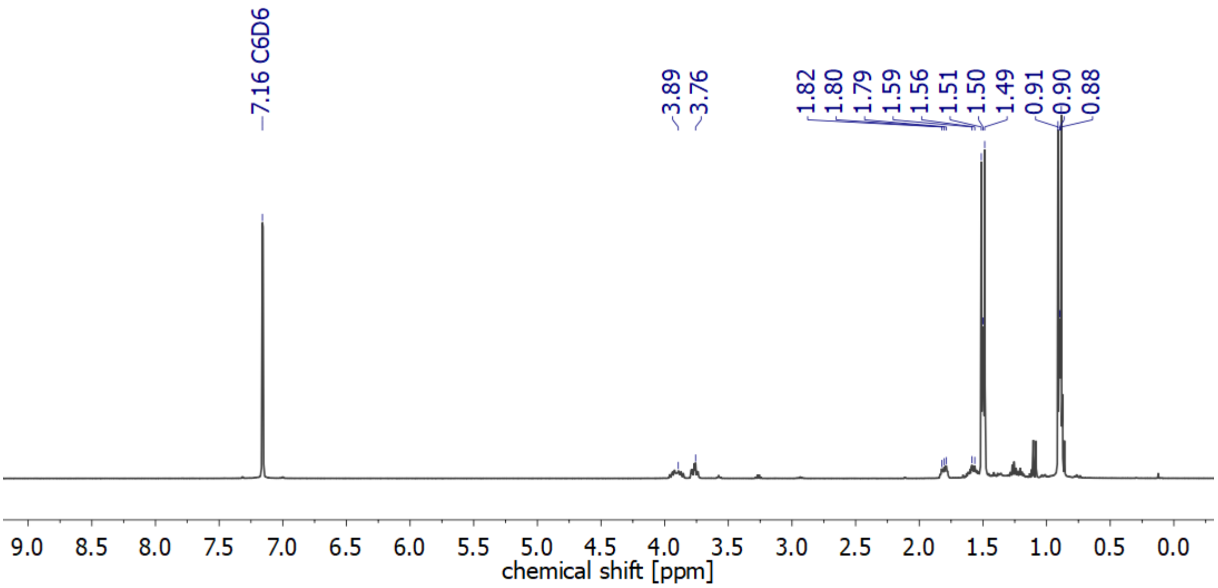

Figure S16. ^{1}H NMR spectrum of **4** in C_6D_6 at room temperature.

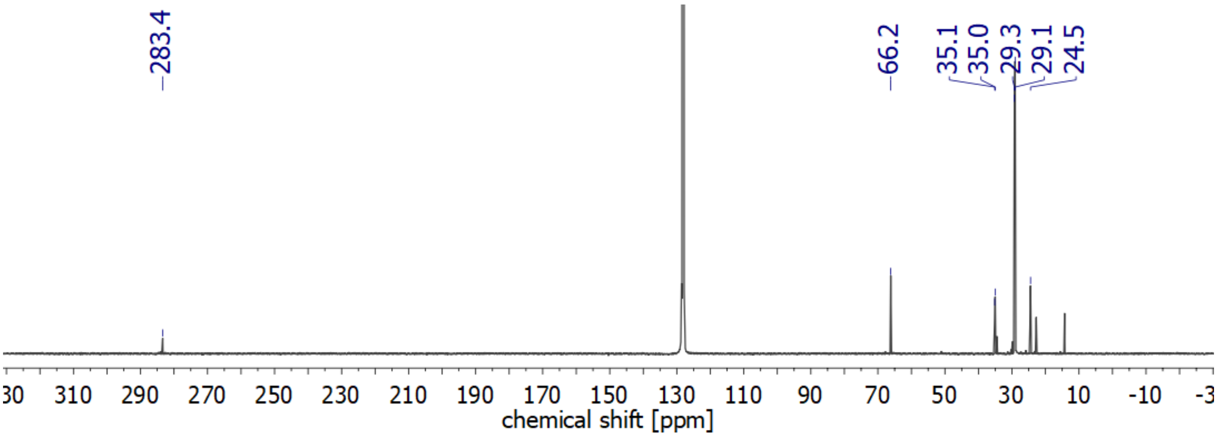

Figure S17. $^{13}C\{^{1}H\}$ NMR spectrum of **4** in C_6D_6 at room temperature.

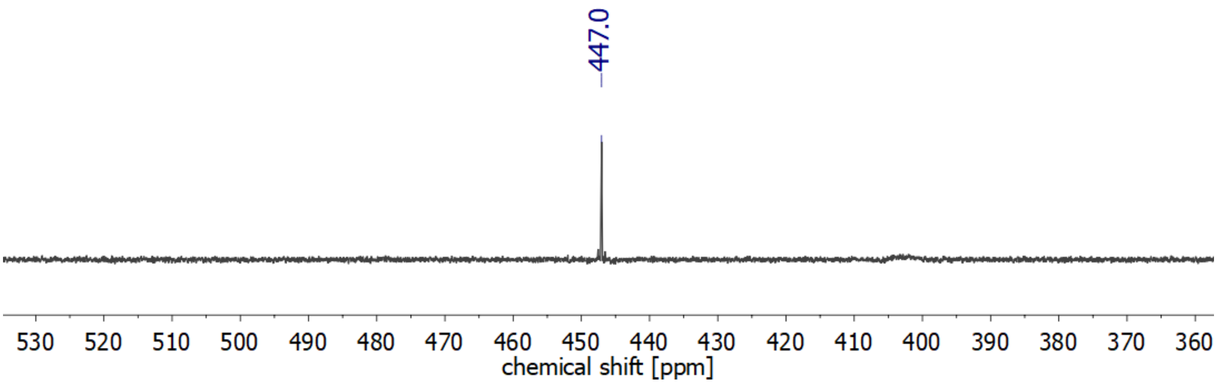

Figure S18. $^{15}N\{^{1}H\}$ NMR spectrum of ^{15}N-**4** in C_6D_6 at room temperature.

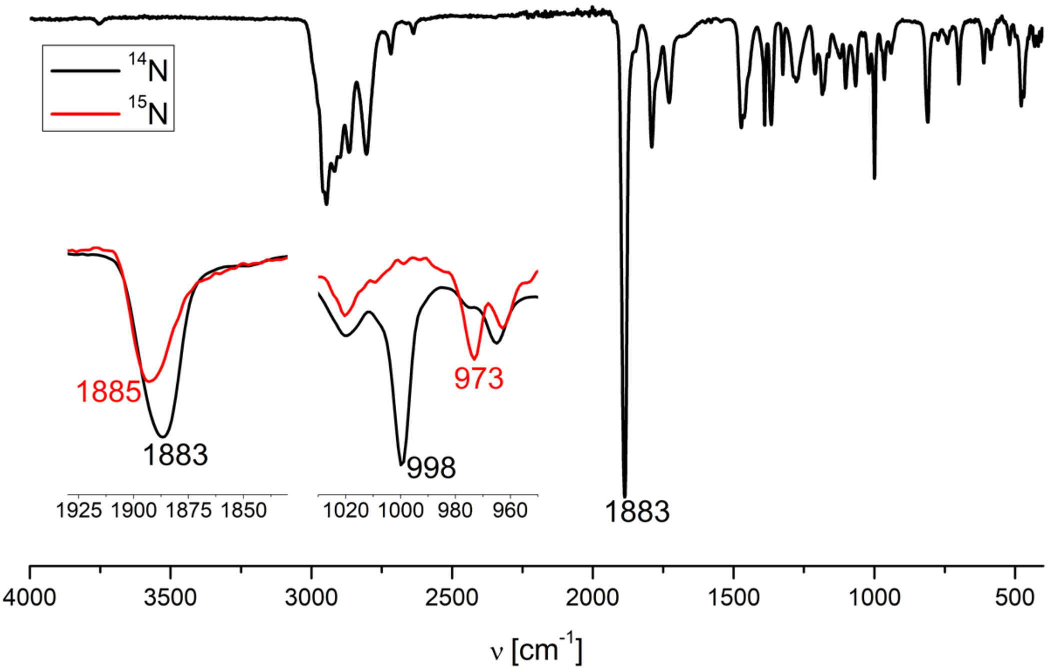

Figure S19. ATR-IR spectrum of solid **4** at room temperature. The inlays show a comparison between **4** (black) and ^{15}N-**4** (red).

2.1.4 Coupling of [W(N)(CO)(PNP)] (**4**)

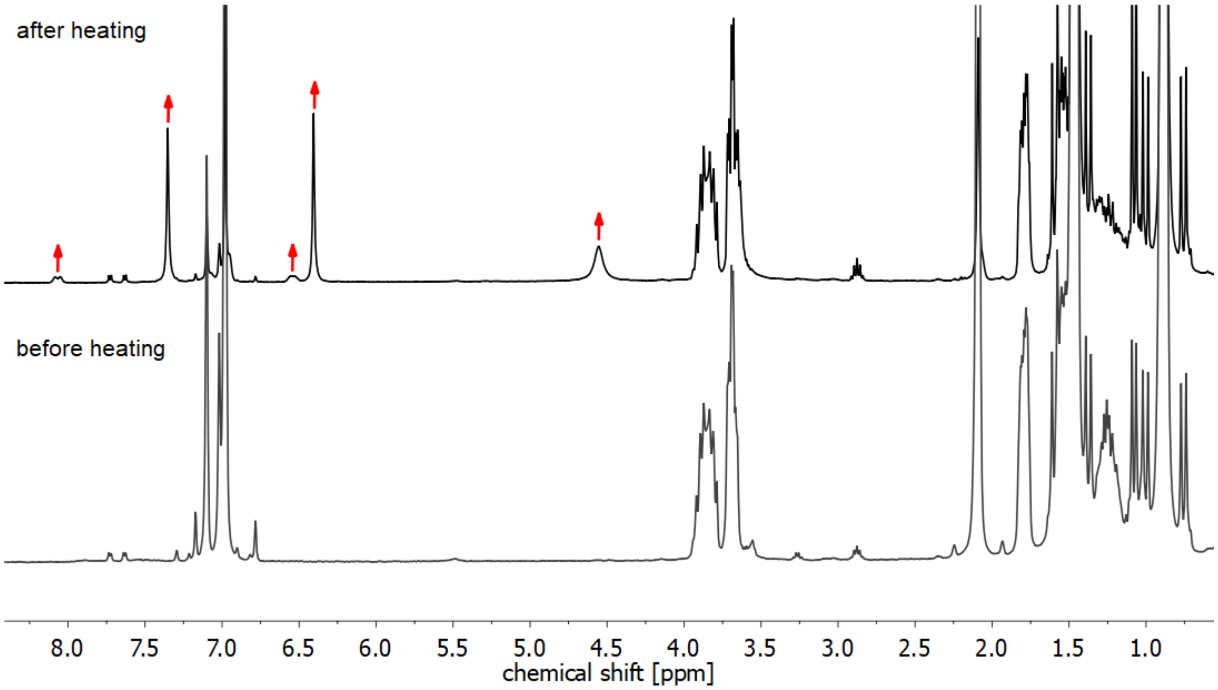

Figure S20. ^{1}H NMR spectra of **4** in toluene-d$_8$ before (bottom) and after (top) heating to 95 °C for 24 h in the absence of light. The red arrows mark the signals of dinuclear **3**.

2.1.5 [W(NCO)(CO)$_2$(PNP)] (**5**)

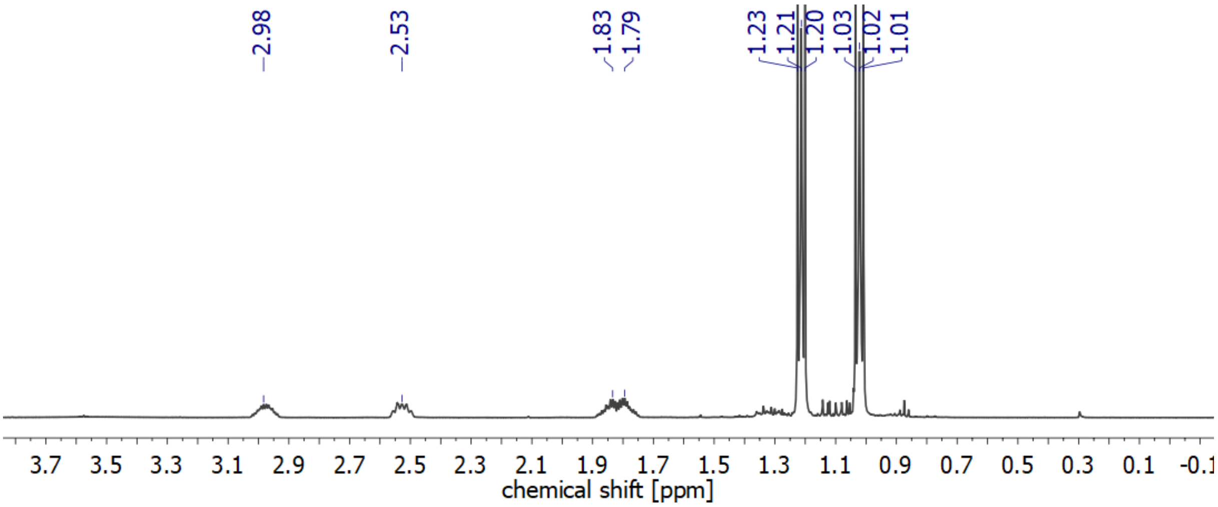

Figure S21. ^{1}H NMR spectrum of **5** in C_6D_6 at room temperature.

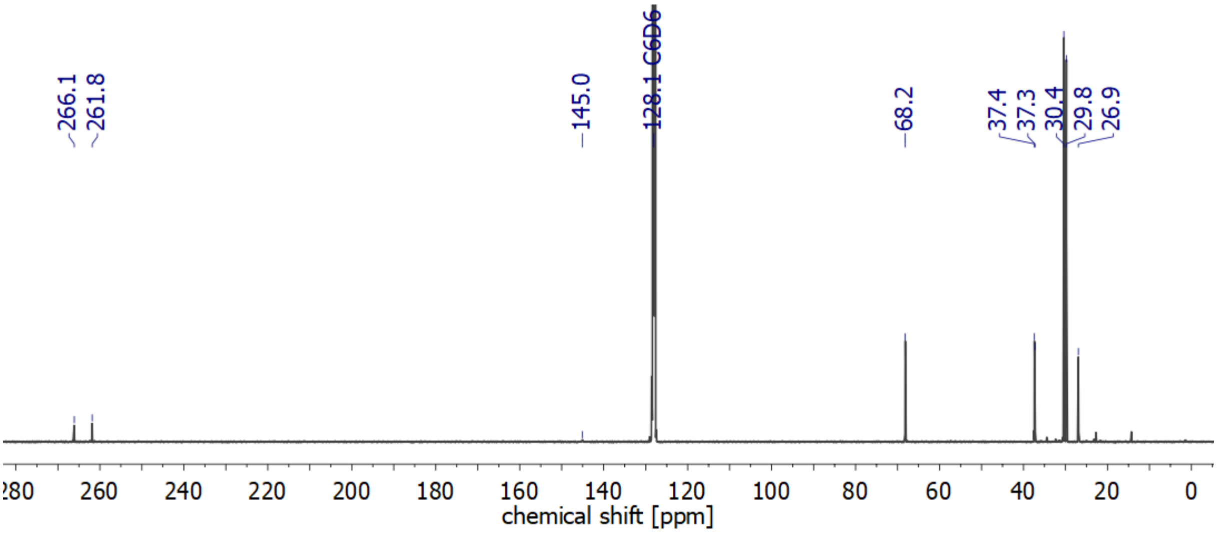

Figure S22. $^{13}C\{^{1}H\}$ NMR spectrum of **5** in C_6D_6 at room temperature.

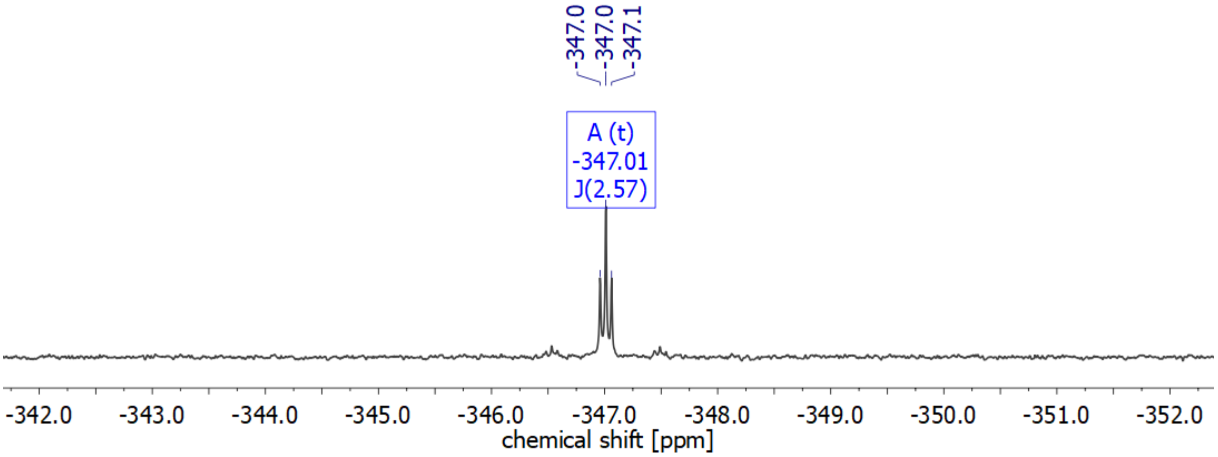

Figure S23. $^{15}N\{^{1}H\}$ NMR spectrum of ^{15}N-**5** in C_6D_6 at room temperature.

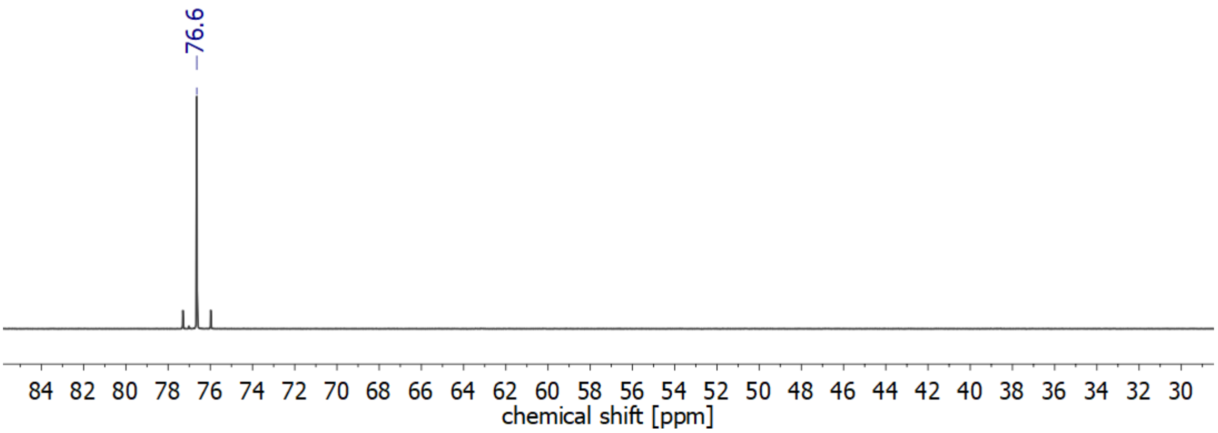

Figure S24. $^{31}P\{^1H\}$ NMR spectrum of **5** in C_6D_6 at room temperature.

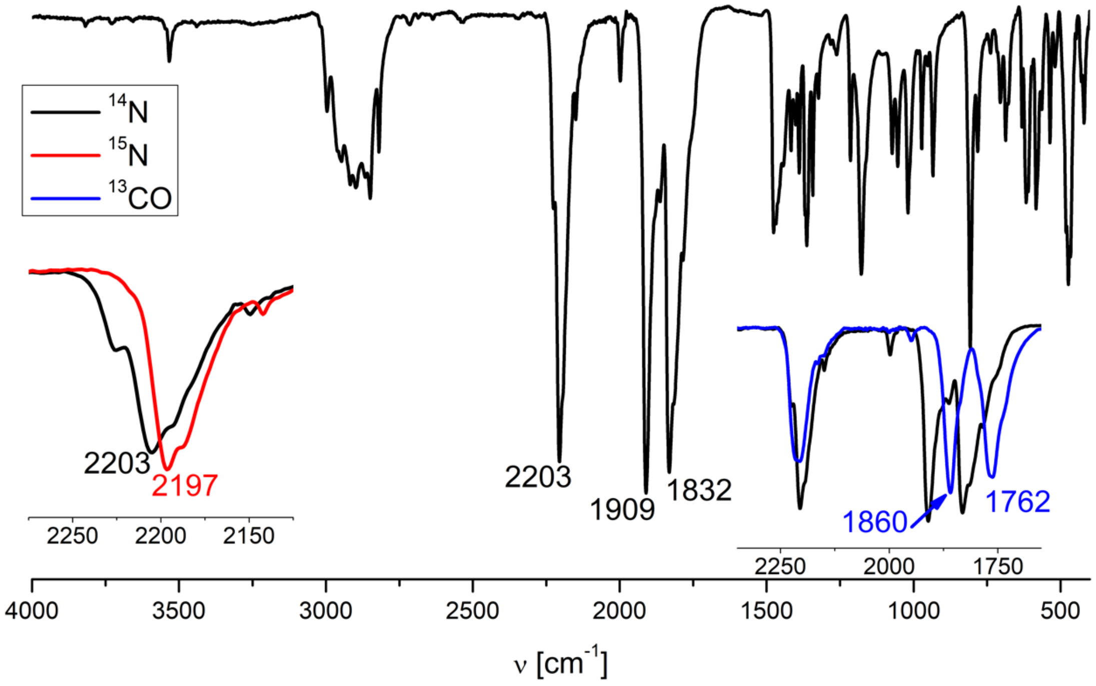

Figure S25. ATR-IR spectra of solid **5** (black), ^{15}N-**5** (red) and ^{13}CO-**5** (blue).

2.1.6 [W(NCO)(^{13}CO)$_2$(PNP)] (^{13}CO-**5**)

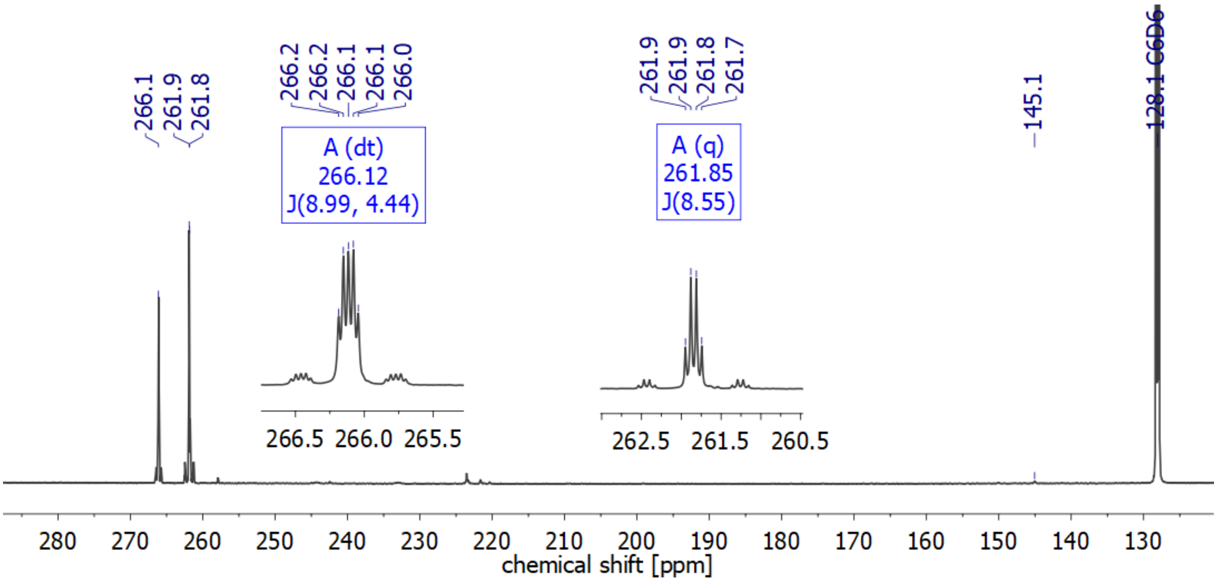

Figure S26. $^{13}C\{^1H\}$ NMR spectrum of ^{13}CO-**5** in C_6D_6 at room temperature.

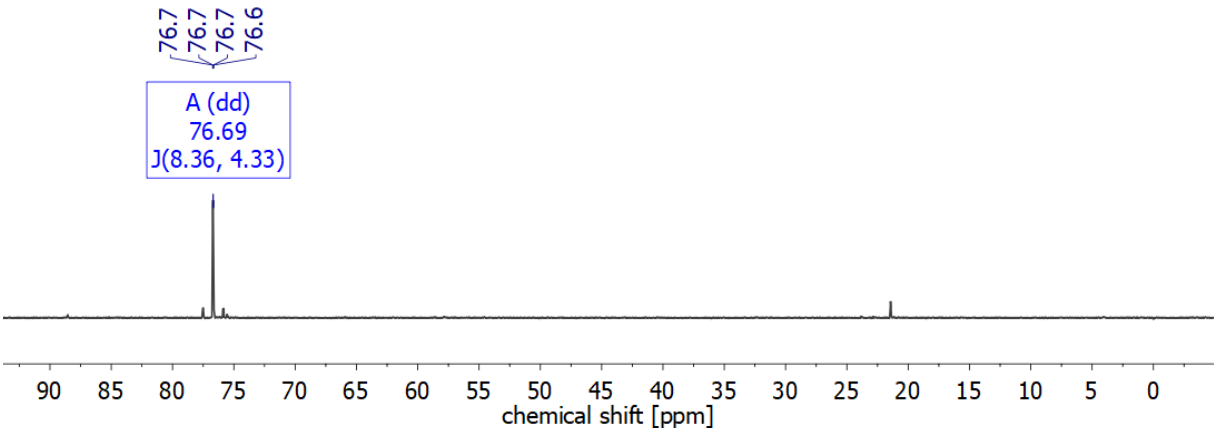

Figure S27. $^{31}P\{^1H\}$ NMR spectrum of ^{13}CO-**5** in C_6D_6 at room temperature.

2.1.7 [W(NCO)(CN-^{t}Bu)$_2$(PNP)] (**6a**)

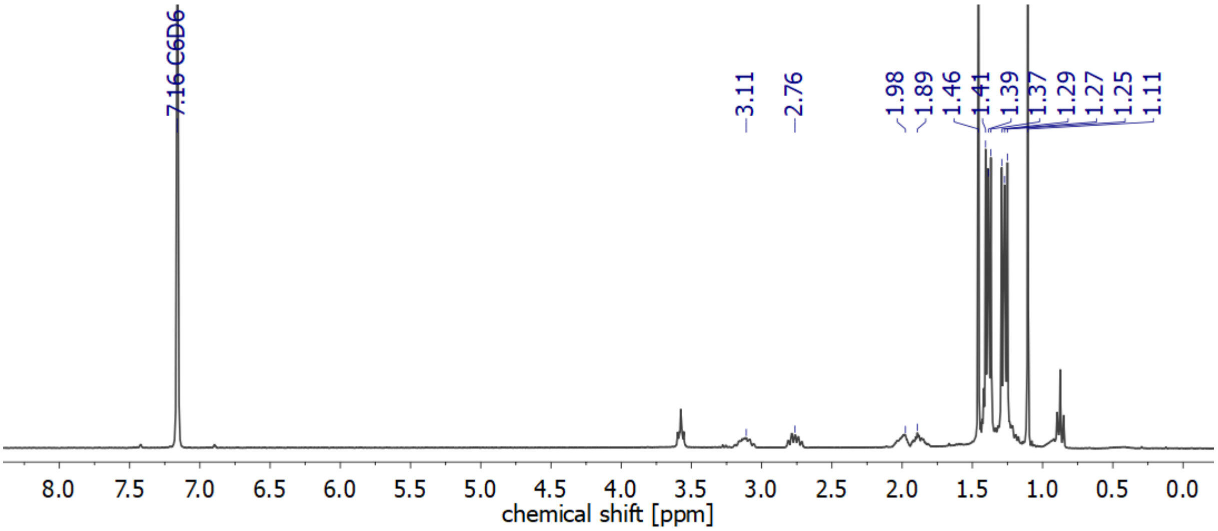

Figure S28. ^{1}H NMR spectrum of **6a** in C_6D_6 at room temperature.

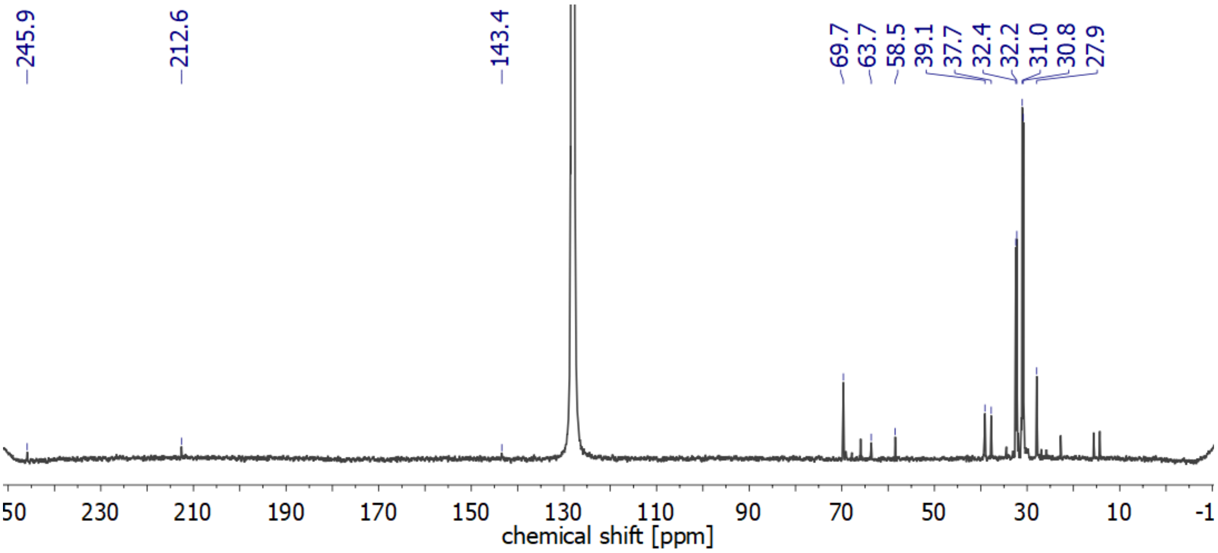

Figure S29. $^{13}C\{^{1}H\}$ NMR spectrum of **6a** in C_6D_6 at room temperature.

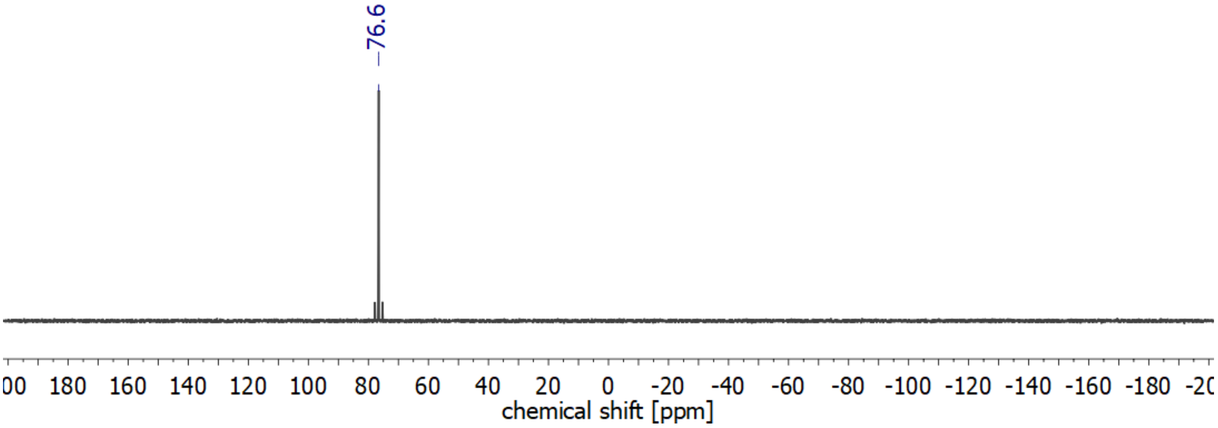

Figure S30. $^{31}P\{^{1}H\}$ NMR spectrum of **6a** in C_6D_6 at room temperature.

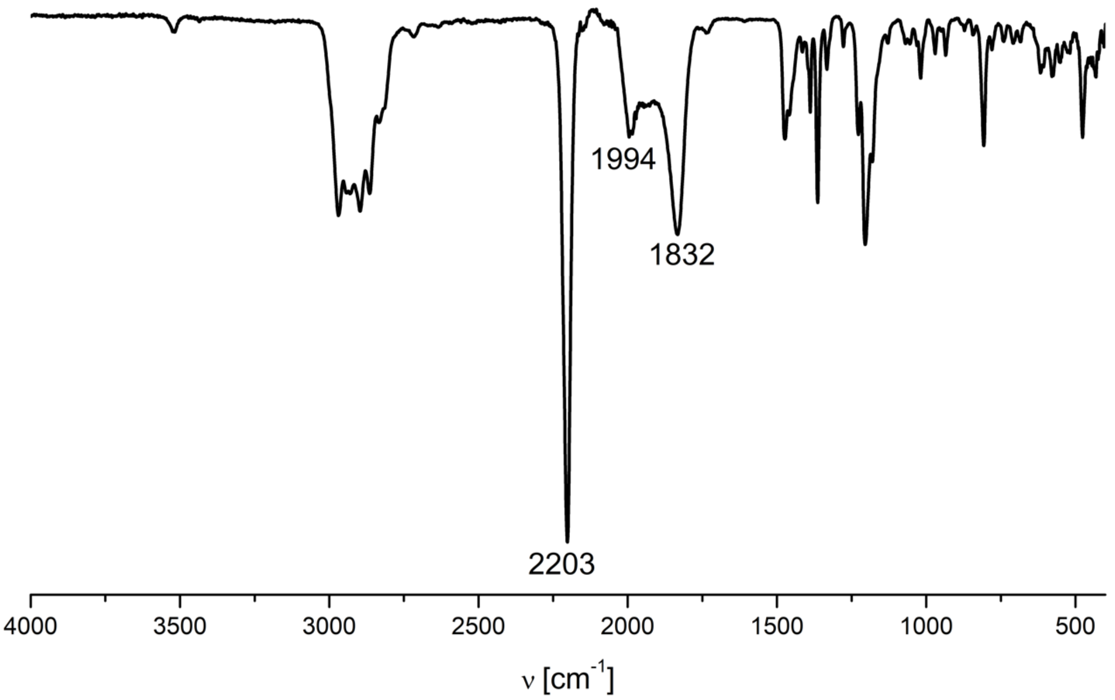

Figure S31. ATR-IR spectrum of solid **6a** at room temperature.

2.1.8 [W(NCO)(CNC$_6$H$_4$OMe)$_2$(PNP)] (**6b**)

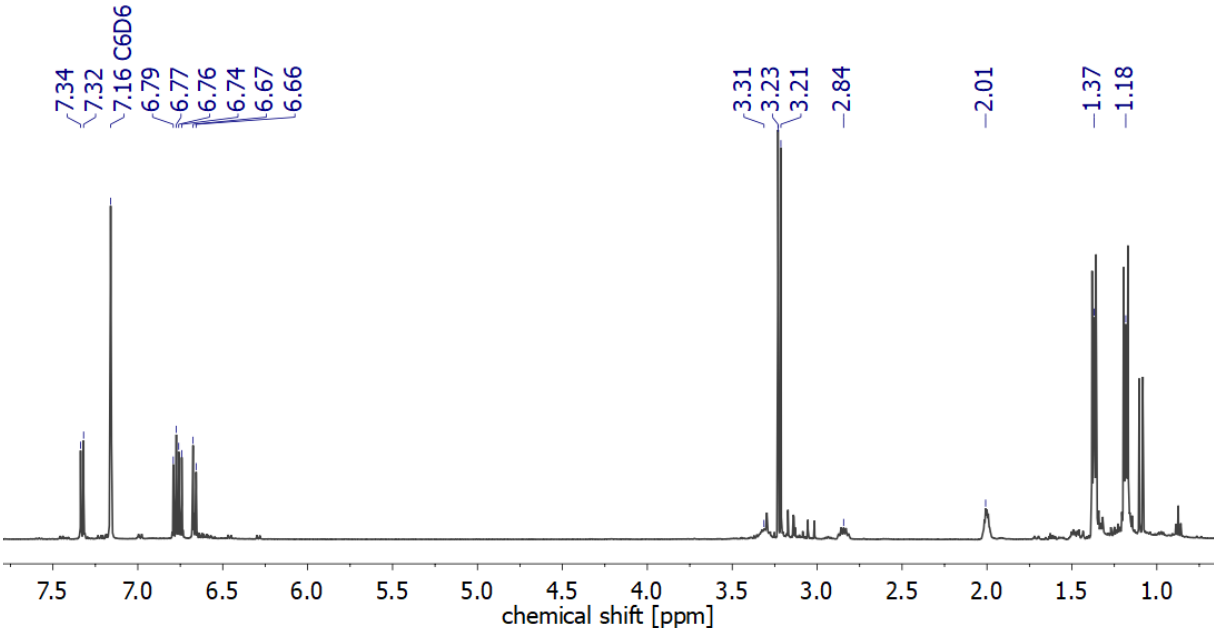

Figure S32. ^{1}H NMR spectrum of **6b** in C_6D_6 at room temperature.

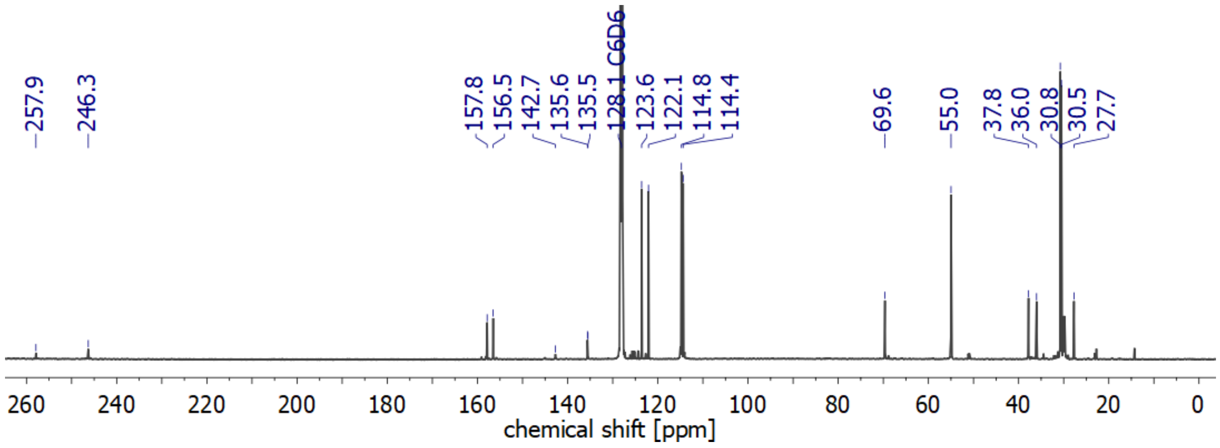

Figure S33. $^{13}C\{^{1}H\}$ NMR spectrum of **6b** in C_6D_6 at room temperature.

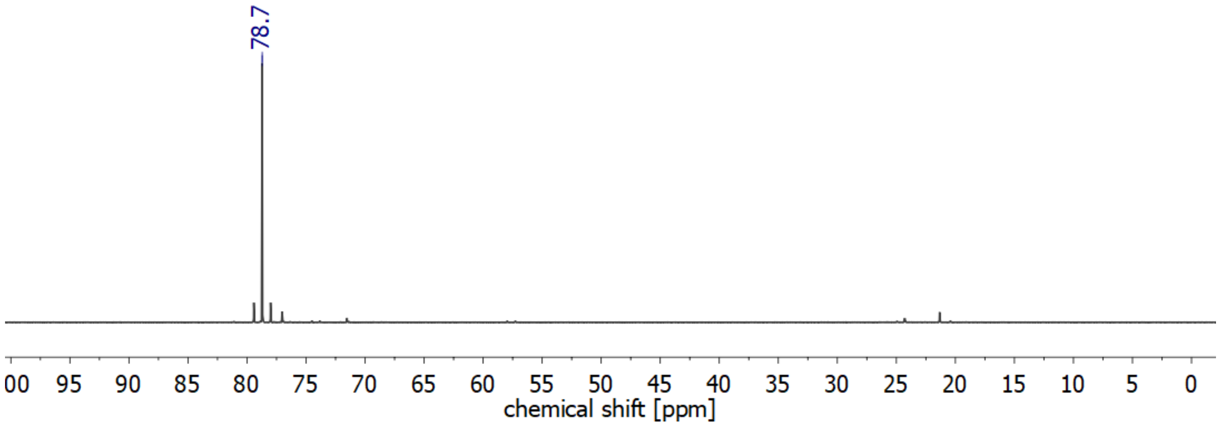

Figure S34. $^{31}P\{^{1}H\}$ NMR spectrum of **6b** in C_6D_6.

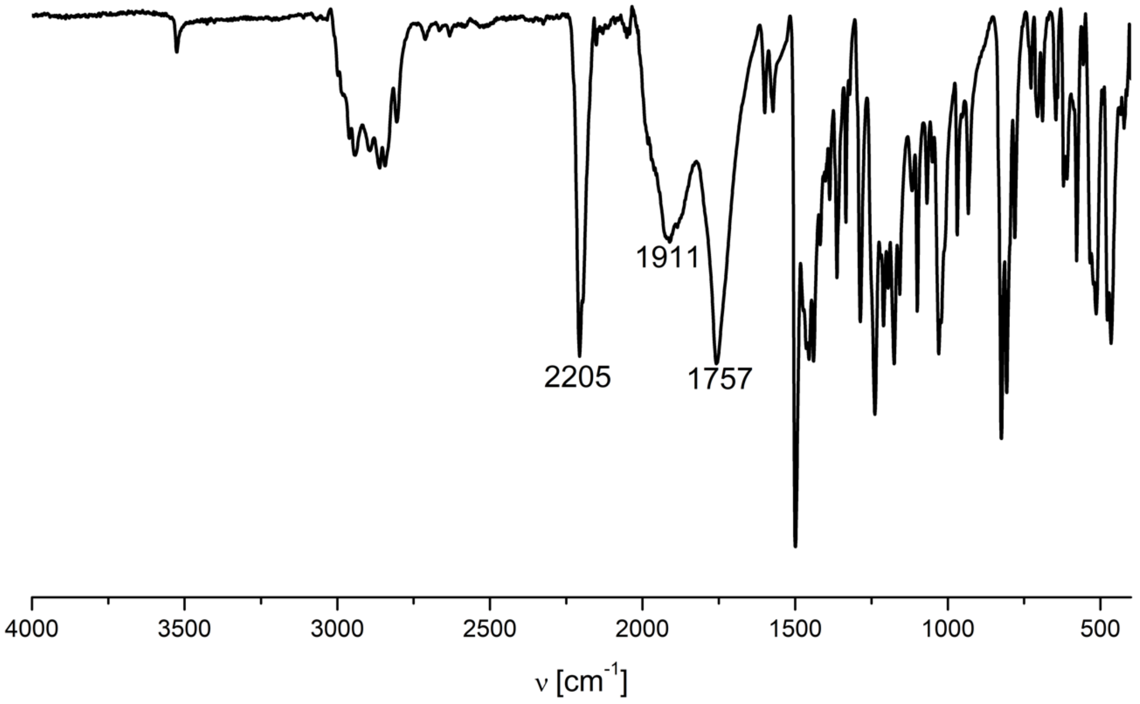

Figure S35. ATR-IR spectrum of solid **6b** at room temperature.

2.1.9 Na[W(CO)$_2$(PNP)] (**7**)

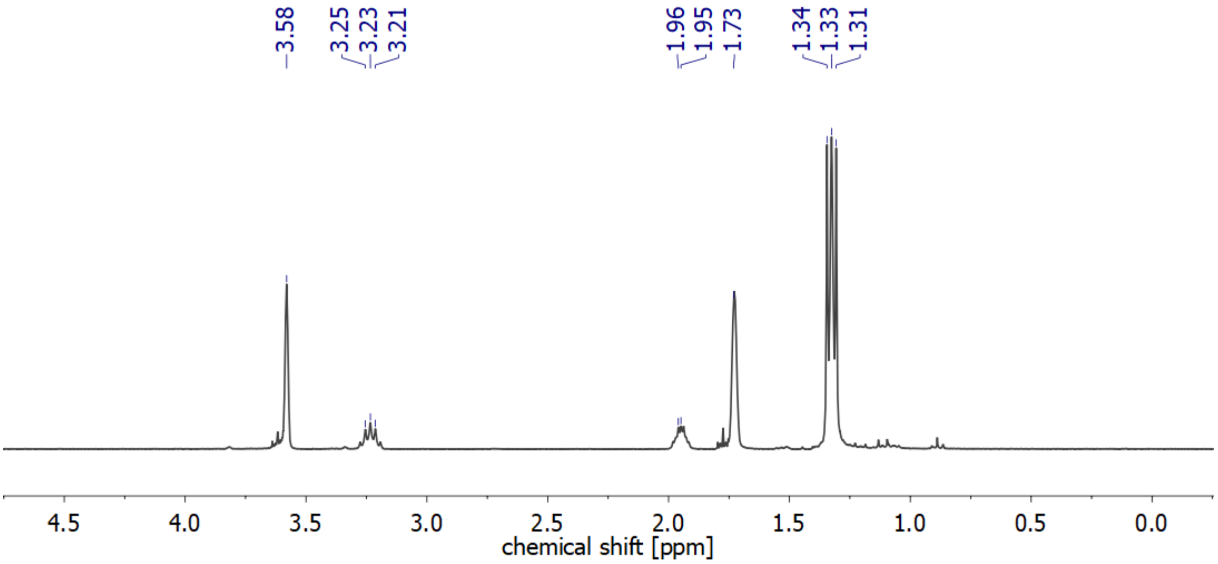

Figure S36. ^{1}H NMR spectrum of **7** in THF-d_8 at room temperature.

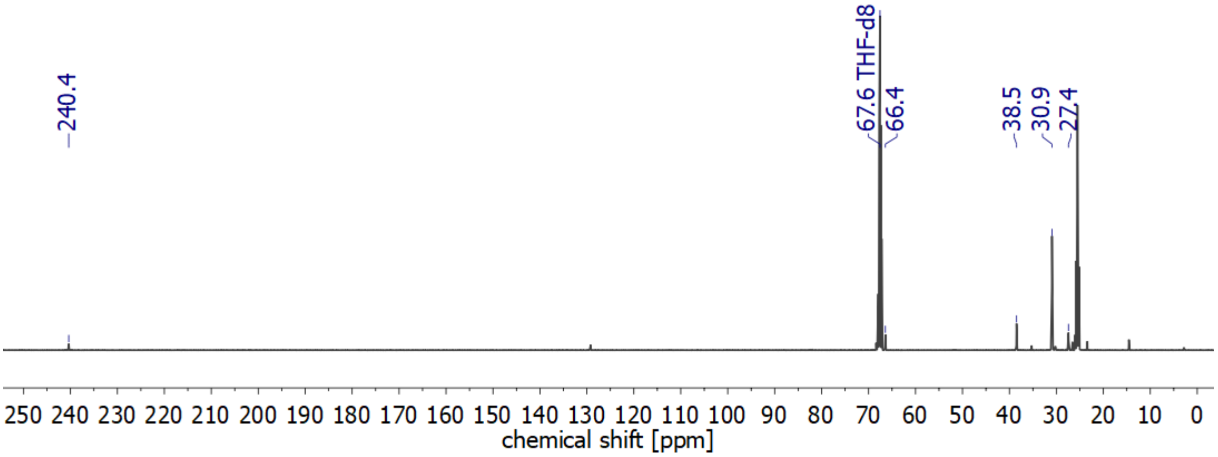

Figure S37. ^{13}C{^{1}H} NMR spectrum of **7** THF-d_8 at room temperature.

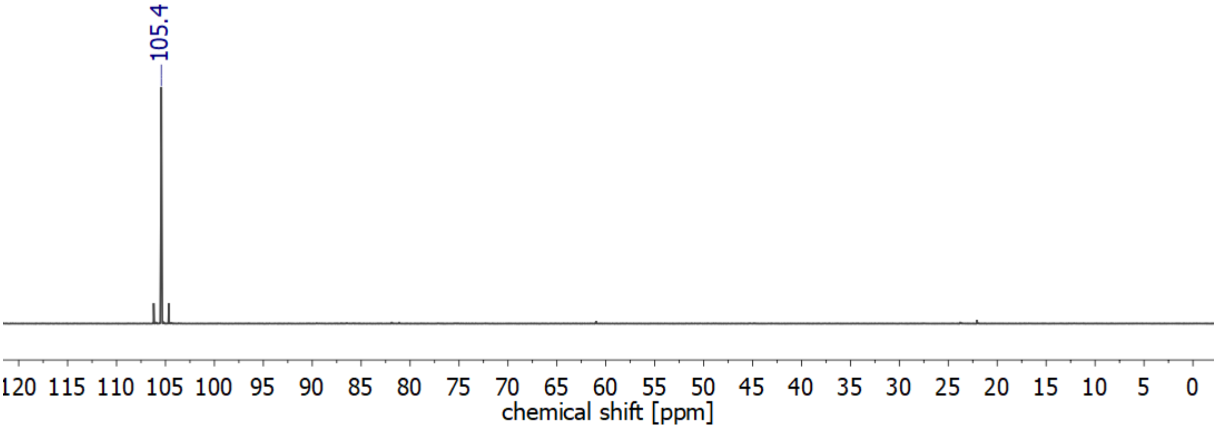

Figure S38. ^{13}C{^{1}H} NMR spectrum of **7** THF-d_8 at room temperature.

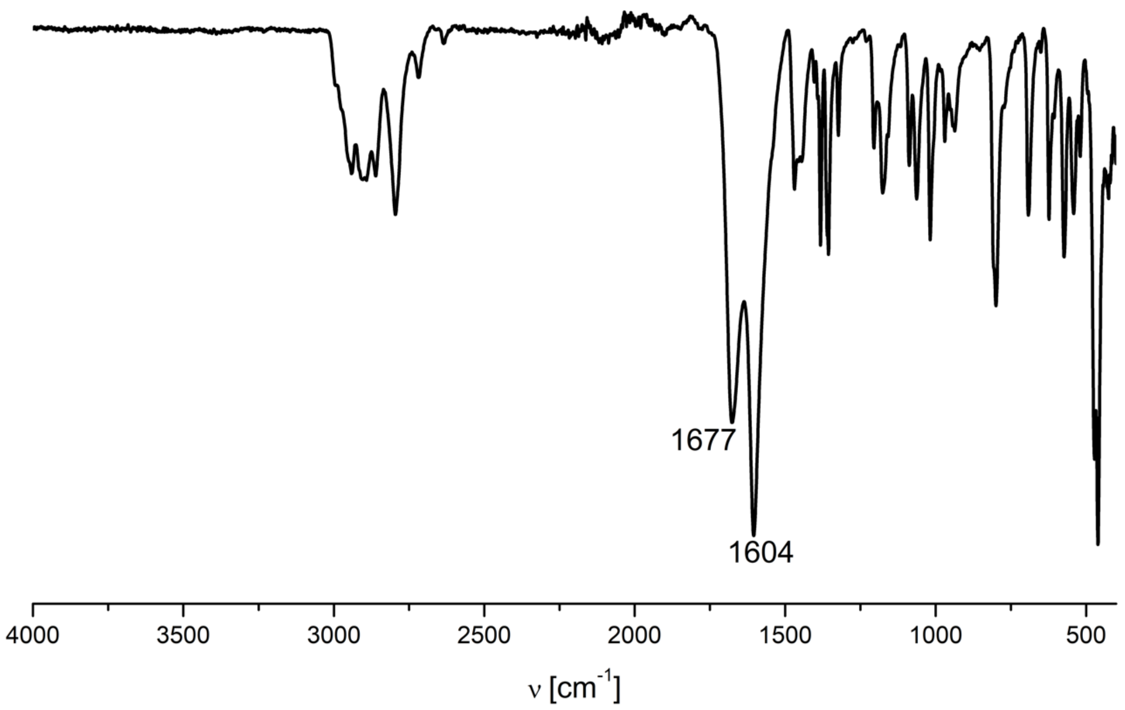

Figure S39. ATR-IR spectrum of solid **7** at room temperature.

2.1.10 [WCl(CO)$_2$(PNP)] (**8**)

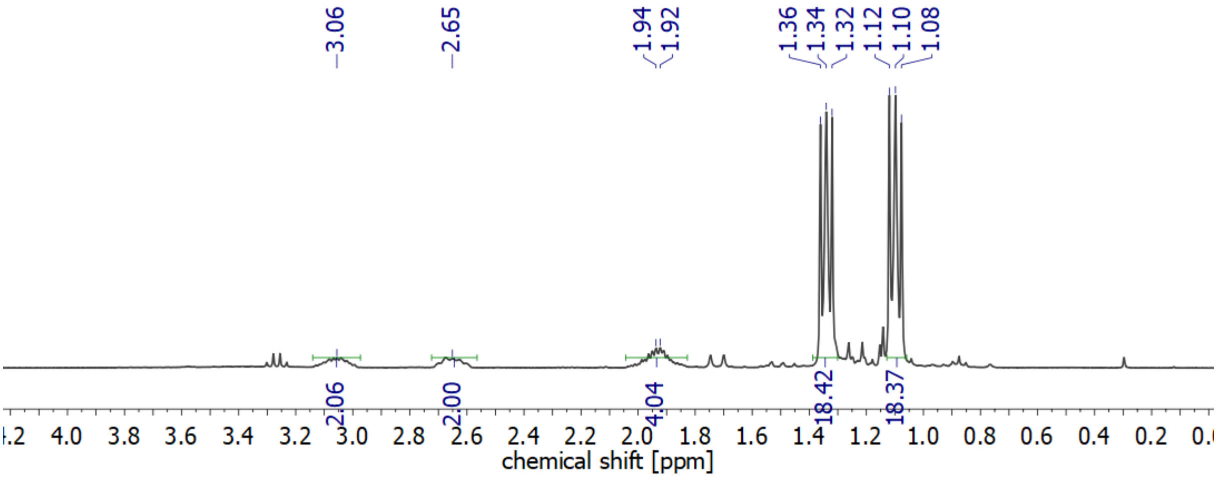

Figure S40. ^{1}H NMR spectrum of **8** in C_6D_6 at room temperature.

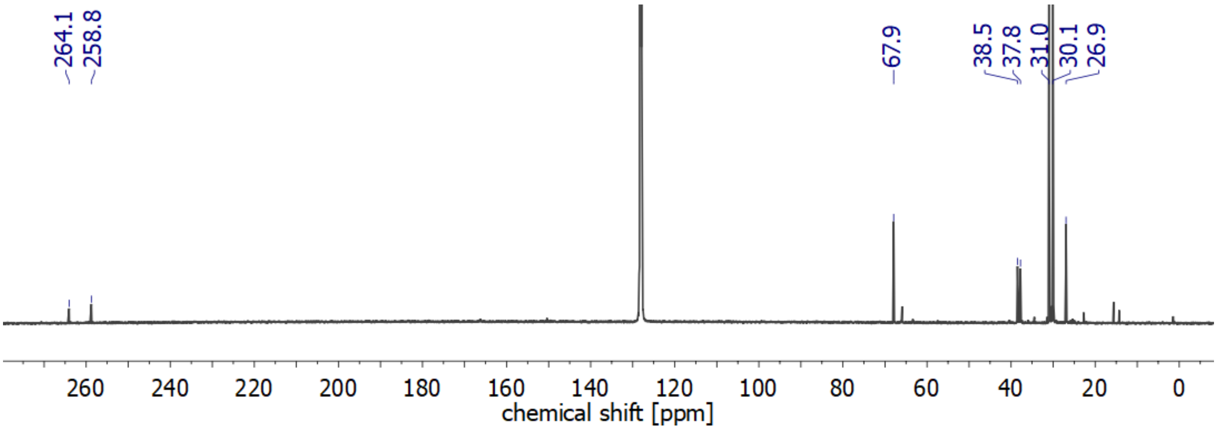

Figure S41. $^{13}C\{^{1}H\}$ NMR spectrum of **8** in C_6D_6 at room temperature.

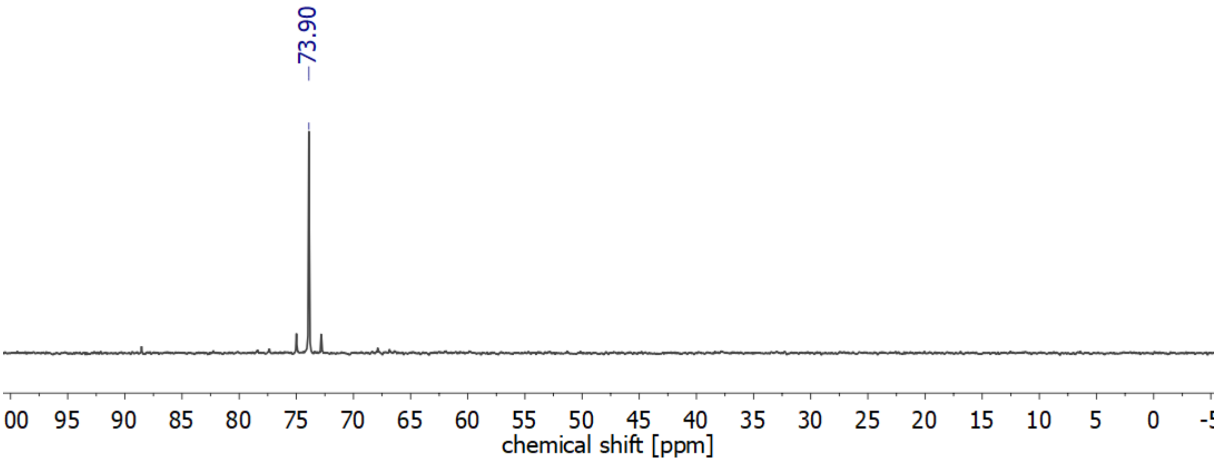

Figure S42. $^{31}P\{^{1}H\}$ NMR spectrum of **8** in C_6D_6 at room temperature.

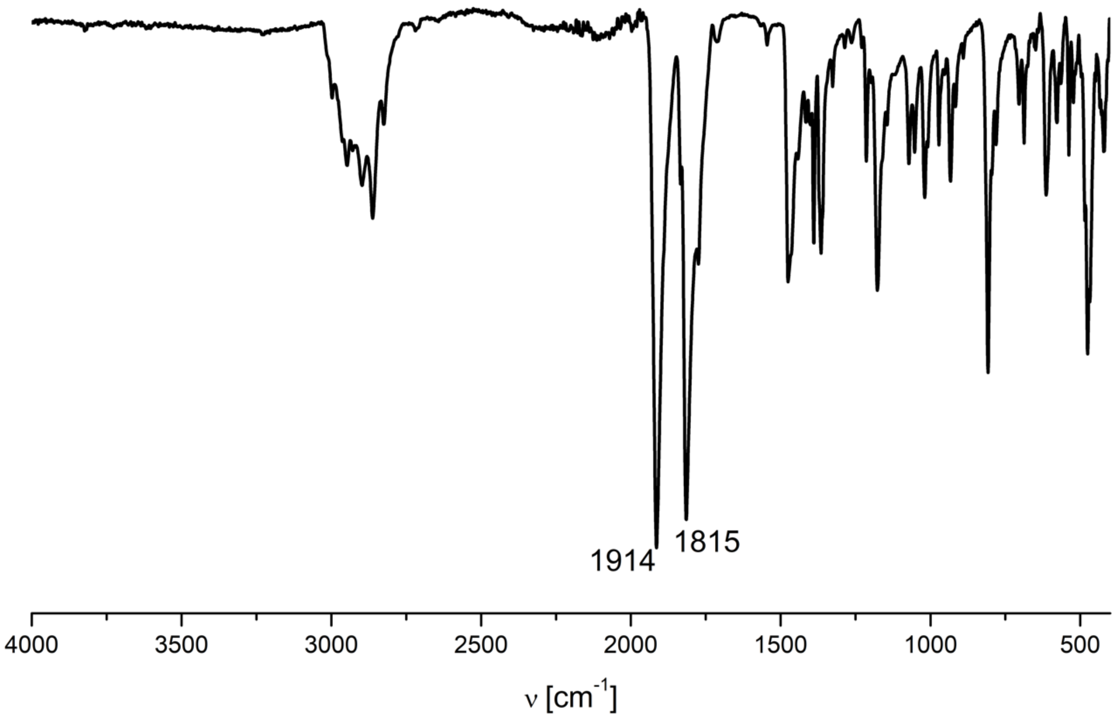

Figure S43. ATR-IR spectrum of solid **8** at room temperature.

2.2. Quantum Yield Determination

2.2.1 Quantum Yield Determination at r.t.

The quantum yield for the photolytic splitting of **3** to **4** was determined at 427 nm (LED) at r.t. The photon flux of the lamp was determined prior to the experiment using a THORLABS S120VC, 200-1100 nm photodiode, which was placed in 20 cm distance to the lamp and photolyzed with 25 % power output:

$$I = 6.5977 \cdot 10^{-7} \pm 1.3 \cdot 10^{-12} \text{ mol cm}^{-2} \text{ min}^{-1}$$

For the quantum yield determination, a solution of complex **3** in THF (2 mL, $5.1 \cdot 10^{-5}$ mol L^{-1}) placed at the same distance from the LED and photolyzed at the same output power for 30 min. The concentration of **3** was monitored every 2 min by UV/vis spectroscopy, following the decay of the band at λ = 512 nm. The quantum yield was determined by

$$\Phi = \frac{\Delta n_{t_x - t_{x-1}}}{n_{Photons,\,2\,min} \cdot (1 - 10^{-A_{427\,\text{nm}}})} \qquad 2.2$$

$\Delta n_{tx\text{-}tx\text{-}1}$ is the amount of **3** that decayed between two measurements (2 min), $n_{\text{Photons, 2 min}}$ is the amount of photons that reached the sample ($2.6391 \cdot 10^{-6}$ mol, calculated by $n = I \cdot t \cdot a$) and $A_{427\text{nm}}$ is the absorbance of the solution at 427 nm at the beginning of each photolysis step, giving a quantum yield of 0.37 ± 0.03 %.

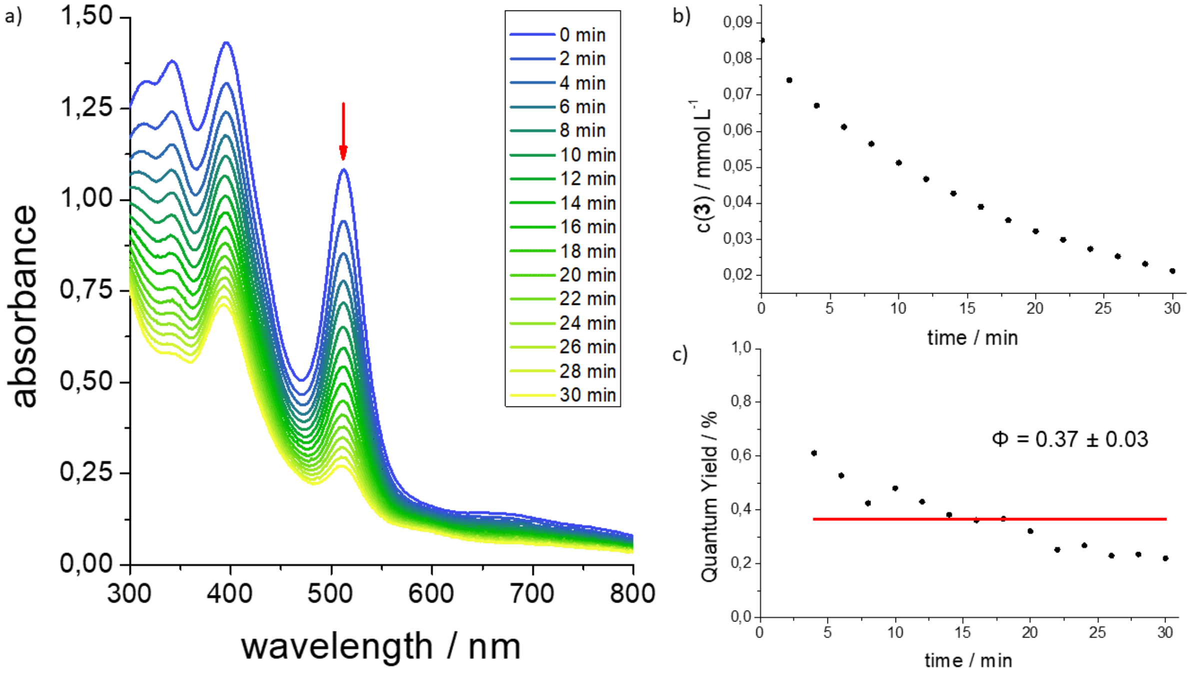

Figure S44. Quantum yield determination of the photodissociation of **3** to **4** with a 427 nm LED lamp. a) UV/vis spectra recorded during photolysis. b) Concentration profile of **3** during the reaction. c) Quantum yields at each irradiation interval.

2.2.1 Quantum Yield Determination at variable temperatures

For quantum yield determination at different temperatures (-80 , -40 and 25 °C) the solution of **3** (THF, 2 mL, 2.98·10^{-5} mol L^{-1}) was placed in a cooled acetone bath. Photolysis for 12 min in the setup described above was monitored by UV/vis spectroscopy every 2 min, following the decay of the band at λ = 512 nm. The quantum yield was determined using eqn. 2.2 assuming the same photon flux (2.6391 · 10^{-6} mol / 2 min).

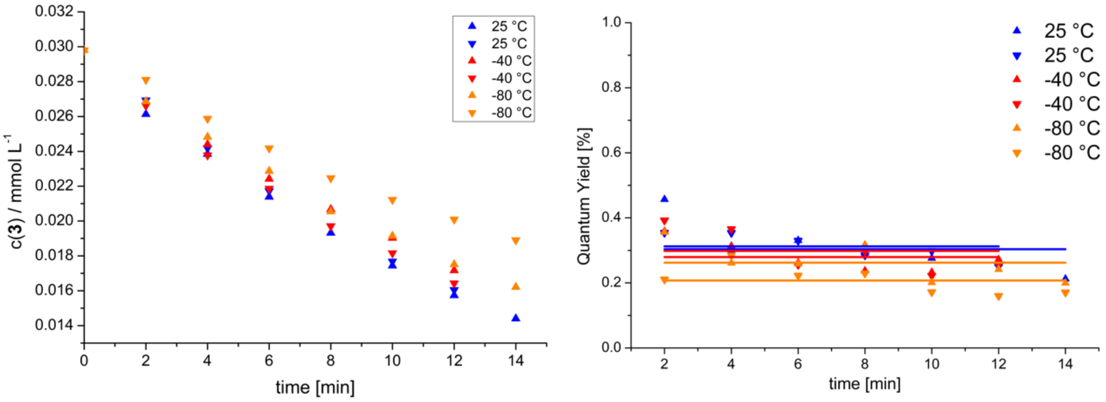

Figure S45. *Left:* Concentration *vs* time plot for the photolysis of **3** at different temperatures. *Right*: Quantum yields for each photolysis interval at different temperatures.

Table S2 Quantum yields at different temperatures.

T [K]	Φ_{427nm} [%]
298	0.30±0.03
298	0.31±0.02
233	0.28±0.02
233	0.30±0.03
193	0.26±0.03
193	0.21±0.02

2.3 Transient Spectroscopy

2.3.1 Transient UV/Vis spectra

The transient UV/Vis-pump-probe setup described in ref. 2 was used, updated with a laser system based on a commercial Ti:sapphire oscillator/regenerative amplifier (Solstice Ace, Spectra Physics) operating at 800 nm producing 35 fs pulses with a mean output power of ~5 W at a 1 kHz repetition rate.

After passing a translational stage (M-415.DG, Physik Instrumente) for producing optical delays of up to ~1.1 ns a small portion (about 3 µJ) was focused into a 4 mm CaF_2 crystal generating a white light continuum. A semi-transparent mirror split the light into a reference and a probe pulse. The latter was superimposed with the pump pulse at a fused silica cuvette (optical path length: 2 mm) containing the sample solution (0.12-0.17 mM in THF) stirred by a magnetic stirrer. Reference and probe continua were measured by two spectrometers each equipped with a 256-element linear diode array detector.

Pump pulses in the UV/Vis region were generated using an automated optical parametric amplifier and subsequent frequency mixer (TOPAS Prime + and NirUVis, Light Conversion) which was pumped by the main part of the Solstice Ace output. The plane of polarization of the resulting pump pulses was adjusted to 54.7° relative to the probe pulses and were attenuated to energies of 0.3 - 1.0 µJ. A synchronized chopper blocking every second pulse was used to measure absorption changes induced by the pump. Transient spectra were corrected for wavelength dependent temporal shifts due to group velocity dispersion.

The transient UV/Vis-pump probe spectra shown in **Figure 7** and **Figure S46** to **Figure S48** show clear evidence for the formation of vibrationally hot molecules being formed after excitation and subsequent ultrafast internal conversion (<70 fs) to the ground state. The excess energy in the molecule leads to a broadening of the electronic absorption bands compared to the ambient spectrum which results in bleaching at the absorption centers and enhanced absorption at the wings. Vibrational relaxation is analyzed by plotting the time dependent integral over the absolute value of the difference spectra $\int|\Delta A(E)|$ (inserts in **Figure 7** and **Figure S46** to **Figure S48**). Independent on excitation wavelength the relaxation appears to occur on two different timescales with average time constants of τ_1 = 1.4 ps and τ_2 = 9.6 ps. Whereas the τ_2 = 9.6 ps time constant is consistent with vibrational cooling to reach thermal equilibrium with the surrounding solvent bath, the fast component τ_1 = 1.4 ps might indicate that directly after internal conversion the excess energy preferentially distributes over those regions and vibrational modes of the molecule which couple to the electronic transition being excited. This is consistent with the observation that the amplitudes of hot band absorptions at 560 and 455 nm depend on whether the 510 nm or the 400 nm band is excited. When the former is addressed (pump wavelength 475 and 530 nm, **Figure 7** and **Figure S46**) the hot band at 560 nm appears stronger than at 455 nm. Conversely, when the 400 nm band is excited (pump wavelength 380 and 330 nm, **Figure S47** and **Figure S48**) the amplitude of the 560 nm hot band is smaller than at 455 nm. The timescale of τ_1 = 1.4 ps is consistent with intramolecular vibrational energy redistribution.

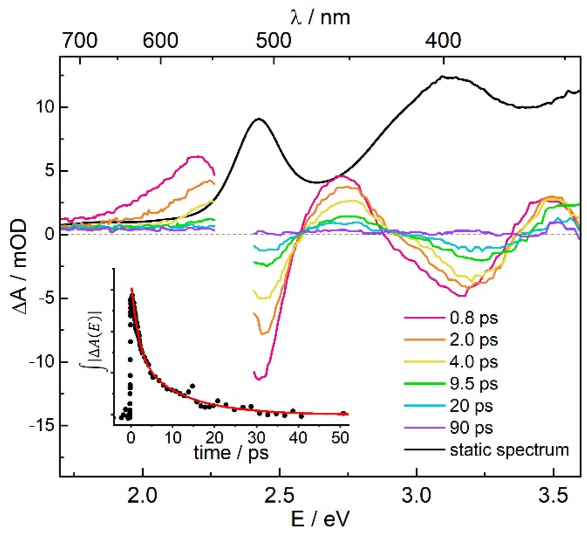

Figure S46. Transient UV/Vis difference spectra of **3** in THF at selected pump-probe delays as indicated (pump wavelength: 530 nm; black line: scaled linear absorption spectrum). Insert: time-dependence of the integrated absolute absorption changes (red line: bi-exponential fit yielding time constants of (1.8±0.3) ps and (11.1±1.5) ps).

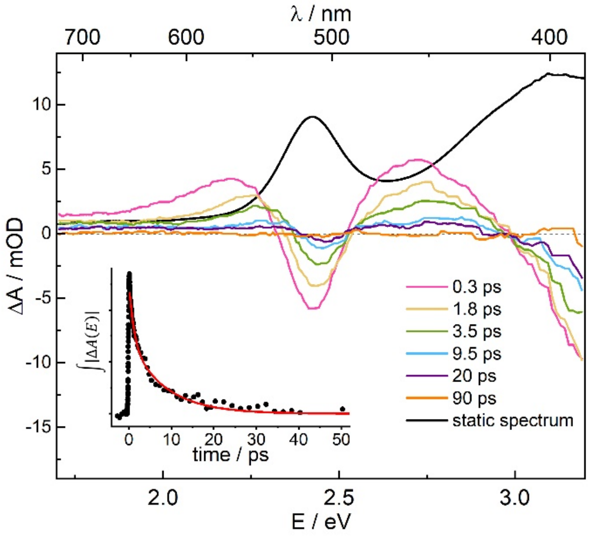

Figure S47. Transient UV/Vis difference spectra of **3** in THF at selected pump-probe delays as indicated (pump wavelength: 380 nm; black line: scaled linear absorption spectrum). Insert: time-dependence of the integrated absolute absorption changes (red line: bi-exponential fit yielding time constants of (1.1±0.4) ps and (8.3±1.6) ps).

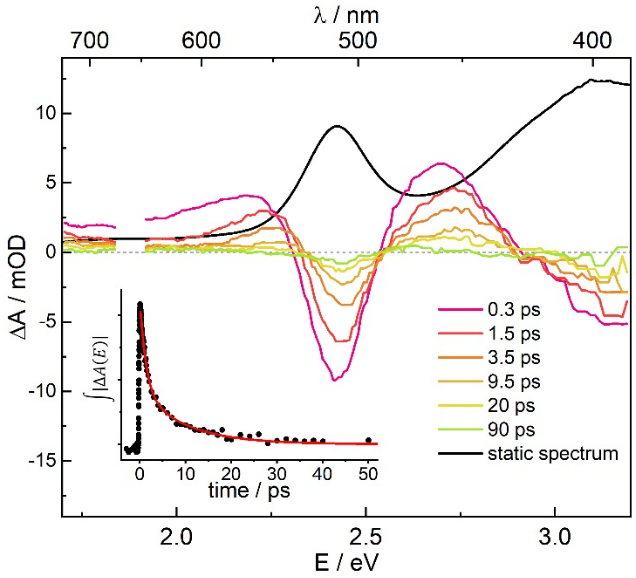

Figure S48. Transient UV/Vis difference spectra of **3** in THF at selected pump-probe delays as indicated (pump wavelength: 330 nm; black line: scaled linear absorption spectrum). Insert: time-dependence of the integrated absolute absorption changes (red line: bi-exponential fit yielding time constants of (1.3±0.4) ps and (9.9±1.4) ps).

2.3.2 Transient IR spectra

UV-pump–IR-probe experiments were performed with a laser setup described elsewhere.[3] Briefly, 40 % of the 750 mW output of a 1 kHz Ti:sapphire oscillator/regenerative amplifier producing 150 fs pulses at 800 nm was used to generate pump pulses at 400 nm by second harmonic generation. Tunable mid-infrared probe pulses were produced by difference frequency mixing of signal and idler pulses from an optical parametric amplifier [4] pumped by the remaining 60% regenerative amplifier energy. The IR light was split into a probe and a reference beam and both focused into the sample cell. The probe pulse was overlaid with the pump pulse, their relative plane of polarization was set to 54.7°. After passing the sample, reference and probe IR light were dispersed in a polychromator (Chromex 250is, Bruker Optics) and their spectra recorded with a liquid nitrogen cooled 2x32 element mercury cadmium telluride detector (IR-6416 system MCT-32/2-10, Infrared Systems Development). Experiments were performed in a hermetically sealed stainless steel cell with two 1mm thick CaF_2 windows and an optical path length of 0.8 mm containing stirred 2mM solutions of **3** in THF.

The transient IR spectra show immediate bleaching of the CO stretching modes superimposed by broadened absorption bands which we attribute to the vibrationally hot electronic ground state (**Figure S49** *left*). Similar to the time resolved spectra in the UV/Vis these features disappear within several tens of picoseconds. Bi-exponential fits (**Figure S49** *right*) yield time constants of (1.9±0.5) ps and (16±3) ps for the relaxation consistent with IVR and subsequent vibrational cooling of the vibrationally hot molecules over the surrounding solvent bath.

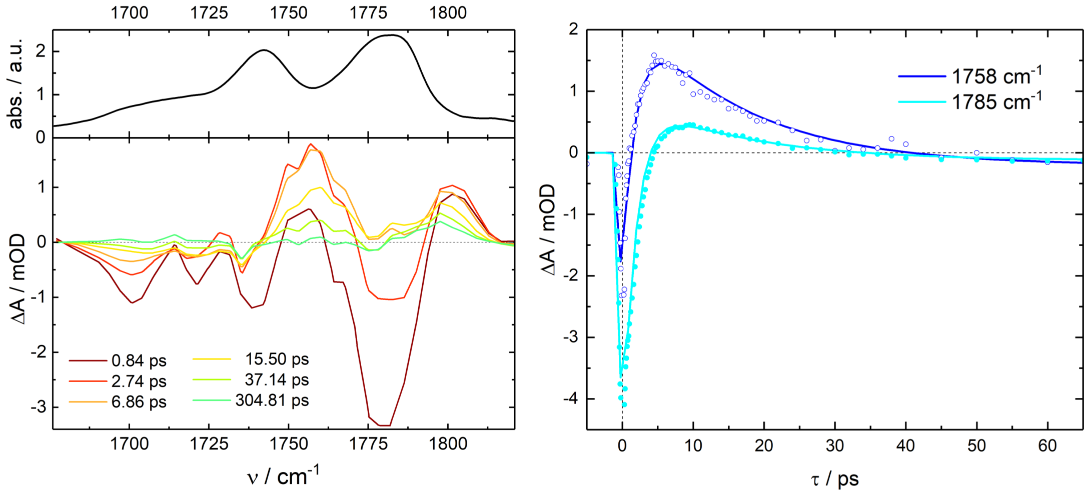

Figure S49. *Left:* transient IR difference spectra of **3** in THF at selected pump-probe delays (pump wavelength: 400 nm, upper panel: linear absorption spectrum of **3** in THF). *Right:* time traces for selected wave numbers (solid lines: bi-exponential fits yielding time constants of (1.9±0.5) ps and (16±3) ps).

3. Crystallographic Details

Suitable single crystals for X-ray structure determination were selected from the mother liquor under an inert gas atmosphere and transferred in protective perfluoro polyether oil on a microscope slide. The selected and mounted crystals were transferred to the cold gas stream on the diffractometer. The diffraction data were obtained at 100 K on a Bruker D8 three-circle diffractometer, equipped with a PHOTON 100 CMOS detector and an INCOATEC microfocus source with Quazar mirror optics (Mo-Kα radiation, λ= 0.71073 Å).

The data obtained were integrated with SAINT and a semi-empirical absorption correction from equivalents with SADABS was applied. The structure was solved and refined using the Bruker SHELX 2014 software package.[5,6,7,8] All non-hydrogen atoms were refined with anisotropic displacement parameters. All C-H hydrogen atoms were refined isotropically on calculated positions by using a riding model with their U_{iso} values constrained to 1.5 U_{eq} of their pivot atoms for terminal sp^3 carbon atoms and 1.2 times for all other atoms.

3.1 Crystal Structure of **2**

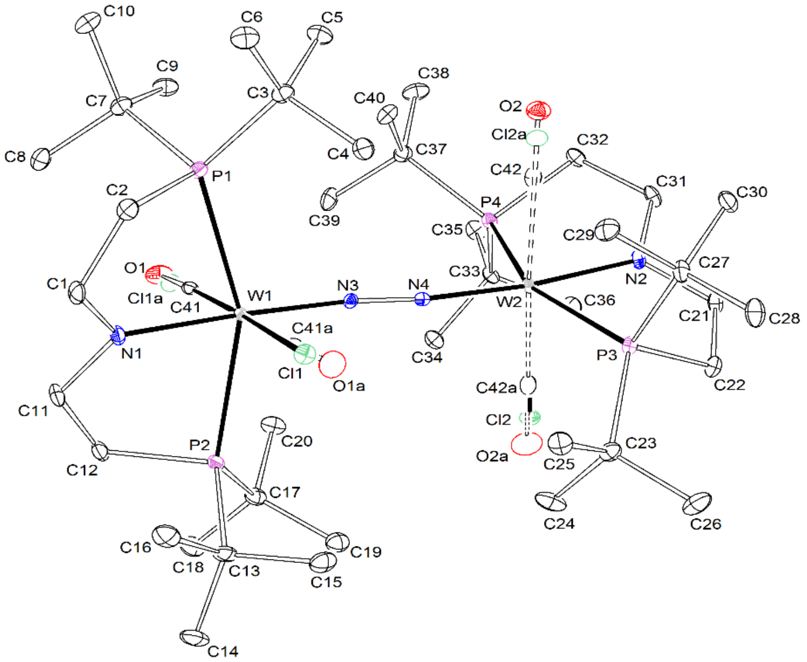

Figure S50. Thermal ellipsoid plot of **2** with the anisotropic displacement parameters drawn at the 50% probability level. The asymmetric unit contains one disordered complex molecule. The disordered complex molecule was refined with population of 0.845(5) and 0.724(8) on their main domains using some restraints and constraints (SADI, EADP). The structure was refined as an inversion twin using the twin law -100 0-10 00-1 (BASF: 0.307(4)). Reflection 0 2 1 was removed from the refinement using OMIT commands.

Table S3 Crystal data and structure refinement for 2.

Identification code	CCDC-2069378	
Empirical formula	$C_{42}H_{88}Cl_2N_4O_2P_4W_2$	
Formula weight	1243.64	
Temperature	101(2) K	
Wavelength	0.71073 Å	
Crystal system	Orthorhombic	
Space group	$P2_12_12_1$	
Unit cell dimensions	a = 12.2138(5) Å	α = 90°
	b = 18.6597(8) Å	β = 90°
	c = 23.1169(9) Å	γ = 90°
Volume	5268.5(4) Å3	
Z	4	
Density (calculated)	1.568 Mg/m^3	
Absorption coefficient	4.622 mm^{-1}	
F(000)	2504	
Crystal size	0.284 x 0.165 x 0.134 mm^3	
Crystal shape and color	Block, dark blue	
Theta range for data collection	2.179 to 28.357°	
Index ranges	-16<=h<=16, -24<=k<=24, -30<=l<=30	
Reflections collected	163801	
Independent reflections	13100 [R(int) = 0.0659]	
Completeness to theta = 25.242°	99.9 %	
Refinement method	Full-matrix least-squares on F2	
Data / restraints / parameters	13100 / 24 / 568	
Goodness-of-fit on F2	1.056	
Final R indices [I>2sigma(I)]	R1 = 0.0187, wR2 = 0.0312	
R indices (all data)	R1 = 0.0233, wR2 = 0.0321	
Largest diff. peak and hole	0.495 and -0.783 $eÅ^{-3}$	

Table S4 Bond lengths [Å] and angles [°] for 2.

N(1)-C(11)	1.460(5)	C(22)-P(3)	1.842(3)
N(1)-C(1)	1.463(5)	C(23)-C(24)	1.526(6)
N(1)-W(1)	2.032(3)	C(23)-C(26)	1.541(5)
N(2)-C(31)	1.457(5)	C(23)-C(25)	1.542(5)
N(2)-C(21)	1.471(5)	C(23)-P(3)	1.906(4)
N(2)-W(2)	2.023(3)	C(27)-C(28)	1.532(5)
N(3)-N(4)	1.223(4)	C(27)-C(30)	1.538(5)
N(3)-W(1)	1.870(3)	C(27)-C(29)	1.540(5)
N(4)-W(2)	1.872(3)	C(27)-P(3)	1.888(4)
C(1)-C(2)	1.530(6)	C(31)-C(32)	1.544(5)
C(2)-P(1)	1.844(4)	C(32)-P(4)	1.843(4)
C(3)-C(5)	1.531(5)	C(33)-C(35)	1.538(5)
C(3)-C(6)	1.534(5)	C(33)-C(34)	1.543(5)
C(3)-C(4)	1.541(6)	C(33)-C(36)	1.543(5)
C(3)-P(1)	1.901(4)	C(33)-P(4)	1.899(4)
C(7)-C(10)	1.530(5)	C(37)-C(40)	1.524(5)
C(7)-C(9)	1.544(5)	C(37)-C(39)	1.536(6)
C(7)-C(8)	1.546(6)	C(37)-C(38)	1.541(5)
C(7)-P(1)	1.901(4)	C(37)-P(4)	1.890(4)
C(11)-C(12)	1.532(6)	W(1)-C(41A)	1.884(14)
C(12)-P(2)	1.845(4)	W(1)-C(41)	1.924(8)
C(13)-C(16)	1.535(6)	W(1)-P(2)	2.5230(10)
C(13)-C(14)	1.540(6)	W(1)-P(1)	2.5434(10)
C(13)-C(15)	1.548(6)	W(1)-Cl(1)	2.572(2)
C(13)-P(2)	1.900(4)	W(1)-Cl(1A)	2.630(8)
C(17)-C(20)	1.535(5)	W(2)-C(42A)	1.890(17)
C(17)-C(18)	1.539(6)	W(2)-C(42)	1.950(6)
C(17)-C(19)	1.545(6)	W(2)-P(4)	2.5279(9)
C(17)-P(2)	1.903(4)	W(2)-Cl(2)	2.5407(18)
C(21)-C(22)	1.534(5)	W(2)-P(3)	2.5508(9)

W(2)-Cl(2A)	2.662(10)	C(16)-C(13)-C(15)	108.2(3)
O(1)-C(41)	1.155(10)	C(14)-C(13)-C(15)	109.4(3)
O(1A)-C(41A)	1.151(16)	C(16)-C(13)-P(2)	108.4(3)
O(2)-C(42)	1.153(8)	C(14)-C(13)-P(2)	114.0(3)
O(2A)-C(42A)	1.144(18)	C(15)-C(13)-P(2)	109.3(3)
		C(20)-C(17)-C(18)	107.2(3)
C(11)-N(1)-C(1)	110.9(3)	C(20)-C(17)-C(19)	106.0(3)
C(11)-N(1)-W(1)	124.5(3)	C(18)-C(17)-C(19)	109.8(3)
C(1)-N(1)-W(1)	124.6(3)	C(20)-C(17)-P(2)	110.1(3)
C(31)-N(2)-C(21)	109.8(3)	C(18)-C(17)-P(2)	112.1(3)
C(31)-N(2)-W(2)	125.4(2)	C(19)-C(17)-P(2)	111.4(3)
C(21)-N(2)-W(2)	124.8(2)	N(2)-C(21)-C(22)	114.6(3)
N(4)-N(3)-W(1)	174.2(3)	C(21)-C(22)-P(3)	112.3(2)
N(3)-N(4)-W(2)	174.2(3)	C(24)-C(23)-C(26)	107.9(4)
N(1)-C(1)-C(2)	113.8(3)	C(24)-C(23)-C(25)	106.0(3)
C(1)-C(2)-P(1)	111.9(3)	C(26)-C(23)-C(25)	110.1(3)
C(5)-C(3)-C(6)	110.0(3)	C(24)-C(23)-P(3)	108.8(3)
C(5)-C(3)-C(4)	107.0(3)	C(26)-C(23)-P(3)	112.5(3)
C(6)-C(3)-C(4)	107.7(3)	C(25)-C(23)-P(3)	111.2(3)
C(5)-C(3)-P(1)	110.8(3)	C(28)-C(27)-C(30)	106.1(3)
C(6)-C(3)-P(1)	113.3(3)	C(28)-C(27)-C(29)	109.3(3)
C(4)-C(3)-P(1)	107.8(3)	C(30)-C(27)-C(29)	109.1(3)
C(10)-C(7)-C(9)	108.9(3)	C(28)-C(27)-P(3)	114.5(3)
C(10)-C(7)-C(8)	106.8(3)	C(30)-C(27)-P(3)	108.2(3)
C(9)-C(7)-C(8)	108.2(3)	C(29)-C(27)-P(3)	109.6(3)
C(10)-C(7)-P(1)	114.0(3)	N(2)-C(31)-C(32)	113.8(3)
C(9)-C(7)-P(1)	110.2(3)	C(31)-C(32)-P(4)	111.5(3)
C(8)-C(7)-P(1)	108.5(3)	C(35)-C(33)-C(34)	108.5(3)
N(1)-C(11)-C(12)	113.8(3)	C(35)-C(33)-C(36)	106.8(3)
C(11)-C(12)-P(2)	111.6(3)	C(34)-C(33)-C(36)	108.6(3)
C(16)-C(13)-C(14)	107.4(4)	C(35)-C(33)-P(4)	115.4(3)

C(34)-C(33)-P(4)	111.2(3)	P(1)-W(1)-Cl(1A)	92.3(2)
C(36)-C(33)-P(4)	106.0(3)	N(4)-W(2)-C(42A)	93.9(9)
C(40)-C(37)-C(39)	107.1(3)	N(4)-W(2)-C(42)	88.85(16)
C(40)-C(37)-C(38)	107.4(3)	N(4)-W(2)-N(2)	174.40(13)
C(39)-C(37)-C(38)	110.3(3)	C(42A)-W(2)-N(2)	91.4(9)
C(40)-C(37)-P(4)	109.9(3)	C(42)-W(2)-N(2)	85.83(16)
C(39)-C(37)-P(4)	109.3(3)	N(4)-W(2)-P(4)	99.79(9)
C(38)-C(37)-P(4)	112.7(3)	C(42A)-W(2)-P(4)	91.9(9)
N(3)-W(1)-C(41A)	87.0(5)	C(42)-W(2)-P(4)	86.44(14)
N(3)-W(1)-C(41)	92.9(2)	N(2)-W(2)-P(4)	78.13(9)
N(3)-W(1)-N(1)	177.38(13)	N(4)-W(2)-Cl(2)	94.79(11)
C(41A)-W(1)-N(1)	91.6(5)	C(42)-W(2)-Cl(2)	176.09(15)
C(41)-W(1)-N(1)	88.4(2)	N(2)-W(2)-Cl(2)	90.48(11)
N(3)-W(1)-P(2)	99.65(9)	P(4)-W(2)-Cl(2)	91.52(6)
C(41A)-W(1)-P(2)	92.2(4)	N(4)-W(2)-P(3)	102.98(9)
C(41)-W(1)-P(2)	86.4(2)	C(42A)-W(2)-P(3)	87.9(9)
N(1)-W(1)-P(2)	78.16(10)	C(42)-W(2)-P(3)	92.72(14)
N(3)-W(1)-P(1)	105.11(9)	N(2)-W(2)-P(3)	79.07(9)
C(41A)-W(1)-P(1)	90.2(5)	P(4)-W(2)-P(3)	157.19(3)
C(41)-W(1)-P(1)	91.2(2)	Cl(2)-W(2)-P(3)	87.87(6)
N(1)-W(1)-P(1)	77.12(10)	N(4)-W(2)-Cl(2A)	88.9(3)
P(2)-W(1)-P(1)	155.22(3)	C(42A)-W(2)-Cl(2A)	177.2(10)
N(3)-W(1)-Cl(1)	90.63(10)	N(2)-W(2)-Cl(2A)	85.9(3)
C(41)-W(1)-Cl(1)	176.5(2)	P(4)-W(2)-Cl(2A)	88.0(3)
N(1)-W(1)-Cl(1)	88.07(10)	P(3)-W(2)-Cl(2A)	91.2(3)
P(2)-W(1)-Cl(1)	93.01(6)	C(2)-P(1)-C(7)	104.81(18)
P(1)-W(1)-Cl(1)	87.89(6)	C(2)-P(1)-C(3)	104.25(18)
N(3)-W(1)-Cl(1A)	93.7(2)	C(7)-P(1)-C(3)	108.63(18)
C(41A)-W(1)-Cl(1A)	177.1(5)	C(2)-P(1)-W(1)	95.27(13)
N(1)-W(1)-Cl(1A)	87.6(2)	C(7)-P(1)-W(1)	117.89(13)
P(2)-W(1)-Cl(1A)	84.9(2)	C(3)-P(1)-W(1)	122.28(12)

C(12)-P(2)-C(13)	103.63(19)	C(23)-P(3)-W(2)	123.09(13)
C(12)-P(2)-C(17)	104.72(19)	C(32)-P(4)-C(37)	104.05(17)
C(13)-P(2)-C(17)	108.76(18)	C(32)-P(4)-C(33)	105.57(17)
C(12)-P(2)-W(1)	94.99(13)	C(37)-P(4)-C(33)	107.97(17)
C(13)-P(2)-W(1)	120.53(14)	C(32)-P(4)-W(2)	95.74(13)
C(17)-P(2)-W(1)	120.15(13)	C(37)-P(4)-W(2)	119.46(12)
C(22)-P(3)-C(27)	105.83(18)	C(33)-P(4)-W(2)	120.71(12)
C(22)-P(3)-C(23)	104.27(17)	O(1)-C(41)-W(1)	177.6(10)
C(27)-P(3)-C(23)	107.45(18)	O(1A)-C(41A)-W(1)	177.5(16)
C(22)-P(3)-W(2)	95.21(12)	O(2)-C(42)-W(2)	177.4(4)
C(27)-P(3)-W(2)	117.70(13)	O(2A)-C(42A)-W(2)	179(4)

Symmetry transformations used to generate equivalent atoms:

Table S5 Torsion angles [°] for 2.

C(11)-N(1)-C(1)-C(2)	-164.7(3)	C(11)-C(12)-P(2)-C(17)	-161.0(3)
W(1)-N(1)-C(1)-C(2)	17.0(4)	C(11)-C(12)-P(2)-W(1)	-38.0(3)
N(1)-C(1)-C(2)-P(1)	20.0(4)	C(16)-C(13)-P(2)-C(12)	-52.8(3)
C(1)-N(1)-C(11)-C(12)	-167.2(3)	C(14)-C(13)-P(2)-C(12)	66.7(4)
W(1)-N(1)-C(11)-C(12)	11.1(5)	C(15)-C(13)-P(2)-C(12)	-170.5(3)
N(1)-C(11)-C(12)-P(2)	24.3(4)	C(16)-C(13)-P(2)-C(17)	-163.8(3)
C(31)-N(2)-C(21)-C(22)	170.2(3)	C(14)-C(13)-P(2)-C(17)	-44.3(4)
W(2)-N(2)-C(21)-C(22)	-7.4(5)	C(15)-C(13)-P(2)-C(17)	78.5(3)
N(2)-C(21)-C(22)-P(3)	-24.6(4)	C(16)-C(13)-P(2)-W(1)	51.5(3)
C(21)-N(2)-C(31)-C(32)	171.1(3)	C(14)-C(13)-P(2)-W(1)	171.0(3)
W(2)-N(2)-C(31)-C(32)	-11.2(5)	C(15)-C(13)-P(2)-W(1)	-66.2(3)
N(2)-C(31)-C(32)-P(4)	-22.8(4)	C(21)-C(22)-P(3)-C(27)	-85.3(3)
C(1)-C(2)-P(1)-C(7)	84.4(3)	C(21)-C(22)-P(3)-C(23)	161.5(3)
C(1)-C(2)-P(1)-C(3)	-161.5(3)	C(21)-C(22)-P(3)-W(2)	35.4(3)
C(1)-C(2)-P(1)-W(1)	-36.3(3)	C(28)-C(27)-P(3)-C(22)	-70.2(3)
C(11)-C(12)-P(2)-C(13)	85.1(3)	C(30)-C(27)-P(3)-C(22)	47.8(3)

C(29)-C(27)-P(3)-C(22)	166.6(3)	C(39)-C(37)-P(4)-C(33)	52.7(3)
C(28)-C(27)-P(3)-C(23)	40.7(3)	C(38)-C(37)-P(4)-C(33)	-70.2(3)
C(30)-C(27)-P(3)-C(23)	158.7(3)	C(40)-C(37)-P(4)-W(2)	26.8(3)
C(29)-C(27)-P(3)-C(23)	-82.4(3)	C(39)-C(37)-P(4)-W(2)	-90.4(3)
C(28)-C(27)-P(3)-W(2)	-175.0(2)	C(38)-C(37)-P(4)-W(2)	146.6(2)
C(30)-C(27)-P(3)-W(2)	-57.0(3)	C(35)-C(33)-P(4)-C(32)	-67.8(3)
C(29)-C(27)-P(3)-W(2)	61.8(3)	C(34)-C(33)-P(4)-C(32)	168.1(3)
C(31)-C(32)-P(4)-C(37)	158.4(3)	C(36)-C(33)-P(4)-C(32)	50.2(3)
C(31)-C(32)-P(4)-C(33)	-88.0(3)	C(35)-C(33)-P(4)-C(37)	43.1(3)
C(31)-C(32)-P(4)-W(2)	36.1(3)	C(34)-C(33)-P(4)-C(37)	-81.1(3)
C(40)-C(37)-P(4)-C(32)	-78.2(3)	C(36)-C(33)-P(4)-C(37)	161.0(2)
C(39)-C(37)-P(4)-C(32)	164.5(3)	C(35)-C(33)-P(4)-W(2)	-174.4(2)
C(38)-C(37)-P(4)-C(32)	41.6(3)	C(34)-C(33)-P(4)-W(2)	61.5(3)
C(40)-C(37)-P(4)-C(33)	170.0(3)	C(36)-C(33)-P(4)-W(2)	-56.4(3)

Symmetry transformations used to generate equivalent atoms:

3.2 Crystal Structure of **3**

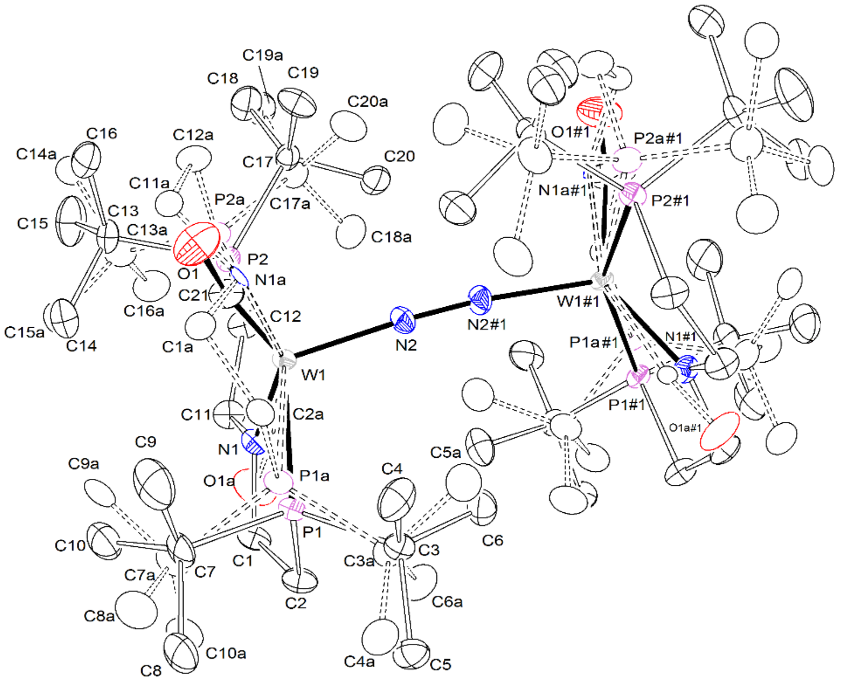

Figure S51 Thermal ellipsoid plot of **3** with the anisotropic displacement parameters drawn at the 25% probability level. The asymmetric unit contains a half disordered complex molecule. The disordered complex molecule was refined with population of 0.799(6) on the main domain using some restraints (SADI, RIGU).

Table S6 Crystal data and structure refinement for 3.

Identification code	CCDC-2069379	
Empirical formula	$C_{42}H_{88}N_4O_2P_4W_2$	
Formula weight	1172.74	
Temperature	100(2) K	
Wavelength	0.71073 Å	
Crystal system	Monoclinic	
Space group	C2/c	
Unit cell dimensions	a = 27.147(3) Å	α = 90°
	b = 9.8359(11) Å	β = 109.232(4)°
	c = 19.638(2) Å	γ = 90°
Volume	4951.0(10) $Å^3$	
Z	4	
Density (calculated)	1.573 Mg/m^3	
Absorption coefficient	4.809 mm^{-1}	
F(000)	2368	

Crystal size	0.219 x 0.169 x 0.070 mm^3
Crystal shape and color	Block, dark blue
Theta range for data collection	2.218 to 25.388°
Index ranges	-29<=h<=32, -11<=k<=11, -23<=l<=23
Reflections collected	33664
Independent reflections	4465 [R(int) = 0.0579]
Completeness to theta = 25.242°	98.0 %
Refinement method	Full-matrix least-squares on F2
Data / restraints / parameters	4465 / 646 / 494
Goodness-of-fit on F2	1.224
Final R indices [I>2sigma(I)]	R1 = 0.0478, wR2 = 0.1012
R indices (all data)	R1 = 0.0555, wR2 = 0.1058
Largest diff. peak and hole	2.492 and -1.568 $eÅ^{-3}$

Table S7 Bond lengths [Å] and angles [°] for 3.

W(1)-N(2)	1.869(7)	O(1)-C(21)	1.190(16)
W(1)-C(21)	1.956(14)	P(2)-C(12)	1.849(12)
W(1)-C(21A)	1.97(2)	P(2)-C(17)	1.892(10)
W(1)-N(1)	2.043(10)	P(2)-C(13)	1.894(10)
W(1)-N(1A)	2.04(2)	C(11)-C(12)	1.503(14)
W(1)-P(1A)	2.398(14)	C(10)-C(7)	1.522(13)
W(1)-P(2)	2.435(4)	C(9)-C(7)	1.523(14)
W(1)-P(1)	2.485(3)	C(8)-C(7)	1.519(13)
W(1)-P(2A)	2.517(17)	C(6)-C(3)	1.509(12)
N(2)-N(2)#1	1.207(14)	C(5)-C(3)	1.525(12)
P(1)-C(2)	1.835(12)	C(4)-C(3)	1.535(12)
P(1)-C(3)	1.889(10)	C(20)-C(17)	1.520(12)
P(1)-C(7)	1.894(10)	C(19)-C(17)	1.526(12)
C(1)-N(1)	1.48(2)	C(18)-C(17)	1.513(12)
C(1)-C(2)	1.513(14)	C(16)-C(13)	1.514(13)
N(1)-C(11)	1.46(2)	C(15)-C(13)	1.523(13)

C(14)-C(13)	1.534(13)
P(1A)-C(2A)	1.85(4)
P(1A)-C(3A)	1.887(16)
P(1A)-C(7A)	1.888(16)
N(1A)-C(11A)	1.46(8)
N(1A)-C(1A)	1.54(8)
C(1A)-C(2A)	1.51(2)
O(1A)-C(21A)	1.21(2)
P(2A)-C(12A)	1.85(5)
P(2A)-C(13A)	1.885(16)
P(2A)-C(17A)	1.888(16)
C(20A)-C(17A)	1.523(17)
C(19A)-C(17A)	1.520(17)
C(18A)-C(17A)	1.524(17)
C(16A)-C(13A)	1.525(17)
C(15A)-C(13A)	1.519(17)
C(14A)-C(13A)	1.523(17)
C(12A)-C(11A)	1.50(2)
C(10A)-C(7A)	1.514(17)
C(9A)-C(7A)	1.516(17)
C(8A)-C(7A)	1.517(17)
C(6A)-C(3A)	1.520(17)
C(5A)-C(3A)	1.518(17)
C(4A)-C(3A)	1.519(17)
N(2)-W(1)-C(21)	103.5(8)
N(2)-W(1)-C(21A)	108(3)
N(2)-W(1)-N(1)	115.6(6)
C(21)-W(1)-N(1)	140.9(9)
N(2)-W(1)-N(1A)	96(2)
C(21A)-W(1)-N(1A)	156(4)
N(2)-W(1)-P(1A)	104.3(4)
C(21A)-W(1)-P(1A)	94(3)
N(1A)-W(1)-P(1A)	85(2)
N(2)-W(1)-P(2)	98.8(2)
C(21)-W(1)-P(2)	93.1(8)
N(1)-W(1)-P(2)	79.3(6)
N(2)-W(1)-P(1)	102.8(2)
C(21)-W(1)-P(1)	94.4(7)
N(1)-W(1)-P(1)	79.7(6)
P(2)-W(1)-P(1)	154.79(13)
N(2)-W(1)-P(2A)	99.4(4)
C(21A)-W(1)-P(2A)	93(3)
N(1A)-W(1)-P(2A)	78(2)
P(1A)-W(1)-P(2A)	151.7(6)
N(2)#1-N(2)-W(1)	173.7(7)
C(2)-P(1)-C(3)	102.2(5)
C(2)-P(1)-C(7)	105.1(6)
C(3)-P(1)-C(7)	109.6(5)
C(2)-P(1)-W(1)	100.1(4)
C(3)-P(1)-W(1)	121.2(4)
C(7)-P(1)-W(1)	115.7(4)
N(1)-C(1)-C(2)	114.2(10)
C(11)-N(1)-C(1)	108.4(9)
C(11)-N(1)-W(1)	125.5(12)
C(1)-N(1)-W(1)	126.1(12)
C(12)-P(2)-C(17)	105.1(5)
C(12)-P(2)-C(13)	105.9(6)
C(17)-P(2)-C(13)	109.4(5)
C(12)-P(2)-W(1)	100.1(4)
C(17)-P(2)-W(1)	119.4(4)
C(13)-P(2)-W(1)	115.1(4)

C(1)-C(2)-P(1)	111.4(8)	C(2A)-P(1A)-W(1)	99.0(13)
N(1)-C(11)-C(12)	114.3(10)	C(3A)-P(1A)-W(1)	118.5(13)
C(8)-C(7)-C(10)	107.8(9)	C(7A)-P(1A)-W(1)	118.9(13)
C(8)-C(7)-C(9)	107.7(9)	C(11A)-N(1A)-C(1A)	96(3)
C(10)-C(7)-C(9)	107.3(10)	C(11A)-N(1A)-W(1)	130(5)
C(8)-C(7)-P(1)	115.4(9)	C(1A)-N(1A)-W(1)	116(4)
C(10)-C(7)-P(1)	107.3(8)	C(2A)-C(1A)-N(1A)	113(4)
C(9)-C(7)-P(1)	111.0(9)	C(12A)-P(2A)-C(13A)	113(2)
C(6)-C(3)-C(5)	109.4(9)	C(12A)-P(2A)-C(17A)	104(2)
C(6)-C(3)-C(4)	107.9(9)	C(13A)-P(2A)-C(17A)	106.2(19)
C(5)-C(3)-C(4)	108.6(8)	C(12A)-P(2A)-W(1)	96.3(16)
C(6)-C(3)-P(1)	107.8(8)	C(13A)-P(2A)-W(1)	121.1(13)
C(5)-C(3)-P(1)	115.2(8)	C(17A)-P(2A)-W(1)	115.0(16)
C(4)-C(3)-P(1)	107.6(8)	C(1A)-C(2A)-P(1A)	109(3)
C(11)-C(12)-P(2)	108.8(7)	O(1A)-C(21A)-W(1)	171(8)
O(1)-C(21)-W(1)	172.7(17)	C(19A)-C(17A)-C(20A)	108.4(16)
C(18)-C(17)-C(20)	108.8(8)	C(19A)-C(17A)-C(18A)	108.0(15)
C(18)-C(17)-C(19)	108.7(9)	C(20A)-C(17A)-C(18A)	107.9(16)
C(20)-C(17)-C(19)	107.9(9)	C(19A)-C(17A)-P(2A)	120(4)
C(18)-C(17)-P(2)	115.2(11)	C(20A)-C(17A)-P(2A)	101(3)
C(20)-C(17)-P(2)	108.5(8)	C(18A)-C(17A)-P(2A)	111(3)
C(19)-C(17)-P(2)	107.6(8)	C(15A)-C(13A)-C(14A)	109.2(15)
C(16)-C(13)-C(15)	110.5(9)	C(15A)-C(13A)-C(16A)	108.8(15)
C(16)-C(13)-C(14)	107.4(9)	C(14A)-C(13A)-C(16A)	108.9(15)
C(15)-C(13)-C(14)	108.4(9)	C(15A)-C(13A)-P(2A)	110(3)
C(16)-C(13)-P(2)	110.5(8)	C(14A)-C(13A)-P(2A)	111(3)
C(15)-C(13)-P(2)	114.2(9)	C(16A)-C(13A)-P(2A)	108(3)
C(14)-C(13)-P(2)	105.5(8)	C(11A)-C(12A)-P(2A)	116(3)
C(2A)-P(1A)-C(3A)	103.6(17)	N(1A)-C(11A)-C(12A)	102(4)
C(2A)-P(1A)-C(7A)	101.7(19)	C(10A)-C(7A)-C(9A)	108.9(15)
C(3A)-P(1A)-C(7A)	111.2(18)	C(10A)-C(7A)-C(8A)	108.5(15)

C(9A)-C(7A)-C(8A)	108.7(15)	C(5A)-C(3A)-C(6A)	109.5(15)
C(10A)-C(7A)-P(1A)	111(3)	C(4A)-C(3A)-C(6A)	109.1(15)
C(9A)-C(7A)-P(1A)	100(3)	C(5A)-C(3A)-P(1A)	108(3)
C(8A)-C(7A)-P(1A)	119(3)	C(4A)-C(3A)-P(1A)	115(3)
C(5A)-C(3A)-C(4A)	109.2(15)	C(6A)-C(3A)-P(1A)	107(3)

Symmetry transformations used to generate equivalent atoms:

#1 -x+1,y,-z+3/2

Table S8 Torsion angles [°] for 3.

C(2)-C(1)-N(1)-C(11)	167.1(12)	C(7)-P(1)-C(3)-C(5)	61.2(9)
C(2)-C(1)-N(1)-W(1)	-14(2)	W(1)-P(1)-C(3)-C(5)	-159.8(7)
N(1)-C(1)-C(2)-P(1)	30.0(15)	C(2)-P(1)-C(3)-C(4)	-171.2(8)
C(3)-P(1)-C(2)-C(1)	-154.8(9)	C(7)-P(1)-C(3)-C(4)	-60.1(9)
C(7)-P(1)-C(2)-C(1)	90.8(9)	W(1)-P(1)-C(3)-C(4)	78.9(8)
W(1)-P(1)-C(2)-C(1)	-29.5(9)	N(1)-C(11)-C(12)-P(2)	-33.7(15)
C(1)-N(1)-C(11)-C(12)	-167.2(12)	C(17)-P(2)-C(12)-C(11)	159.5(9)
W(1)-N(1)-C(11)-C(12)	14(2)	C(13)-P(2)-C(12)-C(11)	-84.8(10)
C(2)-P(1)-C(7)-C(8)	64.1(10)	W(1)-P(2)-C(12)-C(11)	35.1(9)
C(3)-P(1)-C(7)-C(8)	-45.0(10)	C(12)-P(2)-C(17)-C(18)	72.0(9)
W(1)-P(1)-C(7)-C(8)	173.5(8)	C(13)-P(2)-C(17)-C(18)	-41.3(10)
C(2)-P(1)-C(7)-C(10)	-56.1(9)	W(1)-P(2)-C(17)-C(18)	-176.9(7)
C(3)-P(1)-C(7)-C(10)	-165.2(8)	C(12)-P(2)-C(17)-C(20)	-50.2(9)
W(1)-P(1)-C(7)-C(10)	53.3(9)	C(13)-P(2)-C(17)-C(20)	-163.5(8)
C(2)-P(1)-C(7)-C(9)	-173.0(9)	W(1)-P(2)-C(17)-C(20)	60.9(8)
C(3)-P(1)-C(7)-C(9)	77.9(10)	C(12)-P(2)-C(17)-C(19)	-166.7(8)
W(1)-P(1)-C(7)-C(9)	-63.6(9)	C(13)-P(2)-C(17)-C(19)	80.1(9)
C(2)-P(1)-C(3)-C(6)	72.7(9)	W(1)-P(2)-C(17)-C(19)	-55.5(9)
C(7)-P(1)-C(3)-C(6)	-176.2(8)	C(12)-P(2)-C(13)-C(16)	-164.4(8)
W(1)-P(1)-C(3)-C(6)	-37.2(9)	C(17)-P(2)-C(13)-C(16)	-51.6(9)
C(2)-P(1)-C(3)-C(5)	-49.9(9)	W(1)-P(2)-C(13)-C(16)	86.1(8)

C(12)-P(2)-C(13)-C(15)	-39.0(10)	C(12A)-P(2A)-C(13A)-C(16A)	163(2)
C(17)-P(2)-C(13)-C(15)	73.7(10)	C(17A)-P(2A)-C(13A)-C(16A)	50(3)
W(1)-P(2)-C(13)-C(15)	-148.5(8)	W(1)-P(2A)-C(13A)-C(16A)	-84(2)
C(12)-P(2)-C(13)-C(14)	79.9(8)	C(13A)-P(2A)-C(12A)-C(11A)	95(4)
C(17)-P(2)-C(13)-C(14)	-167.3(8)	C(17A)-P(2A)-C(12A)-C(11A)	-150(4)
W(1)-P(2)-C(13)-C(14)	-29.6(8)	W(1)-P(2A)-C(12A)-C(11A)	-33(4)
C(11A)-N(1A)-C(1A)-C(2A)	-179(4)	C(1A)-N(1A)-C(11A)-C(12A)	-175(4)
W(1)-N(1A)-C(1A)-C(2A)	40(6)	W(1)-N(1A)-C(11A)-C(12A)	-43(7)
N(1A)-C(1A)-C(2A)-P(1A)	-49(5)	P(2A)-C(12A)-C(11A)-N(1A)	45(5)
C(3A)-P(1A)-C(2A)-C(1A)	156(3)	C(2A)-P(1A)-C(7A)-C(10A)	-168(2)
C(7A)-P(1A)-C(2A)-C(1A)	-88(3)	C(3A)-P(1A)-C(7A)-C(10A)	-58(3)
W(1)-P(1A)-C(2A)-C(1A)	34(3)	W(1)-P(1A)-C(7A)-C(10A)	85(2)
C(12A)-P(2A)-C(17A)-C(19A)	-83(3)	C(2A)-P(1A)-C(7A)-C(9A)	77(2)
C(13A)-P(2A)-C(17A)-C(19A)	36(3)	C(3A)-P(1A)-C(7A)-C(9A)	-173(2)
W(1)-P(2A)-C(17A)-C(19A)	173(2)	W(1)-P(1A)-C(7A)-C(9A)	-30(2)
C(12A)-P(2A)-C(17A)-C(20A)	36(3)	C(2A)-P(1A)-C(7A)-C(8A)	-41(3)
C(13A)-P(2A)-C(17A)-C(20A)	155(2)	C(3A)-P(1A)-C(7A)-C(8A)	69(3)
W(1)-P(2A)-C(17A)-C(20A)	-68(3)	W(1)-P(1A)-C(7A)-C(8A)	-148(2)
C(12A)-P(2A)-C(17A)-C(18A)	150(3)	C(2A)-P(1A)-C(3A)-C(5A)	-47(2)
C(13A)-P(2A)-C(17A)-C(18A)	-91(3)	C(7A)-P(1A)-C(3A)-C(5A)	-156(2)
W(1)-P(2A)-C(17A)-C(18A)	46(3)	W(1)-P(1A)-C(3A)-C(5A)	61(2)
C(12A)-P(2A)-C(13A)-C(15A)	-78(3)	C(2A)-P(1A)-C(3A)-C(4A)	74(3)
C(17A)-P(2A)-C(13A)-C(15A)	169(3)	C(7A)-P(1A)-C(3A)-C(4A)	-34(3)
W(1)-P(2A)-C(13A)-C(15A)	35(3)	W(1)-P(1A)-C(3A)-C(4A)	-177.3(19)
C(12A)-P(2A)-C(13A)-C(14A)	43(3)	C(2A)-P(1A)-C(3A)-C(6A)	-165(2)
C(17A)-P(2A)-C(13A)-C(14A)	-70(3)	C(7A)-P(1A)-C(3A)-C(6A)	87(2)
W(1)-P(2A)-C(13A)-C(14A)	156.3(19)	W(1)-P(1A)-C(3A)-C(6A)	-56(2)

Symmetry transformations used to generate equivalent atoms:

#1 -x+1,y,-z+3/2

3.3 Crystal Structure of **5**

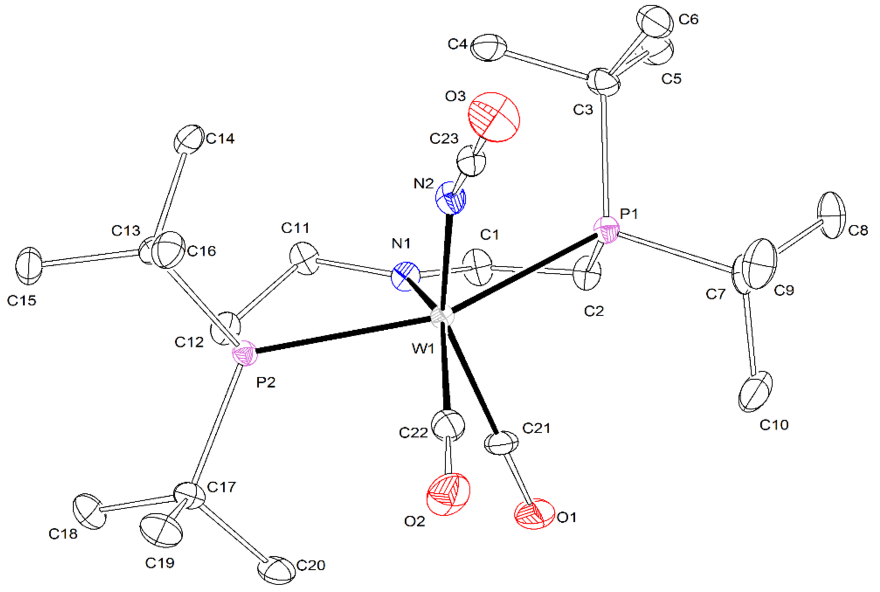

Figure S52 Thermal ellipsoid plot of **5** with the anisotropic displacement parameters drawn at the 50% probability level. The asymmetric unit contains one complex molecule. The reflections 2 0 0 and 8 9 16 are removed from the refinement using OMIT commands.

Table S9 Crystal data and structure refinement for 5.

Identification code	CCDC-2069380	
Empirical formula	$C_{23}H_{44}N_2O_3P_2W$	
Formula weight	642.39	
Temperature	100(2) K	
Wavelength	0.71073 Å	
Crystal system	Orthorhombic	
Space group	Pbca	
Unit cell dimensions	a = 16.8071(14) Å	α = 90°
	b = 13.5576(11) Å	β = 90°
	c = 23.5209(17) Å	γ = 90°
Volume	5359.6(7) Å^3	
Z	8	
Density (calculated)	1.592 Mg/m^3	
Absorption coefficient	4.455 mm^{-1}	
F(000)	2592	
Crystal size	0.231 x 0.093 x 0.051 mm^3	
Crystal shape and color	Plate, clear intense purple	

Theta range for data collection	2.593 to 27.927°
Index ranges	-22<=h<=21, -17<=k<=17, -30<=l<=30
Reflections collected	45436
Independent reflections	6275 [R(int) = 0.0687]
Completeness to theta = 25.242°	97.8 %
Refinement method	Full-matrix least-squares on F2
Data / restraints / parameters	6275 / 0 / 292
Goodness-of-fit on F2	1.025
Final R indices [I>2sigma(I)]	R1 = 0.0288, wR2 = 0.0647
R indices (all data)	R1 = 0.0462, wR2 = 0.0726
Largest diff. peak and hole	2.639 and -2.055 eÅ^{-3}

Table S10 Bond lengths [Å] and angles [°] for 5.

W(1)-C(21)	1.964(4)	C(3)-C(6)	1.527(6)
W(1)-N(1)	2.011(3)	C(3)-C(4)	1.541(6)
W(1)-C(22)	2.028(4)	C(3)-C(5)	1.545(5)
W(1)-N(2)	2.116(3)	C(7)-C(10)	1.536(6)
W(1)-P(2)	2.5030(10)	C(7)-C(9)	1.537(5)
W(1)-P(1)	2.5077(10)	C(7)-C(8)	1.541(5)
P(1)-C(2)	1.839(4)	C(11)-C(12)	1.514(5)
P(1)-C(7)	1.884(4)	C(13)-C(16)	1.533(5)
P(1)-C(3)	1.900(4)	C(13)-C(15)	1.541(5)
P(2)-C(12)	1.849(4)	C(13)-C(14)	1.541(5)
P(2)-C(13)	1.893(4)	C(17)-C(19)	1.535(5)
P(2)-C(17)	1.899(4)	C(17)-C(20)	1.541(5)
O(1)-C(21)	1.153(5)	C(17)-C(18)	1.548(5)
O(2)-C(22)	1.151(5)		
O(3)-C(23)	1.200(5)	C(21)-W(1)-N(1)	85.54(15)
N(1)-C(11)	1.478(5)	C(21)-W(1)-C(22)	77.03(16)
N(1)-C(1)	1.486(5)	N(1)-W(1)-C(22)	153.31(14)
N(2)-C(23)	1.176(5)	C(21)-W(1)-N(2)	146.74(15)
C(1)-C(2)	1.535(5)	N(1)-W(1)-N(2)	122.56(13)

C(22)-W(1)-N(2)	81.21(14)	C(6)-C(3)-C(5)	109.6(3)
C(21)-W(1)-P(2)	103.18(12)	C(4)-C(3)-C(5)	106.9(3)
N(1)-W(1)-P(2)	79.36(9)	C(6)-C(3)-P(1)	112.1(3)
C(22)-W(1)-P(2)	85.06(11)	C(4)-C(3)-P(1)	108.4(3)
N(2)-W(1)-P(2)	99.64(9)	C(5)-C(3)-P(1)	112.5(3)
C(21)-W(1)-P(1)	84.80(12)	C(10)-C(7)-C(9)	107.1(4)
N(1)-W(1)-P(1)	77.77(9)	C(10)-C(7)-C(8)	107.3(3)
C(22)-W(1)-P(1)	119.84(11)	C(9)-C(7)-C(8)	110.9(3)
N(2)-W(1)-P(1)	84.58(9)	C(10)-C(7)-P(1)	109.3(3)
P(2)-W(1)-P(1)	155.09(3)	C(9)-C(7)-P(1)	107.7(3)
C(2)-P(1)-C(7)	107.09(17)	C(8)-C(7)-P(1)	114.3(3)
C(2)-P(1)-C(3)	104.40(18)	N(1)-C(11)-C(12)	111.5(3)
C(7)-P(1)-C(3)	109.64(18)	C(11)-C(12)-P(2)	110.8(3)
C(2)-P(1)-W(1)	96.36(13)	C(16)-C(13)-C(15)	108.9(3)
C(7)-P(1)-W(1)	120.81(13)	C(16)-C(13)-C(14)	108.6(3)
C(3)-P(1)-W(1)	115.85(13)	C(15)-C(13)-C(14)	108.4(3)
C(12)-P(2)-C(13)	104.84(18)	C(16)-C(13)-P(2)	110.3(3)
C(12)-P(2)-C(17)	102.27(18)	C(15)-C(13)-P(2)	114.5(3)
C(13)-P(2)-C(17)	110.48(17)	C(14)-C(13)-P(2)	105.9(3)
C(12)-P(2)-W(1)	100.21(13)	C(19)-C(17)-C(20)	108.3(3)
C(13)-P(2)-W(1)	115.00(12)	C(19)-C(17)-C(18)	109.2(3)
C(17)-P(2)-W(1)	121.09(13)	C(20)-C(17)-C(18)	107.8(3)
C(11)-N(1)-C(1)	108.7(3)	C(19)-C(17)-P(2)	111.3(3)
C(11)-N(1)-W(1)	126.3(2)	C(20)-C(17)-P(2)	107.4(3)
C(1)-N(1)-W(1)	124.9(2)	C(18)-C(17)-P(2)	112.7(3)
C(23)-N(2)-W(1)	156.2(3)	O(1)-C(21)-W(1)	179.2(4)
N(1)-C(1)-C(2)	113.7(3)	O(2)-C(22)-W(1)	177.7(4)
C(1)-C(2)-P(1)	109.7(3)	N(2)-C(23)-O(3)	178.5(4)
C(6)-C(3)-C(4)	106.9(3)		

Symmetry transformations used to generate equivalent atoms:

Table S11 Torsion angles [°] for 5.

C(11)-N(1)-C(1)-C(2)	173.6(3)	W(1)-P(2)-C(12)-C(11)	-24.7(3)
W(1)-N(1)-C(1)-C(2)	-8.7(5)	C(12)-P(2)-C(13)-C(16)	-173.6(3)
N(1)-C(1)-C(2)-P(1)	-26.3(4)	C(17)-P(2)-C(13)-C(16)	76.9(3)
C(7)-P(1)-C(2)-C(1)	164.3(3)	W(1)-P(2)-C(13)-C(16)	-64.5(3)
C(3)-P(1)-C(2)-C(1)	-79.4(3)	C(12)-P(2)-C(13)-C(15)	63.1(3)
W(1)-P(1)-C(2)-C(1)	39.4(3)	C(17)-P(2)-C(13)-C(15)	-46.4(3)
C(2)-P(1)-C(7)-C(10)	-49.8(3)	W(1)-P(2)-C(13)-C(15)	172.1(2)
C(3)-P(1)-C(7)-C(10)	-162.5(3)	C(12)-P(2)-C(13)-C(14)	-56.3(3)
W(1)-P(1)-C(7)-C(10)	58.8(3)	C(17)-P(2)-C(13)-C(14)	-165.8(2)
C(2)-P(1)-C(7)-C(9)	-165.9(3)	W(1)-P(2)-C(13)-C(14)	52.8(3)
C(3)-P(1)-C(7)-C(9)	81.4(3)	C(12)-P(2)-C(17)-C(19)	-162.2(3)
W(1)-P(1)-C(7)-C(9)	-57.3(3)	C(13)-P(2)-C(17)-C(19)	-51.0(3)
C(2)-P(1)-C(7)-C(8)	70.4(3)	W(1)-P(2)-C(17)-C(19)	87.7(3)
C(3)-P(1)-C(7)-C(8)	-42.3(3)	C(12)-P(2)-C(17)-C(20)	79.5(3)
W(1)-P(1)-C(7)-C(8)	179.0(2)	C(13)-P(2)-C(17)-C(20)	-169.3(3)
C(1)-N(1)-C(11)-C(12)	141.4(3)	W(1)-P(2)-C(17)-C(20)	-30.6(3)
W(1)-N(1)-C(11)-C(12)	-36.3(4)	C(12)-P(2)-C(17)-C(18)	-39.1(3)
N(1)-C(11)-C(12)-P(2)	37.3(4)	C(13)-P(2)-C(17)-C(18)	72.1(3)
C(13)-P(2)-C(12)-C(11)	94.7(3)	W(1)-P(2)-C(17)-C(18)	-149.1(2)
C(17)-P(2)-C(12)-C(11)	-149.9(3)		

Symmetry transformations used to generate equivalent atoms:

3.4 Crystal Structure of **6a**

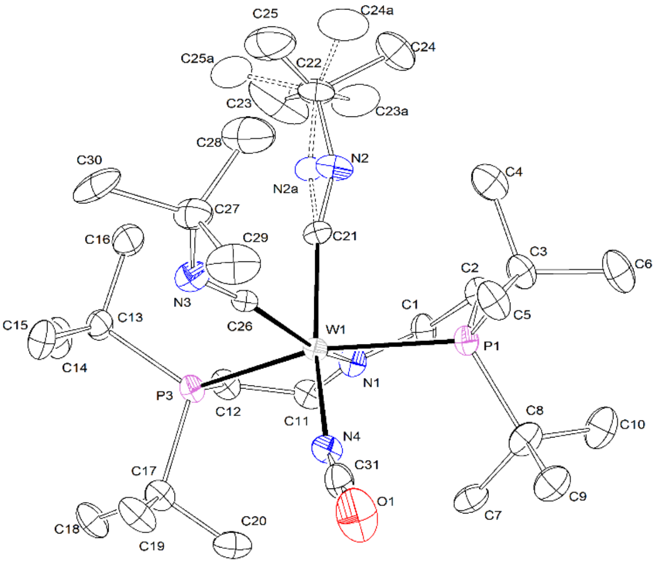

Figure S53 Thermal ellipsoid plot of **6a** with the anisotropic displacement parameters drawn at the 50% probability level. The asymmetric unit contains one disordered complex molecule. The disordered complex molecule was refined with population of 0.67(1) on the main domain using some restraints (RIGU, SADI). The structure was refined as an inversion twin using the twin law -100 0-10 00-1 (BASF: 0.03(1)).

Table S12 Crystal data and structure refinement for 6a.

Identification code	CCDC-2069381	
Empirical formula	$C_{31}H_{62}N_4OP_2W$	
Formula weight	752.63	
Temperature	100(2) K	
Wavelength	0.71073 Å	
Crystal system	Orthorhombic	
Space group	$Pna2_1$	
Unit cell dimensions	a = 13.5380(9) Å	α = 90°
	b = 18.3332(12) Å	β= 90°
	c = 14.4776(9) Å	γ = 90°
Volume	3593.3(4) Å3	
Z	4	
Density (calculated)	1.391 Mg/m^3	
Absorption coefficient	3.331 mm^{-1}	
F(000)	1552	

Crystal size	0.357 x 0.252 x 0.208 mm^3
Crystal shape and color	Block, dark green
Theta range for data collection	2.340 to 30.602°
Index ranges	-19<=h<=19, -26<=k<=26, -20<=l<=20
Reflections collected	59367
Independent reflections	11011 [R(int) = 0.1325]
Completeness to theta = 25.242°	99.7 %
Refinement method	Full-matrix least-squares on F2
Data / restraints / parameters	11011 / 94 / 409
Goodness-of-fit on F2	1.003
Final R indices [I>2sigma(I)]	R1 = 0.0431, wR2 = 0.0698
R indices (all data)	R1 = 0.0833, wR2 = 0.0811
Absolute structure parameter	0.036(12)
Largest diff. peak and hole	2.271 and -1.198 $eÅ^{-3}$

Table S13 Bond lengths [Å] and angles [°] for 6a.

P(1)-C(2)	1.837(8)	N(4)-W(1)	2.102(13)
P(1)-C(3)	1.887(9)	C(1)-C(2)	1.530(12)
P(1)-C(8)	1.904(8)	C(3)-C(4)	1.527(12)
P(1)-W(1)	2.4906(18)	C(3)-C(6)	1.534(11)
P(3)-C(12)	1.848(8)	C(3)-C(5)	1.539(11)
P(3)-C(17)	1.891(9)	C(7)-C(8)	1.544(11)
P(3)-C(13)	1.914(9)	C(8)-C(10)	1.513(14)
P(3)-W(1)	2.4533(19)	C(8)-C(9)	1.565(16)
O(1)-C(31)	1.186(11)	C(11)-C(12)	1.506(12)
N(1)-C(11)	1.467(14)	C(13)-C(16)	1.523(13)
N(1)-C(1)	1.468(13)	C(13)-C(14)	1.536(12)
N(1)-W(1)	2.028(11)	C(13)-C(15)	1.538(12)
N(3)-C(26)	1.205(10)	C(17)-C(18)	1.522(11)
N(3)-C(27)	1.470(12)	C(17)-C(20)	1.543(12)
N(4)-C(31)	1.194(14)	C(17)-C(19)	1.557(12)

C(26)-W(1)	2.035(8)	C(26)-N(3)-C(27)	134.5(8)
C(27)-C(28)	1.505(15)	C(31)-N(4)-W(1)	152.6(11)
C(27)-C(30)	1.508(14)	N(1)-C(1)-C(2)	114.7(8)
C(27)-C(29)	1.529(14)	C(1)-C(2)-P(1)	108.4(6)
W(1)-C(21)	2.014(8)	C(4)-C(3)-C(6)	108.4(7)
C(21)-N(2A)	1.17(4)	C(4)-C(3)-C(5)	107.8(8)
C(21)-N(2)	1.198(18)	C(6)-C(3)-C(5)	109.4(8)
C(22)-N(2A)	1.43(4)	C(4)-C(3)-P(1)	108.1(6)
C(22)-N(2)	1.459(19)	C(6)-C(3)-P(1)	115.9(6)
C(22)-C(23)	1.477(16)	C(5)-C(3)-P(1)	107.1(6)
C(22)-C(24A)	1.48(2)	C(10)-C(8)-C(7)	108.5(9)
C(22)-C(25)	1.507(16)	C(10)-C(8)-C(9)	107.2(8)
C(22)-C(23A)	1.52(2)	C(7)-C(8)-C(9)	106.1(9)
C(22)-C(25A)	1.54(2)	C(10)-C(8)-P(1)	113.4(8)
C(22)-C(24)	1.537(14)	C(7)-C(8)-P(1)	109.1(5)
		C(9)-C(8)-P(1)	112.3(7)
C(2)-P(1)-C(3)	106.6(4)	N(1)-C(11)-C(12)	112.4(8)
C(2)-P(1)-C(8)	102.7(5)	C(11)-C(12)-P(3)	111.6(6)
C(3)-P(1)-C(8)	109.2(4)	C(16)-C(13)-C(14)	109.3(8)
C(2)-P(1)-W(1)	97.1(3)	C(16)-C(13)-C(15)	108.0(8)
C(3)-P(1)-W(1)	120.8(3)	C(14)-C(13)-C(15)	110.1(7)
C(8)-P(1)-W(1)	117.2(3)	C(16)-C(13)-P(3)	106.0(6)
C(12)-P(3)-C(17)	104.1(4)	C(14)-C(13)-P(3)	112.9(7)
C(12)-P(3)-C(13)	101.9(4)	C(15)-C(13)-P(3)	110.3(6)
C(17)-P(3)-C(13)	108.1(4)	C(18)-C(17)-C(20)	108.1(8)
C(12)-P(3)-W(1)	100.6(3)	C(18)-C(17)-C(19)	109.0(7)
C(17)-P(3)-W(1)	115.6(3)	C(20)-C(17)-C(19)	108.1(8)
C(13)-P(3)-W(1)	123.4(3)	C(18)-C(17)-P(3)	116.3(6)
C(11)-N(1)-C(1)	109.1(9)	C(20)-C(17)-P(3)	106.2(6)
C(11)-N(1)-W(1)	126.1(7)	C(19)-C(17)-P(3)	108.8(6)
C(1)-N(1)-W(1)	124.5(8)	N(3)-C(26)-W(1)	172.3(7)

N(3)-C(27)-C(28)	109.9(8)	C(26)-W(1)-P(1)	117.1(2)
N(3)-C(27)-C(30)	107.8(8)	N(4)-W(1)-P(1)	84.0(3)
C(28)-C(27)-C(30)	110.3(9)	P(3)-W(1)-P(1)	155.23(7)
N(3)-C(27)-C(29)	107.2(8)	N(2A)-C(21)-W(1)	172(2)
C(28)-C(27)-C(29)	111.2(10)	N(2)-C(21)-W(1)	167.2(11)
C(30)-C(27)-C(29)	110.4(9)	N(2)-C(22)-C(23)	112.3(14)
O(1)-C(31)-N(4)	177.0(11)	N(2A)-C(22)-C(24A)	118(3)
C(21)-W(1)-N(1)	82.0(4)	N(2)-C(22)-C(25)	106.3(13)
C(21)-W(1)-C(26)	80.5(3)	C(23)-C(22)-C(25)	115.7(16)
N(1)-W(1)-C(26)	156.6(4)	N(2A)-C(22)-C(23A)	107(3)
C(21)-W(1)-N(4)	153.4(4)	C(24A)-C(22)-C(23A)	117(2)
N(1)-W(1)-N(4)	120.1(3)	N(2A)-C(22)-C(25A)	102(2)
C(26)-W(1)-N(4)	81.3(4)	C(24A)-C(22)-C(25A)	105(2)
C(21)-W(1)-P(3)	99.7(2)	C(23A)-C(22)-C(25A)	106(2)
N(1)-W(1)-P(3)	80.2(3)	N(2)-C(22)-C(24)	104.6(11)
C(26)-W(1)-P(3)	87.6(2)	C(23)-C(22)-C(24)	110.6(13)
N(4)-W(1)-P(3)	98.7(3)	C(25)-C(22)-C(24)	106.6(12)
C(21)-W(1)-P(1)	87.3(2)	C(21)-N(2)-C(22)	153.7(16)
N(1)-W(1)-P(1)	77.3(3)	C(21)-N(2A)-C(22)	169(3)

Symmetry transformations used to generate equivalent atoms:

Table S14 Torsion angles [°] for 6a.

C(11)-N(1)-C(1)-C(2)	-178.8(9)	W(1)-P(1)-C(3)-C(4)	-58.1(6)
W(1)-N(1)-C(1)-C(2)	7.4(14)	C(2)-P(1)-C(3)-C(6)	-70.6(8)
N(1)-C(1)-C(2)-P(1)	27.2(11)	C(8)-P(1)-C(3)-C(6)	39.7(9)
C(3)-P(1)-C(2)-C(1)	-165.4(6)	W(1)-P(1)-C(3)-C(6)	-179.9(6)
C(8)-P(1)-C(2)-C(1)	79.8(7)	C(2)-P(1)-C(3)-C(5)	167.0(6)
W(1)-P(1)-C(2)-C(1)	-40.2(6)	C(8)-P(1)-C(3)-C(5)	-82.6(8)
C(2)-P(1)-C(3)-C(4)	51.2(7)	W(1)-P(1)-C(3)-C(5)	57.8(7)
C(8)-P(1)-C(3)-C(4)	161.5(7)	C(1)-N(1)-C(11)-C(12)	-144.7(9)

W(1)-N(1)-C(11)-C(12)	29.0(14)	C(13)-P(3)-C(17)-C(19)	-80.0(7)
N(1)-C(11)-C(12)-P(3)	-33.5(11)	W(1)-P(3)-C(17)-C(19)	62.9(7)
C(17)-P(3)-C(12)-C(11)	-95.5(7)	C(26)-N(3)-C(27)-C(28)	41.1(15)
C(13)-P(3)-C(12)-C(11)	152.2(7)	C(26)-N(3)-C(27)-C(30)	161.3(10)
W(1)-P(3)-C(12)-C(11)	24.5(7)	C(26)-N(3)-C(27)-C(29)	-79.9(13)
C(12)-P(3)-C(17)-C(18)	-64.3(7)	W(1)-C(21)-N(2)-C(22)	169(2)
C(13)-P(3)-C(17)-C(18)	43.5(8)	C(23)-C(22)-N(2)-C(21)	-56(5)
W(1)-P(3)-C(17)-C(18)	-173.6(6)	C(25)-C(22)-N(2)-C(21)	71(5)
C(12)-P(3)-C(17)-C(20)	56.0(6)	C(24)-C(22)-N(2)-C(21)	-176(4)
C(13)-P(3)-C(17)-C(20)	163.8(6)	C(24A)-C(22)-N(2A)-C(21)	-73(27)
W(1)-P(3)-C(17)-C(20)	-53.3(6)	C(23A)-C(22)-N(2A)-C(21)	61(27)
C(12)-P(3)-C(17)-C(19)	172.2(6)	C(25A)-C(22)-N(2A)-C(21)	172(26)

Symmetry transformations used to generate equivalent atoms:

3.5 Crystal Structure of **6b**

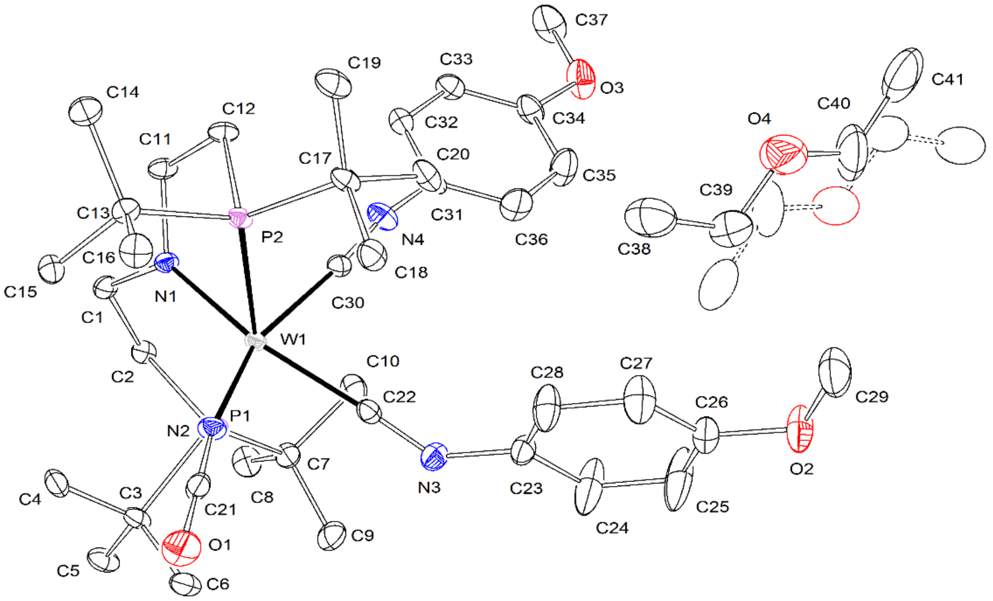

Figure S54 Thermal ellipsoid plot of **6b** with the anisotropic displacement parameters drawn at the 50% probability level. The asymmetric unit contains one complex molecule and a half disordered diethyl ether solvent molecule. The disorder was refined using PART -1 command.

Table S15 Crystal data and structure refinement for 6b.

Identification code	CCDC-2069382	
Empirical formula	$C_{37}H_{58}N_4O_3P_2W$ * ½ $C_4H_{10}O$	
Formula weight	889.72	
Temperature	100(2) K	
Wavelength	0.71073 Å	
Crystal system	Triclinic	
Space group	P-1	
Unit cell dimensions	a = 9.4010(6) Å	α = 104.902(2)°
	b = 11.0756(7) Å	β = 94.164(3)°
	c = 20.7216(14) Å	γ = 101.531(3)°
Volume	2025.3(2) Å3	
Z	2	
Density (calculated)	1.459 Mg/m^3	
Absorption coefficient	2.972 mm^{-1}	
F(000)	914	
Crystal size	0.391 x 0.113 x 0.094 mm^3	

Crystal shape and color	Plate, dark brown
Theta range for data collection	2.230 to 28.394°
Index ranges	-12<=h<=12, -14<=k<=14, -27<=l<=27
Reflections collected	140974
Independent reflections	10120 [R(int) = 0.0882]
Completeness to theta = 25.242°	99.9 %
Refinement method	Full-matrix least-squares on F2
Data / restraints / parameters	10120 / 0 / 485
Goodness-of-fit on F2	1.090
Final R indices [I>2sigma(I)]	R1 = 0.0244, wR2 = 0.0468
R indices (all data)	R1 = 0.0343, wR2 = 0.0501
Largest diff. peak and hole	1.922 and -1.107 eÅ^{-3}

Table S16 Bond lengths [Å] and angles [°] for 6b.

W(1)-C(30)	2.000(2)	N(1)-C(11)	1.479(3)
W(1)-C(22)	2.020(2)	N(2)-C(21)	1.172(3)
W(1)-N(1)	2.020(2)	N(3)-C(22)	1.212(3)
W(1)-N(2)	2.118(2)	N(3)-C(23)	1.414(3)
W(1)-P(1)	2.4735(6)	N(4)-C(30)	1.184(3)
W(1)-P(2)	2.4960(6)	N(4)-C(31)	1.389(3)
P(1)-C(2)	1.841(2)	C(1)-C(2)	1.523(3)
P(1)-C(3)	1.889(2)	C(3)-C(5)	1.532(3)
P(1)-C(7)	1.890(3)	C(3)-C(6)	1.542(3)
P(2)-C(12)	1.838(2)	C(3)-C(4)	1.543(4)
P(2)-C(17)	1.888(3)	C(7)-C(9)	1.537(4)
P(2)-C(13)	1.909(3)	C(7)-C(10)	1.539(4)
O(1)-C(21)	1.204(3)	C(7)-C(8)	1.541(4)
O(2)-C(26)	1.371(3)	C(11)-C(12)	1.532(3)
O(2)-C(29)	1.416(4)	C(13)-C(16)	1.532(3)
O(3)-C(34)	1.371(3)	C(13)-C(14)	1.538(3)
O(3)-C(37)	1.429(4)	C(13)-C(15)	1.543(3)
N(1)-C(1)	1.474(3)	C(17)-C(20)	1.535(4)

C(17)-C(19)	1.535(3)	N(1)-W(1)-P(2)	76.82(6)
C(17)-C(18)	1.542(3)	N(2)-W(1)-P(2)	85.98(6)
C(23)-C(28)	1.367(4)	P(1)-W(1)-P(2)	155.36(2)
C(23)-C(24)	1.375(4)	C(2)-P(1)-C(3)	104.95(11)
C(24)-C(25)	1.385(5)	C(2)-P(1)-C(7)	102.13(12)
C(25)-C(26)	1.375(4)	C(3)-P(1)-C(7)	109.45(11)
C(26)-C(27)	1.369(4)	C(2)-P(1)-W(1)	100.95(8)
C(27)-C(28)	1.390(4)	C(3)-P(1)-W(1)	114.46(8)
C(31)-C(32)	1.385(4)	C(7)-P(1)-W(1)	122.15(8)
C(31)-C(36)	1.394(4)	C(12)-P(2)-C(17)	105.41(11)
C(32)-C(33)	1.391(4)	C(12)-P(2)-C(13)	102.98(11)
C(33)-C(34)	1.386(4)	C(17)-P(2)-C(13)	110.08(11)
C(34)-C(35)	1.387(4)	C(12)-P(2)-W(1)	96.62(8)
C(35)-C(36)	1.383(4)	C(17)-P(2)-W(1)	120.02(8)
O(4)-C(40)	1.393(10)	C(13)-P(2)-W(1)	118.14(8)
O(4)-C(39)	1.406(10)	C(26)-O(2)-C(29)	117.5(2)
C(38)-C(39)	1.518(12)	C(34)-O(3)-C(37)	117.4(2)
C(40)-C(41)	1.447(15)	C(1)-N(1)-C(11)	109.13(18)
		C(1)-N(1)-W(1)	125.75(15)
C(30)-W(1)-C(22)	78.70(10)	C(11)-N(1)-W(1)	124.73(15)
C(30)-W(1)-N(1)	82.18(9)	C(21)-N(2)-W(1)	170.6(2)
C(22)-W(1)-N(1)	155.95(9)	C(22)-N(3)-C(23)	132.4(2)
C(30)-W(1)-N(2)	155.48(9)	C(30)-N(4)-C(31)	161.6(3)
C(22)-W(1)-N(2)	83.42(9)	N(1)-C(1)-C(2)	111.9(2)
N(1)-W(1)-N(2)	118.84(8)	C(1)-C(2)-P(1)	110.60(16)
C(30)-W(1)-P(1)	97.65(7)	C(5)-C(3)-C(6)	109.9(2)
C(22)-W(1)-P(1)	88.34(7)	C(5)-C(3)-C(4)	107.8(2)
N(1)-W(1)-P(1)	79.86(6)	C(6)-C(3)-C(4)	108.9(2)
N(2)-W(1)-P(1)	98.47(6)	C(5)-C(3)-P(1)	114.99(18)
C(30)-W(1)-P(2)	87.03(7)	C(6)-C(3)-P(1)	109.02(17)
C(22)-W(1)-P(2)	116.29(7)	C(4)-C(3)-P(1)	106.05(16)

C(9)-C(7)-C(10)	108.3(2)	C(28)-C(23)-N(3)	122.8(3)
C(9)-C(7)-C(8)	109.4(2)	C(24)-C(23)-N(3)	118.7(3)
C(10)-C(7)-C(8)	107.0(2)	C(23)-C(24)-C(25)	120.3(3)
C(9)-C(7)-P(1)	110.68(19)	C(26)-C(25)-C(24)	120.7(3)
C(10)-C(7)-P(1)	108.06(17)	C(27)-C(26)-O(2)	124.8(3)
C(8)-C(7)-P(1)	113.24(18)	C(27)-C(26)-C(25)	119.4(3)
N(1)-C(11)-C(12)	113.69(19)	O(2)-C(26)-C(25)	115.8(3)
C(11)-C(12)-P(2)	108.58(16)	C(26)-C(27)-C(28)	119.4(3)
C(16)-C(13)-C(14)	108.8(2)	C(23)-C(28)-C(27)	121.7(3)
C(16)-C(13)-C(15)	106.8(2)	N(4)-C(30)-W(1)	173.9(2)
C(14)-C(13)-C(15)	107.1(2)	C(32)-C(31)-N(4)	120.7(2)
C(16)-C(13)-P(2)	112.39(17)	C(32)-C(31)-C(36)	119.3(2)
C(14)-C(13)-P(2)	113.09(17)	N(4)-C(31)-C(36)	119.9(2)
C(15)-C(13)-P(2)	108.40(16)	C(31)-C(32)-C(33)	120.9(2)
C(20)-C(17)-C(19)	109.2(2)	C(34)-C(33)-C(32)	119.5(2)
C(20)-C(17)-C(18)	106.8(2)	O(3)-C(34)-C(33)	124.6(2)
C(19)-C(17)-C(18)	110.2(2)	O(3)-C(34)-C(35)	115.6(3)
C(20)-C(17)-P(2)	107.52(17)	C(33)-C(34)-C(35)	119.8(3)
C(19)-C(17)-P(2)	113.76(18)	C(36)-C(35)-C(34)	120.6(3)
C(18)-C(17)-P(2)	109.14(16)	C(35)-C(36)-C(31)	119.9(3)
N(2)-C(21)-O(1)	178.8(3)	C(40)-O(4)-C(39)	114.0(10)
N(3)-C(22)-W(1)	174.3(2)	O(4)-C(39)-C(38)	107.8(6)
C(28)-C(23)-C(24)	118.5(3)	O(4)-C(40)-C(41)	109.4(11)

Symmetry transformations used to generate equivalent atoms:

Table S17 Torsion angles [°] for 6b.

C(11)-N(1)-C(1)-C(2)	138.9(2)	C(12)-P(2)-C(17)-C(20)	-61.89(19)
W(1)-N(1)-C(1)-C(2)	-34.1(3)	C(13)-P(2)-C(17)-C(20)	-172.30(16)
N(1)-C(1)-C(2)-P(1)	36.2(2)	W(1)-P(2)-C(17)-C(20)	45.40(19)
C(3)-P(1)-C(2)-C(1)	94.67(18)	C(12)-P(2)-C(17)-C(19)	59.2(2)
C(7)-P(1)-C(2)-C(1)	-151.13(17)	C(13)-P(2)-C(17)-C(19)	-51.2(2)
W(1)-P(1)-C(2)-C(1)	-24.55(18)	W(1)-P(2)-C(17)-C(19)	166.49(15)
C(2)-P(1)-C(3)-C(5)	63.2(2)	C(12)-P(2)-C(17)-C(18)	-177.33(17)
C(7)-P(1)-C(3)-C(5)	-45.8(2)	C(13)-P(2)-C(17)-C(18)	72.26(19)
W(1)-P(1)-C(3)-C(5)	172.90(16)	W(1)-P(2)-C(17)-C(18)	-70.03(19)
C(2)-P(1)-C(3)-C(6)	-172.94(17)	C(22)-N(3)-C(23)-C(28)	27.7(5)
C(7)-P(1)-C(3)-C(6)	78.1(2)	C(22)-N(3)-C(23)-C(24)	-152.9(4)
W(1)-P(1)-C(3)-C(6)	-63.23(19)	C(28)-C(23)-C(24)-C(25)	-3.0(6)
C(2)-P(1)-C(3)-C(4)	-55.84(19)	N(3)-C(23)-C(24)-C(25)	177.6(4)
C(7)-P(1)-C(3)-C(4)	-164.80(17)	C(23)-C(24)-C(25)-C(26)	0.9(7)
W(1)-P(1)-C(3)-C(4)	53.87(18)	C(29)-O(2)-C(26)-C(27)	-7.4(5)
C(2)-P(1)-C(7)-C(9)	-161.54(18)	C(29)-O(2)-C(26)-C(25)	172.7(4)
C(3)-P(1)-C(7)-C(9)	-50.7(2)	C(24)-C(25)-C(26)-C(27)	1.8(7)
W(1)-P(1)-C(7)-C(9)	87.09(19)	C(24)-C(25)-C(26)-O(2)	-178.4(4)
C(2)-P(1)-C(7)-C(10)	80.07(19)	O(2)-C(26)-C(27)-C(28)	177.9(3)
C(3)-P(1)-C(7)-C(10)	-169.10(17)	C(25)-C(26)-C(27)-C(28)	-2.3(5)
W(1)-P(1)-C(7)-C(10)	-31.3(2)	C(24)-C(23)-C(28)-C(27)	2.5(5)
C(2)-P(1)-C(7)-C(8)	-38.3(2)	N(3)-C(23)-C(28)-C(27)	-178.1(3)
C(3)-P(1)-C(7)-C(8)	72.6(2)	C(26)-C(27)-C(28)-C(23)	0.2(5)
W(1)-P(1)-C(7)-C(8)	-149.65(16)	C(30)-N(4)-C(31)-C(32)	109.0(8)
C(1)-N(1)-C(11)-C(12)	177.1(2)	C(30)-N(4)-C(31)-C(36)	-75.4(9)
W(1)-N(1)-C(11)-C(12)	-9.7(3)	N(4)-C(31)-C(32)-C(33)	176.9(2)
N(1)-C(11)-C(12)-P(2)	-26.8(3)	C(36)-C(31)-C(32)-C(33)	1.4(4)
C(17)-P(2)-C(12)-C(11)	164.90(17)	C(31)-C(32)-C(33)-C(34)	-1.6(4)
C(13)-P(2)-C(12)-C(11)	-79.70(19)	C(37)-O(3)-C(34)-C(33)	-1.7(4)
W(1)-P(2)-C(12)-C(11)	41.23(17)	C(37)-O(3)-C(34)-C(35)	179.0(3)

C(32)-C(33)-C(34)-O(3)	-178.6(3)	C(32)-C(31)-C(36)-C(35)	-0.2(4)
C(32)-C(33)-C(34)-C(35)	0.7(4)	N(4)-C(31)-C(36)-C(35)	-175.8(3)
O(3)-C(34)-C(35)-C(36)	179.8(3)	C(40)-O(4)-C(39)-C(38)	-173.2(7)
C(33)-C(34)-C(35)-C(36)	0.5(5)	C(39)-O(4)-C(40)-C(41)	-178.8(8)
C(34)-C(35)-C(36)-C(31)	-0.7(5)		

Symmetry transformations used to generate equivalent atoms:

3.6 Crystal Structure of **7**

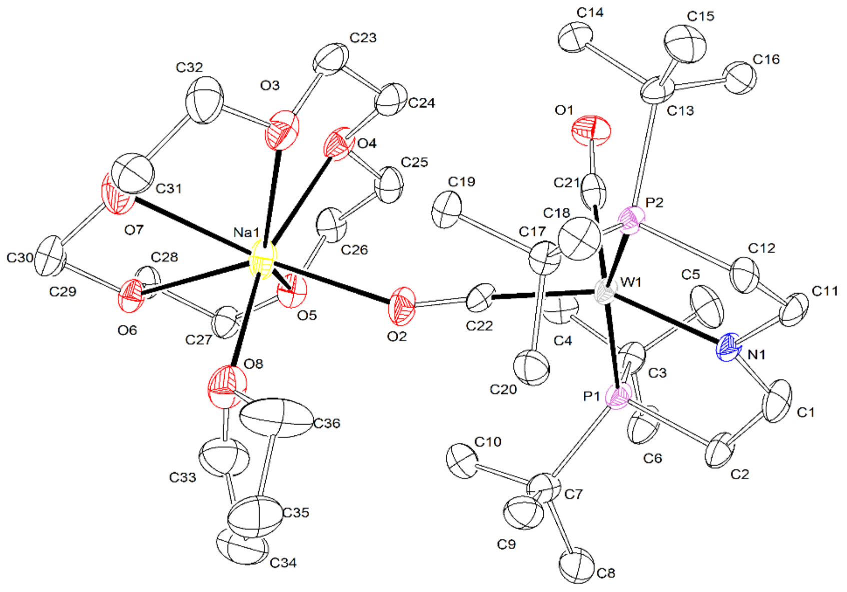

Figure S55 Thermal ellipsoid plot of **7** with the anisotropic displacement parameters drawn at the 50% probability level. The asymmetric unit contains one complex molecule. The reflections -5 5 6 and -5 5 8 are removed from the refinement using OMIT commands.

Table S18 Crystal data and structure refinement for 7.

Identification code	CCDC-2069383	
Empirical formula	$C_{36}H_{72}NNaO_8P_2W$	
Formula weight	915.72	
Temperature	100(2) K	
Wavelength	0.71073 Å	
Crystal system	Monoclinic	
Space group	$P2_1/n$	
Unit cell dimensions	a = 10.2148(9) Å	α = 90°
	b = 21.2618(17) Å	β = 93.444(3)°
	c = 19.0681(17) Å	γ = 90°
Volume	4133.8(6) Å3	
Z	4	
Density (calculated)	1.471 Mg/m^3	
Absorption coefficient	2.929 mm^{-1}	
F(000)	1896	
Crystal size	0.451 x 0.231 x 0.068 mm^3	

Crystal shape and color	Plate, clear intense orange
Theta range for data collection	2.194 to 25.759°
Index ranges	-12<=h<=12, -25<=k<=22, -23<=l<=23
Reflections collected	114649
Independent reflections	7903 [R(int) = 0.0962]
Completeness to theta = 25.242°	99.9 %
Refinement method	Full-matrix least-squares on F2
Data / restraints / parameters	7903 / 0 / 454
Goodness-of-fit on F2	1.350
Final R indices [I>2sigma(I)]	R1 = 0.0521, wR2 = 0.1305
R indices (all data)	R1 = 0.0601, wR2 = 0.1345
Largest diff. peak and hole	2.155 and -1.946 eÅ^{-3}

Table S19 Bond lengths [Å] and angles [°] for 7.

W(1)-C(21)	1.902(9)	Na(1)-O(6)	2.565(6)
W(1)-C(22)	1.911(8)	Na(1)-C(33)	3.127(11)
W(1)-N(1)	2.088(6)	Na(1)-C(25)	3.130(9)
W(1)-P(1)	2.426(2)	O(1)-C(21)	1.206(10)
W(1)-P(2)	2.4484(19)	O(2)-C(22)	1.205(9)
P(1)-C(2)	1.849(8)	O(3)-C(32)	1.409(11)
P(1)-C(3)	1.895(9)	O(3)-C(23)	1.433(11)
P(1)-C(7)	1.900(9)	O(4)-C(25)	1.426(10)
P(2)-C(12)	1.847(8)	O(4)-C(24)	1.434(10)
P(2)-C(17)	1.894(8)	O(5)-C(27)	1.425(10)
P(2)-C(13)	1.895(8)	O(5)-C(26)	1.430(10)
Na(1)-O(2)	2.329(6)	O(6)-C(28)	1.428(9)
Na(1)-O(8)	2.382(7)	O(6)-C(29)	1.427(10)
Na(1)-O(4)	2.439(7)	O(7)-C(31)	1.383(11)
Na(1)-O(3)	2.464(7)	O(7)-C(30)	1.470(11)
Na(1)-O(5)	2.487(6)	O(8)-C(33)	1.370(13)
Na(1)-O(7)	2.535(7)	O(8)-C(36)	1.441(11)

N(1)-C(1)	1.457(11)	C(21)-W(1)-P(2)	101.0(2)
N(1)-C(11)	1.458(10)	C(22)-W(1)-P(2)	98.0(2)
C(1)-C(2)	1.494(11)	N(1)-W(1)-P(2)	78.97(18)
C(3)-C(4)	1.526(12)	P(1)-W(1)-P(2)	156.74(6)
C(3)-C(5)	1.531(12)	C(2)-P(1)-C(3)	105.1(4)
C(3)-C(6)	1.541(11)	C(2)-P(1)-C(7)	100.3(4)
C(7)-C(8)	1.525(12)	C(3)-P(1)-C(7)	109.2(4)
C(7)-C(10)	1.532(12)	C(2)-P(1)-W(1)	102.2(3)
C(7)-C(9)	1.549(12)	C(3)-P(1)-W(1)	119.8(3)
C(11)-C(12)	1.523(11)	C(7)-P(1)-W(1)	117.2(3)
C(13)-C(14)	1.529(10)	C(12)-P(2)-C(17)	101.9(4)
C(13)-C(16)	1.532(12)	C(12)-P(2)-C(13)	103.0(4)
C(13)-C(15)	1.538(10)	C(17)-P(2)-C(13)	110.6(4)
C(17)-C(18)	1.531(11)	C(12)-P(2)-W(1)	102.6(3)
C(17)-C(19)	1.533(10)	C(17)-P(2)-W(1)	115.2(3)
C(17)-C(20)	1.542(11)	C(13)-P(2)-W(1)	120.4(2)
C(23)-C(24)	1.491(13)	O(2)-Na(1)-O(8)	78.7(2)
C(25)-C(26)	1.501(12)	O(2)-Na(1)-O(4)	83.9(2)
C(27)-C(28)	1.502(11)	O(8)-Na(1)-O(4)	162.2(2)
C(29)-C(30)	1.494(12)	O(2)-Na(1)-O(3)	88.1(2)
C(31)-C(32)	1.487(14)	O(8)-Na(1)-O(3)	107.4(3)
C(33)-C(34)	1.516(15)	O(4)-Na(1)-O(3)	68.1(2)
C(34)-C(35)	1.550(16)	O(2)-Na(1)-O(5)	91.0(2)
C(35)-C(36)	1.456(15)	O(8)-Na(1)-O(5)	114.1(3)
		O(4)-Na(1)-O(5)	69.5(2)
C(21)-W(1)-C(22)	87.7(3)	O(3)-Na(1)-O(5)	137.5(3)
C(21)-W(1)-N(1)	124.3(3)	O(2)-Na(1)-O(7)	144.1(3)
C(22)-W(1)-N(1)	148.0(3)	O(8)-Na(1)-O(7)	87.0(3)
C(21)-W(1)-P(1)	99.3(2)	O(4)-Na(1)-O(7)	105.4(2)
C(22)-W(1)-P(1)	94.0(2)	O(3)-Na(1)-O(7)	64.8(2)
N(1)-W(1)-P(1)	80.25(18)	O(5)-Na(1)-O(7)	124.9(2)

O(2)-Na(1)-O(6)	144.6(2)	C(28)-O(6)-C(29)	111.8(6)
O(8)-Na(1)-O(6)	86.0(2)	C(28)-O(6)-Na(1)	112.9(4)
O(4)-Na(1)-O(6)	110.6(2)	C(29)-O(6)-Na(1)	114.8(5)
O(3)-Na(1)-O(6)	127.1(2)	C(31)-O(7)-C(30)	114.6(7)
O(5)-Na(1)-O(6)	66.4(2)	C(31)-O(7)-Na(1)	102.7(5)
O(7)-Na(1)-O(6)	65.1(2)	C(30)-O(7)-Na(1)	107.0(5)
O(2)-Na(1)-C(33)	80.0(3)	C(33)-O(8)-C(36)	106.0(8)
O(8)-Na(1)-C(33)	24.3(3)	C(33)-O(8)-Na(1)	110.0(6)
O(4)-Na(1)-C(33)	153.6(3)	C(36)-O(8)-Na(1)	123.9(6)
O(3)-Na(1)-C(33)	131.6(3)	C(1)-N(1)-C(11)	109.2(6)
O(5)-Na(1)-C(33)	89.9(3)	C(1)-N(1)-W(1)	123.3(5)
O(7)-Na(1)-C(33)	100.0(3)	C(11)-N(1)-W(1)	123.4(5)
O(6)-Na(1)-C(33)	73.5(3)	N(1)-C(1)-C(2)	114.1(7)
O(2)-Na(1)-C(25)	72.2(2)	C(1)-C(2)-P(1)	112.1(6)
O(8)-Na(1)-C(25)	144.8(3)	C(4)-C(3)-C(5)	109.2(8)
O(4)-Na(1)-C(25)	26.1(2)	C(4)-C(3)-C(6)	110.4(7)
O(3)-Na(1)-C(25)	91.4(2)	C(5)-C(3)-C(6)	107.4(7)
O(5)-Na(1)-C(25)	48.4(2)	C(4)-C(3)-P(1)	110.0(6)
O(7)-Na(1)-C(25)	128.3(3)	C(5)-C(3)-P(1)	105.2(6)
O(6)-Na(1)-C(25)	106.4(2)	C(6)-C(3)-P(1)	114.3(6)
C(33)-Na(1)-C(25)	127.6(3)	C(8)-C(7)-C(10)	110.3(7)
C(22)-O(2)-Na(1)	153.4(6)	C(8)-C(7)-C(9)	107.4(8)
C(32)-O(3)-C(23)	113.4(7)	C(10)-C(7)-C(9)	107.0(7)
C(32)-O(3)-Na(1)	117.2(5)	C(8)-C(7)-P(1)	115.8(7)
C(23)-O(3)-Na(1)	114.9(5)	C(10)-C(7)-P(1)	110.0(6)
C(25)-O(4)-C(24)	111.8(6)	C(9)-C(7)-P(1)	105.9(6)
C(25)-O(4)-Na(1)	105.1(5)	N(1)-C(11)-C(12)	111.6(6)
C(24)-O(4)-Na(1)	107.4(5)	C(11)-C(12)-P(2)	109.8(5)
C(27)-O(5)-C(26)	114.6(6)	C(14)-C(13)-C(16)	108.2(6)
C(27)-O(5)-Na(1)	115.1(5)	C(14)-C(13)-C(15)	108.6(7)
C(26)-O(5)-Na(1)	110.9(5)	C(16)-C(13)-C(15)	108.3(7)

C(14)-C(13)-P(2)	110.0(5)	C(26)-C(25)-Na(1)	82.2(5)
C(16)-C(13)-P(2)	105.2(5)	O(5)-C(26)-C(25)	108.3(7)
C(15)-C(13)-P(2)	116.2(6)	O(5)-C(27)-C(28)	112.1(7)
C(18)-C(17)-C(19)	108.9(7)	O(6)-C(28)-C(27)	105.9(6)
C(18)-C(17)-C(20)	107.9(7)	O(6)-C(29)-C(30)	107.5(6)
C(19)-C(17)-C(20)	108.3(7)	O(7)-C(30)-C(29)	103.3(7)
C(18)-C(17)-P(2)	114.7(6)	O(7)-C(31)-C(32)	108.1(8)
C(19)-C(17)-P(2)	110.8(5)	O(3)-C(32)-C(31)	108.9(8)
C(20)-C(17)-P(2)	106.0(5)	O(8)-C(33)-C(34)	111.1(9)
O(1)-C(21)-W(1)	175.4(7)	O(8)-C(33)-Na(1)	45.7(4)
O(2)-C(22)-W(1)	176.7(7)	C(34)-C(33)-Na(1)	148.8(8)
O(3)-C(23)-C(24)	108.3(7)	C(33)-C(34)-C(35)	101.5(9)
O(4)-C(24)-C(23)	107.8(7)	C(36)-C(35)-C(34)	103.2(9)
O(4)-C(25)-C(26)	106.6(7)	O(8)-C(36)-C(35)	107.1(9)
O(4)-C(25)-Na(1)	48.8(4)		

Symmetry transformations used to generate equivalent atoms:

Table S20 Torsion angles [°] for 7.

C(11)-N(1)-C(1)-C(2)	170.1(8)	C(7)-P(1)-C(3)-C(6)	-45.5(7)
W(1)-N(1)-C(1)-C(2)	-32.0(11)	W(1)-P(1)-C(3)-C(6)	175.3(5)
N(1)-C(1)-C(2)-P(1)	30.6(10)	C(2)-P(1)-C(7)-C(8)	-41.6(7)
C(3)-P(1)-C(2)-C(1)	108.0(7)	C(3)-P(1)-C(7)-C(8)	68.5(7)
C(7)-P(1)-C(2)-C(1)	-138.7(7)	W(1)-P(1)-C(7)-C(8)	-151.1(6)
W(1)-P(1)-C(2)-C(1)	-17.8(7)	C(2)-P(1)-C(7)-C(10)	-167.4(6)
C(2)-P(1)-C(3)-C(4)	-173.7(6)	C(3)-P(1)-C(7)-C(10)	-57.3(7)
C(7)-P(1)-C(3)-C(4)	79.4(7)	W(1)-P(1)-C(7)-C(10)	83.1(6)
W(1)-P(1)-C(3)-C(4)	-59.8(7)	C(2)-P(1)-C(7)-C(9)	77.3(6)
C(2)-P(1)-C(3)-C(5)	-56.2(7)	C(3)-P(1)-C(7)-C(9)	-172.7(6)
C(7)-P(1)-C(3)-C(5)	-163.1(6)	W(1)-P(1)-C(7)-C(9)	-32.2(6)
W(1)-P(1)-C(3)-C(5)	57.7(7)	C(1)-N(1)-C(11)-C(12)	-160.8(7)
C(2)-P(1)-C(3)-C(6)	61.3(7)	W(1)-N(1)-C(11)-C(12)	41.3(9)

N(1)-C(11)-C(12)-P(2)	-38.8(8)	C(24)-O(4)-C(25)-Na(1)	116.2(6)
C(17)-P(2)-C(12)-C(11)	142.4(6)	C(27)-O(5)-C(26)-C(25)	167.6(7)
C(13)-P(2)-C(12)-C(11)	-103.0(6)	Na(1)-O(5)-C(26)-C(25)	35.1(8)
W(1)-P(2)-C(12)-C(11)	22.8(6)	O(4)-C(25)-C(26)-O(5)	-68.2(8)
C(12)-P(2)-C(13)-C(14)	175.1(6)	Na(1)-C(25)-C(26)-O(5)	-25.5(6)
C(17)-P(2)-C(13)-C(14)	-76.7(6)	C(26)-O(5)-C(27)-C(28)	-90.7(8)
W(1)-P(2)-C(13)-C(14)	61.8(6)	Na(1)-O(5)-C(27)-C(28)	39.8(8)
C(12)-P(2)-C(13)-C(16)	58.8(6)	C(29)-O(6)-C(28)-C(27)	-180.0(7)
C(17)-P(2)-C(13)-C(16)	167.0(5)	Na(1)-O(6)-C(28)-C(27)	48.7(7)
W(1)-P(2)-C(13)-C(16)	-54.5(6)	O(5)-C(27)-C(28)-O(6)	-58.8(9)
C(12)-P(2)-C(13)-C(15)	-61.1(7)	C(28)-O(6)-C(29)-C(30)	-165.8(7)
C(17)-P(2)-C(13)-C(15)	47.1(7)	Na(1)-O(6)-C(29)-C(30)	-35.5(8)
W(1)-P(2)-C(13)-C(15)	-174.4(5)	C(31)-O(7)-C(30)-C(29)	-179.4(7)
C(12)-P(2)-C(17)-C(18)	49.9(7)	Na(1)-O(7)-C(30)-C(29)	-66.3(6)
C(13)-P(2)-C(17)-C(18)	-59.0(7)	O(6)-C(29)-C(30)-O(7)	67.6(8)
W(1)-P(2)-C(17)-C(18)	160.1(5)	C(30)-O(7)-C(31)-C(32)	-176.7(7)
C(12)-P(2)-C(17)-C(19)	173.7(6)	Na(1)-O(7)-C(31)-C(32)	67.6(8)
C(13)-P(2)-C(17)-C(19)	64.7(6)	C(23)-O(3)-C(32)-C(31)	150.6(8)
W(1)-P(2)-C(17)-C(19)	-76.1(6)	Na(1)-O(3)-C(32)-C(31)	13.0(10)
C(12)-P(2)-C(17)-C(20)	-69.0(6)	O(7)-C(31)-C(32)-O(3)	-56.0(11)
C(13)-P(2)-C(17)-C(20)	-178.0(5)	C(36)-O(8)-C(33)-C(34)	18.4(13)
W(1)-P(2)-C(17)-C(20)	41.2(6)	Na(1)-O(8)-C(33)-C(34)	154.7(8)
C(32)-O(3)-C(23)-C(24)	-167.3(7)	C(36)-O(8)-C(33)-Na(1)	-136.2(10)
Na(1)-O(3)-C(23)-C(24)	-28.7(8)	O(8)-C(33)-C(34)-C(35)	1.6(13)
C(25)-O(4)-C(24)-C(23)	-176.1(7)	Na(1)-C(33)-C(34)-C(35)	37.8(19)
Na(1)-O(4)-C(24)-C(23)	-61.3(7)	C(33)-C(34)-C(35)-C(36)	-20.5(13)
O(3)-C(23)-C(24)-O(4)	60.3(8)	C(33)-O(8)-C(36)-C(35)	-32.7(14)
C(24)-O(4)-C(25)-C(26)	179.5(7)	Na(1)-O(8)-C(36)-C(35)	-161.1(8)
Na(1)-O(4)-C(25)-C(26)	63.3(7)	C(34)-C(35)-C(36)-O(8)	32.8(14)

Symmetry transformations used to generate equivalent atoms:

3.7 Crystal Structure of **8**

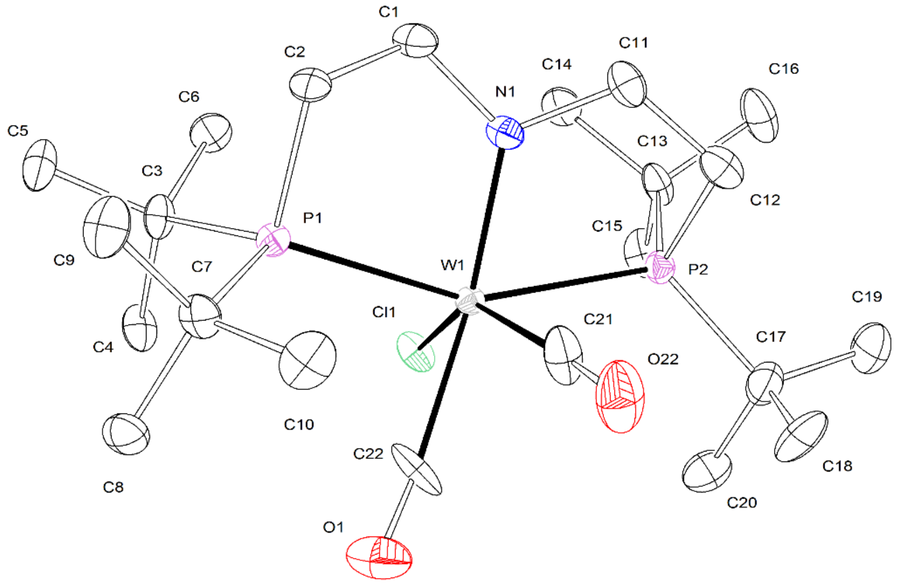

Figure S56 Thermal ellipsoid plot of **8** with the anisotropic displacement parameters drawn at the 50 % probability level. The asymmetric unit contains one complex molecule. The structure was refined as an inversion twin using the twin law -100 0-10 00-1 (BASF: 0.009(10)).

Table S21 Crystal data and structure refinement for 8.

Identification code	CCDC-2069384	
Empirical formula	$C_{22}H_{44}ClNO_2P_2W$	
Formula weight	635.82	
Temperature	100(2) K	
Wavelength	0.71073 Å	
Crystal system	Monoclinic	
Space group	$P2_1$	
Unit cell dimensions	a = 8.1299(2) Å	α = 90°
	b = 13.7862(4) Å	β = 101.851(2)°
	c = 11.9710(4) Å	γ = 90°
Volume	1313.12(7) $Å^3$	
Z	2	
Density (calculated)	1.608 Mg/m^3	
Absorption coefficient	4.640 mm^{-1}	
F(000)	640	
Crystal size	0.253 x 0.106 x 0.059 mm^3	
Crystal shape and color	Plate, intense purple	
Theta range for data collection	2.281 to 28.365°.	
Index ranges	-10<=h<=10, -18<=k<=18, -16<=l<=15	
Reflections collected	32990	
Independent reflections	6541 [R(int) = 0.0733]	

Completeness to theta = 25.242°	100.0 %
Refinement method	Full-matrix least-squares on F^2
Data / restraints / parameters	6541 / 1 / 275
Goodness-of-fit on F2	1.047
Final R indices [I>2sigma(I)]	R1 = 0.0312, wR2 = 0.0584
R indices (all data)	R1 = 0.0403, wR2 = 0.0606
Absolute structure parameter	0.009(10)
Largest diff. peak and hole	1.417 and -1.378 eÅ^{-3}

Table S22 Bond lengths [Å] and angles [°] for 8.

W(1)-C(21)	1.939(8)	C(17)-C(18)	1.546(17)
W(1)-N(1)	2.013(6)		
W(1)-C(22)	2.056(8)	C(21)-W(1)-N(1)	89.2(3)
W(1)-Cl(1)	2.4682(19)	C(21)-W(1)-C(22)	73.0(3)
W(1)-P(2)	2.516(2)	N(1)-W(1)-C(22)	152.9(3)
W(1)-P(1)	2.5175(19)	C(21)-W(1)-Cl(1)	143.0(3)
P(1)-C(2)	1.845(7)	N(1)-W(1)-Cl(1)	122.32(18)
P(1)-C(3)	1.891(10)	C(22)-W(1)-Cl(1)	82.3(2)
P(1)-C(7)	1.892(7)	C(21)-W(1)-P(2)	84.2(2)
P(2)-C(12)	1.832(9)	N(1)-W(1)-P(2)	77.02(18)
P(2)-C(17)	1.888(9)	C(22)-W(1)-P(2)	119.8(2)
P(2)-C(13)	1.901(8)	Cl(1)-W(1)-P(2)	84.91(6)
O(1)-C(22)	1.104(9)	C(21)-W(1)-P(1)	102.6(3)
O(22)-C(21)	1.159(10)	N(1)-W(1)-P(1)	79.28(18)
N(1)-C(1)	1.469(10)	C(22)-W(1)-P(1)	84.8(2)
N(1)-C(11)	1.486(10)	Cl(1)-W(1)-P(1)	101.96(6)
C(1)-C(2)	1.515(11)	P(2)-W(1)-P(1)	155.24(6)
C(3)-C(4)	1.527(16)	C(2)-P(1)-C(3)	104.9(4)
C(3)-C(6)	1.532(10)	C(2)-P(1)-C(7)	102.3(4)
C(3)-C(5)	1.546(12)	C(3)-P(1)-C(7)	110.1(4)
C(7)-C(9)	1.526(11)	C(2)-P(1)-W(1)	99.6(2)
C(7)-C(10)	1.529(11)	C(3)-P(1)-W(1)	115.7(4)
C(7)-C(8)	1.547(11)	C(7)-P(1)-W(1)	121.2(2)
C(11)-C(12)	1.507(12)	C(12)-P(2)-C(17)	106.6(4)
C(13)-C(15)	1.530(12)	C(12)-P(2)-C(13)	103.8(4)
C(13)-C(16)	1.542(11)	C(17)-P(2)-C(13)	108.4(4)
C(13)-C(14)	1.547(11)	C(12)-P(2)-W(1)	96.1(3)
C(17)-C(20)	1.520(13)	C(17)-P(2)-W(1)	121.2(3)
C(17)-C(19)	1.528(12)	C(13)-P(2)-W(1)	117.7(3)

C(1)-N(1)-C(11)	110.8(6)	N(1)-C(11)-C(12)	114.4(7)
C(1)-N(1)-W(1)	124.9(5)	C(11)-C(12)-P(2)	110.0(6)
C(11)-N(1)-W(1)	124.1(5)	C(15)-C(13)-C(16)	109.0(7)
N(1)-C(1)-C(2)	111.3(6)	C(15)-C(13)-C(14)	108.2(7)
C(1)-C(2)-P(1)	109.9(5)	C(16)-C(13)-C(14)	107.1(7)
C(4)-C(3)-C(6)	109.1(11)	C(15)-C(13)-P(2)	112.5(6)
C(4)-C(3)-C(5)	109.8(6)	C(16)-C(13)-P(2)	111.8(6)
C(6)-C(3)-C(5)	107.4(6)	C(14)-C(13)-P(2)	107.9(5)
C(4)-C(3)-P(1)	109.6(5)	C(20)-C(17)-C(19)	110.6(8)
C(6)-C(3)-P(1)	106.6(5)	C(20)-C(17)-C(18)	108.4(8)
C(5)-C(3)-P(1)	114.2(10)	C(19)-C(17)-C(18)	106.9(7)
C(9)-C(7)-C(10)	107.6(7)	C(20)-C(17)-P(2)	107.9(6)
C(9)-C(7)-C(8)	108.2(6)	C(19)-C(17)-P(2)	115.1(6)
C(10)-C(7)-C(8)	107.8(7)	C(18)-C(17)-P(2)	107.9(7)
C(9)-C(7)-P(1)	113.0(5)	O(22)-C(21)-W(1)	176.5(8)
C(10)-C(7)-P(1)	108.9(5)	O(1)-C(22)-W(1)	172.1(7)
C(8)-C(7)-P(1)	111.2(5)		

Symmetry transformations used to generate equivalent atoms:

Table S23 Torsion angles [°] for 8.

C(11)-N(1)-C(1)-C(2)	-134.1(7)	W(1)-P(1)-C(3)-C(5)	-173.5(5)
W(1)-N(1)-C(1)-C(2)	41.6(9)	C(2)-P(1)-C(7)-C(9)	40.9(6)
N(1)-C(1)-C(2)-P(1)	-41.7(8)	C(3)-P(1)-C(7)-C(9)	-70.2(7)
C(3)-P(1)-C(2)-C(1)	-93.6(6)	W(1)-P(1)-C(7)-C(9)	150.3(5)
C(7)-P(1)-C(2)-C(1)	151.5(5)	C(2)-P(1)-C(7)-C(10)	-78.6(6)
W(1)-P(1)-C(2)-C(1)	26.4(6)	C(3)-P(1)-C(7)-C(10)	170.3(6)
C(2)-P(1)-C(3)-C(4)	171.5(6)	W(1)-P(1)-C(7)-C(10)	30.8(7)
C(7)-P(1)-C(3)-C(4)	-79.1(8)	C(2)-P(1)-C(7)-C(8)	162.8(5)
W(1)-P(1)-C(3)-C(4)	62.8(6)	C(3)-P(1)-C(7)-C(8)	51.7(7)
C(2)-P(1)-C(3)-C(6)	53.5(8)	W(1)-P(1)-C(7)-C(8)	-87.8(5)
C(7)-P(1)-C(3)-C(6)	162.9(7)	C(1)-N(1)-C(11)-C(12)	-172.1(7)
W(1)-P(1)-C(3)-C(6)	-55.2(8)	W(1)-N(1)-C(11)-C(12)	12.2(9)
C(2)-P(1)-C(3)-C(5)	-64.8(6)	N(1)-C(11)-C(12)-P(2)	24.4(8)
C(7)-P(1)-C(3)-C(5)	44.6(7)	C(17)-P(2)-C(12)-C(11)	-164.3(6)

C(13)-P(2)-C(12)-C(11)	81.4(6)	C(13)-P(2)-C(17)-C(19)	42.6(8)
W(1)-P(2)-C(12)-C(11)	-39.1(6)	W(1)-P(2)-C(17)-C(19)	-176.6(6)
C(12)-P(2)-C(17)-C(20)	167.5(6)	C(12)-P(2)-C(17)-C(18)	50.6(7)
C(13)-P(2)-C(17)-C(20)	-81.3(6)	C(13)-P(2)-C(17)-C(18)	161.8(6)
W(1)-P(2)-C(17)-C(20)	59.5(7)	W(1)-P(2)-C(17)-C(18)	-57.4(7)
C(12)-P(2)-C(17)-C(19)	-68.5(8)		

Symmetry transformations used to generate equivalent atoms:

4. Computational Details

4.1 Computational details

All calculations used the ORCA 4.1.2 program suite.[9,10] The optimizations were carried out using the PBE[11] functional. Ahlrich's split-valence basis set including polarization functions, def2-SVP, and the def2/J auxiliary basis set were used on all atoms except the heteroatoms (W, P, N, O), for which Ahlrich's def2-TZVP with the def2/J auxiliary basis set were chosen. For W, the SD (60,MWB) effective core potential replacing 60 core electrons was used.[12,13,14] The CPCM continuum solvation model for THF, Grimme's dispersion correction with Becke-Johnson damping (D3BJ)[15,16] and the Resolution of Identity (RI-J) approximation[17,18] to minimize the computational costs were applied. To evaluate the thermal path of N_2 splitting, the structures were optimized using the 'tight' convergence criteria and the grid size was increased to 7 in the ORCA nomenclature. The integration accuracy was set to 7.0. For **3** the optimizations started from the crystal structure, while the conformations **3'** and **3''** started from the optimized geometries of **3** and were modified concerning the pincer conformation. To verify the geometries thus found were local minima or transition states structures and to obtain thermodynamic data, numerical frequencies were computed at the same level of theory as the geometry optimizations. For the thermodynamic data, several density functionals and different basis set sizes for selected functionals were tested using otherwise the same settings as in the geometry optimizations. Depending on the functional different dispersion corrections were used, the details are listed in the appropriate sections. For these variations only the electronic energies of the tested functionals were used, while the thermodynamic quantities are all based on the PBE calculations for each structure. The results of a subset of these tests are shown in sections 4.4.1 and 4.4.2, respectively. No other functional or larger basis set resulted in a better agreement with the experiment.

To predict the electronic excitation spectra, time-dependent DFT with the PBE0[19] functional was conducted with the chain of spheres approximation[20] otherwise using the same settings as in the geometry optimizations. The Tamm-Dancoff approximation[21] was employed and 40 excitations were computed.

4.2 Geometries and Mayer bond orders

Table S24 Key geometric parameters of the optimized geometries.

Distances (Å)	^{3}**3**	^{1}**3**	Distances (Å)	^{3}**3'**	^{1}**3'**	^{3}**3''**	^{1}**3''**
W0-N1	1.885	1.889	W0-N1	1.889	1.884	1.941	1.936
W3-N2	1.885	1.889	W3-N2	1.888	1.884	1.921	1.936
N1-N2	1.206	1.208	N1-N2	1.211	1.214	1.205	1.202
W0-N9	2.072	2.078	W0-N75	2.077	2.078	2.094	2.086
W3-N4	2.072	2.078	W3-N8	2.076	2.077	2.091	2.086
W0-P13	2.464	2.456	W0-P76	2.490	2.493	2.484	2.466
W0-P10	2.499	2.526	W0-P77	2.496	2.494	2.501	2.516
W3-P5	2.499	2.525	W3-P10	2.495	2.493	2.507	2.516
W3-P8	2.464	2.456	W3-P9	2.489	2.493	2.491	2.466
W0-C11	1.960	1.939	W0-C6	1.957	1.955	1.916	1.899
W3-C6	1.960	1.939	W3-C4	1.958	1.956	1.935	1.899
Angles (°)			Angles (°)				
W0-N1-N2	168.56	164.96	W0-N1-N2	176.36	176.54	174.49	174.56
W3-N2-N1	168.59	164.97	W3-N2-N1	175.71	176.21	176.18	174.60

C11-W0-N1	96.62	96.62	C6-W0-N1	95.75	96.68	96.312	95.26
C6-W3-N2	96.59	96.62	C4-W3-N2	95.83	96.69	93.66	95.18
P13-W0-P10	154.60	153.41	P76-W0-P77	151.97	151.69	156.53	154.45
P5-W3-P8	154.56	153.39	P10-W3-P9	151.56	151.48	154.60	154.42
N9-W0-N1	119.83	122.92	N75-W0-N1	122.28	122.156	149.85	152.234
N4-W3-N2	119.73	122.90	N8-W3-N2	121.66	121.77	136.33	152.32
N9-W0-C11	143.33	138.99	N75-W0-C6	141.89	141.13	113.47	111.58
N4-W3-C6	143.47	139.01	N8-W3-C4	142.412	141.50	129.91	111.57

Table S25 Computed Mayer bond orders of the optimized geometries.

B.O.	^{3}**3**	^{1}**3**	B.O.	^{3}**3’**	^{1}**3’**	^{3}**3”**	^{1}**3”**
W0-W3	0.2055	0.2344	W0-W3	0.2242	0.2444	0.2196	<0.1000
W0-N1	1.4283	1.3793	W0-N1	1.4108	1.6745	1.2615	1.1156
W0-N2	0.1280	0.1283	W0-N2	0.1368	0.1203	0.1528	0.1774
W0-C11	1.6625	1.6699	W0-C6	1.6876	1.6501	1.8119	1.6082
W0-N9	0.9180	0.8856	W0-N75	0.8750	0.8251	0.8859	0.7640
W0-P13	0.6827	0.7176	W0-P76	0.6346	0.5725	0.6683	0.5111
W0-P10	0.6036	0.5914	W0-P77	0.6292	0.6737	0.6406	0.5677
N1-N2	1.0877	1.2097	N1-N2	1.0975	0.8795	1.3011	1.1681
W3-N1	0.1281	0.1281	W3-N1	0.1367	0.1194	0.1621	<0.1000
W3-N2	1.4286	1.3793	W3-N2	1.4114	1.6701	1.3107	0.9393
W3-C6	1.6635	1.6725	W3-C4	1.6835	1.6586	1.7492	2.0924
W3-N4	0.9180	0.8852	W3-N8	0.8739	0.8280	0.8791	0.7152
W3-P5	0.6038	0.5914	W3-P10	0.6299	0.6729	0.6185	0.5348
W3-P8	0.6827	0.7172	W3-P9	0.6353	0.5718	0.6525	0.6185

4.3 Computed UV-vis spectra

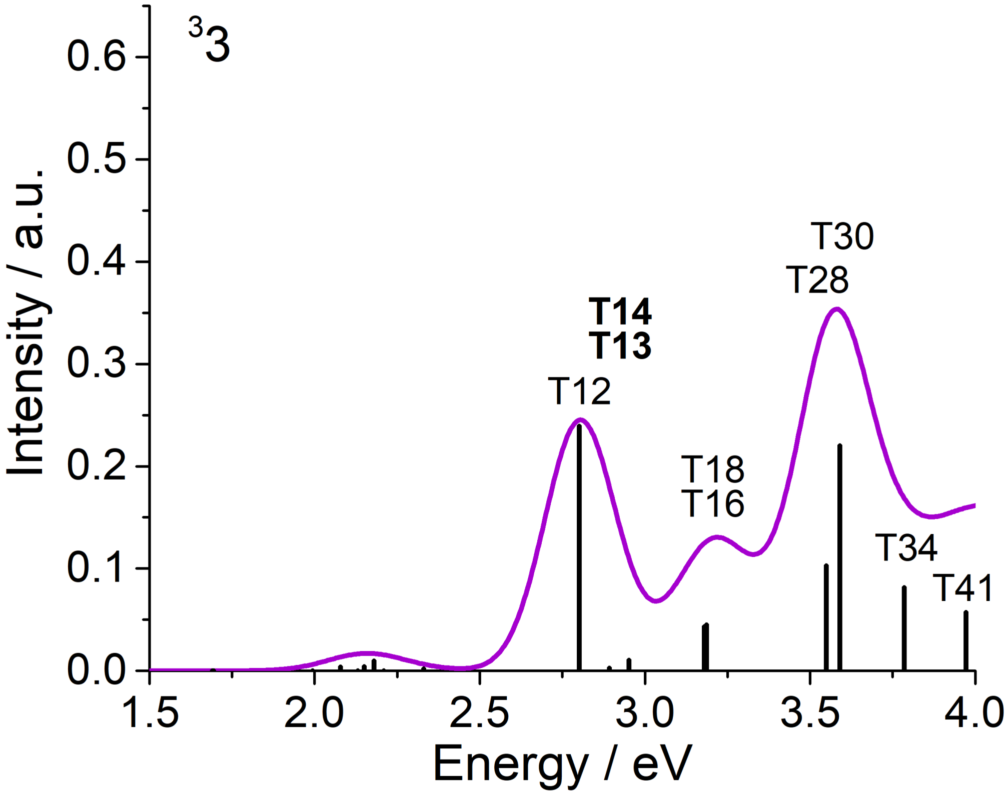

Figure S57 Predicted UV-vis spectrum (PBE0) of $^{3}3$.

Table S26 Energies, oscillator strengths and difference densities of the labelled transitions in the spectrum of $^{3}3$.

State	*E* (eV)	*E* (nm)	f_{osc}	
**12**	2.80	442.8	0.2391	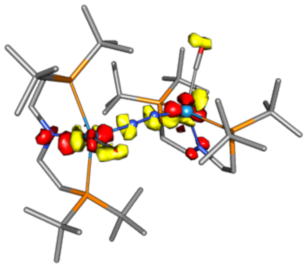

**13**	2.89	429.0	0.0003	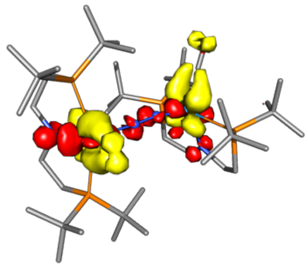
**14**	2.89	429.0	0.0028	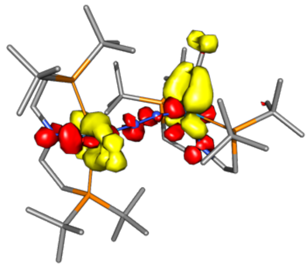
**16**	3.18	389.9	0.0430	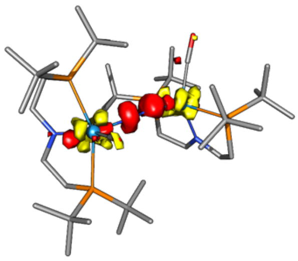
**18**	3.19	388.7	0.0451	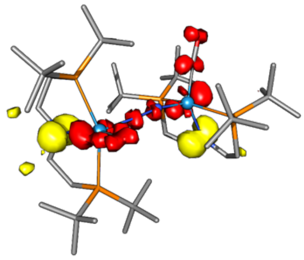
**28**	3.55	349.3	0.1028	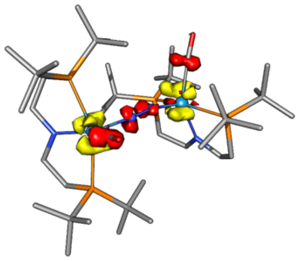

**30**	3.59	345.4	0.2199	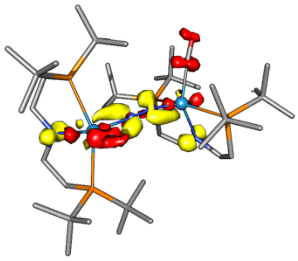
**34**	3.78	328.0	0.0814	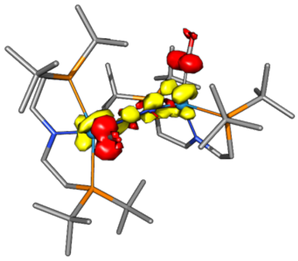
**41**	3.97	312.3	0.0571	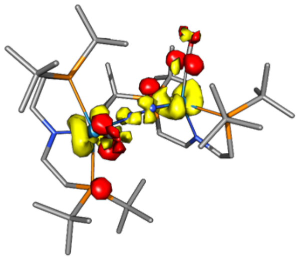

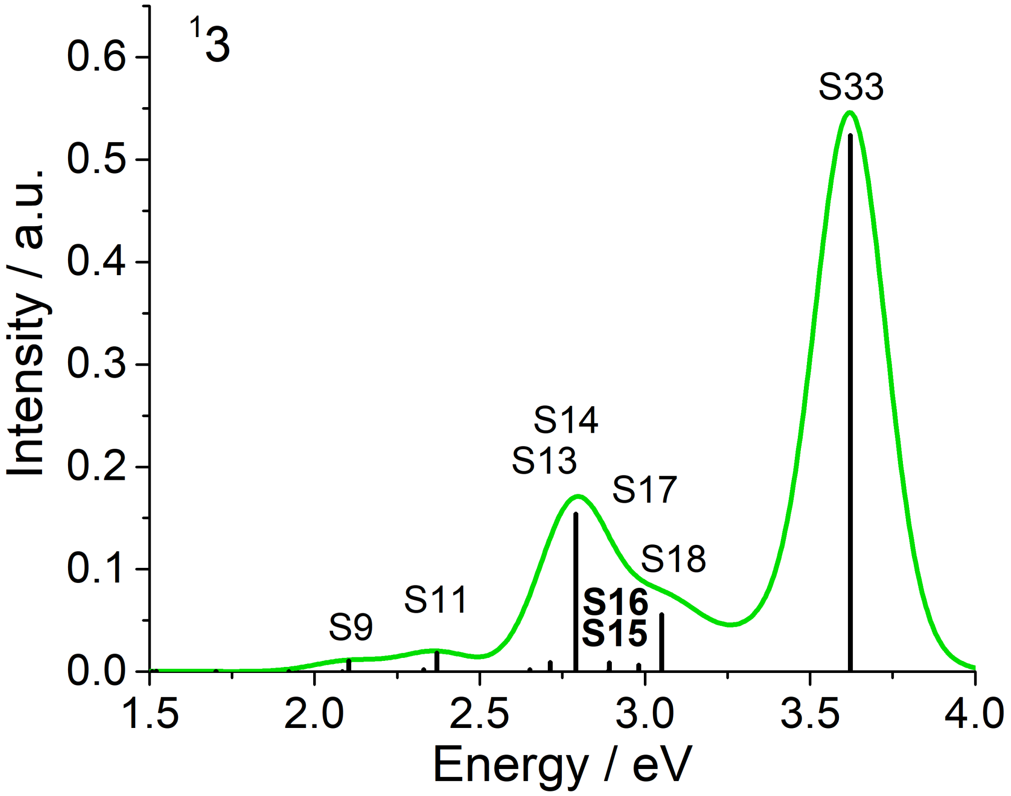

Figure S58 Predicted UV-vis spectrum (PBE0) of ^{1}3.

Table S27 Energies, oscillator strengths and difference densities of the labelled transitions in the spectrum of ^{1}3.

State	*E* (eV)	*E* (nm)	f_{osc}	
**9**	2.10	590.4	0.0104	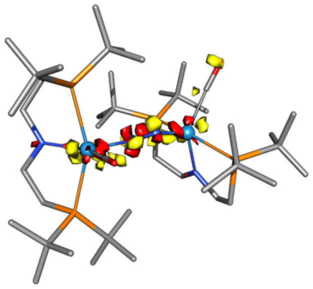

**11**	2.37	523.1	0.0179	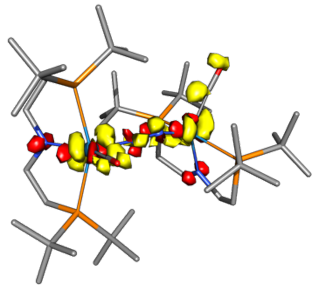
**13**	2.71	457.5	0.0089	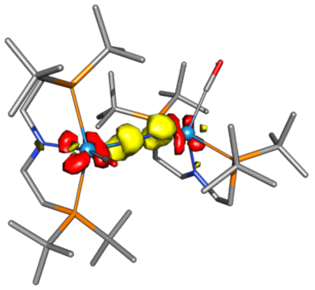
**14**	2.79	444.4	0.1538	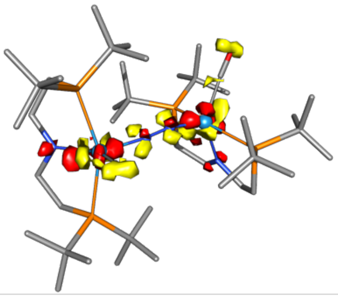
**15**	2.89	429.0	0.0001	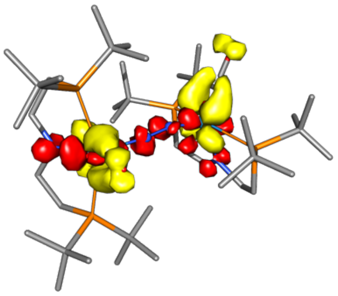
**16**	2.89	429.0	0.0085	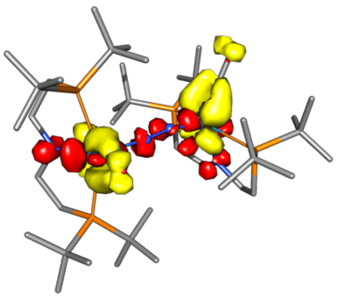

**17**	2.98	416.1	0.0065	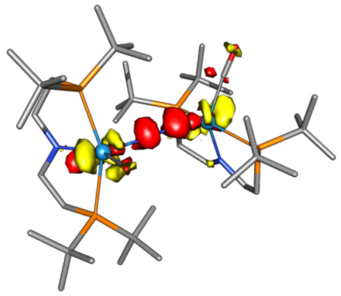
**18**	3.05	406.5	0.0555	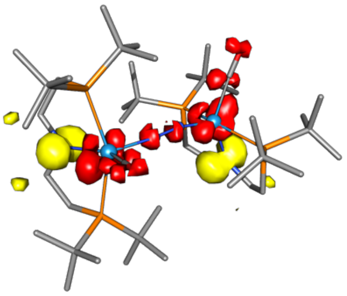
**33**	3.62	342.5	0.0052	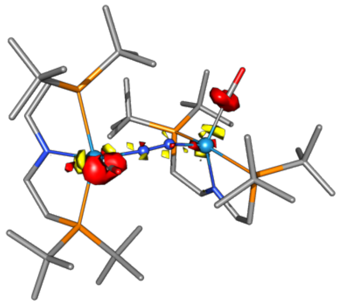

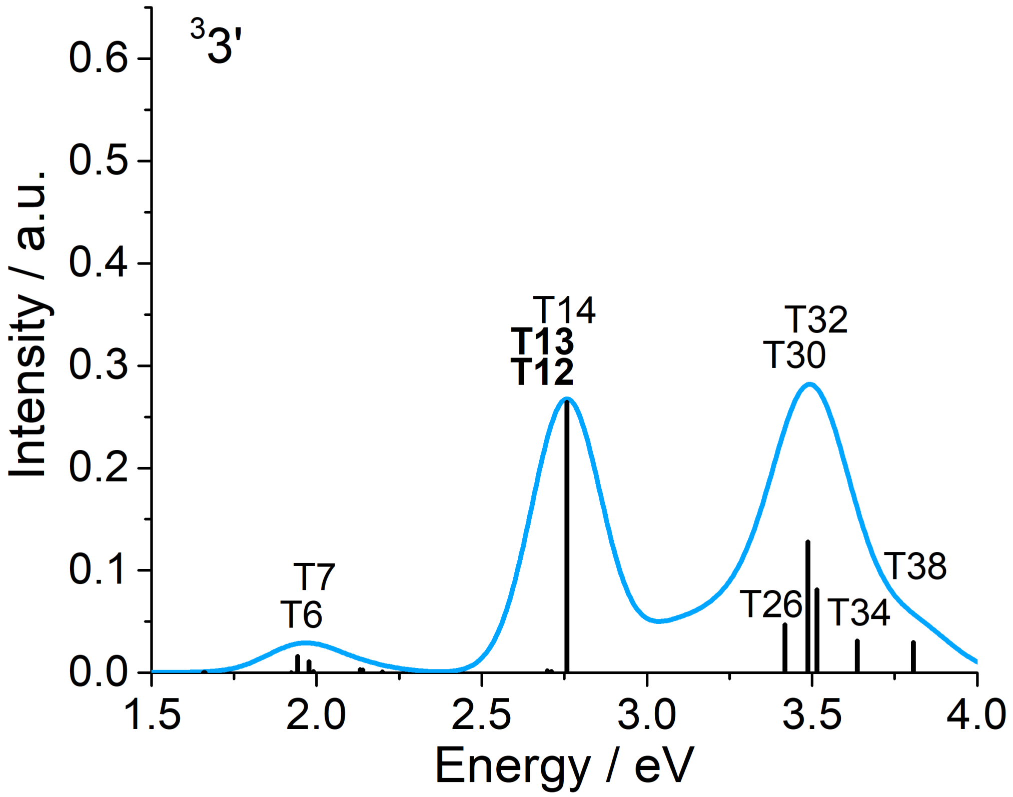

Figure S59 Predicted UV-vis spectrum (PBE0) of ^{3}3'.

Table S28 Energies, oscillator strengths and difference densities of the labelled transitions in the spectrum of ^{3}3'.

State	*E* (eV)	*E* (nm)	f_{osc}	
**6**	1.94	639.1	0.0160	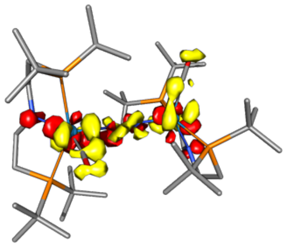
**7**	1.98	626.2	0.0109	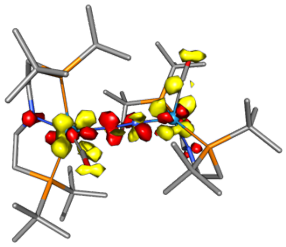

**12**	2.70	459.2	0.0018	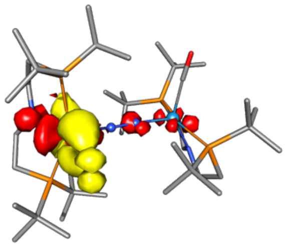
**13**	2.71	457.5	0.0013	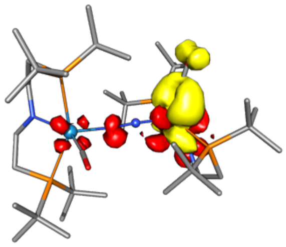
**14**	2.76	449.2	0.2642	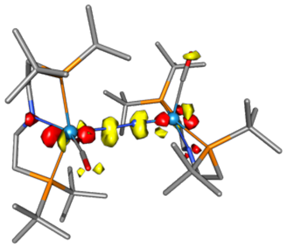
**26**	3.42	362.5	0.0469	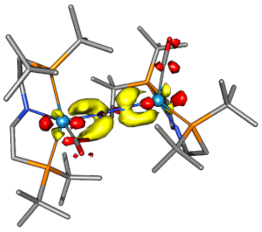
**30**	3.49	355.3	0.1276	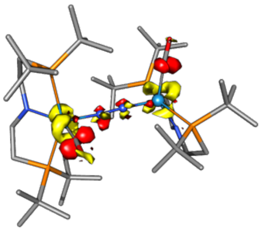
**32**	3.51	353.2	0.0811	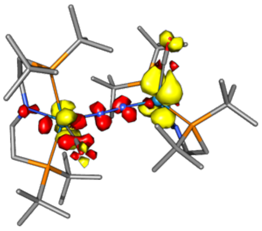
**34**	3.64	340.6	0.0309	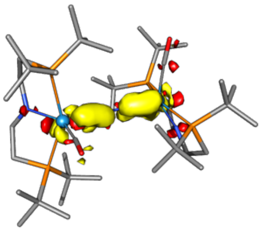

**38**	3.81	325.4	0.0296	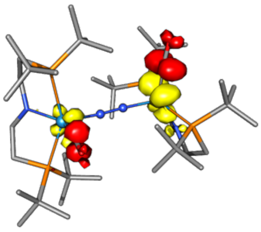

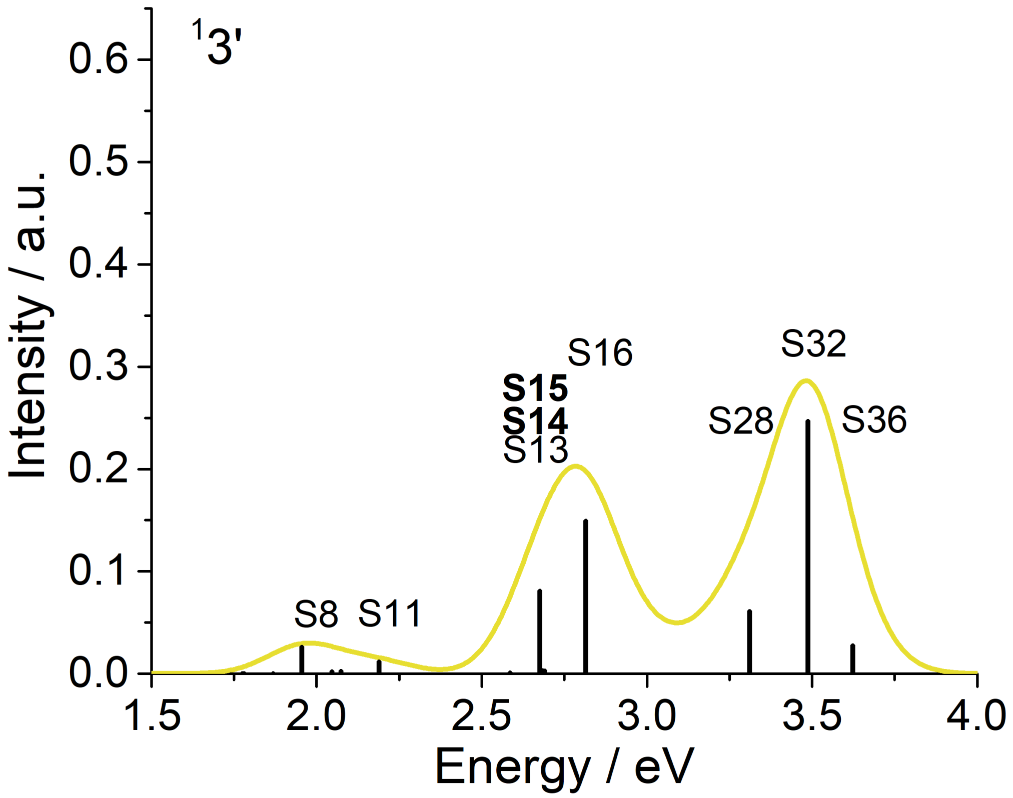

Figure S60 Predicted UV-vis spectrum (PBE0) of ¹3'.

Table S29 Energies, oscillator strengths and difference densities of the labelled transitions in the spectrum of ¹3'.

State	*E* (eV)	*E* (nm)	f_{osc}	
**8**	1.95	635.8	0.0260	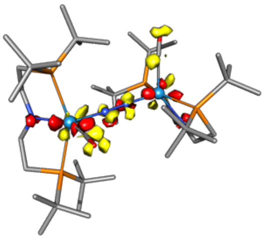

**11**	2.19	566.1	0.0114	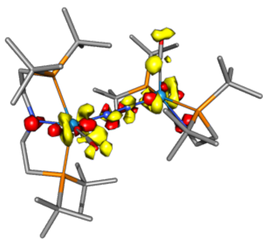
**13**	2.68	462.6	0.0807	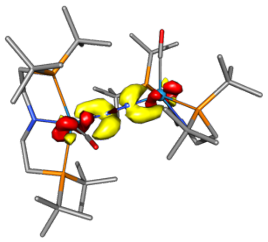
**14**	2.68	462.6	0.0031	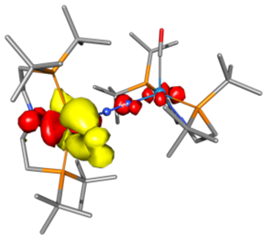
**15**	2.69	460.9	0.0026	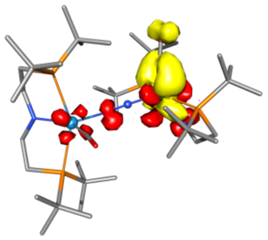
**16**	2.81	441.2	0.1490	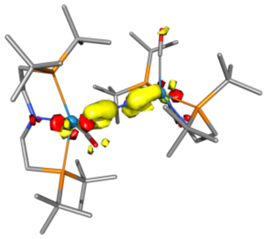
**28**	3.31	374.6	0.0609	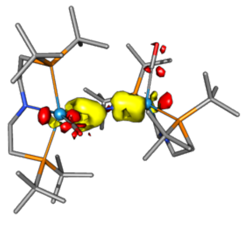
**32**	3.49	355.3	0.2464	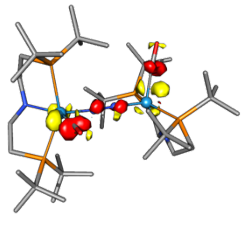

**36**	3.62	342.5	0.0272	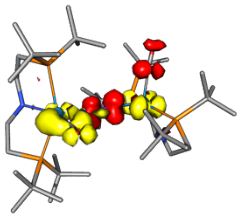

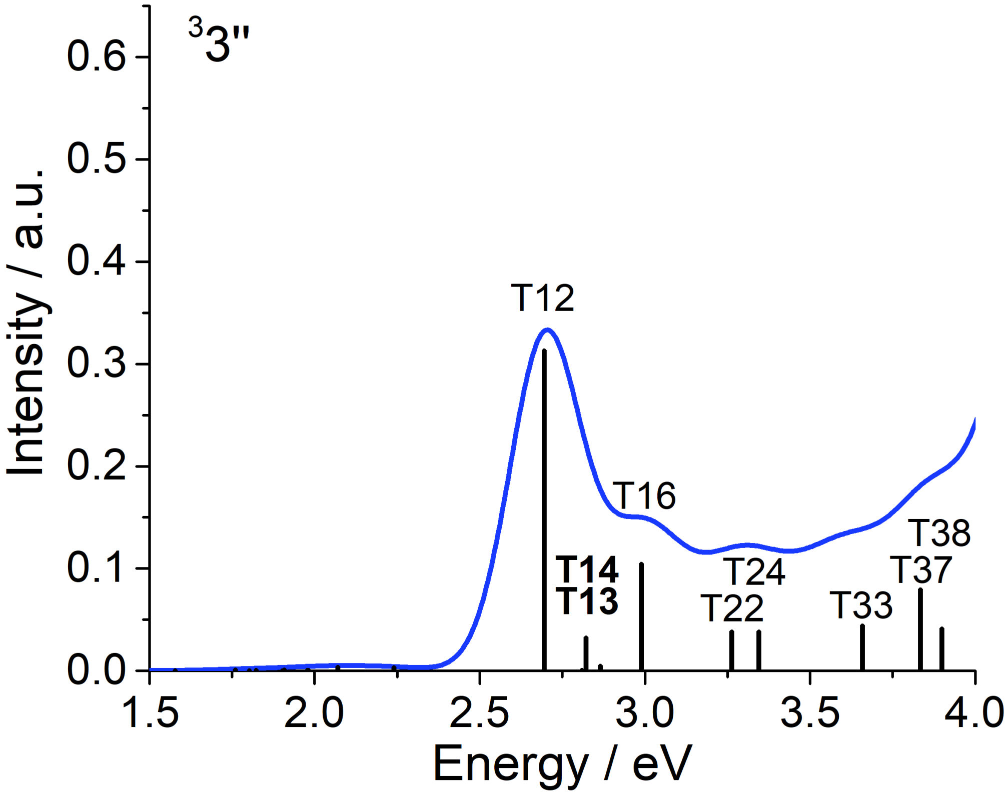

Figure S61 Predicted UV-vis spectrum (PBE0) of ^{3}3''.

Table S30 Energies, oscillator strengths and difference densities of the labelled transitions in the spectrum of ^{3}3''.

State	*E* (eV)	*E* (nm)	f_{osc}	
**12**	2.69	460.9	0.3127	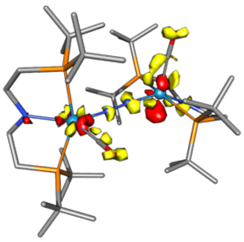

**13**	2.81	441.2	0.0004	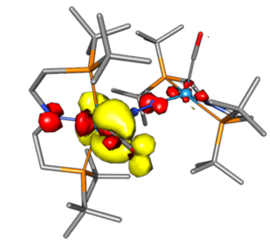
**14**	2.82	439.7	0.0321	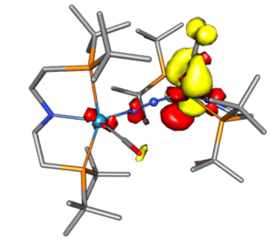
**16**	2.86	433.5	0.0044	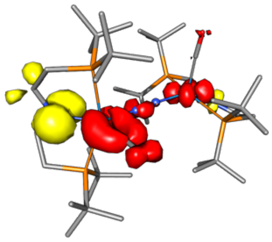
**22**	3.26	380.3	0.0381	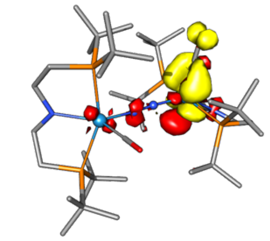
**24**	3.30	375.7	0.0381	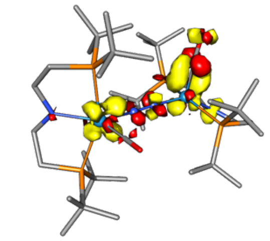
**33**	3.66	338.8	0.0440	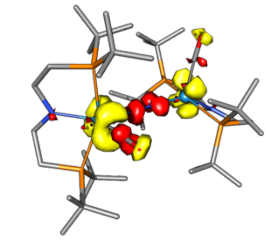
**37**	3.83	323.7	0.0791	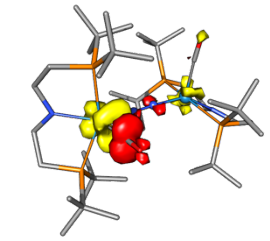

**38**	3.90	317.9	0.0409	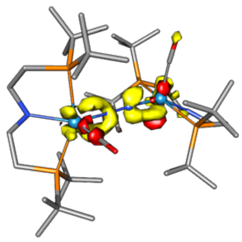

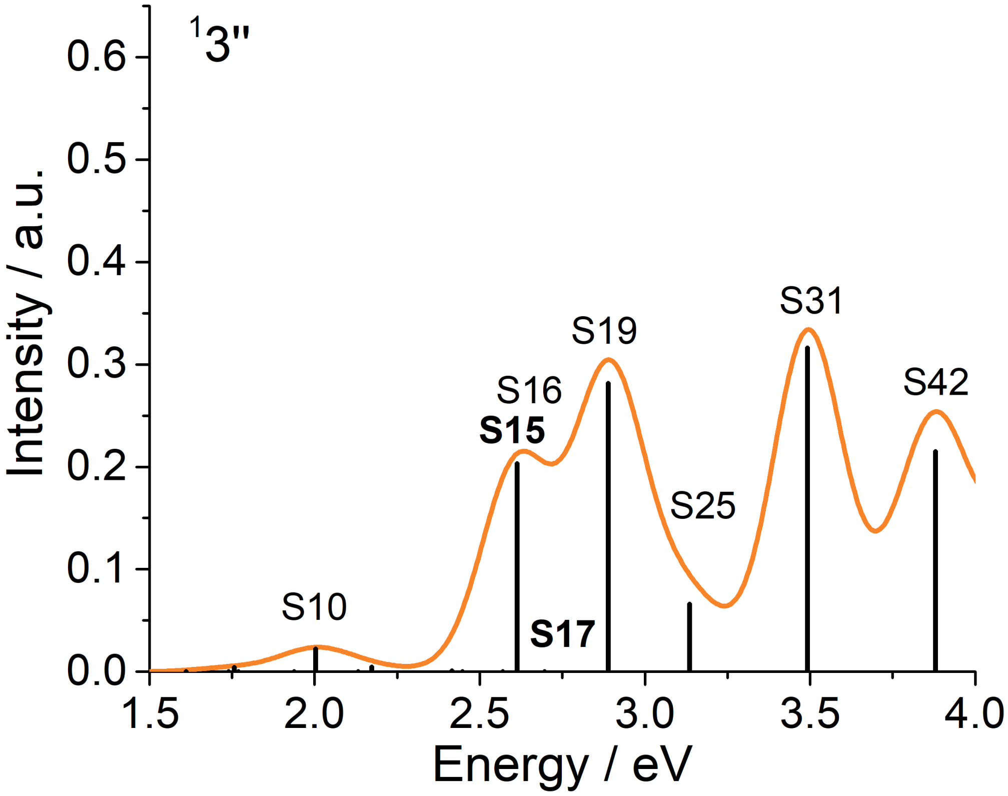

Figure S62 Predicted UV-vis spectrum (PBE0) of [1]3''.

Table S31 Energies, oscillator strengths and difference densities of the labelled transitions in the spectrum of [1]3''.

State	*E* (eV)	*E* (nm)	f_{osc}	
**10**	2.00	619.9	0.0222	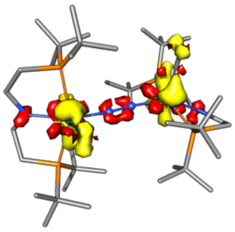

**15**	2.57	482.4	0.0000	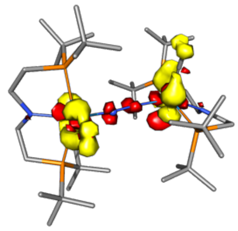
**16**	2.61	475.0	0.2032	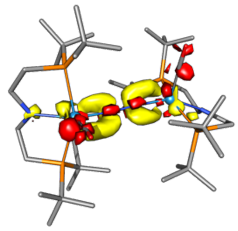
**17**	2.70	459.2	0.0000	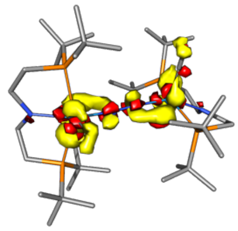
**19**	2.89	429.0	0.2817	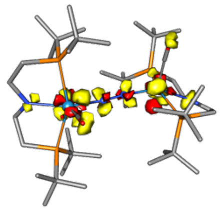
**25**	3.13	396.1	0.0658	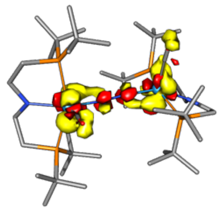
**31**	3.49	355.3	0.3160	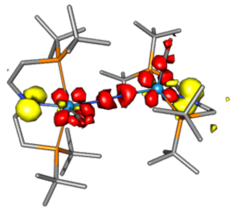
**42**	3.88	319.5	0.2147	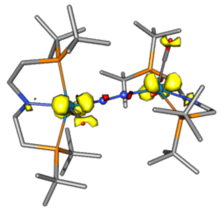

4.4 Thermochemistry data

4.4.1 Thermodynamics and kinetics for thermal dissociation of tungsten dimer for various functionals

All energies in kcal/mol

GS(^{3}**3**): ground state with triplet multiplicity

GS(^{1}**3**): ground state with closed shell singlet multiplicity

^{1}**4**: monomer 1 (same conformation of the pincer backbone as in **3**), singlet multiplicity

^{1}**4'**: monomer 2 (same conformation of the pincer backbone as in **3'**), singlet multiplicity

TS(^{3}**3**): transition state of **3** with triplet multiplicity

TS(^{1}**3**): transition state[1] of **3** with singlet multiplicity

TS(^{1}**3'**): transition state of **3'** with singlet multiplicity

Table S32 Relative electronic energies and thermodynamic data (kcal/mol) for all species potentially involved in the dissociation process computed with the density functional PBE with D3BJ (see computational details).

	$FSPE_{rel}$	ΔH	$T\Delta S$	ΔG
GS(^{3}**3**) -> TS(^{3}**3**)	61.5	59.1	0.2	58.9
GS(^{3}**3**) -> TS(^{1}**3**)	40.5	37.6	-1.9	39.5
GS(^{1}**3**) -> TS(^{1}**3**)	39.4	36.8	-1.5	38.3
GS(^{3}**3**) -> TS(^{1}**3'**)	35.7	33.6	-0.6	**34.2**
GS(^{1}**3**) -> TS(^{1}**3'**)	34.6	33.6	-0.3	33.0
GS(^{3}**3**) -> ^{1}**4**	24.1	21.8	16.5	**5.3**
GS(^{3}**3**) -> ^{1}**4'**	18.0	14.3	-25.5	**-0.7**

Table S33 Relative electronic energies and thermodynamic data (kcal/mol) for all species potentially involved in the dissociation process computed with the density functional PBE0 with D3BJ (see computational details) (no improvement).

	$FSPE_{rel}$	ΔH	T*ΔS	ΔG
GS(^{3}**3**) -> TS(^{3}**3**)	73.9	71.5	0.2	71.4
GS(^{3}**3**) -> TS(^{1}**3**)	49.7	46.9	-1.9	48.8
GS(^{1}**3**) -> TS(^{1}**3**)	47.7	45.1	-1.5	46.6
GS(^{3}**3**) -> TS(^{1}**3'**)	44.8	42.7	-0.6	**43.4**
GS(^{1}**3**) -> TS(^{1}**3'**)	42.7	40.9	-0.3	41.2
GS(^{3}**3**) -> ^{1}**4**	23.8	21.5	16.5	**5.0**
GS(^{3}**3**) -> ^{1}**4'**	17.0	13.3	-25.5	**-1.8**

[1] two imaginary frequencies (–9 cm^{-1}, –357 cm^{-1})

Table S34 Relative electronic energies and thermodynamic data (kcal/mol) for all species potentially involved in the dissociation process computed with the density functional B3LYP with D3BJ (see computational details) (no improvement).

	$FSPE_{rel}$	ΔH	T*ΔS	ΔG
GS(^{3}**3**) -> TS(^{3}**3**)	66.8	64.4	0.2	64.3
GS(^{3}**3**) -> TS(^{1}**3**)	44.0	41.1	-1.9	43.0
GS(^{1}**3**) -> TS(^{1}**3**)	38.8	-59.4	-1.5	37.7
GS(^{3}**3**) -> TS(^{1}**3'**)	39.0	36.9	-0.6	**37.6**
GS(^{1}**3**) -> TS(^{1}**3'**)	33.8	31.9	-0.3	32.2
GS(^{3}**3**) -> ^{1}**4**	20.3	17.9	16.5	**1.5**
GS(^{3}**3**) -> ^{1}**4'**	13.5	9.8	-25.5	**-5.2**

Table S35 Relative electronic energies and thermodynamic data (kcal/mol) for all species potentially involved in the dissociation process computed with the density functional M06 with D3ZERO (see computational details) (no improvement).

	$FSPE_{rel}$	ΔH	T*ΔS	ΔG
GS(^{3}**3**) -> TS(^{3}**3**)	70.1	67.7	0.2	67.5
GS(^{3}**3**) -> TS(^{1}**3**)	45.5	42.6	-1.9	44.5
GS(^{1}**3**) -> TS(^{1}**3**)	39.3	-61.7	-1.5	38.2
GS(^{3}**3**) -> TS(^{1}**3'**)	39.3	37.2	-0.6	**37.9**
GS(^{1}**3**) -> TS(^{1}**3'**)	33.1	31.3	-0.3	31.5
GS(^{3}**3**) -> ^{1}**4**	19.4	17.1	16.5	**0.6**
GS(^{3}**3**) -> ^{1}**4'**	12.5	8.8	-25.5	**-6.3**

Table S36 Relative electronic energies and thermodynamic data (kcal/mol) for all species potentially involved in the dissociation process computed with the density functional M062X with D3ZERO (see computational details) (no improvement).

	$FSPE_{rel}$	ΔH	T*ΔS	ΔG
GS(^{3}**3**) -> TS(^{3}**3**)	73.7	71.3	0.2	71.2
GS(^{3}**3**) -> TS(^{1}**3**)	51.6	48.8	-1.9	50.7
GS(^{1}**3**) -> TS(^{1}**3**)	41.3	38.6	-1.5	40.1
GS(^{3}**3**) -> TS(^{1}**3'**)	44.2	42.2	-0.6	**42.8**
GS(^{1}**3**) -> TS(^{1}**3'**)	33.8	32.0	-0.3	32.3
GS(^{3}**3**) -> ^{1}**4**	12.8	10.5	16.5	**-6.0**
GS(^{3}**3**) -> ^{1}**4'**	4.9	1.1	-25.5	**-13.9**

Table S37 Relative electronic energies and thermodynamic data (kcal/mol) for all species potentially involved in the dissociation process computed with the density functional TPSSh with D3BJ (see computational details) (no improvement).

	$FSPE_{rel}$	ΔH	T*ΔS	ΔG
GS(^{3}**3**) -> TS(^{3}**3**)	66.6	64.2	0.2	64.1
GS(^{3}**3**) -> TS(^{1}**3**)	44.7	41.9	-1.9	43.8
GS(^{1}**3**) -> TS(^{1}**3**)	39.6	36.9	-1.5	38.5
GS(^{3}**3**) -> TS(^{1}**3’**)	40.4	38.3	-0.6	**39.0**
GS(^{1}**3**) -> TS(^{1}**3’**)	35.2	33.4	-0.3	33.7
GS(^{3}**3**) -> ^{1}**4**	24.8	22.5	16.5	**6.0**
GS(^{3}**3**) -> ^{1}**4’**	18.6	14.9	-25.5	**-0.2**

Table S38 Relative electronic energies and thermodynamic data (kcal/mol) for all species potentially involved in the dissociation process computed with the density functional PWPB95 with D3BJ (see computational details) (no improvement).

	$FSPE_{rel}$	ΔH	T*ΔS	ΔG
GS(^{3}**3**) -> TS(^{3}**3**)	66.8	64.3	0.2	64.2
GS(^{3}**3**) -> TS(^{1}**3**)	34.7	31.8	-1.9	33.7
GS(^{1}**3**) -> TS(^{1}**3**)	34.4	31.7	0.6	33.3
GS(^{3}**3**) -> ^{1}**4**	7.5	5.2	16.5	**-11.3**
GS(^{3}**3**) -> ^{1}**4’**	-0.6	-4.3	-25.5	**-19.3**

Table S39 Relative electronic energies and thermodynamic data (kcal/mol) for all species potentially involved in the dissociation process computed with the density functional B2GP-PLYP with D3BJ (see computational details) (no improvement).

	$FSPE_{rel}$	ΔH	T*ΔS	ΔG
GS(^{3}**3**) -> TS(^{3}**3**)	60.4	58.0	0.2	57.9
GS(^{3}**3**) -> TS(^{1}**3**)	17.7	14.9	-1.9	16.8
GS(^{1}**3**) -> TS(^{1}**3**)	17.9	-58.4	0.6	16.8
GS(^{3}**3**) -> ^{1}**4**	-3.3	-5.7	16.5	**-22.1**
GS(^{3}**3**) -> ^{1}**4’**	-11.8	-15.5	-25.5	**-30.5**

Table S40 Relative electronic energies and thermodynamic data (kcal/mol) for all species potentially involved in the dissociation process computed with the density functional B2K-PLYP with D3BJ (see computational details) (no improvement).

	$FSPE_{rel}$	ΔH	T*ΔS	ΔG
GS(^{3}**3**) -> TS(^{3}**3**)	60.7	58.3	0.2	58.1
GS(^{3}**3**) -> TS(^{1}**3**)	13.7	10.9	-1.9	12.8
GS(^{1}**3**) -> TS(^{1}**3**)	13.3	10.7	0.6	12.2
GS(^{3}**3**) -> ^{1}**4**	-7.6	-9.9	16.5	**-26.4**
GS(^{3}**3**) -> ^{1}**4’**	-16.2	-19.9	-25.5	**-34.9**

Table S41 Relative electronic energies and thermodynamic data (kcal/mol) for all species potentially involved in the dissociation process computed with the density functional B2K-PLYP without D3BJ (see computational details) (no improvement).

	$FSPE_{rel}$	ΔH	T*ΔS	ΔG
GS(^{3}**3**) -> TS(^{3}**3**)	59.9	57.5	0.2	57.3
GS(^{3}**3**) -> TS(^{1}**3**)	12.4	9.6	-1.9	11.4
GS(^{1}**3**) -> TS(^{1}**3**)	12.0	9.4	0.6	10.9
GS(^{3}**3**) -> ^{1}**4**	-17.0	-19.3	16.5	**-35.8**
GS(^{3}**3**) -> ^{1}**4’**	-25.9	-29.6	-25.5	**-44.6**

4.4.2 Thermodynamics and kinetics for thermal dissociation of tungsten dimer for selected functionals with extended basis sets

All energies in kcal/mol

GS(^{3}**3**): ground state with triplet multiplicity

GS(^{1}**3**): ground state with closed shell singlet multiplicity

^{1}**4**: monomer 1 ((same conformation of the pincer backbone as in **3**), singlet multiplicity

^{1}**4’**: monomer 2 (same conformation of the pincer backbone as in **3’**), singlet multiplicity

TS(^{3}**3**): transition state of **3** with triplet multiplicity

TS(^{1}**3**): transition state[1] of **3** with singlet multiplicity

Table S42 Relative electronic energies and thermodynamic data (kcal/mol) for all species potentially involved in the dissociation process computed with the density functional PBE/def2-TZVP for the important atoms; PBE/def2-SVP for the other atoms.

	$FSPE_{rel}$	ΔH	TΔS	ΔG
GS(^{3}**3**) -> TS(^{3}**3**)	61.5	59.1	0.2	58.9
GS(^{3}**3**) -> TS(^{1}**3**)	40.5	37.6	-1.9	39.5
GS(^{1}**3**) -> TS(^{1}**3**)	39.4	36.8	-1.5	38.3
GS(^{3}**3**) -> ^{1}**4**	24.1	21.8	16.5	**5.3**
GS(^{3}**3**) -> ^{1}**4’**	18.0	14.3	-25.5	**-0.7**

[1] two imaginary frequencies (–9 cm^{-1}, –357 cm^{-1})

Table S43 Relative electronic energies and thermodynamic data (kcal/mol) for all species potentially involved in the dissociation process computed with the density functional PBE/def2-TZVP for all atoms.

	$FSPE_{rel}$	ΔH	TΔS	ΔG
GS(^{3}**3**) -> TS(^{3}**3**)	61.7	59.3	0.2	59.1
GS(^{3}**3**) -> TS(^{1}**3**)	39.9	37.0	-1.9	38.9
GS(^{1}**3**) -> TS(^{1}**3**)	36.9	34.3	-1.5	35.8
GS(^{3}**3**) -> ^{1}**4**	21.6	19.3	16.5	**2.8**
GS(^{3}**3**) -> ^{1}**4'**	14.8	11.1	-25.5	**-3.9**

Table S44 Relative electronic energies and thermodynamic data (kcal/mol) for all species potentially involved in the dissociation process computed with the density functional PBE/def2-QZPP for the important atoms; PBE/def2-TZVP for the other atoms.

	$FSPE_{rel}$	ΔH	TΔS	ΔG
GS(^{3}**3**) -> TS(^{3}**3**)	60.7	58.3	0.2	58.2
GS(^{3}**3**) -> TS(^{1}**3**)	39.3	36.5	-1.9	38.4
GS(^{1}**3**) -> TS(^{1}**3**)	36.4	33.7	-1.5	35.2
GS(^{3}**3**) -> ^{1}**4**	20.2	17.9	16.5	**1.4**
GS(^{3}**3**) -> ^{1}**4'**	13.4	9.6	-25.5	**-5.4**

Table S45 Relative electronic energies and thermodynamic data (kcal/mol) for all species potentially involved in the dissociation process computed with the density functional PBE0/def2-TZVP for the important atoms; PBE0/def2-SVP for the other atoms.

	$FSPE_{rel}$	ΔH	TΔS	ΔG
GS(^{3}**3**) -> TS(^{3}**3**)	73.9	71.5	0.2	71.4
GS(^{3}**3**) -> TS(^{1}**3**)	49.7	46.9	-1.9	48.8
GS(^{1}**3**) -> TS(^{1}**3**)	47.7	45.1	0.6	46.6
GS(^{3}**3**) -> ^{1}**4**	23.8	21.5	16.5	**5.0**
GS(^{3}**3**) -> ^{1}**4'**	17.0	13.3	-25.5	**-1.8**

Table S46 Relative electronic energies and thermodynamic data (kcal/mol) for all species potentially involved in the dissociation process computed with the density functional PBE0/def2-TZVP for all atoms.

	$FSPE_{rel}$	ΔH	TΔS	ΔG
GS(^{3}**3**) -> TS(^{3}**3**)	73.9	71.5	0.2	71.4
GS(^{3}**3**) -> TS(^{1}**3**)	49.2	46.4	-1.9	48.3
GS(^{1}**3**) -> TS(^{1}**3**)	47.1	44.5	0.6	46.0
GS(^{3}**3**) -> ^{1}**4**	21.8	19.4	16.5	**3.0**
GS(^{3}**3**) -> ^{1}**4'**	14.7	10.9	-25.5	**-4.1**

Table S47 Relative electronic energies and thermodynamic data (kcal/mol) for all species potentially involved in the dissociation process computed with the density functional PBE0/def2-QZVPP for the important atoms; PBE0/def2-TZVP for the other atoms.

	$FSPE_{rel}$	ΔH	TΔS	ΔG
GS(^{3}**3**) -> TS(^{3}**3**)	72.8	70.3	0.2	70.2
GS(^{3}**3**) -> TS(^{1}**3**)	48.6	45.8	-1.9	47.7
GS(^{1}**3**) -> TS(^{1}**3**)	46.6	44.0	0.6	45.5
GS(^{3}**3**) -> ^{1}**4**	20.3	18.0	16.5	**1.5**
GS(^{3}**3**) -> ^{1}**4’**	13.2	9.5	-25.5	-5.5

Table S48 Relative electronic energies and thermodynamic data (kcal/mol) for all species potentially involved in the dissociation process computed with the density functional PWPB95/def2-TZVP for the important atoms; PWPB95/def2-SVP for the other atoms.

	$FSPE_{rel}$	ΔH	TΔS	ΔG
GS(^{3}**3**) -> TS(^{3}**3**)	66.8	64.3	0.2	64.2
GS(^{3}**3**) -> TS(^{1}**3**)	34.7	31.8	-1.9	33.7
GS(^{1}**3**) -> TS(^{1}**3**)	34.4	31.7	0.6	33.3
GS(^{3}**3**) -> ^{1}**4**	7.5	5.2	16.5	**-11.3**
GS(^{3}**3**) -> ^{1}**4’**	-0.6	-4.3	-25.5	**-19.3**

Table S49 Relative electronic energies and thermodynamic data (kcal/mol) for all species potentially involved in the dissociation process computed with the density functional PWPB95/def2-TZVP for all atoms.

	$FSPE_{rel}$	ΔH	T*ΔS	ΔG
GS(^{3}**3**) -> TS(^{3}**3**)	66.7	64.3	0.2	64.1
GS(^{3}**3**) -> TS(^{1}**3**)	33.7	30.9	-1.9	32.8
GS(^{1}**3**) -> TS(^{1}**3**)	33.5	30.8	0.6	32.3
GS(^{3}**3**) -> ^{1}**4**	5.8	3.4	16.5	**-13.0**
GS(^{3}**3**) -> ^{1}**4’**	-2.0	-5.7	-25.5	**-20.8**

Table S50 Relative electronic energies and thermodynamic data (kcal/mol) for all species potentially involved in the dissociation process computed with the density functional PWPB95/def2-QZVPP for the important atoms; PWPB95/def2-TZVP for the other atoms.

	$FSPE_{rel}$	ΔH	TΔS	ΔG
GS(^{3}**3**) -> TS(^{3}**3**)	65.4	63.0	0.2	62.8
GS(^{3}**3**) -> TS(^{1}**3**)	32.7	29.9	-1.9	31.8
GS(^{1}**3**) -> TS(^{1}**3**)	34.4	31.7	0.6	33.2
GS(^{3}**3**) -> ^{1}**4**	3.5	1.2	16.5	**-15.3**
GS(^{3}**3**) -> ^{1}**4’**	-3.9	-7.7	-25.5	**-22.7**

4.5 Cartesian coordinates of ground state structures (Å)

Cartesian Coordinates of ^{3}3 GS

W	6.444760147597	7.618934699440	12.965724981037
N	8.143687635829	7.897220257732	13.733839901519
N	9.299339093340	7.897301429587	14.078855237668
W	10.998697904510	7.619956444320	14.846021158367
N	11.244220462877	6.165155469325	16.301283524688
P	11.962641575661	5.753073030519	13.493122034310
C	11.907148719513	9.087444319714	13.916938384387
O	12.508367476499	10.001012641228	13.413140404974
P	10.674869875780	8.943266036062	16.899271898057
N	6.197083046755	6.164756711507	11.510463085332
P	5.481790674490	5.752326450170	14.320622546379
C	5.534670986567	9.087119656108	13.891955172895
O	4.932156940885	10.001105139704	14.393110695366
P	6.768714605216	8.941734338693	10.911957468506
H	6.858630355670	5.469998461428	9.624242709303
C	2.978857106820	5.878358833984	13.145987654949
H	3.457284706151	6.671688439796	12.536815940771
H	1.896540760447	6.112753832883	13.226741824632
H	3.072056623449	4.918843355773	12.602888555998
C	3.186081433323	7.085399865918	15.316058544466
H	3.537315948221	7.990554986133	14.789241387581
H	3.583389324494	7.116400959507	16.346763388153
H	2.077904374379	7.130916929223	15.382050984504
C	3.008465867297	4.578281003898	15.280362843620
H	1.899400912154	4.620394651809	15.228348623792
H	3.283015503636	4.551352885662	16.352004758018
H	3.323262889327	3.624377385012	14.812153652408
C	3.586650011417	5.806276656579	14.560904861014
C	7.830675611185	5.344216319810	15.724707435596
H	8.210609722795	4.831134573255	14.819695212800
H	8.376117539181	4.924241073285	16.595939646636
H	8.103945359319	6.407980618605	15.635812463641
C	6.062120671018	3.664370906062	16.243367271918
H	6.426585891125	3.000135176969	15.435587430836
H	4.998726149921	3.433715589094	16.427237365708
H	6.625342995251	3.396703515868	17.163224067186
C	5.874400271567	6.030816327674	17.107487033935
H	5.957980031015	7.107768675095	16.859849019503
H	6.531559081508	5.831759440925	17.980951906444
H	4.833979885136	5.821334157985	17.422091233826
C	6.318512624350	5.149499529704	15.928781156028
C	7.165960613339	7.606840950510	9.705887606154
H	7.032510803633	7.898367039319	8.646188301194
H	8.237784527868	7.384427635104	9.862713079413
H	5.049843708755	3.494014289336	13.349448916074
C	9.525233552009	9.339776313689	10.918423365521
H	9.724545738480	8.750105863293	10.003031788480
H	9.527743504166	8.661071945994	11.790767558475
H	10.377080661642	10.038353570604	11.053565492881
C	8.151395881580	11.091679690728	12.039256996033
H	9.063233375635	11.724575494786	12.073737173578
H	8.101499181352	10.501708154322	12.973950160356
H	7.274005855504	11.762692327011	12.013188241120
C	8.272631048299	10.978085126221	9.510678406234
H	8.298672088970	10.326299523355	8.614304651576
H	9.200688054861	11.588985661024	9.502651635688
H	7.420496286320	11.676536556570	9.410963305889
C	8.225799202997	10.162793650105	10.812764456746
C	4.968809428154	11.132080671685	10.882652431003
H	5.703794564833	11.879579679216	10.529359052760
H	5.023640849765	11.077556149776	11.987272473033
H	3.959259733005	11.508841079824	10.612705518157
C	5.154639670672	9.867979152789	8.703456964294
H	5.166537009815	8.875021926096	8.212647656326
H	5.998366862639	10.459415451297	8.304414818924
H	4.215479093940	10.374053162520	8.392798917855
C	3.996943635853	8.852231724849	10.671257797232
H	3.048049404026	9.291960807626	10.296238148673
H	3.931778202986	8.782613544983	11.774861658250

H	4.074910220585	7.825030780665	10.267564978019
C	5.173721723023	9.749993262182	10.237224893594
H	6.770196698506	3.962727475749	13.415629535341
C	5.719362322859	4.789436795254	11.714596116031
H	4.690882356993	4.673469645698	11.296034958263
C	11.716576231400	4.787963865868	16.096950544265
H	11.075543722561	4.097870566645	16.697703959740
H	12.743400583525	4.667162045283	16.518094907248
H	6.355866846950	4.097370215595	11.111431409516
C	6.350537430137	6.364179638955	10.059627839331
H	5.350295176025	6.397559682631	9.559734010186
C	11.683898378469	4.331244438503	14.638838022898
H	10.667250254174	3.965595848209	14.392964841119
H	12.386731124853	3.492696731390	14.461829680036
C	11.092055501356	6.365177636420	17.752139248095
H	12.092622929872	6.397548709589	18.251409602690
H	10.582972081746	5.471954481868	18.188300919962
C	14.462322394312	5.867544047193	14.678012106443
H	13.982989045493	6.658372595132	15.289703282909
H	15.545131648824	6.101211923722	14.601572802147
H	14.366642848373	4.905380440307	15.216005174084
C	14.265378507543	7.084783212507	12.513102114066
H	13.919566085904	7.987895069275	13.046996377179
H	13.866564825140	7.125149792104	11.483296230682
H	15.373682078693	7.125144802287	12.445936953171
C	14.436238287959	4.576553165487	12.538361873987
H	15.545232950831	4.615028479974	12.595018385056
H	14.165941101893	4.555034269428	11.465526484394
H	14.116138850329	3.621804552028	13.001330390123
C	13.858915554482	5.803177182951	13.260781498006
C	9.617934489985	5.350150466445	12.080661722263
H	9.234449131780	4.833170096664	12.981962247377
H	9.075038913674	4.934726804395	11.205656864395
H	9.345296990793	6.413715819857	12.173812427404
C	11.387148686303	3.671002218531	11.561672067136
H	11.023325978989	3.004090345896	12.367579010073
H	12.450450989988	3.441011782863	11.376599021270
H	10.823501575263	3.405751604594	10.641355888551
C	11.578979338304	6.040425227194	10.706337536950
H	11.494802959741	7.116533269366	10.957473060553
H	10.924710166358	5.844783400058	9.829940921006
H	12.620389036978	5.831774687573	10.394354825073
C	11.130693982561	5.155191754431	11.880619327823
C	10.277954537952	7.608688064907	18.106001961312
H	10.412389618495	7.900205583848	19.165610859306
H	9.205920588512	7.386748829569	17.949972366744
C	5.754048891973	4.331327333984	13.172126613395
C	7.917989109478	9.341173507552	16.894577289249
H	7.718464818094	8.753806162054	17.811444525634
H	7.915258944379	8.660396585936	16.023813919817
H	7.066157304441	10.039461138370	16.757964247773
C	9.291072042144	11.091880836385	15.770921280348
H	8.379477592952	11.725150892103	15.736599884815
H	9.339463704380	10.500831878888	14.836828915935
H	10.168837882056	11.762517456059	15.795272870303
C	9.171752332852	10.980920321228	18.299718962768
H	9.146656639779	10.330083077769	19.196809538918
H	8.243576453950	11.591653773355	18.308211749236
H	10.023826822502	11.679655108750	18.397924669286
C	9.217575767004	10.164260438231	16.998442932392
C	12.475102058543	11.133129453075	16.925576861871
H	11.740542043173	11.881229945813	17.278508869646
H	12.419568392440	11.077431915863	15.821038369665
H	13.484929673082	11.509890780236	17.194492476872
C	12.290344486887	9.871254233394	19.106223228096
H	12.278071077158	8.878689121146	19.597753395985
H	11.447335409496	10.463740967168	19.505288127769
H	13.230080784294	10.376858632915	19.415847508467
C	13.446126915246	8.852940379852	17.138761701053
H	14.395582333784	9.292577433333	17.512452322652
H	13.510347993617	8.781799342856	16.035158608722
H	13.367764016473	7.826392510467	17.544008621169

C	12.270169424569	9.751835545456	17.572618223681

Cartesian Coordinates of ^{1}3 GS

W	6.455935534179	7.613214506008	12.978958842373
N	8.138247486308	7.990556432057	13.750933133862
N	9.305357172107	7.990497418065	14.061879549950
W	10.987803197747	7.613302610464	14.833628602676
N	11.269021741595	6.124069765234	16.256026800489
P	12.023298659745	5.783963383338	13.433886760403
C	11.899178730694	9.188065580705	14.163695634371
O	12.529022088804	10.163724417653	13.828361324518
P	10.660120651426	8.859326555996	16.924698314587
N	6.174288086278	6.124247853101	11.556536036595
P	5.421043246518	5.783763388048	14.379077365591
C	5.544479099924	9.188335607376	13.648416350785
O	4.914471245475	10.163891608925	13.983224332352
P	6.784374859529	8.859106052788	10.887865019429
H	6.887125752221	5.348416197944	9.721234259773
C	2.927915094268	6.124368939171	13.281838397382
H	3.446934594163	6.906532107643	12.690239808318
H	1.864657198090	6.425956481517	13.386719337444
H	2.952306015658	5.179356224629	12.706865434736
C	3.274957482799	7.261987101655	15.474291856235
H	3.742400920073	8.138997234537	14.989664695045
H	3.640226696942	7.209205477471	16.515633978086
H	2.177421079002	7.429034771064	15.513440033793
C	2.904924848576	4.772615806224	15.392915898630
H	1.801033466639	4.896583816757	15.377100169668
H	3.209732957215	4.701488885977	16.454506152781
H	3.135036338604	3.809180114539	14.895571122398
C	3.551865993265	5.970977829877	14.683233165935
C	7.810388262713	5.281513015854	15.672859834239
H	8.118628344927	4.708248837381	14.776806116119
H	8.375328002688	4.872412843459	16.536555236952
H	8.132256668422	6.325944850775	15.522146422063
C	5.974673723254	3.704898963318	16.295063480090
H	6.268388047605	3.010280590819	15.484238562733
H	4.906612038528	3.540859524849	16.521681131655
H	6.555928352636	3.417159325257	17.197169858786
C	5.950359559302	6.080850894998	17.139649659348
H	6.060994534091	7.151122394014	16.879014554238
H	6.640453106770	5.862906262931	17.982393577034
H	4.919359068755	5.912440583426	17.505386288127
C	6.301291108862	5.169842914070	15.952464689383
C	7.235215112981	7.483692815636	9.748139903447
H	7.161707790553	7.735365526286	8.672834224426
H	8.294271784286	7.257516996464	9.972451108995
H	4.822253828041	3.561931410854	13.420466305429
C	9.537229065304	9.303311131198	10.906609847822
H	9.761265694942	8.705240575841	10.002647476413
H	9.536748052548	8.637622791890	11.788005425047
H	10.375244530595	10.017900192489	11.046210793351
C	8.120665817572	11.073162275852	11.950878188259
H	9.006867195385	11.742540793955	11.954918136577
H	8.096706046869	10.518625811980	12.908322359048
H	7.216650150144	11.706978626758	11.902557488870
C	8.289406619414	10.867281902316	9.429411380243
H	8.350404142563	10.181435901289	8.560699465812
H	9.208259296207	11.492013609672	9.420960344410
H	7.431009552432	11.547535158126	9.278014260599
C	8.226833164893	10.100566460582	10.761095239407
C	4.984200398608	11.057576374183	10.627594553658
H	5.737565497619	11.772426967256	10.248018986710
H	4.983853142425	11.097005463588	11.733228552643
H	3.990925438238	11.411344955148	10.277199468556
C	5.246675946559	9.617794847705	8.570930459885
H	5.238847304049	8.588522299885	8.161532815118
H	6.123542671276	10.146974543484	8.156236029161
H	4.337213576625	10.127819954263	8.187029521504
C	4.001206885602	8.776720654919	10.563969160142
H	3.082255182632	9.176135871536	10.083546687091
H	3.868826392496	8.823569066649	11.660729873975

H	4.097175048872	7.712179207978	10.278841542492
C	5.207062024967	9.624133812344	10.110089562292
H	6.572292517188	3.910995870734	13.466316863611
C	5.567901438259	4.802714974210	11.774863079972
H	4.526600520101	4.785538800151	11.371370856313
C	11.874339365528	4.802069486860	16.037660583548
H	11.316842674940	4.047911972573	16.643853985065
H	12.915365120906	4.783770622516	16.441841948833
H	6.124191502983	4.048313501308	11.167873617417
C	6.384218181880	6.268564946401	10.106711028847
H	5.403895711348	6.307868677395	9.568684140309
C	11.861645380164	4.347366303984	14.579556572968
H	10.870306261176	3.911613386878	14.345601451821
H	12.620126462475	3.561327697281	14.392037226407
C	11.059250432918	6.268515272678	17.705803713611
H	12.039657180183	6.307097428032	18.243743214218
H	10.555815706686	5.348707082971	18.091438895165
C	14.515685561091	6.124094644268	14.533434786589
H	13.997190844859	6.907098294771	15.124328209759
H	15.579421905685	6.424054903410	14.428930373555
H	14.489766973466	5.179321224054	15.108735771328
C	14.170585042646	7.261568914857	12.340544662498
H	13.702303167333	8.138706793108	12.824143723597
H	13.806905964823	7.208205186718	11.298676907968
H	15.268169657919	7.428655237455	12.303038163675
C	14.539720410334	4.772186442295	12.422550729742
H	15.643557521449	4.896362814235	12.438719377546
H	14.234510032688	4.700700990476	11.361159034502
H	14.309455973683	3.809012179954	12.920338188069
C	13.892716596063	5.970776357074	13.131637361516
C	9.634824933544	5.282318686102	12.138121647607
H	9.325533812656	4.706859908809	13.032419160439
H	9.070336917293	4.875703245502	11.272930521652
H	9.312978637473	6.326429031898	12.291121061400
C	11.471582418285	3.706797969829	11.515208599394
H	11.178637399889	3.011020365852	12.325313305532
H	12.539638475537	3.543935491483	11.287717236409
H	10.890285131838	3.419233493173	10.613021604818
C	11.495730911315	6.083657519796	10.673390207379
H	11.386607325227	7.153647706018	10.935835093990
H	10.804766987501	5.868241348046	9.830697762944
H	12.526390153053	5.914219007862	10.307133943623
C	11.144145939746	5.171224613817	11.859344185514
C	10.209045309390	7.484120228518	18.064571907161
H	10.282693668131	7.735613247731	19.139924182744
H	9.149824674970	7.258741795166	17.840290405326
C	5.581171009987	4.347514128239	13.232813700546
C	7.907242611403	9.303515747465	16.905877185430
H	7.683302741852	8.705635443719	17.810001361889
H	7.907933668529	8.637613431996	16.024643505811
H	7.069288645354	10.018125659536	16.765900623353
C	9.323886392198	11.073224580107	15.861361181235
H	8.437336625913	11.742158893648	15.856553046783
H	9.348690455909	10.518577757537	14.904004799389
H	10.227552394982	11.707507504893	15.909966862242
C	9.155146361678	10.867654314193	18.382924745228
H	9.094666316132	10.181857109970	19.251703680482
H	8.235998288991	11.491975199568	18.391799644947
H	10.013318453343	11.548268607389	18.533997997954
C	9.217651206318	10.100828097164	17.051291554322
C	12.460561940806	11.057540144071	17.183654992127
H	11.707339273928	11.772660298623	17.563006667928
H	12.460683750882	11.096334454725	16.077994356649
H	13.453943708303	11.411379136770	17.533668690520
C	12.198169445265	9.619002765007	19.241148756954
H	12.206239698129	8.590002950683	19.651191864570
H	11.321239475042	10.148154161038	19.655681674097
H	13.107614286722	10.129321060936	19.624687027517
C	13.443326038876	8.776634647546	17.248502915477
H	14.362459221357	9.176660190356	17.728063988292
H	13.575134655456	8.822302392738	16.151619531084
H	13.347771140394	7.712328812088	17.534763019949

C 12.237603976535 9.624413673704 17.702005380493

Cartesian Coordinates of ^{3}3’ GS

W -0.059224000000 0.275945000000 0.055366000000
N -0.147979000000 0.122460000000 1.935937000000
N -0.223431000000 -0.049874000000 3.131847000000
W -0.239609000000 -0.221453000000 5.012350000000
C 0.273094000000 -2.110559000000 5.044185000000
O 0.566929000000 -3.271422000000 5.169355000000
C 1.861752000000 0.640115000000 -0.021287000000
O 3.030582000000 0.886000000000 -0.174098000000
N -0.633544000000 1.397166000000 6.250601000000
P 2.056408000000 0.567346000000 5.562162000000
P -2.619203000000 -0.649544000000 5.629501000000
H -1.787033000000 2.260856000000 7.804779000000
C 2.411710000000 2.256393000000 3.429762000000
H 1.397415000000 1.941638000000 3.125653000000
H 2.958496000000 2.542486000000 2.507199000000
H 2.329416000000 3.159209000000 4.066714000000
C 3.315473000000 -0.054144000000 3.135131000000
H 2.328515000000 -0.406160000000 2.785413000000
H 3.857695000000 -0.913555000000 3.569253000000
H 3.875805000000 0.293079000000 2.243314000000
C 4.558133000000 1.614677000000 4.555532000000
H 5.066230000000 2.058989000000 3.672920000000
H 5.203514000000 0.795099000000 4.924811000000
H 4.504447000000 2.399543000000 5.336697000000
C 3.171769000000 1.112199000000 4.127075000000
C 1.980699000000 -0.975378000000 7.846195000000
H 1.496538000000 -0.127697000000 8.371279000000
H 2.484462000000 -1.604029000000 8.611012000000
H 1.179321000000 -1.573583000000 7.372332000000
C 4.133956000000 0.255928000000 7.564830000000
H 3.754963000000 1.154907000000 8.089194000000
H 4.962879000000 0.566129000000 6.905244000000
H 4.560656000000 -0.416189000000 8.339998000000
C 3.624713000000 -1.727773000000 6.105242000000
H 2.874516000000 -2.249793000000 5.481477000000
H 3.995559000000 -2.446568000000 6.866793000000
H 4.485920000000 -1.451921000000 5.466546000000
C 3.024184000000 -0.502461000000 6.813539000000
C -2.877188000000 0.586233000000 6.982247000000
H -3.936226000000 0.903412000000 7.059521000000
H -2.624963000000 0.058473000000 7.922219000000
H 2.458180000000 2.904752000000 6.295225000000
C -1.925752000000 -2.567505000000 7.512155000000
H -1.879587000000 -1.796795000000 8.307364000000
H -0.935235000000 -2.620683000000 7.028797000000
H -2.123116000000 -3.541768000000 8.007796000000
C -3.074087000000 -3.407484000000 5.441024000000
H -3.141298000000 -4.395972000000 5.942974000000
H -2.152130000000 -3.395740000000 4.826547000000
H -3.946394000000 -3.318893000000 4.764336000000
C -4.379488000000 -2.263638000000 7.281815000000
H -4.421241000000 -1.425204000000 8.005024000000
H -4.463051000000 -3.203623000000 7.868243000000
H -5.267789000000 -2.203463000000 6.631366000000
C -3.053861000000 -2.290241000000 6.497726000000
C -3.577460000000 -0.905387000000 2.999058000000
H -2.566883000000 -0.614661000000 2.658477000000
H -4.305593000000 -0.601709000000 2.216547000000
H -3.606692000000 -2.008207000000 3.076680000000
C -5.384067000000 -0.545445000000 4.714192000000
H -5.666796000000 -0.120485000000 5.697719000000
H -5.575003000000 -1.635099000000 4.730988000000
H -6.066549000000 -0.105638000000 3.955366000000
C -3.824956000000 1.309054000000 4.127984000000
H -4.457543000000 1.604956000000 3.267171000000
H -2.783099000000 1.617222000000 3.909109000000
H -4.185057000000 1.871538000000 5.010258000000
C -3.937294000000 -0.212301000000 4.324159000000

H	1.893879000000	1.849614000000	7.613315000000
C	0.289271000000	2.544243000000	6.349737000000
H	0.199048000000	3.192512000000	5.445375000000
H	-0.002999000000	3.187954000000	7.212560000000
C	-1.937669000000	1.777547000000	6.808745000000
H	-2.417872000000	2.561056000000	6.174150000000
C	1.737661000000	2.102478000000	6.547998000000
N	-1.715202000000	-0.024639000000	-1.161247000000
P	0.213171000000	-2.115336000000	-0.584267000000
P	-0.727680000000	2.627779000000	-0.444970000000
H	-3.043696000000	0.705040000000	-2.642908000000
C	-1.159352000000	-3.224463000000	1.514398000000
H	-1.309438000000	-2.177733000000	1.834784000000
H	-1.179429000000	-3.856286000000	2.426921000000
H	-2.012751000000	-3.527173000000	0.875892000000
C	1.317370000000	-3.078502000000	1.807448000000
H	1.217253000000	-2.050275000000	2.198337000000
H	2.324146000000	-3.186647000000	1.365076000000
H	1.245746000000	-3.767176000000	2.673831000000
C	0.325848000000	-4.860050000000	0.328717000000
H	0.138031000000	-5.537652000000	1.189081000000
H	1.340988000000	-5.086903000000	-0.049009000000
H	-0.408891000000	-5.116068000000	-0.461069000000
C	0.196965000000	-3.406511000000	0.806886000000
C	1.568476000000	-1.316316000000	-2.849596000000
H	0.593399000000	-1.223269000000	-3.368509000000
H	2.348642000000	-1.481491000000	-3.622868000000
H	1.771166000000	-0.351928000000	-2.346845000000
C	1.372663000000	-3.795919000000	-2.645537000000
H	0.396077000000	-3.818736000000	-3.167688000000
H	1.446905000000	-4.698617000000	-2.014748000000
H	2.159937000000	-3.871153000000	-3.426080000000
C	2.955109000000	-2.536198000000	-1.149528000000
H	3.107682000000	-1.656587000000	-0.495516000000
H	3.761271000000	-2.537615000000	-1.913874000000
H	3.075702000000	-3.453692000000	-0.541755000000
C	1.588695000000	-2.493352000000	-1.853085000000
C	-1.991216000000	2.379423000000	-1.773487000000
H	-2.742386000000	3.194006000000	-1.789617000000
H	-1.436102000000	2.428332000000	-2.730014000000
H	-1.720660000000	-3.458679000000	-1.369333000000
C	1.239143000000	2.917432000000	-2.380190000000
H	0.540634000000	2.587879000000	-3.175076000000
H	1.718187000000	2.020041000000	-1.952356000000
H	2.025280000000	3.533567000000	-2.866124000000
C	1.567307000000	4.225771000000	-0.259786000000
H	2.410512000000	4.740686000000	-0.767260000000
H	1.978602000000	3.364091000000	0.301995000000
H	1.131102000000	4.939717000000	0.466025000000
C	-0.085572000000	4.986205000000	-2.016426000000
H	-0.887809000000	4.694253000000	-2.722793000000
H	0.705686000000	5.489364000000	-2.612424000000
H	-0.495323000000	5.734945000000	-1.318285000000
C	0.535190000000	3.769441000000	-1.304467000000
C	-0.840268000000	3.496177000000	2.227476000000
H	0.137280000000	4.005938000000	2.140358000000
H	-0.652753000000	2.448540000000	2.525603000000
H	-1.407657000000	3.990281000000	3.045160000000
C	-2.001015000000	5.028509000000	0.603597000000
H	-2.530588000000	5.136086000000	-0.363774000000
H	-1.104649000000	5.676875000000	0.590770000000
H	-2.675800000000	5.418874000000	1.395613000000
C	-2.972560000000	2.793775000000	1.129000000000
H	-3.491116000000	3.199061000000	2.020847000000
H	-2.789840000000	1.714001000000	1.300371000000
H	-3.659636000000	2.906759000000	0.268961000000
C	-1.658507000000	3.567533000000	0.926848000000
H	-1.025668000000	-2.426224000000	-2.642837000000
C	-2.343291000000	-1.350415000000	-1.316531000000
H	-2.958659000000	-1.595100000000	-0.417707000000
H	-3.057678000000	-1.329094000000	-2.173082000000
C	-2.644804000000	1.005583000000	-1.643476000000

H	-3.539677000000	1.062777000000	-0.977806000000
C	-1.314928000000	-2.448920000000	-1.575471000000

Cartesian Coordinates of [1]3' GS

W	-0.061210000000	0.282842000000	0.062690000000
N	-0.173104000000	0.141714000000	1.938125000000
N	-0.244910000000	-0.022866000000	3.138745000000
W	-0.246676000000	-0.219749000000	5.012151000000
C	0.213264000000	-2.120695000000	5.041890000000
O	0.476073000000	-3.290416000000	5.164053000000
C	1.848612000000	0.693845000000	-0.020851000000
O	3.012216000000	0.966771000000	-0.175877000000
N	-0.613259000000	1.384224000000	6.280214000000
P	2.064242000000	0.536539000000	5.560480000000
P	-2.624231000000	-0.630566000000	5.640579000000
H	-1.764266000000	2.260843000000	7.828944000000
C	2.398034000000	2.277255000000	3.466044000000
H	1.373425000000	1.977994000000	3.180110000000
H	2.926232000000	2.575007000000	2.536372000000
H	2.339615000000	3.168620000000	4.121480000000
C	3.292040000000	-0.027801000000	3.102475000000
H	2.301890000000	-0.352937000000	2.736683000000
H	3.818068000000	-0.907859000000	3.514377000000
H	3.862340000000	0.335260000000	2.223398000000
C	4.555332000000	1.599789000000	4.550757000000
H	5.052665000000	2.069786000000	3.675408000000
H	5.203314000000	0.767844000000	4.886115000000
H	4.514127000000	2.360323000000	5.356314000000
C	3.162504000000	1.112956000000	4.125091000000
C	2.002031000000	-1.052391000000	7.810313000000
H	1.545576000000	-0.209218000000	8.366384000000
H	2.506677000000	-1.711054000000	8.548762000000
H	1.180257000000	-1.621587000000	7.335504000000
C	4.169554000000	0.151990000000	7.519563000000
H	3.814406000000	1.050745000000	8.060878000000
H	4.996474000000	0.455213000000	6.854218000000
H	4.592291000000	-0.539171000000	8.279969000000
C	3.603886000000	-1.792360000000	6.025487000000
H	2.834146000000	-2.286122000000	5.402095000000
H	3.973542000000	-2.535094000000	6.764248000000
H	4.459660000000	-1.517077000000	5.379241000000
C	3.036294000000	-0.573470000000	6.771106000000
C	-2.872001000000	0.603273000000	6.996838000000
H	-3.927004000000	0.934333000000	7.070453000000
H	-2.631094000000	0.069356000000	7.936217000000
H	2.499308000000	2.847585000000	6.352904000000
C	-1.954229000000	-2.560731000000	7.521919000000
H	-1.899399000000	-1.790751000000	8.317276000000
H	-0.963700000000	-2.626947000000	7.040784000000
H	-2.165905000000	-3.532193000000	8.017220000000
C	-3.115783000000	-3.384304000000	5.449418000000
H	-3.194497000000	-4.371939000000	5.951436000000
H	-2.196418000000	-3.385434000000	4.831433000000
H	-3.989743000000	-3.283902000000	4.776530000000
C	-4.402718000000	-2.224688000000	7.291556000000
H	-4.433143000000	-1.387379000000	8.016551000000
H	-4.497966000000	-3.164657000000	7.876215000000
H	-5.290642000000	-2.151865000000	6.641976000000
C	-3.077747000000	-2.267940000000	6.506615000000
C	-3.589766000000	-0.876481000000	3.010951000000
H	-2.576157000000	-0.599199000000	2.668514000000
H	-4.315382000000	-0.563861000000	2.229649000000
H	-3.632932000000	-1.978841000000	3.089338000000
C	-5.389461000000	-0.493912000000	4.727904000000
H	-5.666299000000	-0.065914000000	5.711768000000
H	-5.593895000000	-1.581111000000	4.744500000000
H	-6.067154000000	-0.045505000000	3.969828000000
C	-3.809465000000	1.341758000000	4.140607000000
H	-4.444236000000	1.645782000000	3.284397000000
H	-2.765608000000	1.638052000000	3.914908000000
H	-4.157210000000	1.907872000000	5.025546000000

C	-3.939009000000	-0.178339000000	4.336481000000
H	1.922105000000	1.765521000000	7.644195000000
C	0.326182000000	2.515322000000	6.401055000000
H	0.243411000000	3.185211000000	5.511775000000
H	0.044920000000	3.144093000000	7.278516000000
C	-1.915687000000	1.781618000000	6.831190000000
H	-2.380860000000	2.573055000000	6.195116000000
C	1.768330000000	2.048881000000	6.586130000000
N	-1.701479000000	-0.042889000000	-1.170738000000
P	0.240232000000	-2.110985000000	-0.565476000000
P	-0.743782000000	2.622262000000	-0.466545000000
H	-3.037528000000	0.664689000000	-2.656262000000
C	-1.141667000000	-3.217916000000	1.529233000000
H	-1.304960000000	-2.170351000000	1.841041000000
H	-1.161956000000	-3.843089000000	2.446193000000
H	-1.986712000000	-3.534263000000	0.886318000000
C	1.331700000000	-3.051985000000	1.842316000000
H	1.212312000000	-2.028904000000	2.240940000000
H	2.342807000000	-3.140617000000	1.405671000000
H	1.266024000000	-3.747518000000	2.703554000000
C	0.366860000000	-4.846697000000	0.362692000000
H	0.173133000000	-5.521866000000	1.223559000000
H	1.388199000000	-5.067500000000	-0.001525000000
H	-0.356049000000	-5.111088000000	-0.435162000000
C	0.222290000000	-3.392284000000	0.833871000000
C	1.597613000000	-1.308343000000	-2.825319000000
H	0.629244000000	-1.245179000000	-3.360881000000
H	2.394257000000	-1.458936000000	-3.584573000000
H	1.766336000000	-0.334950000000	-2.326824000000
C	1.447280000000	-3.789921000000	-2.600396000000
H	0.471072000000	-3.840298000000	-3.121428000000
H	1.543394000000	-4.685298000000	-1.962084000000
H	2.235734000000	-3.852997000000	-3.380779000000
C	2.992632000000	-2.485239000000	-1.102264000000
H	3.120207000000	-1.596781000000	-0.454662000000
H	3.806185000000	-2.476697000000	-1.858643000000
H	3.126104000000	-3.394898000000	-0.485464000000
C	1.632293000000	-2.476407000000	-1.818631000000
C	-2.002798000000	2.354411000000	-1.795529000000
H	-2.761917000000	3.161442000000	-1.817144000000
H	-1.446652000000	2.403397000000	-2.751432000000
H	-1.672858000000	-3.477994000000	-1.357777000000
C	1.223623000000	2.924609000000	-2.402971000000
H	0.528865000000	2.577485000000	-3.193640000000
H	1.719714000000	2.039224000000	-1.970444000000
H	1.997213000000	3.551363000000	-2.895501000000
C	1.530750000000	4.253225000000	-0.290240000000
H	2.367929000000	4.774356000000	-0.801371000000
H	1.953038000000	3.403701000000	0.281508000000
H	1.083011000000	4.969071000000	0.426576000000
C	-0.129927000000	4.975609000000	-2.054441000000
H	-0.923705000000	4.666911000000	-2.763162000000
H	0.656429000000	5.487467000000	-2.649499000000
H	-0.555007000000	5.721917000000	-1.363081000000
C	0.507062000000	3.772857000000	-1.332553000000
C	-0.865453000000	3.508906000000	2.199517000000
H	0.106127000000	4.029589000000	2.109389000000
H	-0.664992000000	2.465218000000	2.502693000000
H	-1.438592000000	4.000688000000	3.014562000000
C	-2.042468000000	5.018376000000	0.565780000000
H	-2.573762000000	5.113764000000	-0.401905000000
H	-1.153351000000	5.676559000000	0.548462000000
H	-2.721037000000	5.406532000000	1.355654000000
C	-2.991319000000	2.778227000000	1.105028000000
H	-3.516326000000	3.187388000000	1.991242000000
H	-2.798935000000	1.702024000000	1.287749000000
H	-3.677138000000	2.876185000000	0.242141000000
C	-1.684530000000	3.563133000000	0.898458000000
H	-0.978119000000	-2.446888000000	-2.632469000000
C	-2.315861000000	-1.375594000000	-1.321835000000
H	-2.935318000000	-1.621245000000	-0.426080000000
H	-3.024436000000	-1.365974000000	-2.183403000000

C	-2.642071000000	0.974971000000	-1.658543000000
H	-3.537510000000	1.025974000000	-0.993100000000
C	-1.275341000000	-2.465699000000	-1.567218000000

Cartesian Coordinates of ^{3}3” GS

W	6.764831000000	7.851210000000	12.181008000000
N	8.172400000000	7.760634000000	13.513761000000
N	9.107432000000	7.775704000000	14.273560000000
W	10.517451000000	7.836768000000	15.577305000000
C	11.454663000000	9.271652000000	14.678388000000
O	12.079914000000	10.183750000000	14.197661000000
C	5.756187000000	9.238918000000	13.033675000000
O	5.075444000000	10.124636000000	13.494073000000
N	10.949250000000	6.877327000000	17.384669000000
P	9.362790000000	9.302300000000	17.227407000000
P	12.157552000000	6.077708000000	14.868420000000
H	11.902385000000	5.344938000000	18.503727000000
C	7.406776000000	7.342079000000	17.262163000000
H	7.567155000000	7.139296000000	16.187110000000
H	6.380540000000	7.010825000000	17.523986000000
H	8.116330000000	6.721171000000	17.840177000000
C	6.619836000000	9.621791000000	16.624784000000
H	6.914680000000	9.485199000000	15.565928000000
H	6.591754000000	10.704674000000	16.849199000000
H	5.583416000000	9.240117000000	16.730880000000
C	7.116608000000	9.075494000000	19.038709000000
H	6.039952000000	8.819731000000	19.140651000000
H	7.241207000000	10.122599000000	19.368584000000
H	7.672641000000	8.423129000000	19.740475000000
C	7.542600000000	8.846198000000	17.578266000000
C	11.086325000000	11.481520000000	17.363398000000
H	11.425161000000	11.345911000000	18.408748000000
H	11.280237000000	12.539653000000	17.088682000000
H	11.704793000000	10.842128000000	16.704064000000
C	8.812817000000	11.931049000000	18.302273000000
H	9.072670000000	11.554153000000	19.311734000000
H	7.715634000000	11.865284000000	18.175896000000
H	9.082108000000	13.008640000000	18.272017000000
C	9.154277000000	11.705858000000	15.807756000000
H	9.724379000000	11.192494000000	15.013571000000
H	9.360873000000	12.795239000000	15.737860000000
H	8.079374000000	11.553803000000	15.605416000000
C	9.579812000000	11.194197000000	17.192875000000
C	12.556335000000	5.216696000000	16.455271000000
H	12.704151000000	4.130181000000	16.298125000000
H	13.517314000000	5.627913000000	16.816321000000
H	9.720112000000	9.112063000000	19.686924000000
C	14.352432000000	7.707732000000	15.298951000000
H	14.516137000000	7.286617000000	16.310658000000
H	13.626043000000	8.535107000000	15.388374000000
H	15.321328000000	8.127095000000	14.954172000000
C	13.776332000000	7.275084000000	12.896283000000
H	14.730858000000	7.782422000000	12.641024000000
H	12.966828000000	8.028764000000	12.862203000000
H	13.584020000000	6.518858000000	12.110733000000
C	14.941359000000	5.513806000000	14.275342000000
H	14.986209000000	4.962753000000	15.235649000000
H	15.943075000000	5.965270000000	14.110125000000
H	14.774474000000	4.782811000000	13.465521000000
C	13.888671000000	6.636719000000	14.289703000000
C	10.948297000000	5.302078000000	12.475308000000
H	10.252982000000	6.132663000000	12.695170000000
H	10.403744000000	4.535808000000	11.885412000000
H	11.758250000000	5.699734000000	11.836735000000
C	12.459585000000	3.543715000000	13.449811000000
H	12.965219000000	3.144419000000	14.351597000000
H	13.233465000000	3.854835000000	12.723538000000
H	11.895871000000	2.705256000000	12.987170000000
C	10.294213000000	4.106596000000	14.571378000000
H	9.743606000000	3.382019000000	13.937196000000

H	9.584097000000	4.903770000000	14.871292000000
H	10.622363000000	3.568080000000	15.481216000000
C	11.480372000000	4.680884000000	13.772794000000
H	11.214844000000	9.358805000000	18.738983000000
C	10.541224000000	7.319982000000	18.723272000000
H	9.654829000000	6.741278000000	19.086287000000
H	11.353292000000	7.086732000000	19.453873000000
C	11.470844000000	5.503499000000	17.487722000000
H	10.640733000000	4.763008000000	17.400609000000
C	10.253842000000	8.813463000000	18.764466000000
N	6.001598000000	7.292921000000	10.312674000000
P	8.030587000000	9.330773000000	10.637833000000
P	5.232518000000	5.983378000000	12.826317000000
H	4.575152000000	6.224481000000	9.163557000000
C	9.410082000000	7.033224000000	9.949773000000
H	9.066559000000	6.634626000000	10.922828000000
H	10.361739000000	6.520097000000	9.697386000000
H	8.664403000000	6.755128000000	9.182729000000
C	10.772217000000	8.817004000000	11.036945000000
H	10.479289000000	8.521031000000	12.063621000000
H	11.084149000000	9.877903000000	11.063018000000
H	11.665038000000	8.218732000000	10.759133000000
C	10.088190000000	9.025273000000	8.612892000000
H	11.019989000000	8.491007000000	8.327308000000
H	10.300729000000	10.107804000000	8.567202000000
H	9.328697000000	8.788476000000	7.841718000000
C	9.656225000000	8.554084000000	10.011436000000
C	6.909838000000	11.851840000000	11.015906000000
H	6.539113000000	11.998010000000	9.982541000000
H	7.005536000000	12.857293000000	11.477364000000
H	6.151172000000	11.289012000000	11.591819000000
C	9.226108000000	11.913604000000	10.087706000000
H	8.882760000000	11.848580000000	9.035758000000
H	10.269805000000	11.550861000000	10.140360000000
H	9.239311000000	12.989279000000	10.365702000000
C	8.824748000000	11.228387000000	12.489233000000
H	8.138729000000	10.706631000000	13.183031000000
H	8.908398000000	12.288898000000	12.807219000000
H	9.820580000000	10.762931000000	12.600720000000
C	8.289882000000	11.165511000000	11.049320000000
C	4.350977000000	5.674457000000	11.234098000000
H	3.988490000000	4.632516000000	11.148029000000
H	3.464188000000	6.332987000000	11.247072000000
H	7.400649000000	9.661885000000	8.247927000000
C	2.884310000000	7.342986000000	13.467502000000
H	2.249204000000	6.947070000000	12.651331000000
H	3.452539000000	8.212117000000	13.085517000000
H	2.202914000000	7.706907000000	14.264896000000
C	4.421645000000	6.826073000000	15.360432000000
H	3.610563000000	7.157386000000	16.043355000000
H	5.073282000000	7.692797000000	15.142515000000
H	5.026319000000	6.075556000000	15.898899000000
C	2.980151000000	5.009138000000	14.364343000000
H	2.575673000000	4.546358000000	13.441759000000
H	2.114190000000	5.290372000000	15.001291000000
H	3.556734000000	4.243486000000	14.917512000000
C	3.811649000000	6.265955000000	14.066687000000
C	6.590509000000	4.321524000000	14.657740000000
H	5.748786000000	4.190909000000	15.364257000000
H	7.127081000000	5.255062000000	14.920238000000
H	7.289329000000	3.473534000000	14.814091000000
C	5.308065000000	3.067991000000	12.885849000000
H	5.036318000000	2.997070000000	11.814412000000
H	4.382292000000	2.991275000000	13.482950000000
H	5.930856000000	2.178216000000	13.121182000000
C	7.363014000000	4.349207000000	12.280185000000
H	7.937574000000	3.409308000000	12.420320000000
H	8.033903000000	5.193681000000	12.528377000000
H	7.096460000000	4.418237000000	11.207730000000
C	6.122069000000	4.335623000000	13.193401000000
H	6.106208000000	10.038923000000	9.421826000000
C	6.299886000000	7.929441000000	9.024325000000

H	6.968435000000	7.279420000000	8.406896000000
H	5.358014000000	8.012951000000	8.429176000000
C	5.231370000000	6.070207000000	10.052770000000
H	5.911489000000	5.230015000000	9.766653000000
C	6.909548000000	9.319348000000	9.179550000000

Cartesian Coordinates of ^{1}3” GS

W	6.882601000000	8.035707000000	12.175735000000
N	8.264986000000	7.900577000000	13.524461000000
N	9.195244000000	7.859460000000	14.284399000000
W	10.581943000000	7.861999000000	15.635440000000
C	11.389046000000	9.478170000000	15.049468000000
O	11.946097000000	10.502410000000	14.719834000000
C	6.115118000000	9.627189000000	12.872067000000
O	5.583067000000	10.640001000000	13.271193000000
N	11.419306000000	7.191356000000	17.424346000000
P	9.298216000000	9.086494000000	17.347584000000
P	12.224507000000	6.132006000000	14.836687000000
H	12.550138000000	5.774384000000	18.523233000000
C	8.278949000000	6.538768000000	17.858650000000
H	8.509011000000	6.317821000000	16.796776000000
H	7.424876000000	5.891299000000	18.151030000000
H	9.151840000000	6.242846000000	18.467289000000
C	6.611256000000	8.253138000000	17.219947000000
H	6.810465000000	8.135290000000	16.136419000000
H	6.166581000000	9.252200000000	17.381315000000
H	5.844629000000	7.502190000000	17.503411000000
C	7.601724000000	8.246912000000	19.550460000000
H	6.772369000000	7.581250000000	19.874273000000
H	7.301260000000	9.285311000000	19.777270000000
H	8.482296000000	7.998774000000	20.175784000000
C	7.879324000000	8.016133000000	18.056388000000
C	9.948077000000	11.792163000000	17.125480000000
H	10.433035000000	11.873589000000	18.117873000000
H	9.626009000000	12.814023000000	16.832807000000
H	10.697131000000	11.452535000000	16.387445000000
C	7.795930000000	11.376229000000	18.299883000000
H	8.285625000000	11.283020000000	19.289998000000
H	6.823995000000	10.850595000000	18.339308000000
H	7.577576000000	12.453968000000	18.140375000000
C	7.986559000000	10.992419000000	15.802051000000
H	8.639419000000	10.627062000000	14.987722000000
H	7.732402000000	12.054092000000	15.597641000000
H	7.050356000000	10.409737000000	15.754343000000
C	8.702307000000	10.883941000000	17.159407000000
C	12.821722000000	5.381938000000	16.418878000000
H	12.865200000000	4.278560000000	16.339751000000
H	13.857199000000	5.732002000000	16.581424000000
H	10.165115000000	9.460874000000	19.668974000000
C	14.434381000000	7.774374000000	15.001414000000
H	14.825363000000	7.309126000000	15.927297000000
H	13.687979000000	8.539649000000	15.287801000000
H	15.285505000000	8.282706000000	14.501569000000
C	13.483393000000	7.464282000000	12.717910000000
H	14.387764000000	7.969004000000	12.317033000000
H	12.705007000000	8.233389000000	12.890551000000
H	13.118469000000	6.767660000000	11.940490000000
C	14.868518000000	5.632355000000	13.770632000000
H	15.047042000000	5.005982000000	14.667809000000
H	15.839008000000	6.095907000000	13.492098000000
H	14.572485000000	4.968125000000	12.938137000000
C	13.835540000000	6.740720000000	14.025605000000
C	10.858974000000	5.155765000000	12.588186000000
H	10.226301000000	6.050678000000	12.742217000000
H	10.229077000000	4.359835000000	12.138449000000
H	11.644415000000	5.410817000000	11.852243000000
C	12.369830000000	3.458463000000	13.675579000000
H	12.897698000000	3.131170000000	14.593470000000
H	13.126278000000	3.663047000000	12.896462000000
H	11.758435000000	2.600195000000	13.323238000000

C	10.288481000000	4.232206000000	14.840689000000
H	9.712681000000	3.417410000000	14.354180000000
H	9.587296000000	5.072061000000	15.024182000000
H	10.642462000000	3.850900000000	15.818423000000
C	11.447520000000	4.662226000000	13.918135000000
H	11.282499000000	9.935909000000	18.351713000000
C	11.272676000000	7.807541000000	18.746839000000
H	10.703334000000	7.125868000000	19.426690000000
H	12.275133000000	7.921945000000	19.228598000000
C	11.941821000000	5.826898000000	17.590784000000
H	11.106897000000	5.099105000000	17.745548000000
C	10.569198000000	9.158503000000	18.682483000000
N	6.034132000000	7.512573000000	10.343457000000
P	8.200585000000	9.345756000000	10.554841000000
P	5.193065000000	6.298426000000	12.850466000000
H	4.871496000000	6.204341000000	9.147203000000
C	9.160632000000	6.816676000000	9.868263000000
H	8.925429000000	6.527798000000	10.912501000000
H	9.998995000000	6.171044000000	9.529967000000
H	8.280923000000	6.584904000000	9.241913000000
C	10.868240000000	8.442406000000	10.622632000000
H	10.666991000000	8.251871000000	11.695447000000
H	11.335523000000	9.440111000000	10.532070000000
H	11.617629000000	7.696687000000	10.284545000000
C	9.879343000000	8.621596000000	8.298587000000
H	10.694072000000	7.961728000000	7.929037000000
H	10.202664000000	9.666332000000	8.143667000000
H	8.993942000000	8.436666000000	7.658371000000
C	9.595318000000	8.294315000000	9.773317000000
C	7.614309000000	12.044719000000	10.963819000000
H	7.133023000000	12.205299000000	9.979334000000
H	7.960447000000	13.036171000000	11.325531000000
H	6.856273000000	11.673647000000	11.677098000000
C	9.758540000000	11.660041000000	9.764990000000
H	9.269042000000	11.647647000000	8.770479000000
H	10.717848000000	11.115533000000	9.689719000000
H	10.001963000000	12.718581000000	9.999313000000
C	9.552860000000	11.108013000000	12.229966000000
H	8.888988000000	10.703157000000	13.016245000000
H	9.832485000000	12.146335000000	12.507930000000
H	10.474255000000	10.500390000000	12.237687000000
C	8.838077000000	11.111442000000	10.867870000000
C	4.581240000000	5.675994000000	11.219210000000
H	4.507570000000	4.571408000000	11.221962000000
H	3.556168000000	6.064500000000	11.079451000000
H	7.348617000000	9.902763000000	8.264665000000
C	3.029407000000	8.008534000000	12.798883000000
H	2.632741000000	7.620488000000	11.840407000000
H	3.795869000000	8.773685000000	12.571530000000
H	2.187982000000	8.500613000000	13.330435000000
C	3.965581000000	7.514127000000	15.055863000000
H	3.075224000000	8.017243000000	15.488806000000
H	4.767552000000	8.269237000000	14.938062000000
H	4.306630000000	6.755551000000	15.784633000000
C	2.533297000000	5.798746000000	13.876184000000
H	2.338469000000	5.244059000000	12.936209000000
H	1.575829000000	6.268029000000	14.188195000000
H	2.809650000000	5.068111000000	14.658291000000
C	3.597076000000	6.893235000000	13.700554000000
C	6.520930000000	5.128881000000	15.029650000000
H	5.738306000000	5.348715000000	15.779835000000
H	7.176061000000	6.016660000000	14.942957000000
H	7.129154000000	4.287093000000	15.422134000000
C	4.972385000000	3.554145000000	13.818049000000
H	4.442574000000	3.306166000000	12.876728000000
H	4.216139000000	3.723214000000	14.605839000000
H	5.559174000000	2.657637000000	14.112192000000
C	7.080130000000	4.354720000000	12.719564000000
H	7.628400000000	3.488983000000	13.146276000000
H	7.806253000000	5.185380000000	12.604706000000
H	6.723325000000	4.058871000000	11.713680000000
C	5.926947000000	4.747522000000	13.665683000000

H	6.239403000000	10.310272000000	9.611046000000
C	6.199971000000	8.215468000000	9.067192000000
H	6.755291000000	7.569327000000	8.342512000000
H	5.202210000000	8.387107000000	8.592739000000
C	5.477102000000	6.176852000000	10.082466000000
H	6.293557000000	5.439812000000	9.880182000000
C	6.935023000000	9.541349000000	9.227042000000

4.6 Cartesian coordinates of the transition state structures (Å)

Cartesian Coordinates of ^{3}3 TS

W	0.039036000000	0.040697000000	0.392887000000
N	0.160596000000	-0.013223000000	2.139596000000
N	-0.372262000000	-0.227257000000	4.035465000000
W	-0.846421000000	0.195083000000	5.668124000000
N	-1.210079000000	2.252424000000	5.970673000000
P	-3.335742000000	0.300148000000	5.519874000000
C	-0.737820000000	-1.537483000000	6.641664000000
O	-0.659018000000	-2.582226000000	7.227637000000
P	1.361924000000	0.981444000000	6.471431000000
N	-1.926301000000	-0.150696000000	-0.355215000000
P	-0.570517000000	2.419426000000	-0.054627000000
C	1.937056000000	0.163415000000	-0.194361000000
O	3.086318000000	0.224056000000	-0.535187000000
P	-0.107699000000	-2.399008000000	-0.015172000000
H	-3.657946000000	-1.364210000000	-0.350452000000
C	-0.044067000000	1.944713000000	-2.723154000000
H	0.654538000000	1.146498000000	-2.405215000000
H	0.274335000000	2.301798000000	-3.725542000000
H	-1.050852000000	1.497518000000	-2.827764000000
C	1.429863000000	3.632666000000	-1.636631000000
H	2.098819000000	2.864958000000	-1.199683000000
H	1.515231000000	4.558329000000	-1.036319000000
H	1.800212000000	3.863744000000	-2.657786000000
C	-0.940998000000	4.235406000000	-2.267110000000
H	-0.542753000000	4.594943000000	-3.239803000000
H	-1.004263000000	5.106917000000	-1.590816000000
H	-1.968438000000	3.866293000000	-2.453800000000
C	-0.019259000000	3.125918000000	-1.733276000000
C	-1.049447000000	3.182685000000	2.582294000000
H	-2.132542000000	3.373457000000	2.450777000000
H	-0.722519000000	3.769682000000	3.464933000000
H	-0.894574000000	2.113013000000	2.824565000000
C	-0.593264000000	5.085287000000	1.017052000000
H	-1.651731000000	5.179259000000	0.700997000000
H	0.048897000000	5.511777000000	0.223036000000
H	-0.460182000000	5.715532000000	1.922134000000
C	1.266563000000	3.525808000000	1.745154000000
H	1.522295000000	2.481950000000	2.007975000000
H	1.457786000000	4.167679000000	2.631709000000
H	1.943740000000	3.858411000000	0.938120000000
C	-0.222075000000	3.634680000000	1.363666000000
C	-1.935666000000	-2.621637000000	-0.001677000000
H	-2.279098000000	-3.577891000000	-0.441736000000
H	-2.212188000000	-2.618793000000	1.069139000000
H	-2.881959000000	3.143390000000	-0.686149000000
C	-0.304900000000	-3.314228000000	2.612532000000
H	-1.307006000000	-3.772774000000	2.504985000000
H	-0.414008000000	-2.243102000000	2.875157000000
H	0.191669000000	-3.812527000000	3.471691000000
C	1.998270000000	-3.059302000000	1.702115000000
H	2.391393000000	-3.686415000000	2.530481000000
H	1.990163000000	-2.003561000000	2.035666000000
H	2.694869000000	-3.150112000000	0.848634000000
C	0.547788000000	-5.017314000000	0.995167000000
H	-0.470041000000	-5.362522000000	0.723637000000
H	0.870462000000	-5.605175000000	1.880716000000
H	1.236218000000	-5.268891000000	0.166559000000
C	0.568503000000	-3.523925000000	1.361391000000
C	1.986971000000	-3.228243000000	-1.722668000000
H	2.283288000000	-4.105806000000	-1.117987000000

H	2.528809000000	-2.340010000000	-1.342567000000
H	2.328292000000	-3.412084000000	-2.763470000000
C	-0.271344000000	-4.259732000000	-2.210363000000
H	-1.357620000000	-4.081170000000	-2.333523000000
H	-0.138245000000	-5.122042000000	-1.532637000000
H	0.126307000000	-4.552084000000	-3.205693000000
C	0.174243000000	-1.852833000000	-2.721797000000
H	0.500474000000	-2.161444000000	-3.737737000000
H	0.731364000000	-0.937519000000	-2.444757000000
H	-0.900214000000	-1.595162000000	-2.774547000000
C	0.465165000000	-2.997164000000	-1.730989000000
H	-2.753577000000	2.303139000000	0.886370000000
C	-2.787277000000	0.951336000000	-0.789638000000
H	-2.801327000000	1.034794000000	-1.906812000000
C	-2.525951000000	2.894877000000	5.986850000000
H	-2.445708000000	3.907318000000	5.520077000000
H	-2.864920000000	3.091437000000	7.036479000000
H	-3.844840000000	0.723384000000	-0.508696000000
C	-2.589460000000	-1.415917000000	-0.677690000000
H	-2.641873000000	-1.573370000000	-1.785774000000
C	-3.589351000000	2.100810000000	5.226318000000
H	-3.424382000000	2.239794000000	4.142402000000
H	-4.612239000000	2.452258000000	5.461688000000
C	-0.202947000000	3.240852000000	6.362155000000
H	-0.316332000000	3.527751000000	7.439270000000
H	-0.381798000000	4.190284000000	5.798234000000
C	-3.393032000000	0.399530000000	8.279066000000
H	-2.424199000000	-0.136780000000	8.280393000000
H	-3.902049000000	0.193592000000	9.244519000000
H	-3.184061000000	1.485193000000	8.231767000000
C	-4.474235000000	-1.603383000000	7.269098000000
H	-3.513814000000	-2.142141000000	7.146243000000
H	-5.203990000000	-2.012922000000	6.545051000000
H	-4.858253000000	-1.822462000000	8.287814000000
C	-5.646423000000	0.633511000000	7.217630000000
H	-6.133498000000	0.350856000000	8.175156000000
H	-6.337470000000	0.361090000000	6.399596000000
H	-5.533691000000	1.735313000000	7.223363000000
C	-4.289108000000	-0.083467000000	7.121667000000
C	-3.554643000000	0.092911000000	2.749201000000
H	-3.973301000000	1.106406000000	2.593082000000
H	-3.853295000000	-0.518797000000	1.873367000000
H	-2.448500000000	0.150988000000	2.746964000000
C	-5.639931000000	-0.503201000000	4.003439000000
H	-6.004797000000	0.542750000000	4.047130000000
H	-6.104668000000	-1.071654000000	4.831427000000
H	-6.009559000000	-0.943883000000	3.053027000000
C	-3.628443000000	-2.044181000000	4.017956000000
H	-2.523031000000	-2.090715000000	4.014914000000
H	-4.002860000000	-2.541240000000	3.097437000000
H	-3.999366000000	-2.620191000000	4.884642000000
C	-4.104981000000	-0.579093000000	4.021648000000
C	1.227634000000	2.777092000000	6.085124000000
H	1.974131000000	3.409329000000	6.603764000000
H	1.417312000000	2.846559000000	4.997784000000
C	-2.404985000000	2.292932000000	-0.162291000000
C	2.795731000000	0.907936000000	4.080050000000
H	3.017728000000	1.991952000000	4.039083000000
H	1.811376000000	0.713316000000	3.609686000000
H	3.561110000000	0.394204000000	3.460960000000
C	2.787559000000	-1.186053000000	5.426754000000
H	3.651054000000	-1.564327000000	4.839141000000
H	1.854926000000	-1.487287000000	4.911570000000
H	2.812190000000	-1.675022000000	6.417857000000
C	4.217525000000	0.788071000000	6.131163000000
H	4.289411000000	1.890373000000	6.224073000000
H	5.041829000000	0.458511000000	5.463462000000
H	4.402700000000	0.339943000000	7.125538000000
C	2.879028000000	0.350379000000	5.513328000000
C	2.165586000000	-0.514580000000	8.731378000000
H	3.197094000000	-0.701870000000	8.378700000000
H	1.506893000000	-1.309497000000	8.329836000000

H	2.171124000000	-0.607648000000	9.838096000000
C	2.609158000000	1.971827000000	8.874292000000
H	2.205363000000	2.990363000000	8.711143000000
H	3.612856000000	1.921052000000	8.415401000000
H	2.735549000000	1.842789000000	9.970501000000
C	0.279132000000	1.076790000000	9.025383000000
H	0.404541000000	1.017590000000	10.127449000000
H	-0.427464000000	0.282965000000	8.716230000000
H	-0.180969000000	2.054441000000	8.788387000000
C	1.652470000000	0.886219000000	8.352225000000

Cartesian Coordinates of ^{1}3 TS

W	-0.134565000000	-0.008632000000	0.160485000000
N	-0.668457000000	0.076945000000	1.825665000000
N	-0.547682000000	0.726392000000	3.509374000000
W	-0.804290000000	0.519777000000	5.233334000000
N	-1.205280000000	2.325922000000	6.276630000000
P	-3.287951000000	0.526722000000	5.321203000000
C	-0.659802000000	-1.440308000000	5.478065000000
O	-0.592798000000	-2.609038000000	5.709199000000
P	1.464134000000	1.147420000000	6.052761000000
N	-1.833433000000	-0.320114000000	-1.064006000000
P	-0.540730000000	2.250475000000	-0.816054000000
C	1.828868000000	0.266309000000	0.355885000000
O	3.006692000000	0.420324000000	0.442181000000
P	-0.051741000000	-2.466635000000	-0.211906000000
H	-3.472073000000	-1.624463000000	-1.380814000000
C	-0.300802000000	1.601946000000	-3.512559000000
H	-0.364207000000	0.569033000000	-3.121151000000
H	0.325207000000	1.591527000000	-4.429480000000
H	-1.317771000000	1.924616000000	-3.806991000000
C	1.823208000000	2.219098000000	-2.350060000000
H	1.966529000000	1.150647000000	-2.100302000000
H	2.332948000000	2.814026000000	-1.569915000000
H	2.331392000000	2.414279000000	-3.318285000000
C	0.175586000000	3.996585000000	-3.018754000000
H	0.588404000000	4.038219000000	-4.049430000000
H	0.733213000000	4.735844000000	-2.413342000000
H	-0.885157000000	4.311647000000	-3.075112000000
C	0.329471000000	2.563792000000	-2.486653000000
C	-1.008989000000	3.383205000000	1.659811000000
H	-2.096691000000	3.195469000000	1.572597000000
H	-0.878653000000	4.228678000000	2.368660000000
H	-0.552930000000	2.481170000000	2.110824000000
C	-1.131575000000	5.016997000000	-0.214134000000
H	-2.198417000000	4.801682000000	-0.422468000000
H	-0.681473000000	5.449406000000	-1.123464000000
H	-1.104960000000	5.799734000000	0.573870000000
C	1.105661000000	4.079081000000	0.523178000000
H	1.651952000000	3.174943000000	0.857887000000
H	1.210840000000	4.852546000000	1.313203000000
H	1.593751000000	4.469527000000	-0.390794000000
C	-0.386594000000	3.774246000000	0.308754000000
C	-1.813490000000	-2.785470000000	-0.642810000000
H	-1.984430000000	-3.763052000000	-1.134431000000
H	-2.338560000000	-2.798752000000	0.330476000000
H	-2.655977000000	2.881595000000	-1.975070000000
C	-0.780220000000	-3.293715000000	2.337316000000
H	-1.770802000000	-3.683414000000	2.032853000000
H	-0.869316000000	-2.210370000000	2.549789000000
H	-0.500457000000	-3.799364000000	3.283416000000
C	1.656561000000	-3.136392000000	1.909792000000
H	1.813733000000	-3.700144000000	2.853529000000
H	1.641252000000	-2.057275000000	2.152488000000
H	2.521273000000	-3.332487000000	1.251753000000
C	0.322205000000	-5.065767000000	0.945192000000
H	-0.624541000000	-5.387294000000	0.466335000000
H	0.434695000000	-5.650498000000	1.882831000000
H	1.162635000000	-5.345404000000	0.281600000000
C	0.314089000000	-3.568046000000	1.290534000000

C	2.436616000000	-3.202191000000	-1.346885000000
H	2.602080000000	-4.094036000000	-0.713602000000
H	2.835467000000	-2.314306000000	-0.816981000000
H	3.032239000000	-3.338813000000	-2.274205000000
C	0.408834000000	-4.288203000000	-2.392946000000
H	-0.616509000000	-4.139609000000	-2.784483000000
H	0.395575000000	-5.157748000000	-1.710984000000
H	1.054805000000	-4.550522000000	-3.257834000000
C	0.879679000000	-1.857436000000	-2.742507000000
H	1.466434000000	-2.121501000000	-3.647805000000
H	1.307843000000	-0.926042000000	-2.320708000000
H	-0.158171000000	-1.644174000000	-3.059299000000
C	0.958464000000	-3.014326000000	-1.729077000000
H	-2.841247000000	2.413354000000	-0.260731000000
C	-2.711117000000	0.712955000000	-1.615220000000
H	-2.750115000000	0.641796000000	-2.731344000000
C	-2.534585000000	2.860084000000	6.568689000000
H	-2.520871000000	3.975655000000	6.474904000000
H	-2.829421000000	2.673070000000	7.635689000000
H	-3.763435000000	0.517835000000	-1.282541000000
C	-2.356773000000	-1.621758000000	-1.476175000000
H	-2.168603000000	-1.809784000000	-2.567231000000
C	-3.610409000000	2.313823000000	5.629456000000
H	-3.496965000000	2.797016000000	4.641257000000
H	-4.632237000000	2.527879000000	5.998382000000
C	-0.216193000000	3.119374000000	7.004991000000
H	-0.254872000000	2.914214000000	8.107790000000
H	-0.467599000000	4.206337000000	6.914987000000
C	-3.044382000000	-0.290213000000	7.963367000000
H	-2.096953000000	-0.800483000000	7.706255000000
H	-3.469270000000	-0.789446000000	8.860278000000
H	-2.804285000000	0.754482000000	8.236739000000
C	-4.250865000000	-1.874546000000	6.472966000000
H	-3.314217000000	-2.329287000000	6.094909000000
H	-5.049772000000	-2.045006000000	5.726943000000
H	-4.539750000000	-2.416421000000	7.398558000000
C	-5.389675000000	0.234621000000	7.271204000000
H	-5.790444000000	-0.364855000000	8.116437000000
H	-6.159419000000	0.248707000000	6.478360000000
H	-5.255213000000	1.270021000000	7.640898000000
C	-4.060009000000	-0.384517000000	6.806849000000
C	-3.833857000000	1.191940000000	2.675538000000
H	-4.304154000000	2.172742000000	2.885220000000
H	-4.187631000000	0.857582000000	1.677266000000
H	-2.735673000000	1.308846000000	2.609822000000
C	-5.743947000000	0.098838000000	3.875772000000
H	-6.130613000000	1.053666000000	4.285771000000
H	-6.096023000000	-0.726799000000	4.523050000000
H	-6.209311000000	-0.045358000000	2.877199000000
C	-3.690556000000	-1.228482000000	3.182291000000
H	-2.608653000000	-1.152745000000	2.966031000000
H	-4.210686000000	-1.468109000000	2.229875000000
H	-3.856493000000	-2.068056000000	3.880908000000
C	-4.215986000000	0.118327000000	3.712236000000
C	1.209601000000	2.920023000000	6.486034000000
H	1.966138000000	3.316650000000	7.190644000000
H	1.319017000000	3.467430000000	5.531874000000
C	-2.336540000000	2.145523000000	-1.210008000000
C	2.459834000000	2.173172000000	3.657679000000
H	2.591595000000	3.213783000000	4.013710000000
H	1.419540000000	2.034491000000	3.301067000000
H	3.133174000000	2.031602000000	2.787535000000
C	2.823616000000	-0.253797000000	4.052530000000
H	3.580807000000	-0.262040000000	3.240834000000
H	1.833378000000	-0.454390000000	3.600672000000
H	3.061542000000	-1.077273000000	4.749653000000
C	4.218340000000	1.460063000000	5.298317000000
H	4.230878000000	2.434539000000	5.827121000000
H	4.942805000000	1.527977000000	4.458952000000
H	4.590766000000	0.680846000000	5.990554000000
C	2.828933000000	1.129072000000	4.730764000000
C	2.611734000000	-1.078885000000	7.352643000000

H	3.593550000000	-1.020079000000	6.845508000000
H	1.923662000000	-1.674302000000	6.720323000000
H	2.761140000000	-1.629986000000	8.305296000000
C	3.030495000000	1.142735000000	8.479413000000
H	2.598363000000	2.109244000000	8.804777000000
H	3.965809000000	1.350345000000	7.928936000000
H	3.300913000000	0.580687000000	9.398764000000
C	0.750052000000	0.113190000000	8.504341000000
H	1.014718000000	-0.384708000000	9.461326000000
H	0.019595000000	-0.529741000000	7.976181000000
H	0.251180000000	1.070703000000	8.747427000000
C	2.027555000000	0.309751000000	7.663790000000

Cartesian Coordinates of ^{3}3' TS

W	0.072084000000	0.340191000000	-0.036766000000
N	-0.252177000000	0.277715000000	1.672670000000
N	-0.388385000000	0.589835000000	3.681347000000
W	-0.370201000000	-0.121208000000	5.277229000000
C	0.019684000000	-2.029259000000	4.912420000000
O	0.241946000000	-3.200065000000	4.784483000000
C	2.004576000000	0.841647000000	-0.247974000000
O	3.162748000000	1.140575000000	-0.363351000000
N	-0.731639000000	1.300413000000	6.833266000000
P	1.945116000000	0.449908000000	6.003972000000
P	-2.786649000000	-0.508643000000	5.797132000000
H	-2.089631000000	2.388460000000	8.045095000000
C	2.250312000000	2.641310000000	4.357482000000
H	1.193578000000	2.456833000000	4.081496000000
H	2.749804000000	3.123826000000	3.491329000000
H	2.291260000000	3.353270000000	5.205313000000
C	2.984963000000	0.461207000000	3.388994000000
H	1.957490000000	0.290139000000	3.015502000000
H	3.473575000000	-0.519470000000	3.538786000000
H	3.545452000000	1.001489000000	2.597033000000
C	4.413147000000	1.627829000000	5.116421000000
H	4.900670000000	2.256469000000	4.341057000000
H	5.025186000000	0.712231000000	5.226285000000
H	4.448267000000	2.192519000000	6.070014000000
C	2.976706000000	1.318247000000	4.667727000000
C	1.933483000000	-1.617604000000	7.815419000000
H	1.467402000000	-0.934714000000	8.553865000000
H	2.457919000000	-2.417428000000	8.380054000000
H	1.119985000000	-2.082779000000	7.227194000000
C	4.082661000000	-0.343366000000	7.784290000000
H	3.718612000000	0.384611000000	8.535614000000
H	4.883415000000	0.138997000000	7.197124000000
H	4.541651000000	-1.186961000000	8.343102000000
C	3.502119000000	-1.894019000000	5.878712000000
H	2.713156000000	-2.245513000000	5.185484000000
H	3.910487000000	-2.778805000000	6.411807000000
H	4.325212000000	-1.457512000000	5.280644000000
C	2.945260000000	-0.893201000000	6.904627000000
C	-3.133213000000	0.740791000000	7.116528000000
H	-4.161746000000	1.146510000000	7.043099000000
H	-3.058415000000	0.208289000000	8.084214000000
H	2.457663000000	2.487385000000	7.332298000000
C	-2.128853000000	-2.438228000000	7.639825000000
H	-2.031295000000	-1.641068000000	8.403890000000
H	-1.150717000000	-2.540161000000	7.132926000000
H	-2.347401000000	-3.388947000000	8.170712000000
C	-3.296609000000	-3.269666000000	5.581413000000
H	-3.382980000000	-4.251491000000	6.093524000000
H	-2.373341000000	-3.282870000000	4.970322000000
H	-4.165830000000	-3.172686000000	4.902523000000
C	-4.592834000000	-2.096058000000	7.402770000000
H	-4.604417000000	-1.295532000000	8.168250000000
H	-4.734848000000	-3.060067000000	7.936674000000
H	-5.464927000000	-1.952971000000	6.741197000000
C	-3.260589000000	-2.148371000000	6.633103000000
C	-3.489044000000	-0.836822000000	3.099009000000
H	-2.453265000000	-0.554611000000	2.827148000000

H	-4.157248000000	-0.561843000000	2.254491000000
H	-3.530808000000	-1.935849000000	3.215416000000
C	-5.430099000000	-0.401082000000	4.657771000000
H	-5.783125000000	0.074127000000	5.595083000000
H	-5.628485000000	-1.488260000000	4.717966000000
H	-6.051453000000	0.000410000000	3.828898000000
C	-3.800247000000	1.422758000000	4.119944000000
H	-4.336211000000	1.691874000000	3.185544000000
H	-2.735577000000	1.696318000000	3.991058000000
H	-4.235629000000	2.026069000000	4.940413000000
C	-3.955079000000	-0.090255000000	4.362831000000
H	1.728351000000	1.175305000000	8.295598000000
C	0.268074000000	2.311409000000	7.178475000000
H	0.286638000000	3.139547000000	6.424541000000
H	-0.009240000000	2.803054000000	8.144380000000
C	-2.070698000000	1.840837000000	7.069834000000
H	-2.345362000000	2.606877000000	6.300286000000
C	1.667065000000	1.711015000000	7.328781000000
N	-1.727086000000	-0.172632000000	-1.009214000000
P	0.509472000000	-2.026988000000	-0.778548000000
P	-0.860556000000	2.574799000000	-0.678150000000
H	-3.785460000000	0.318906000000	-1.222520000000
C	-0.926815000000	-3.132096000000	1.303612000000
H	-1.168544000000	-2.083044000000	1.563956000000
H	-0.940457000000	-3.718638000000	2.245242000000
H	-1.714729000000	-3.538601000000	0.639099000000
C	1.518363000000	-2.793704000000	1.714750000000
H	1.301200000000	-1.770448000000	2.075761000000
H	2.549925000000	-2.817276000000	1.318704000000
H	1.468741000000	-3.473242000000	2.589134000000
C	0.726561000000	-4.701093000000	0.259299000000
H	0.555111000000	-5.354473000000	1.140825000000
H	1.767585000000	-4.870710000000	-0.075573000000
H	0.037857000000	-5.039097000000	-0.541163000000
C	0.475845000000	-3.243590000000	0.674574000000
C	1.978751000000	-1.232567000000	-2.985793000000
H	1.027784000000	-1.128866000000	-3.545882000000
H	2.782524000000	-1.425116000000	-3.727512000000
H	2.186240000000	-0.268029000000	-2.486477000000
C	1.749821000000	-3.708164000000	-2.762294000000
H	0.814372000000	-3.709245000000	-3.354840000000
H	1.751039000000	-4.602402000000	-2.115503000000
H	2.590520000000	-3.815357000000	-3.480385000000
C	3.275592000000	-2.432566000000	-1.210263000000
H	3.408767000000	-1.534484000000	-0.576489000000
H	4.112429000000	-2.460899000000	-1.939677000000
H	3.363485000000	-3.331506000000	-0.570626000000
C	1.942772000000	-2.397217000000	-1.976187000000
C	-2.600254000000	2.133282000000	-1.140237000000
H	-3.328480000000	2.850000000000	-0.713074000000
H	-2.680763000000	2.198304000000	-2.241953000000
H	-1.240601000000	-3.463085000000	-1.800988000000
C	0.035730000000	2.177843000000	-3.239425000000
H	-0.913486000000	1.654462000000	-3.469374000000
H	0.735868000000	1.428353000000	-2.820733000000
H	0.455953000000	2.562192000000	-4.192630000000
C	1.203549000000	3.992252000000	-1.968524000000
H	1.682854000000	4.286529000000	-2.925988000000
H	1.880111000000	3.283115000000	-1.452855000000
H	1.112160000000	4.905743000000	-1.349862000000
C	-1.102371000000	4.386030000000	-2.913484000000
H	-2.103039000000	3.966051000000	-3.135562000000
H	-0.661907000000	4.706811000000	-3.881540000000
H	-1.237674000000	5.290746000000	-2.295379000000
C	-0.163732000000	3.354053000000	-2.263593000000
C	0.416082000000	3.919200000000	1.402953000000
H	1.177320000000	4.357321000000	0.730884000000
H	0.766109000000	2.920684000000	1.727648000000
H	0.342850000000	4.568088000000	2.301341000000
C	-1.475570000000	5.202800000000	0.313544000000
H	-2.446937000000	5.150203000000	-0.218343000000
H	-0.749868000000	5.735013000000	-0.330815000000

H	-1.627793000000	5.825047000000	1.220963000000
C	-1.944955000000	3.211028000000	1.769217000000
H	-1.955947000000	3.857776000000	2.671732000000
H	-1.619515000000	2.200657000000	2.085088000000
H	-2.983850000000	3.159549000000	1.388407000000
C	-0.971362000000	3.817368000000	0.743367000000
H	-0.665430000000	-2.152422000000	-2.864269000000
C	-2.149084000000	-1.501311000000	-1.430508000000
H	-2.731559000000	-1.990326000000	-0.610545000000
H	-2.857055000000	-1.428454000000	-2.292162000000
C	-2.874307000000	0.688939000000	-0.698739000000
H	-3.103014000000	0.659269000000	0.393443000000
C	-0.972202000000	-2.388485000000	-1.827106000000

Cartesian Coordinates of ^{1}3' TS

W	-0.116726000000	0.308182000000	0.008654000000
N	-0.627384000000	0.134943000000	1.678585000000
N	-0.413543000000	0.422083000000	3.432013000000
W	-0.297540000000	-0.166903000000	5.081943000000
C	0.055431000000	-2.109083000000	4.873109000000
O	0.245767000000	-3.286593000000	4.833332000000
C	1.792837000000	0.836513000000	0.140795000000
O	2.935480000000	1.181268000000	0.139188000000
N	-0.616242000000	1.416182000000	6.467570000000
P	2.032870000000	0.467754000000	5.762123000000
P	-2.665245000000	-0.556565000000	5.789715000000
H	-1.909479000000	2.561541000000	7.695055000000
C	2.480572000000	2.290369000000	3.742534000000
H	1.424523000000	2.097181000000	3.471992000000
H	3.019331000000	2.569386000000	2.813102000000
H	2.527197000000	3.155571000000	4.432959000000
C	3.146863000000	-0.065139000000	3.257478000000
H	2.127292000000	-0.225202000000	2.860450000000
H	3.531935000000	-1.031461000000	3.630336000000
H	3.789131000000	0.259823000000	2.414067000000
C	4.573656000000	1.364635000000	4.775505000000
H	5.113870000000	1.814159000000	3.915405000000
H	5.138965000000	0.464136000000	5.083065000000
H	4.602240000000	2.099160000000	5.605475000000
C	3.140091000000	1.030370000000	4.336190000000
C	1.842135000000	-1.237735000000	7.896858000000
H	1.361241000000	-0.412427000000	8.458840000000
H	2.298142000000	-1.933084000000	8.632831000000
H	1.044003000000	-1.781459000000	7.356695000000
C	4.056744000000	-0.082232000000	7.750023000000
H	3.699124000000	0.780985000000	8.344620000000
H	4.901294000000	0.255955000000	7.124373000000
H	4.452953000000	-0.831460000000	8.468355000000
C	3.481247000000	-1.930453000000	6.134396000000
H	2.706888000000	-2.376316000000	5.480144000000
H	3.822413000000	-2.713778000000	6.843966000000
H	4.350239000000	-1.648496000000	5.509086000000
C	2.930487000000	-0.734710000000	6.928400000000
C	-2.948076000000	0.792754000000	7.021415000000
H	-3.998838000000	1.144780000000	7.016003000000
H	-2.752261000000	0.351382000000	8.017863000000
H	2.556296000000	2.722394000000	6.649850000000
C	-1.867681000000	-2.350584000000	7.728218000000
H	-1.671264000000	-1.476204000000	8.380743000000
H	-0.943214000000	-2.565622000000	7.162713000000
H	-2.078519000000	-3.220824000000	8.385268000000
C	-3.194982000000	-3.328014000000	5.838769000000
H	-3.244320000000	-4.262877000000	6.436507000000
H	-2.324726000000	-3.404935000000	5.158828000000
H	-4.115631000000	-3.277801000000	5.226354000000
C	-4.335544000000	-1.993564000000	7.653061000000
H	-4.268727000000	-1.151911000000	8.369749000000
H	-4.454045000000	-2.922310000000	8.251186000000
H	-5.254048000000	-1.865628000000	7.055003000000
C	-3.067893000000	-2.124787000000	6.788075000000
C	-3.538013000000	-1.106376000000	3.183548000000

H	-2.535837000000	-0.810247000000	2.816680000000
H	-4.268793000000	-0.917212000000	2.367638000000
H	-3.531387000000	-2.193787000000	3.384172000000
C	-5.388156000000	-0.596042000000	4.830338000000
H	-5.695960000000	-0.048500000000	5.743721000000
H	-5.555051000000	-1.677649000000	4.993789000000
H	-6.068153000000	-0.279243000000	4.010902000000
C	-3.851258000000	1.212583000000	4.049982000000
H	-4.473646000000	1.394524000000	3.149744000000
H	-2.810200000000	1.501036000000	3.807932000000
H	-4.233713000000	1.868225000000	4.856297000000
C	-3.943270000000	-0.278573000000	4.416458000000
H	1.852721000000	1.633173000000	7.875004000000
C	0.362438000000	2.500710000000	6.572999000000
H	0.355367000000	3.155483000000	5.664465000000
H	0.091146000000	3.172216000000	7.424583000000
C	-1.952172000000	1.925695000000	6.775664000000
H	-2.332047000000	2.603400000000	5.969510000000
C	1.771545000000	1.959391000000	6.820839000000
N	-1.747201000000	-0.077177000000	-1.303047000000
P	0.289778000000	-2.058512000000	-0.720592000000
P	-0.825177000000	2.617511000000	-0.640434000000
H	-3.406680000000	0.604469000000	-2.431312000000
C	-1.037571000000	-3.273784000000	1.367507000000
H	-1.296753000000	-2.242931000000	1.677295000000
H	-1.004772000000	-3.900417000000	2.282837000000
H	-1.840139000000	-3.670289000000	0.714713000000
C	1.401011000000	-2.866152000000	1.711494000000
H	1.135650000000	-1.879168000000	2.133291000000
H	2.415547000000	-2.799507000000	1.278678000000
H	1.429999000000	-3.592561000000	2.548709000000
C	0.633815000000	-4.749204000000	0.211450000000
H	0.511169000000	-5.442591000000	1.070444000000
H	1.670069000000	-4.867014000000	-0.159118000000
H	-0.064417000000	-5.079871000000	-0.583778000000
C	0.345437000000	-3.317832000000	0.688900000000
C	1.613837000000	-1.114950000000	-2.923323000000
H	0.632690000000	-1.043275000000	-3.433647000000
H	2.399356000000	-1.206399000000	-3.702916000000
H	1.775830000000	-0.164868000000	-2.379397000000
C	1.557313000000	-3.613841000000	-2.801294000000
H	0.590133000000	-3.668678000000	-3.338114000000
H	1.665675000000	-4.527892000000	-2.191471000000
H	2.358757000000	-3.629554000000	-3.570561000000
C	3.054706000000	-2.302595000000	-1.254074000000
H	3.152394000000	-1.419132000000	-0.593495000000
H	3.866509000000	-2.256007000000	-2.010607000000
H	3.218803000000	-3.214525000000	-0.648230000000
C	1.695418000000	-2.324650000000	-1.971857000000
C	-2.227533000000	2.300118000000	-1.802298000000
H	-2.999061000000	3.093541000000	-1.746026000000
H	-1.799595000000	2.326641000000	-2.823211000000
H	-1.562144000000	-3.500808000000	-1.516434000000
C	1.037627000000	2.696334000000	-2.674060000000
H	0.304820000000	2.140167000000	-3.292558000000
H	1.663813000000	1.956370000000	-2.143759000000
H	1.693288000000	3.270126000000	-3.362778000000
C	1.434826000000	4.304182000000	-0.792830000000
H	2.221451000000	4.761461000000	-1.429871000000
H	1.919757000000	3.550256000000	-0.143198000000
H	1.021191000000	5.105976000000	-0.151583000000
C	-0.354685000000	4.760839000000	-2.513496000000
H	-1.116203000000	4.338615000000	-3.197771000000
H	0.398634000000	5.279556000000	-3.144226000000
H	-0.842046000000	5.525132000000	-1.884079000000
C	0.359440000000	3.671145000000	-1.691798000000
C	-0.563518000000	3.611824000000	1.964662000000
H	0.407822000000	4.077401000000	1.714794000000
H	-0.378987000000	2.575287000000	2.308327000000
H	-1.003030000000	4.179340000000	2.813298000000
C	-1.913348000000	5.077763000000	0.406060000000
H	-2.591347000000	5.127497000000	-0.469543000000

H	-1.020917000000	5.697685000000	0.197256000000
H	-2.445116000000	5.545680000000	1.261897000000
C	-2.834817000000	2.901287000000	1.216221000000
H	-3.217369000000	3.374940000000	2.143701000000
H	-2.631384000000	1.835840000000	1.437611000000
H	-3.634992000000	2.969720000000	0.453698000000
C	-1.552542000000	3.633597000000	0.785319000000
H	-0.953129000000	-2.388235000000	-2.770995000000
C	-2.306328000000	-1.428122000000	-1.386651000000
H	-2.848985000000	-1.710647000000	-0.448489000000
H	-3.074742000000	-1.468974000000	-2.197616000000
C	-2.801605000000	0.908157000000	-1.540719000000
H	-3.530442000000	0.946034000000	-0.691639000000
C	-1.223741000000	-2.462220000000	-1.700734000000

Cartesian Coordinates of ^{3}3” TS

W	0.030906000000	-0.037410000000	-0.004965000000
N	-0.631142000000	0.076387000000	1.611468000000
N	-0.279998000000	0.435687000000	3.621307000000
W	-0.058626000000	-0.231294000000	5.224284000000
C	0.196791000000	-2.167609000000	4.928597000000
O	0.370242000000	-3.348770000000	4.793830000000
C	1.987389000000	-0.062739000000	0.260680000000
O	3.182874000000	-0.095797000000	0.374575000000
N	-0.057531000000	0.995951000000	6.964386000000
P	2.353659000000	0.351693000000	5.549683000000
P	-2.460905000000	-0.331824000000	5.991959000000
H	-1.102766000000	2.264705000000	8.309115000000
C	1.617948000000	2.971370000000	4.863047000000
H	0.724182000000	2.523426000000	4.391945000000
H	1.868035000000	3.908078000000	4.321197000000
H	1.372167000000	3.245963000000	5.905117000000
C	3.136130000000	1.801552000000	3.279642000000
H	2.303677000000	1.273130000000	2.773330000000
H	4.070037000000	1.232256000000	3.114512000000
H	3.258761000000	2.788515000000	2.785605000000
C	4.021729000000	2.697739000000	5.480848000000
H	4.217852000000	3.676363000000	4.992761000000
H	4.953481000000	2.108287000000	5.426316000000
H	3.803928000000	2.904848000000	6.547587000000
C	2.833620000000	2.025871000000	4.771057000000
C	3.455133000000	-2.087711000000	6.257738000000
H	3.789062000000	-1.806350000000	7.275473000000
H	4.041379000000	-2.978337000000	5.948926000000
H	2.388828000000	-2.385206000000	6.303573000000
C	5.135731000000	-0.454891000000	5.383374000000
H	5.317075000000	0.013857000000	6.371371000000
H	5.413012000000	0.268380000000	4.593166000000
H	5.828560000000	-1.318698000000	5.293328000000
C	3.467437000000	-1.530395000000	3.821048000000
H	2.427787000000	-1.885007000000	3.699535000000
H	4.153726000000	-2.389052000000	3.660664000000
H	3.658278000000	-0.791968000000	3.022006000000
C	3.692324000000	-0.959391000000	5.233038000000
C	-2.488743000000	0.879841000000	7.391198000000
H	-3.429383000000	1.465413000000	7.399690000000
H	-2.455727000000	0.296121000000	8.330784000000
H	3.275765000000	1.120532000000	7.738670000000
C	-1.900905000000	-2.392760000000	7.720400000000
H	-1.697987000000	-1.665425000000	8.531790000000
H	-0.963432000000	-2.527720000000	7.147249000000
H	-2.170577000000	-3.359869000000	8.194957000000
C	-3.288468000000	-3.001756000000	5.720875000000
H	-3.431077000000	-3.995083000000	6.196581000000
H	-2.421190000000	-3.070453000000	5.034771000000
H	-4.193169000000	-2.788117000000	5.119304000000
C	-4.322930000000	-1.779561000000	7.674902000000
H	-4.209726000000	-0.986914000000	8.440813000000
H	-4.509969000000	-2.732061000000	8.215427000000
H	-5.224658000000	-1.556050000000	7.078675000000

C	-3.060570000000	-1.940162000000	6.809595000000
C	-3.573392000000	-0.426265000000	3.415579000000
H	-2.541739000000	-0.362163000000	3.017903000000
H	-4.255429000000	0.024254000000	2.662686000000
H	-3.849128000000	-1.492246000000	3.515830000000
C	-5.165084000000	0.304314000000	5.231963000000
H	-5.292836000000	0.776536000000	6.226913000000
H	-5.557367000000	-0.729354000000	5.280855000000
H	-5.803482000000	0.863027000000	4.514677000000
C	-3.303931000000	1.812363000000	4.481163000000
H	-3.943493000000	2.218476000000	3.669385000000
H	-2.249216000000	1.878901000000	4.146920000000
H	-3.450946000000	2.454695000000	5.371871000000
C	-3.711528000000	0.346778000000	4.736996000000
H	2.322892000000	-0.390112000000	7.819960000000
C	1.096248000000	1.392889000000	7.762763000000
H	1.289766000000	2.491287000000	7.670210000000
H	0.885782000000	1.230630000000	8.849304000000
C	-1.244652000000	1.774780000000	7.318550000000
H	-1.411143000000	2.605563000000	6.591183000000
C	2.356916000000	0.621256000000	7.373600000000
N	-1.209733000000	-0.155868000000	-1.733285000000
P	-0.267467000000	-2.496475000000	-0.360880000000
P	-0.170196000000	2.372299000000	-0.725406000000
H	-2.597401000000	0.754183000000	-3.058098000000
C	-2.942936000000	-2.085147000000	0.376300000000
H	-2.595818000000	-1.150911000000	0.853946000000
H	-3.834711000000	-2.450956000000	0.928129000000
H	-3.261208000000	-1.860092000000	-0.657998000000
C	-1.575523000000	-3.475361000000	1.919032000000
H	-1.139925000000	-2.593180000000	2.429461000000
H	-0.897626000000	-4.337840000000	2.061081000000
H	-2.532599000000	-3.719831000000	2.426471000000
C	-2.398734000000	-4.431357000000	-0.281240000000
H	-3.338981000000	-4.748178000000	0.218753000000
H	-1.702983000000	-5.287755000000	-0.249804000000
H	-2.648070000000	-4.225267000000	-1.341261000000
C	-1.859403000000	-3.181804000000	0.436010000000
C	2.272361000000	-3.291478000000	-1.123724000000
H	2.014905000000	-3.641561000000	-2.142342000000
H	3.230915000000	-3.773064000000	-0.838248000000
H	2.441223000000	-2.196922000000	-1.156450000000
C	0.864875000000	-5.165901000000	-0.252018000000
H	0.404115000000	-5.387150000000	-1.235645000000
H	0.193035000000	-5.538420000000	0.544432000000
H	1.806031000000	-5.752601000000	-0.186503000000
C	1.762737000000	-3.404520000000	1.320363000000
H	1.994256000000	-2.332049000000	1.452873000000
H	2.699033000000	-3.986747000000	1.455986000000
H	1.066384000000	-3.692527000000	2.127983000000
C	1.197644000000	-3.675215000000	-0.085922000000
C	-1.387836000000	2.280101000000	-2.116376000000
H	-2.082993000000	3.142848000000	-2.101573000000
H	-0.812101000000	2.337068000000	-3.059758000000
H	-0.962035000000	-3.473099000000	-2.551030000000
C	1.926473000000	2.105794000000	-2.480863000000
H	1.221567000000	1.833895000000	-3.291805000000
H	2.180043000000	1.179575000000	-1.930123000000
H	2.849103000000	2.501403000000	-2.955745000000
C	2.382513000000	3.513047000000	-0.455222000000
H	3.347684000000	3.780731000000	-0.934951000000
H	2.559108000000	2.647392000000	0.213384000000
H	2.069483000000	4.374867000000	0.165433000000
C	1.023793000000	4.433423000000	-2.375841000000
H	0.243657000000	4.241072000000	-3.138778000000
H	1.941209000000	4.747309000000	-2.918298000000
H	0.697688000000	5.287772000000	-1.757547000000
C	1.345169000000	3.181597000000	-1.540658000000
C	-0.186033000000	3.445201000000	1.870134000000
H	0.833805000000	3.858824000000	1.764263000000
H	-0.110797000000	2.407298000000	2.249195000000
H	-0.711660000000	4.050012000000	2.640196000000

C	-1.136369000000	4.960568000000	0.090899000000
H	-1.632161000000	5.044003000000	-0.897291000000
H	-0.162493000000	5.483731000000	0.040343000000
H	-1.764628000000	5.509173000000	0.824759000000
C	-2.385015000000	2.909213000000	0.821316000000
H	-2.860453000000	3.477911000000	1.648055000000
H	-2.312435000000	1.849107000000	1.136710000000
H	-3.050795000000	2.987803000000	-0.060827000000
C	-0.986506000000	3.505198000000	0.559190000000
H	0.425439000000	-2.349381000000	-2.642453000000
C	-1.486703000000	-1.340108000000	-2.538164000000
H	-2.553418000000	-1.660663000000	-2.428123000000
H	-1.371547000000	-1.100963000000	-3.624844000000
C	-2.125652000000	0.936067000000	-2.065064000000
H	-2.969419000000	0.988444000000	-1.335219000000
C	-0.566854000000	-2.507101000000	-2.180097000000

Cartesian Coordinates of ^{1}3” TS

W	-0.068795000000	0.103822000000	0.024493000000
N	-0.643894000000	0.066164000000	1.697918000000
N	-0.345469000000	0.432467000000	3.419207000000
W	-0.035102000000	-0.167966000000	5.055311000000
C	0.467774000000	-2.038062000000	4.750201000000
O	0.816253000000	-3.175657000000	4.629948000000
C	1.850342000000	0.451538000000	0.226056000000
O	3.031960000000	0.626405000000	0.281627000000
N	-0.216434000000	0.957785000000	6.868032000000
P	2.258462000000	0.783391000000	5.391320000000
P	-2.379529000000	-0.669555000000	5.839130000000
H	-1.390591000000	1.847328000000	8.391542000000
C	1.118940000000	3.263724000000	4.756788000000
H	0.298299000000	2.684845000000	4.297216000000
H	1.206360000000	4.230050000000	4.216497000000
H	0.850548000000	3.491841000000	5.803988000000
C	2.781319000000	2.372141000000	3.142160000000
H	2.023137000000	1.743624000000	2.634884000000
H	3.782053000000	1.939843000000	2.954574000000
H	2.760089000000	3.375666000000	2.667937000000
C	3.543697000000	3.363757000000	5.344839000000
H	3.575293000000	4.367455000000	4.869419000000
H	4.555753000000	2.929678000000	5.268612000000
H	3.312190000000	3.519529000000	6.417186000000
C	2.466827000000	2.522594000000	4.638471000000
C	3.720817000000	-1.463418000000	6.063768000000
H	4.007331000000	-1.149291000000	7.086207000000
H	4.434698000000	-2.250989000000	5.744012000000
H	2.711917000000	-1.920132000000	6.103127000000
C	5.132150000000	0.419302000000	5.210330000000
H	5.241214000000	0.898654000000	6.203828000000
H	5.292037000000	1.186576000000	4.429086000000
H	5.950049000000	-0.325702000000	5.109071000000
C	3.641974000000	-0.881504000000	3.638279000000
H	2.664824000000	-1.383906000000	3.518948000000
H	4.445002000000	-1.629254000000	3.465843000000
H	3.718924000000	-0.114764000000	2.846898000000
C	3.783191000000	-0.298565000000	5.054776000000
C	-2.579601000000	0.393135000000	7.336167000000
H	-3.602441000000	0.814825000000	7.394206000000
H	-2.442387000000	-0.258118000000	8.219730000000
H	3.066848000000	1.682514000000	7.564771000000
C	-1.538543000000	-2.755447000000	7.428622000000
H	-1.450178000000	-2.068161000000	8.293370000000
H	-0.583900000000	-2.721487000000	6.872273000000
H	-1.673775000000	-3.782429000000	7.829031000000
C	-2.805868000000	-3.410937000000	5.365169000000
H	-2.796130000000	-4.446544000000	5.766322000000
H	-1.939597000000	-3.296909000000	4.685507000000
H	-3.732603000000	-3.293602000000	4.770725000000
C	-4.019808000000	-2.497091000000	7.379827000000
H	-4.036311000000	-1.748295000000	8.196253000000
H	-4.062837000000	-3.500276000000	7.855466000000

H	-4.940225000000	-2.376088000000	6.782823000000
C	-2.741376000000	-2.407929000000	6.527441000000
C	-3.546943000000	-0.735984000000	3.287329000000
H	-2.535566000000	-0.538389000000	2.881322000000
H	-4.287200000000	-0.315605000000	2.572778000000
H	-3.705151000000	-1.829460000000	3.320836000000
C	-5.168199000000	-0.321690000000	5.169503000000
H	-5.327837000000	0.051760000000	6.200949000000
H	-5.439543000000	-1.393954000000	5.138746000000
H	-5.881407000000	0.214932000000	4.507903000000
C	-3.514520000000	1.444068000000	4.505298000000
H	-4.220648000000	1.831749000000	3.741434000000
H	-2.485373000000	1.661774000000	4.156712000000
H	-3.706796000000	1.997640000000	5.445217000000
C	-3.740562000000	-0.073813000000	4.659177000000
H	2.371129000000	0.037623000000	7.660519000000
C	0.870541000000	1.592366000000	7.609451000000
H	0.868639000000	2.705914000000	7.490146000000
H	0.720434000000	1.427544000000	8.706043000000
C	-1.499161000000	1.475885000000	7.345029000000
H	-1.831495000000	2.362418000000	6.750775000000
C	2.237195000000	1.039368000000	7.211504000000
N	-1.276594000000	-0.156585000000	-1.724253000000
P	0.045607000000	-2.360239000000	-0.414323000000
P	-0.673444000000	2.459256000000	-0.644946000000
H	-2.695707000000	0.564046000000	-3.123895000000
C	-2.640452000000	-2.433791000000	0.386967000000
H	-2.441705000000	-1.468236000000	0.884810000000
H	-3.440779000000	-2.956966000000	0.951996000000
H	-3.025506000000	-2.237230000000	-0.629707000000
C	-1.025011000000	-3.620839000000	1.849104000000
H	-0.755920000000	-2.689203000000	2.384587000000
H	-0.192992000000	-4.342101000000	1.953624000000
H	-1.908966000000	-4.059143000000	2.357863000000
C	-1.726117000000	-4.633422000000	-0.364819000000
H	-2.583904000000	-5.121350000000	0.145689000000
H	-0.893855000000	-5.358535000000	-0.378892000000
H	-2.037349000000	-4.441280000000	-1.410766000000
C	-1.387122000000	-3.333192000000	0.384027000000
C	2.659839000000	-2.676951000000	-1.254061000000
H	2.438665000000	-3.027065000000	-2.281000000000
H	3.694648000000	-2.994689000000	-1.008394000000
H	2.637285000000	-1.569124000000	-1.244616000000
C	1.620892000000	-4.797196000000	-0.424185000000
H	1.180781000000	-5.062103000000	-1.406469000000
H	1.041706000000	-5.305370000000	0.370075000000
H	2.649621000000	-5.215794000000	-0.397256000000
C	2.243516000000	-2.955221000000	1.190325000000
H	2.284188000000	-1.862386000000	1.350355000000
H	3.272180000000	-3.362952000000	1.286973000000
H	1.631628000000	-3.386389000000	2.002582000000
C	1.696218000000	-3.278021000000	-0.210553000000
C	-1.822336000000	2.230064000000	-2.072637000000
H	-2.645977000000	2.970556000000	-2.047256000000
H	-1.240017000000	2.418302000000	-2.994019000000
H	-0.533736000000	-3.391069000000	-2.602590000000
C	1.458799000000	2.641628000000	-2.377183000000
H	0.817001000000	2.280188000000	-3.205133000000
H	1.881837000000	1.756080000000	-1.868384000000
H	2.294012000000	3.218953000000	-2.827148000000
C	1.645339000000	4.016744000000	-0.287084000000
H	2.554093000000	4.465865000000	-0.740914000000
H	1.962482000000	3.170180000000	0.351769000000
H	1.181998000000	4.786833000000	0.359645000000
C	0.163901000000	4.766172000000	-2.186957000000
H	-0.568665000000	4.477356000000	-2.966325000000
H	1.017544000000	5.253140000000	-2.705291000000
H	-0.304510000000	5.526750000000	-1.538558000000
C	0.690622000000	3.553570000000	-1.398497000000
C	-0.929019000000	3.458170000000	1.964664000000
H	-0.009956000000	4.067171000000	1.885713000000
H	-0.651881000000	2.446423000000	2.320021000000

H	-1.571522000000	3.926255000000	2.741435000000
C	-2.124438000000	4.807821000000	0.206514000000
H	-2.606148000000	4.826259000000	-0.791679000000
H	-1.268775000000	5.509306000000	0.195933000000
H	-2.861647000000	5.202691000000	0.937904000000
C	-2.968948000000	2.536830000000	0.857999000000
H	-3.563782000000	2.982854000000	1.682377000000
H	-2.702881000000	1.502590000000	1.153826000000
H	-3.617506000000	2.504988000000	-0.039362000000
C	-1.706085000000	3.395244000000	0.641734000000
H	0.631617000000	-2.038048000000	-2.709963000000
C	-1.417350000000	-1.379792000000	-2.509894000000
H	-2.409777000000	-1.873407000000	-2.349532000000
H	-1.402254000000	-1.127745000000	-3.600077000000
C	-2.331662000000	0.787847000000	-2.093310000000
H	-3.228304000000	0.682303000000	-1.434340000000
C	-0.299447000000	-2.378533000000	-2.219746000000

References

[1] S. Hoops, S. Sahle, R. Gauges, C. Lee, J. Pahle, N. Simus, M. Singhal, L. Xu, P. Mendes, U. Kummer COPASI- a COmplex PAthway Simulator, *Bioinformatics*, **2006**, *22*, 3067-3074.

[2] F. Schneck, J. Ahrens, M. Finger, A. C. Stückl, C. Würtele, D. Schwarzer, S. Schneider, The elusive abnormal CO_2 insertion enabled by metal-ligand cooperative photochemical selectivity inversion *Nat. Commun.* **2018**, *9*, 1161-1168.

[3] H. Vennekate, A. Walter, D. Fischer, J. Schroeder, D. Schwarzer Photodecarbonylation of Diphenylcyclopropenone – a Direct Pathway to Electronically Excited Diphenylacetylene? *Z. Phys. Chem.* **2011**, *225*, 1089-1104.

[4] R. A. Kaindl, M. Wurm, K. Reimann, P. Hamm, A. M. Weiner, M. Woerner Generation, shaping, and characterization of intense femtosecond pulses tunable from 3 to 20 µm *J. Opt. Soc. Am. B,* **2000**, *Vol. 17*, 12, 2086-2094.

[5] APEX3 v2016.9-0 (SAINT/SADABS/SHELXT/SHELXL), Bruker AXS Inc., Madison, WI, USA, **2016**.

[6] G.M. Sheldrick SHELXT – Integrated space-group and crystal-structure determination *Acta Cryst.,* **2015**, A71, 3-8.

[7] G.M. Sheldrick Crystal structure refinement with *SHELXL Acta Cryst*., **2015**, C71, 3-8.

[8] G.M. Sheldrick A short history of *SHELX Acta Cryst*., **2008**, A64, 112-122.

[9] F. Neese The ORCA program system *Wiley Interdiscip. Rev. Comput. Mol. Sci.* **2012**, *2*, 73-78.

[10] F. Neese Software update: the ORCA program system, version 4.0 *Wiley Interdiscip. Rev. Comput. Mol. Sci.* **2017,** 1327.

[11] J.P. Perdew, K. Burke, M. Ernzerhof Generalized Gradient Approximation Made Simple *Phys. Rev. Lett.* **1996**, *77*, 3385.

[12] F. Weigend, R. Ahlrichs Balanced basis sets of split valence, triple zeta valence and quadruple zeta valence quality for H to Rn: Design and assessment of accuracy *Phys. Chem. Chem. Phys.* **2005**, *7*, 3297.

[13] F. Weigend Accurate Coulomb-fitting basis sets for H to Rn *Phys. Chem. Chem. Phys.* **2006**, *8*, 1057.

[14] D. Andrae, U. Haeussermann, M. Dolg, H. Stoll, H. Preuss Energy-adjusted *ab initio* pseudopotentials for the second and third row transition elements *Theor. Chim. Acta* **1990**, *77*, 123.

[15] S. Grimme, J. Anthony, S. Ehrlich, H. Krieg A consistent and accurate *ab initio* parametrization of density functional dispersion correction (DFT-D) for the 94 elements H-Pu *J. Chem. Phys.* **2010**, *132*, 154104.

[16] S. Grimme, S. Ehrlich and L. Goerigk Effect of the damping function in dispersion corrected density functional theory *J. Comput. Chem.* **2011**, *32*, 1456-1465.

[17] B.I. Dunlap, J.W.D. Connolly, J. R. Sabin On some approximations in applications of Xα theory *J. Chem. Phys.* **1979**, *71*, 3396-3402.

[18] M. Feyereisen, G.Fitzgerald, A. Komornicki Use of approximate integrals in ab initio theory. An application in MP2 energy calculations *Chem. Phys. Lett.* **1993**, *208*, 359-363.

[19] C. Adamo, V. Barone Toward reliable density functional methods without adjustable parameters: The PBE0 model *J. Chem. Phys.* **1999**, *110*, 6158-6170.

[20] F. Neese, F. Wennmohs, A. Hansen, U. Becker Efficient, approximate and parallel Hartree–Fock and hybrid DFT calculations. A 'chain-of-spheres' algorithm for the Hartree–Fock exchange *Chem. Phys.* **2009**, *356*, 98-109.

[21] F. Neese, G. Olbrich Efficient use of the resolution of the identity approximation in time-dependent density functional calculations with hybrid density functionals *Chem. Phys. Lett.* **2002**, *362*, 170-178.
